# Selective Acetylation of Unprotected Thioglycosides and Fully Unprotected Monosaccharides with Candida antarctica Lipase‑B

**DOI:** 10.1021/acsomega.5c02467

**Published:** 2025-05-07

**Authors:** Kaarel Erik Hunt, Annette Miller, Tatsiana Jarg, Kadri Kriis, Tõnis Kanger

**Affiliations:** Department of Chemistry and Biotechnology, Tallinn University of Technology, Akadeemia tee 15, Tallinn 12618, Estonia

## Abstract

A selective enzymatic acetylation method for the protection of the second and the sixth positions of thio-d-galactopyranoside was found using immobilized Candida antarctica lipase-B (CAL-B). Unfortunately, it was determined that the immobilized enzyme cannot be recycled effectively. The optimized acetylation method was screened with different thioglycosides and with fully unprotected saccharides. New methods for several new partially protected saccharides were found, while the synthesis of some known saccharides, e.g., the third and the sixth position-protected d-glucose or the fourth position-protected l-rhamnose, was improved. Furthermore, an enzymatic acetal formation between the fourth and the sixth positions was discovered. The main limitation for acetylation reactions with CAL-B has been determined to be the substrate solubility.

## Graphical abstract

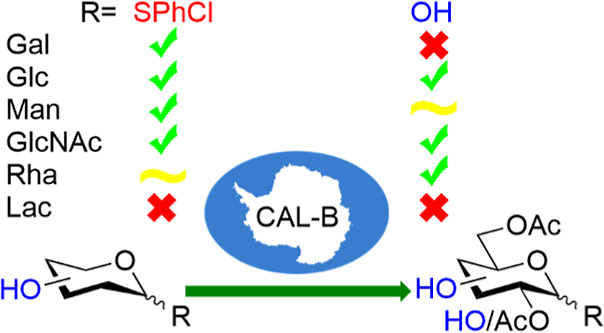

## Introduction

Regioselective protection and deprotection of functional groups in carbohydrate chemistry are vital parts of oligosaccharide synthesis. Generally, in total synthesis, several different protecting groups are used in multiple steps to arrive at the targeted specifically protected saccharide.^1–3^ Some positions of monosaccharides can be more easily protected than others.^4–6^ In pyranoses, the sixth position is a primary hydroxyl group and can be selectively protected and deprotected in high yields.^7–10^ Chemical regioselective modifications in the second to fourth positions, which are all secondary hydroxyl groups, are more complicated. It can be achieved by relying on steric interactions, metal complexes, cyclic protecting groups, and shifting reaction conditions toward the formation of the kinetic or thermodynamic product.^11–18^ Enzymatic methods for the selective protection and deprotection mostly cover ester-protecting groups, mainly in the sixth or the first position.^19,20^ Candida antarctica lipase-B (CAL-B) has been shown to work in organic media while exhibiting a good regioselectivity in a wide range of substrates.^21^ We have previously used immobilized CAL-B, Novozyme N435, for the regioselective deacetylation of monosaccharides in organic media.^22,23^ As for selective acetylation, Riva et al. (1997)^24^ have shown that when the anomeric position of various α-monosaccharides is protected, CAL-B can selectively acetylate the sixth position in organic media with 74–99% yield (Scheme 1A), while β-anomers depend more on the anomeric protecting group and can have either the sixth position or the sixth and the second/third positions acetylated, resulting in a mixture of products. Following on their work, Holmstrøm and Pedersen (2020)^25^ increased the selectivity toward the third and sixth position acetylations with various anomerically protected monosaccharides (Scheme 1B).

**Scheme 1:**
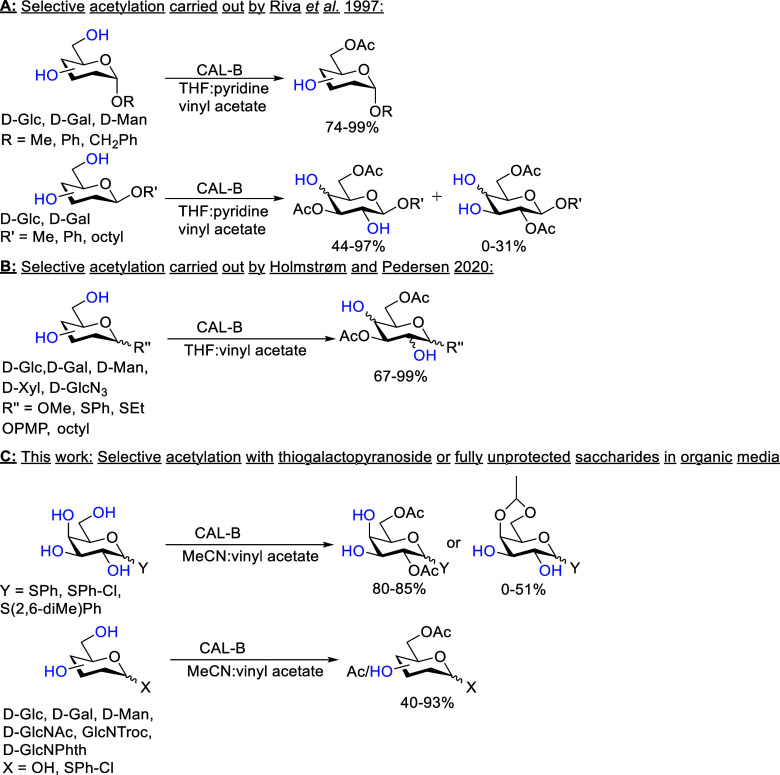
(A) Riva et al. (1997)^24^ Showed That Anomerically Protected Saccharides React Differently Based on the Anomeric Configuration, (B) Holmstrøm and Pedersen (2020)^25^ Focused More on the Selective Acetylation of 3,6-*O*-diAc Products, (C) This Work Focuses on 2,6-*O*-diAc Formation for Thio-d-galactopyranosides and Selective Protection of Other Thioglycosides and Fully Unprotected Saccharides

Here, we report a regioselective acetylation of the second and the sixth positions with different d-galactopyranose thioethers (Scheme 1C). Resulting products are soluble in organic media with the third and the fourth positions left unprotected, making them useful intermediates for the synthesis of various natural oligosaccharides, including galactooligosaccharides and human milk oligosaccharides.^26–28^ In addition, we have also found conditions for the selective enzymatic synthesis of an acetal between the fourth and the sixth positions, with thio-d-galactopyranoside leaving the second and the third positions unprotected. Furthermore, we report here using the optimized acetylation method to synthesize selectively protected saccharides from fully unprotected pyranose monosaccharides (d-glucose, d-glucosamine, d-mannose, l-rhamnose) and their thioether counterparts. To our knowledge, fully unprotected monosaccharides have not been selectively acetylated in organic media. Finally, some limitations for the acetylation reactions with CAL-B were discovered.

## Results and Discussion

First, we investigated the role of solvent in the selectivity of acetylation. The intention was to obtain selectively protected diols in the second and the sixth positions (Table 1). So far, 2,6-diprotected monosaccharides have been obtained via multistep synthesis^29^ or via separation from other regioisomers.^25^ CAL-B, like other lipases, is an enzyme that has been used in diverse media, like water, organic solvents, mixtures of various solvents, etc. The main reason is that the hydrophobic component in the solvent system is required for CAL-B to adopt the active form.^21^ Clearly, solvent influences the conformation of the active site pocket of CAL-B.^30,31^ Unprotected thio-d-galactopyranoside **1** or **2** was used as a model substrate (Scheme 2). Both thiophenol and *p*-chlorothiophenol pyranosides were tested, with no differences observed in the acetylation reaction with CAL-B. The latter group was still preferred as the less foul-smelling protecting group. Immobilized CAL-B was used as a weight ratio with the substrate as the method of choice following previous examples^24,25,32^ instead of mmol and enzyme activity units. The w/w method was used to investigate only the regioselectivity pattern of the acetylation reaction. To compare reactivities with different molecular weight saccharides, a molar ratio to enzyme activity units should be used. First, Riva et al.’s^24^ conditions were tested with thio-d-galactopyranoside **1**. Equal amounts of monosaccharide and immobilized enzyme, CAL-B (100% (w/w)), were stirred in the mixture of tetrahydrofuran (THF)/pyridine (4:1) with 20 equiv of vinyl acetate as the acylating reagent at 45 °C (Scheme 2, Table 1 no. 1). The main outcome of the reaction was a mixture of different monoacetates. Different solvents were screened where it was known CAL-B has high activity^26^ and/or were polar solvents. Changing THF to methyl *tert*-butyl ether (MTBE), which was the best solvent for the deacetylation,^22^ resulted in a ∼ 2.5:1 mixture of 2,6-*O*-diAc **3** and 3,6-*O*-diAc **5** isomers (Table 1, no. 2). Pyridine has been shown to inhibit enzymatic activity,^24^ so reactions were performed in pure chloroform or pure MTBE. Surprisingly, these reactions gave, under unoptimized conditions, a single diastereomer of an ethylidene acetal between the fourth and sixth positions (product **7**) in around 50% yield (Table 1, nos. 3 and 4). Acetals are commonly used protecting groups in oligosaccharide synthesis, although rarely synthesized by enzymatic methods other than glycosidic bond formation. Acetaldehyde needed for acetal formation is a byproduct of the transesterification, but it is also formed via the hydrolysis of vinyl acetate even under dry conditions.^33^ To confirm, a reaction with thioglycoside **2** in chloroform and vinyl acetate without CAL-B was run, and it showed no acetal formation. Thus, a new enzymatic method was found for 4,6-*O*-ethylidene acetal formation with vinyl acetate. In further screening of solvents, we found that only traces of the desired 2,6-*O*-diAc product **3** were formed with both vinyl acetate and acetic anhydride as acylating reagents running the reaction in acetone (Table 1, nos. 5 and 6). Acylation with acetic anhydride in MeCN favored the formation of the 3,6-*O*-diAc isomer **6** (Table 1, no. 7). Its selective synthesis has been described previously.^25^ Replacing acetic anhydride with vinyl acetate changed the regioselectivity of the acetylation, selectively affording 2,6-*O*-diAc product **4** in high yield (72%) in 48 h (Table 1, no. 8). However, there were some unreacted starting materials and monoacetates still present. It is supposed that the acylating reagent as a cosolvent influences the conformation of the active site pocket of CAL-B as well and thus the regioselectivity.^30,29,25^ Increasing the amount of vinyl acetate in THF gave high selectivity toward the wanted 2,6-*O*-diAc **6** (Table 1, no. 9). The reaction time was considerably long (96 h) as only 20% CAL-B was used. The selectivity was increased further, and the reaction time decreased to 48 h by switching THF to MeCN, which had shown the best results with a lower amount of vinyl acetate, still with 20% CAL-B (Table 1, no. 10). Increasing the amount of CAL-B to 100% decreased reaction time to 24 h, and diacetate **4** was isolated in 80% yield (Table 1, no. 11). (For full optimization, see Table S1 in the SI.) Finally, the background reaction was investigated. CAL-B was omitted from the reaction mixture, and no acetylation occurred in 96 h (Table 1, no. 12).

**Table 1:** Solvent Screening for Acetylation with Thio-d-galactopyranosides **1** and **2** with CAL-B^a^

no.	S.M.	solvent	acylation reagent	time (h)	products yield (%)^b^			
					mono-OAc	**3** or **4**	**5** or **6**	7
1	**1**	THF/pyridine 4:1	Vin.Ac	168	mix	traces	traces	
2	**1**	MTBE/pyridine 4:1	Vin.Ac	48	traces	49	20	
3	**1**	chloroform	Vin.Ac	72	traces			51
4	**1**	MTBE	Vin.Ac	72	traces			49
5	**1**	acetone	Vin.Ac	96	mix	traces	traces	
6	**1**	acetone	Ac_2_O	96	mix	traces	traces	
7	**2**	MeCN	Ac_2_O	48	mix	18*	50*	
8	**2**	MeCN	Vin.Ac	48	traces	72		
9^c^ ^,^ ^d^	**2**	THF	Vin.Ac	96	traces	83* (56)	17*	
10^c^ ^,^ ^d^	**2**	MeCN	Vin.Ac	48		92* (57)	8*	
11^c^	**2**	MeCN	Vin.Ac	24		93* (80)	7*	
12^c^ ^,^ ^e^	**2**	MeCN	Vin.Ac	96				

a Reaction condition: thioglycoside **1** or **2** (60 mg), vinyl acetate (20 equiv), solvent (5 mL), CAL-B (60 mg), 45 °C.

b Isolated yield shown or in ().

c Ratio of solvent:vinyl acetate 1:1 (2.4 mL).

d 20% w/w CAL-B used.

e Reaction without CAL-B; S.M.—starting material, mix—mixture of products, not isolated or characterized; *—determined by ^**1**^H NMR from the crude mixture.

**Scheme 2:**
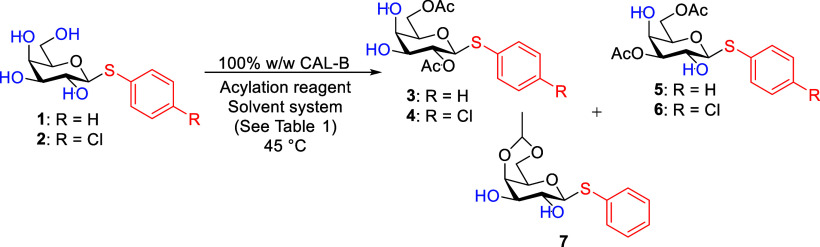
Acetylation of Thio-d-galactopyranosides **1** and **2** with CAL-B Resulting in 2,6-*O*-diAc (Compounds **3** and **4**) and 3,6-*O*-diAc (Compounds **5** and **6**) Regioisomers or Formation of 4,6-*O*-ethylidene Acetal **7**

Previously, it has been shown that Novozyme 435 can be recycled successfully in the deacetylation reactions.^23^ Three cycles with thio-d-galactopyranoside **2** were conducted using the same recycling conditions (Table S2). After each reaction, the immobilized enzyme was washed with dichloromethane (DCM) (∼50 mL) and air-dried for 1 h. The first cycle resulted once again in an 80% isolated yield but started to fall after the second cycle in 50% yield. By the end of the third cycle, monoacetylated products started to dominate; some starting material was leftover, and the yield of 2,6-*O*-diAc **4** decreased further to 42%. We surmise that the enzyme or the resin was acetylated, which led to either the enzyme deactivation or inhibited the starting material from reaching the catalytic site.

Next, the acetylation of thioglycosides of various monosaccharides was studied (Scheme 3). When the leaving group in d-galactose was changed to 2,6-dimethyl thiophenol (compound **8**), the reaction efficiency decreased, but the regioselectivity of the reaction remained high. Increased amounts of enzyme, higher temperature, and longer reaction time were needed to get to 2,6-*O*-diAc product **9** in a similar yield (85%) as with thioglycosides **1** and **2**. It is known that changing the stereochemistry of monosaccharides, i.e., switching from one sugar to another, drastically influences regioselectivity and reactivity. That was proven once again as thio-β-d-glucopyranoside (compound **10**) had reached full conversion in just 30 min with 80% yield of the sixth position acetylated product **11**. The results obtained with thioglycoside epimers (d-galactose and d-glucose) **2** and **10** showed that the fourth position influences mainly the rate of the reaction as well as whether the acetylation reaction is producing a diacetate or a monoacetate. Thio-d-glucopyranosamine substrates **12** and **14** reacted for 4 h with acetylation occurring in the sixth position, resulting in products **13** and **15**. The reaction with the *N*-acetyl protecting group (compound **12**) was slightly less selective, affording monoacetate **13** in 71% yield with unselective overreaction products and starting material left, while phthalate had a very high selectivity, and 91% yield was achieved after product **15** was purified by crystallization (Scheme S2). d-Glucose-based compounds (**10**, **12**, and **14**) all reacted similarly; it seems that the amide in the second position does not influence regioselectivity but slightly lowers the rate of the reaction. Thio-d-mannopyranoside **16** gave selectively the sixth position β-product **17** in 76% yield in 20 h. The α-anomer of **16** did not react selectively, giving several products. The reaction with thio-d-mannopyranose **16** further showed the influence of the second position as comparing thioglycoside epimers (d-glucose and d-mannose) **10** and **16**, the rate of the reaction decreased 40 times. Acetylation of thio-l-rhamnopyranoside **18** resulted in the mixture of three different monoacetates **19**–**21**. Only 4-*O*-Ac **21** was managed to be isolated separately from this mixture. d-Lactose with *p*-chlorothiophenol leaving group **22** did not react at all. We surmise that it is due to solubility issues.

**Scheme 3:**
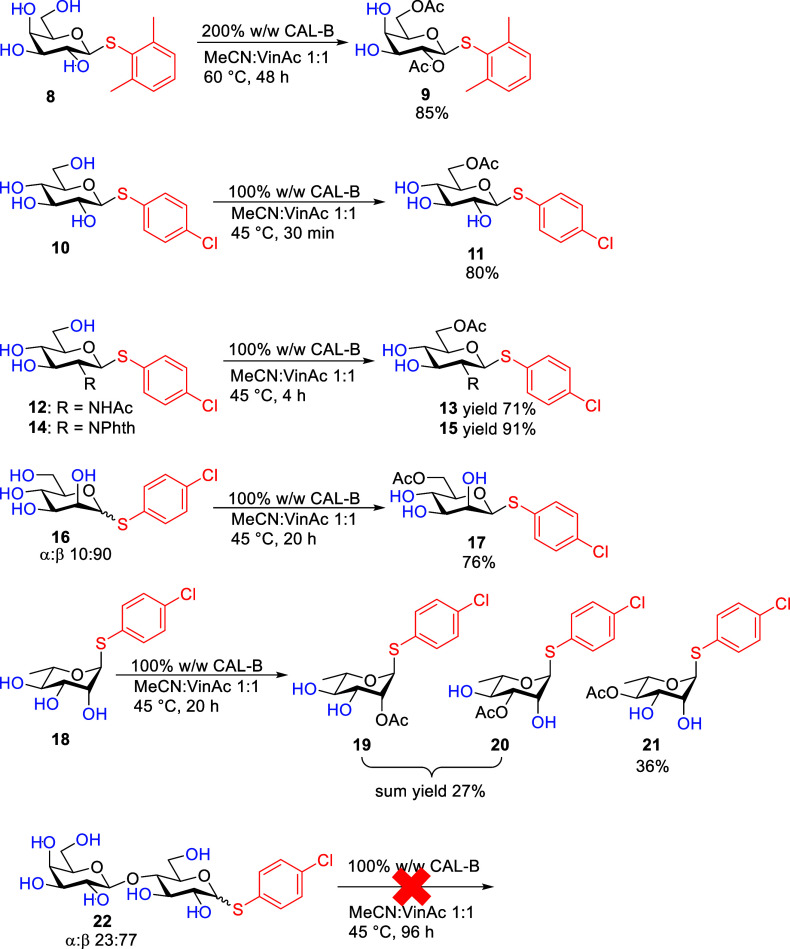
CAL-B Acetylation Reactions with Thioglycosides, Where: Thio-d-Galactose, d-Glucose, d-Glucosamine, and d-Mannose Gave Selective Acetylation Reactions; Thio-l-Rhamnose, a Mixture of Monoacetylated Products, and Thio-d-Lactose Did Not React^a^

a Reaction conditions: thioglycoside (60–100 mg), MeCN:vinyl acetate 1:1 (0.08 M of substrate), 100–200% w/w CAL-B, 45–60 °C; yields shown are isolated yields.

Selective derivatization of fully unprotected saccharides is a challenge because of their poor solubility in organic solvents. The solubility greatly influences the reaction outcome of unprotected saccharides and is the main limitation in CAL-B acetylation reactions.

Fully unprotected d-glucopyranose **23** reacted slowly with vinyl acetate in the presence of CAL-B (Scheme 4). Still, the reaction was highly selective, affording 3,6-*O*-diAc isomer **24** in 91% yield. Both increasing the temperature and the amount of enzyme led to a decrease in yield and still needed 3 days of reaction time to reach full conversion (Scheme S1). Unprotected d-galactopyranose **25** did not react selectively and led to a mixture of products. Some compounds were characterized, but they remained in mixtures (Table S3). Surprisingly, the corresponding thioglycosides (**1**, **2**, and **8**) reacted very selectively. For the d-glucopyranosamine series, solubility seemed to influence the outcome. Hydrochloric salt of d-glucosamine and phthalate-protected compounds had very poor solubility and did not react at all after 3 days (Scheme S2). Acetyl-protected glucosamine **26** reacted slowly and needed harsher conditions to get the sixth position-protected product **27** in 40% yield in 3 days, while 2,2,2-trichloroethoxycarbonyl (Troc)-protected glucosamine **28** reacted in just 2 h in 81% yield toward the sixth position-protected product **29**. The large difference in the rate of the reactions between the two d-glucopyranosamine compounds can be attributed to solubility. Similar to the thioglycoside counterparts (**12** and **14**), only the sixth position was acetylated. d-Mannopyranose **30** showed limited selectivity, giving a diacetate fraction with 81% yield including 1,6-*O*-diAc **31** (13%, α-anomer characterized only), 2,6-*O*-diAc **32** (63%, α/β 88:12), and 3,6-*O*-diAc **33** (24%, α-anomer characterized only). Some minor uncharacterized peaks most likely correspond to β-anomers of products **31** and **32**. It is contrary to thio-d-mannopyranoside **16**, which gave selectively the sixth position acetylation. The unprotected first position seems to allow selective diacetate formation for both epimers, d-glucopyranose and d-mannopyranose (compounds **23** and **30**), while the second position’s configuration determines the regioselectivity. l-Rhamnopyranose **34** gave a slow reaction as well but with very high selectivity toward the fourth position acetylation with a 93% isolated yield of product **35**. The fourth position-acetylated l-rhamnopyranose had been previously synthesized in four steps, starting with benzyl α-l-rhamnopyranoside.^34,35^ Similar to the thioglycoside of lactose, fully unprotected d-lactose **36** did not react within 4 days, most probably because of solubility issues.

**Scheme 4:**
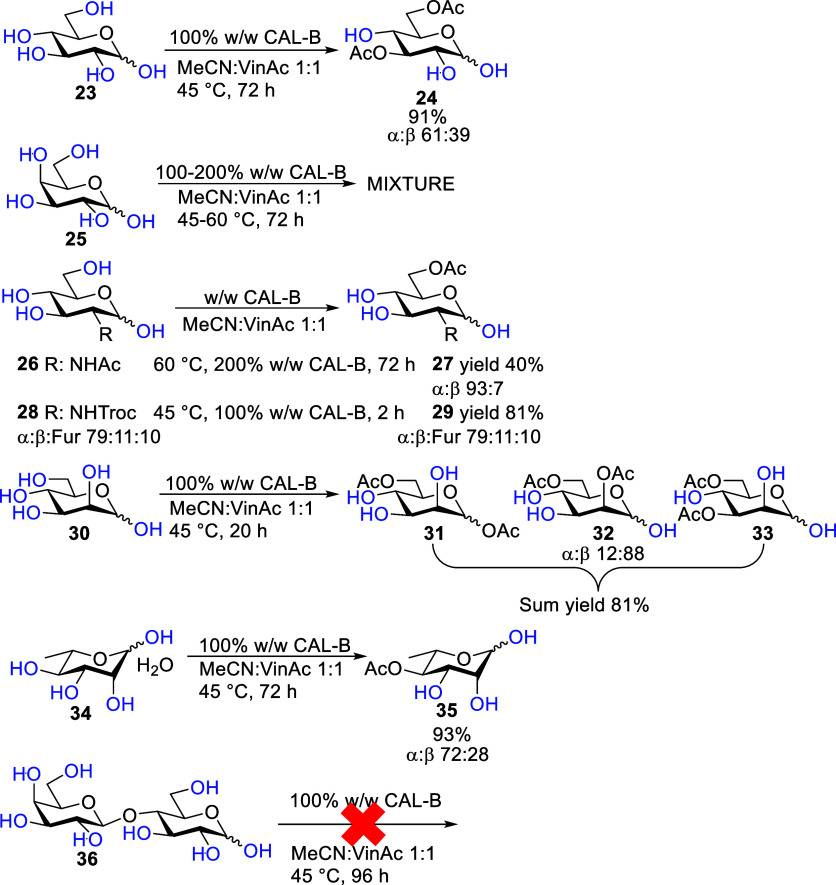
CAL-B Acetylation Reactions with Fully Unprotected Saccharides, Where: d-Glucose, d-Glucosamines, and l-Rhamnose Gave Selective Acetylation Products; d-Mannose, a Mixture of Diols; d-Galactose an Unpurifiable Mixture; and d-Lactose Did Not React^a^

a Reaction conditions: saccharide (60–100 mg), MeCN/vinyl acetate 1:1 (0.08 M of substrate), 100–200% w/w CAL-B, 45–60 °C; yield shown are isolated yields.

Chemically selective acetylation occurs most often in the primary position of the target saccharide. Generally, it is followed by full protection of the saccharide and purification. In most cases, if partially protected sugar is needed, the anomeric position is protected by either a thio or methoxy group. Furthermore, high-boiling-point solvents like pyridine and dimethylformamide are used, which complicate the workup procedures; reactions are generally overnight if not longer, and high temperatures are used, e.g., with trityl chloride up to 100 °C.^36^ While in our case most thioglycosides were also protected in the sixth position, the workup is much simpler and reaction times shorter with consistently high yields. For unprotected sugars, acetyl protecting groups cannot be selectively added via chemical means as the first position would be protected as well.

## Conclusions

In conclusion, starting from thio-d-galactopyranoside, a new enzymatic acetylation method for the synthesis of the second and the sixth position-protected diacetate using CAL-B was found and optimized. During optimization, an unprecedented enzymatic acetal-forming method between the fourth and the sixth positions was also discovered. Unfortunately, recycling tests showed that CAL-B cannot be recycled as the yield dropped from 80% to 42% after the third cycle. Several new partially protected thioglycosides were synthesized while testing the CAL-B acetylation reaction. Furthermore, fully unprotected saccharides were used as substrates and acetylated selectively. For known compounds, the previously known synthesis pathways were shortened. To summarize, we have shown that CAL-B can be used to synthesize partially protected saccharides, which can be used further in the total synthesis of natural oligosaccharides.

## Experimental Section

### General Experimental Information

Full assignment of ^1^H and ^13^C chemical shifts was based on the 1D and 2D (COSY, HSQC, and HMBC) FT NMR spectra measured with a Bruker AVANCE III 400 MHz instrument. Residual solvent signals were used (CDCl_3_: δ = 7.26 ^1^H NMR, 77.2 ^13^C NMR; CD_3_OD: δ = 3.31 ^1^H NMR, 49.0 ^13^C NMR; (CD_3_)_2_SO: δ = 2.50 ^1^H NMR, 39.5 ^13^C NMR; D_2_O: δ = 4.79 ^1^H NMR) as internal standards. High-resolution mass spectra were recorded with an Agilent Technologies 6540 UHD Accurate-Mass QTOF LC/MS spectrometer by using AJ-ESI ionization. Prior to analysis, the instrument was calibrated in a mass range of *m*/*z* 50–3200. Optical rotations were obtained with an Anton Paar GWB Polarimeter MCP 500. Melting points were determined using a NAGEMA-K8 polarizing optical microscope. Precoated Merck silica gel 60 F_254_ plates were used for TLC, and column chromatography was performed with Merck 60 (0.040–0.063 mm) mesh silica gel. Commercial reagents and solvents were generally used as received. DCM was distilled over CaH or phosphorus pentoxide, ethyl acetate (EtOAc) and acetone over phosphorus pentoxide, and MeOH and toluene over sodium. Petroleum ether (PE) had a boiling point of 40–60 °C. Silicon oil bath on top of a magnetic stirrer with heating was used as a heat source for reactions requiring heating. Immobilized C. antarctica lipase-B, Novozyme N435, with 10,000 (propyl laurate unit/g), was a kind gift from Novozymes A/S.

#### General Procedure for Preparation of 4-Chlorophenyl 2,6-Di-*O*-acetyl-1-thio-β-d-galactopyranoside **4** with CAL-B

4-Chlorophenyl 1-thio-β-d-galactopyranoside **2** (60 mg, 0.196 mmol) was dissolved in MeCN/vinyl acetate 1:1 (2.4 mL), heated to 45 °C, and dissolved or stirred for 10 min. Stirring was set to 100 rpm, and CAL-B (60% w/w, 36 mg) was added. The reaction vessel was equipped with an air cooler with a CaCl_2_ tube. The reaction was followed by TLC (DCM/EtOAc 1:2, *R*_f_ = 0.34), and upon completion, the reaction mixture was filtered. Immobilized enzymes were washed with DCM (∼50 mL), and the filtrate was concentrated in vacuo. The crude mixture was purified by silica gel column chromatography (PE/EtOAc 2:3 → 1:9), yielding a white solid (61 mg, 80%); mp 134–137 °C (from DCM); [α]_D_^20^ + 7.1 (acetone, *c* 0.08); ^1^H NMR (400 MHz, CDCl_3_): δ 7.41–7.46 (m, 2H), 7.24–7.30 (m, 2H), 4.98 (t, *J* = 9.7 Hz, 1H, H-2), 4.56 (d, *J* = 10.0 Hz, 1H, H-1), 4.36 (dd, *J* = 5.6, 11.7 Hz, 1H, H-6*a*/6b), 4.29 (dd, *J* = 7.0, 11.7 Hz, 1H, H-6*a*/6b), 3.95 (d, *J* = 3.2 Hz, 1H, H-4), 3.70 (t, *J* = 6.7 Hz, 1H, H-5), 3.68 (dd, *J* = 3.3, 9.4 Hz, 1H, H-3), 3.07 (s, 2H, OH-3,4), 2.15 (s, 3H), 2.08 (s, 3H); ^13^C­{^1^H} NMR (101 MHz, CDCl_3_): δ 171.3, 171.3, 134.4, 134.0 (2xC), 131.1, 129.1 (2xC), 85.9 (C-1), 76.2 (C-5), 73.4 (C-3), 71.0 (C-2), 68.9 (C-4), 63.1 (C-6), 21.2, 21.0; HRMS (AJS-ESI): [M + Na]^+^ for C_16_H_19_ClO_7_SNa^+^, 413.0432; found, 413.0426.

Note: there is a small amount of leaching of resin occurring during the DCM wash.

#### Mmol-Scale Synthesis of 2,6-Dimethylphenyl 2,6-Di-*O*-acetyl-1-thio-β-d-galactopyranoside **9** with CAL-B

According to the general procedure with 2,6-dimethylphenyl 1-thio-β-d-galactopyranoside **8** (1.78 mmol, 534 mg), MeCN (11 mL), vinyl acetate (11 mL), 60 °C, and 200% CAL-B (1068 mg), the reaction was run for 48 h, resulting in a white solid (581 mg, 85%). TLC—DCM/EtOAc 1:4, *R*_f_ = 0.52; column chromatography eluent system DCM/acetone 0% → 15% acetone; mp 190–194 °C (from DCM); [α]_D_^20^ + 41.5 (acetone, c 0.09); ^1^H NMR (400 MHz, MeOD): δ 7.09–7.16 (m, 3H), 5.14 (t, *J* = 9.8 Hz, 1H, H-2), 4.35 (d, *J* = 10.2 Hz, 1H, H-1), 4.31 (dd, *J* = 8.2, 11.5 Hz, 1H, H-6*a*/6b), 4.08 (dd, *J* = 4.2, 11.5 Hz, 1H, H-6*a*/6b), 3.84 (ap.d, *J* = 3.0 Hz, 1H, H-4), 3.65 (dd, *J* = 3.4, 9.5 Hz, 1H, H-3), 3.58 (ddd, *J* = 0.8, 4.2, 8.1 Hz, 1H, H-5), 2.53 (s, 6H, 2xCH_3_), 2.16 (s, 3H), 1.94 (s, 3H); ^13^C­{^1^H} NMR (101 MHz, MeOD): δ 172.4, 172.1, 145.20 (2xC), 133.0, 130.2, 129.2 (2xC), 90.4 (C-1), 77.6 (C-5), 74.0 (C-3), 72.7 (C-2), 70.5 (C-4), 64.9 (C-6), 22.7 (2xC), 21.1, 20.7; HRMS (AJS-ESI): [M + Na]^+^ for C_18_H_24_O_7_SNa^+^, 407.1135; found, 407.1131.

## Supplementary Material



## Data Availability

The data underlying this study are available in the published article and its Supporting Information.

## Notes

Experimental procedures, characterization data, and NMR spectra for all synthesized compounds (PDF)
