# Supplementary material for: Selective Acetylation of Unprotected Thioglycosides and Fully Unprotected Monosaccharides with Candida antarctica Lipase‑B
Source: ACS Omega. 2025 May 7;10(19):20047–53. doi: 10.1021/acsomega.5c02467 (PMC12096258; doi:10.1021/acsomega.5c02467)
Supplement: Supplementary file 1 [file ao5c02467_si_001.pdf]

Supporting Information

Selective acetylation of unprotected thioglycosides and fully unprotected monosaccharides with *Candida antarctica* lipase-B

Kaarel Erik Hunt, Annette Miller, Tatsiana Jarg, Kadri Kriis and Tõnis Kanger*

Department of Chemistry and Biotechnology, Tallinn University of Technology, Akadeemia tee 15, 12618 Tallinn, Estonia.

Corresponding Author: Tõnis Kanger tonis.kanger@taltech.ee

Contents

Table S1: Further optimization with thioglycoside 2 ....S4

Scheme S1: Increasing temperature and amount of enzyme with D-glucose 23 in acetylation reactions....S5

Table S2: CAL-B recycling reaction with 4-chlorophenyl 1-thio-β-D-galactopyranoside 2 ....S6

Table S3: Compounds found with acetylation of D-galactose 25 with CAL-B S7

Scheme S2: Reactions of D-glucosamine HCl salt S1 and phthalate protected S2....S8

General Experimental Information....S9

Starting Material Synthesis S9

General Procedures S10

General procedure for acetylation reactions with CAL-B S10

Experimental procedures and characterisation of products....S10

Phenyl 2,6-di-*O*-acetyl-1-thio-β-D-galactopyranoside (3) S10

4-chlorophenyl 2,6-di-*O*-acetyl-1-thio-β-D-galactopyranoside (4) S10

Phenyl 3,6-di-*O*-acetyl-1-thio-β-D-galactopyranoside (5) S10

4-chlorophenyl 3,6-di-*O*-acetyl-1-thio-β-D-galactopyranoside (6) S11

Phenyl 4,6-*O*-ethylidene-1-thio-β-D-galactopyranoside (7) S11

2,6-dimethylphenyl 2,6-di-*O*-acetyl-1-thio-β-D-galactopyranoside (**9**) S11

4-chlorophenyl 6-*O*-acetyl-1-thio-β-D-glucopyranoside (**11**) S11

4-chlorophenyl 2-acetamido-6-*O*-acetyl-2-deoxy-β-D-glucopyranoside (13)..... S12

4-chlorophenyl 6-*O*-acetyl-2-deoxy-2-phthalimido-β-D-glucopyranoside (15) .. S12

4-chlorophenyl 1-thio-D-mannopyranoside (16) S12

4-chlorophenyl 6-*O*-acetyl-1-thio-β-D-mannopyranoside (17)........................... S13

4-chlorophenyl 6-deoxy-1-thio-α-L-mannopyranoside (18) S13

4-chlorophenyl 2-*O*-acetyl-6-deoxy-1-thio-α-L-mannopyranoside (19), **4-chlorophenyl 3-*O*-acetyl-6-deoxy-1-thio-α-L-mannopyranoside (20)** and **4-chlorophenyl 4-*O*-acetyl-6-deoxy-1-thio-α-L-mannopyranoside (21)**................. S13

4-chlorophenyl (β-D-galactopyranosyl)-(1→4)-1-thio-β-D-glucopyranoside (**22**).. S14

3,6-di-*O*-acetyl-D-glucopyranose (**24**) S14

2-acetomido-6-*O*-acetyl-2-deoxy-D-glucopyranose (**27**)...................................... S15

6-*O*-acetyl-2-deoxy-2-(2,2,2-trichloroethoxycarbonylamino)-D-glucopyranose (29) S15

1,6-di-*O*-acetyl-D-mannopyranoside (**31**), 2,6-di-*O*-acetyl-D-mannopyranose (**32**) and **3,6-di-*O*-acetyl-D-mannopyranose (33)**.. S15

4-*O*-acetyl-6-deoxy-L-mannopyranose (**35**) S16

NMR Spectra S17

Figure S1: Phenyl 1-thio-β-D-glucopyranoside (**1**) S17

Figure S2: 4-chlorophenyl 1-thio-β-D-glucopyranoside (**2**).................................... S18

Figure S3: Phenyl 2,6-di-*O*-acetyl-1-thio-β-D-galactopyranoside (**3**) S19

Figure S8: 4-chlorophenyl 2,6-di-*O*-acetyl-1-thio-β-D-galactopyranoside (**4**) S24

Figure S13: Phenyl 3,6-di-*O*-acetyl-1-thio-β-D-galactopyranoside (**5**) S29

Figure S18: Mixture, where dominant is **4-chlorophenyl 3,6-di-*O*-acetyl-1-thio-β-D-galactopyranoside** (**6**) S34

Figure S23: Phenyl 4,6-*O*-ethylidene-1-thio-β-D-galactopyranoside (**7**) S39

Figure S28: 2,6-dimethylphenyl 1-thio-β-D-galactopyranoside (**8**)......................... S44

Figure S29: 2,6-dimethylphenyl 2,6-di-*O*-acetyl-1-thio-β-D-galactopyranoside (**9**) S45

Figure S34: 4-chlorophenyl 1-thio-β-D-glucopyranoside (**10**) S50

Figure S35: 4-chlorophenyl 6-*O*-acetyl-1-thio-β-D-glucopyranoside (**11**) S51

Figure S40: 4-chlorophenyl 2-acetamido-2-deoxy-1-thio-β-D-glucopyranoside (**12**)S56

Figure S41: 4-chlorophenyl 2-acetamido-6-*O*-acetyl-2-deoxy-1-thio-β-D-glucopyranoside (**13**).. S57

Figure S46: 4-chlorophenyl 6-acetyl-2-deoxy-2-phthalimido-1-thio-β-D-glucopyranoside (**14**) S62

Figure S47: 4-chlorophenyl 6-acetyl-2-deoxy-2-phthalimido-1-thio-β-D-glucopyranoside (**15**).. S63

Figure S52: 4-chlorophenyl 1-thio-D-mannopyranoside (**16**) α:β 10:90 .... S68

Figure S57: 4-chlorophenyl 6-*O*-acetyl-1-thio-β-D-mannopyranoside (**17**) S73

Figure S62: 4-chlorophenyl 6-deoxy-1-thio-α-L-mannopyranoside (**18**) S78

Figure S67: 4-chlorophenyl 2-*O*-acetyl-6-deoxy-1-thio-α-L-mannopyranoside (**19**) shown from mixture S83

Figure S72: 4-chlorophenyl 3-*O*-acetyl-6-deoxy-1-thio-α-L-mannopyranoside (**20**) shown from mixture S88

Figure S74: 4-chlorophenyl 4-*O*-acetyl-6-deoxy-1-thio-α-L-mannopyranoside (**21**) S90

Figure S79: 4-chlorophenyl (β-D-galactopyranosyl)-(1→4)-1-thio-β-D-glucopyranoside (**22**).. S95

Figure S84: 3,6-di-*O*-acetyl-D-glucopyranose (**24**) α:β 61:39................................S100

Figure S88: 2-acetomido-6-*O*-acetyl-2-deoxy-D-glucopyranose (**27**) α:β 97:3 ....S104

Figure S92: 2-deoxy-2-(2,2,2-trichloroethoxycarbonylamino)-D-glucopyranose (**28**) α:β:Fur 79:11:10..S108

Figure S93: 6-*O*-acetyl-2-deoxy-2-(2,2,2-trichloroethoxycarbonylamino)-D-glucopyranose (29)..S109

Figure S98: Mixture, where **1,6-di-*O*-acetyl-D-mannopyranoside (31**) is shown...S114

Figure S103: Mixture, where **2,6-di-*O*-acetyl-D-mannopyranose (32**) is shown α:β 12:88 ....S119

Figure S105: Mixture, where **3,6-di-*O*-acetyl-D-mannopyranose (33**) is shown....S121

Figure S107: 4-*O*-acetyl-6-deoxy-L-mannopyranose (**35**) S123

References S127

Table S1: Further optimization with thioglycoside 2

When increasing the amount of CAL-B in the acetylation reaction with thioglycoside **2** from 20% to 40% decreased the reaction time to 24 h, but there were still some traces of starting material left (Table S1, no. 1, 2). 60% CAL-B showed full conversion overnight with the same product ratios as 20% and 40% CAL-B reactions and the product **4** was successfully purified by crystallization with 72% yield (Table S1, no. 3). The last step of all the unprotected thioglycoside starting material synthesis is Zemplén deacetylation (See Starting Material Synthesis). Sodium methoxide used in Zemplén deacetylation is generally neutralised by acid resin, but due to colour leaching, acetic acid and dry ice were decided to be used as alternatives. As such, 2 equiv of NaOAc and 2 equiv of Na_2CO_3 were added as additives (Table S1, no. 4, 5). There was no difference when Na_2CO_3 was used as additive, but NaOAc slowed the reaction slightly and increased the amount of 3,6-*O*-Ac isomer **6**. Thus, thioglycoside starting materials contain small amounts of Na_2CO_3 impurities as dry ice is used to neutralise sodium methoxide. Increasing the amount of CAL-B to 100% did not decrease the reaction time and showed the same product ratios (Table S1, no. 6). Both increasing and decreasing the reaction concentration led to decrease in selectivity (Table S1, no. 7, 8).

*Table S1 Further optimization of acetylation reaction with thioglycoside **2** and CAL-B[a]*

No	Time (h)	w/w CAL-B (%)	Products Ratio **4:6** (%)[b]	Yield[c] (%)
1	48	20	92:8	57
2[d]	24	40	94:6	-
3	24	60	93:7	72[e]
4[f]	24	60	72:28	-
5[g]	24	60	93:7	-
6	24	100	93:7	80
7[h]	24	100	74:26	-
8[i]	24	100	83:17	-

[a] reaction conditions: thioglycoside **2** (60 mg), MeCN:vinyl acetate 1:1 (2.4 mL), w/w CAL-B, 45 °C; [b] ratio based on ^1H NMR analysis; [c] isolated yield of product **4**; [d] traces of starting material seen in ^1H NMR; [e] purified by crystallization with Pe:EtOAc; [f] 2 equiv of NaOAc added and 8% of monoacetylated side product; [g] 2 equiv of Na_2CO_3 added; [h] MeCN:VinAc 1:1 (4.8 mL); [i] MeCN:VinAc 1:1 (1.2 mL)

Scheme S1: Increasing temperature and amount of enzyme with D-glucose 23 in acetylation reactions

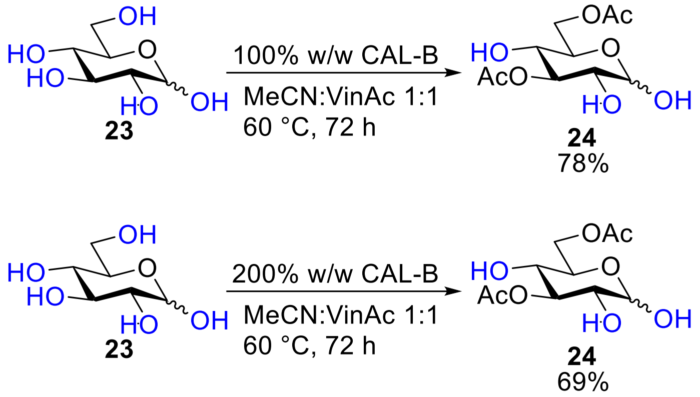

*Scheme S1 Acetylation reaction with D-glucose **23** and CAL-B*

In order to decrease the reaction time, temperature was increased to 60 °C from 45 °C. Unfortunately, not only the time remained the same, but the selectivity also decreased thus decreasing the isolated yield as well from 91% to 78%. Increase of the amount of enzyme led to same consequences as the time remained the same and isolated yield decreased even further to 69%.

Table S2: CAL-B recycling reaction with 4-chlorophenyl 1-thio-β-D-galactopyranoside 2

CAL-B acetylation reactions were carried out according to general procedure (see below p S10) with 4-chlorophenyl 1-thio-β-D-galactopyranoside **2** (100 mg x 3) and 60 mg CAL-B. After each reaction was complete, the reaction mixture was filtered using glass filter and CAL-B washed with ~50 mL DCM. The filtrate was concentrated in vacuo, analysed by NMR and purified by silica gel column chromatography. Isolated yields of product **4** together with the inseparable mixture of isomers **4** and **5** are shown in Table S2 and more detailed information about the product is shown in the "Experimental procedures and characterisation of products" (p S10). Filtered CAL-B was left on the filter to air-dry for 1 h and then used again straight away.

*Table S2 The outcomes of the recycling reactions with CAL-B and thioglycoside **2***

CAL-B Acetylation Reactions with 4-chlorophenyl 1-thio-β-D-galactopyranoside **2**			
	Cycle 1	Cycle 2	Cycle 3
Product **4** Yield (mg)	102	63	54
Product **4** Yield (%)	80	50	42
Mix of Isomers **4** & **5** Yield (mg)	19	26	17
Mix of Isomers **4** & **5** Yield (%)	15	20	13

Table S3: Compounds found with acetylation of D-galactose 25 with CAL-B

No one compound was successfully purified. The identity of compounds listed were confirmed by NMR. Most of the starting material did not react (~63%), while most prominent products were diacetates (~25%). Interestingly, quite a lot of products were furanoses (14%), which we have not seen with any other saccharides so far.

*Table S3 Products found from the acetylation reaction with D-galactose **25** with CAL-B*

Compound(s)	% of the mixture
2x unknown tetraacetates pyranose	1%
1,2,6-tri-*O*-acetyl-α-D-galactofuranoside	11%
2,6-di-*O*-acetyl-D-galactopyranoside	8%
3,6-di-*O*-acetyl-D-galactopyranoside	12%
2x unknown diacetates fyranose	3%
2x unknown diacetates pyranose	2%
Total	37%

Scheme S2: Reactions of D-glucosamine HCl salt S1 and phthalate protected S2

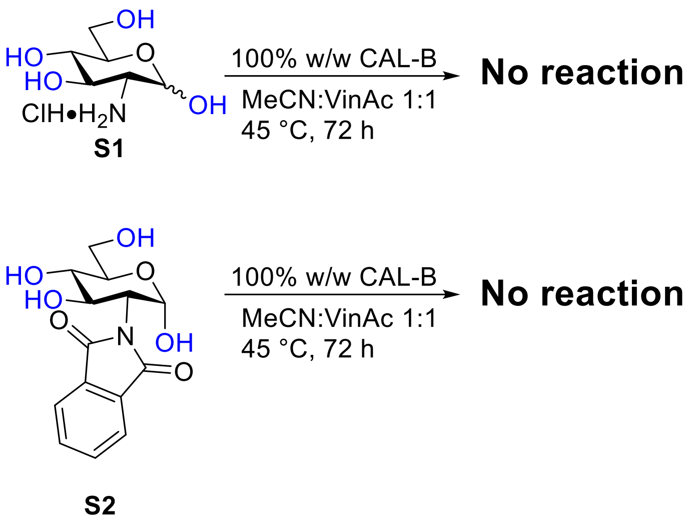

Scheme S2 Acetylation reactions with D-glucosamine HCl salt and with phthtalate protecting group

Both substrates did not show any signs of dissolving, nor did they have any products forming according to TLC analysis. Samples for TLC were dissolved either in MeOH or in H_2O and then applied to TLC plate. As there was nothing happening in 3 days, reactions were stopped. Further reactions with increased temperature and amount of enzyme were not carried out as was the case with acetyl protected D-glucosamine **26**, which did have some conversion with 45 °C and 100% w/w CAL-B.

General Experimental Information

Full assignment of ^{1}H and ^{13}C chemical shifts were based on the 1D and 2D (COSY, HSQC, HMBC) FT NMR spectra measured with a Bruker Avance III 400 MHz instrument. Residual solvent signals were used ($CDCl_3$: δ = 7.26 ^{1}H NMR, 77.2 $^{13}C\{^{1}H\}$ NMR; CD_3OD: δ = 3.31 ^{1}H NMR, 49.0 $^{13}C\{^{1}H\}$ NMR; $(CD_3)_2SO$: δ = 2.50 ^{1}H NMR, 39.5 $^{13}C\{^{1}H\}$ NMR; D_2O: δ = 4.79 ^{1}H NMR) as internal standards. High-resolution mass spectra were recorded with an Agilent Technologies 6540 UHD Accurate-Mass QTOF LC/MS spectrometer by using AJ-ESI ionisation. Prior to analysis the instrument was calibrated in the mass range of m/z 50–3200. Optical rotations were obtained with an Anton Paar GWB Polarimeter MCP 500. Melting points were determined using polarising optical microscope Nagema-K8. Precoated Merck silica gel 60 F_{254} plates were used for TLC and column chromatography was performed with Merck 60 (0.040–0.063 mm) mesh silica gel. Commercial reagents, and solvents were generally used as received. DCM was distilled over CaH or phosphorous pentoxide, ethyl acetate (EtOAc) and acetone over phosphorus pentoxide, MeOH and toluene over sodium. Petroleum ether (PE) had a boiling point of 40-60 °C. Silicon oil bath on top of magnetic stirrer with heating was used as a heat source for reactions requiring heating. Immobilised *Candida antarctica* Lipase-B on hydrophobic acrylic resin, Novozyme N435, with 10000 (propyl laurate unit/g) activity was a kind gift from Novozymes A/S.

Starting Material Synthesis

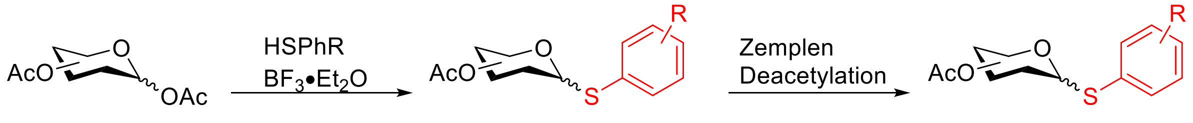

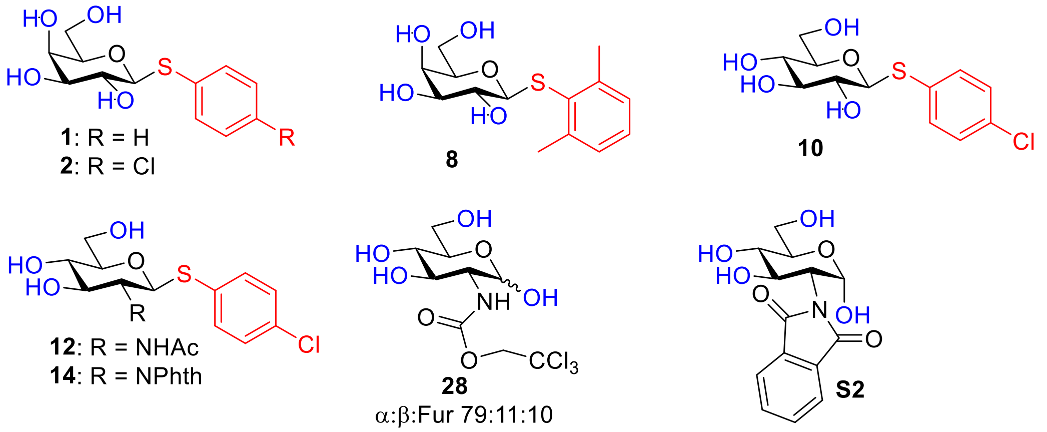

Starting materials were synthesised by first attaching the thio leaving group[1] followed by Zémplen deacetylation[2]. Thiogalactopyranosides (**1**[3], **2**[4] and **8**[5]), thioglucopyranoside **10**[6], thioglucopyranosamines (**12**[7] and **14**[4]), Troc-protected glucosamine **28**[8] phthalate-protected glucosamine **S2**[9] NMRs match with previously reported values. Commercially available saccharides (**23**, **25**, **26**, **30**, **34**, **36, S1**) were used as received.

General Procedures

General procedure for acetylation reactions with CAL-B

Unprotected saccharide or thiosaccharide (60 or 100 mg) was dissolved in MeCN:vinyl acetate 1:1 (0.08 M to substrate), heated up to 45 or 60 °C and stirred for 10 min. Stirring was set to 100 rpm and CAL-B (20-200% w/w) was added. The reaction vessel was equipped with air-cooler or water-cooler with $CaCl_2$ tube. The reaction was followed by TLC and upon completion, the reaxtion mixture was filtered. Immobilized enzymes were washed with DCM or MeOH or MeOH/H_2O (~50 mL) and the filtrate was concentrated in vacuo. The crude mixture was purified by silica gel column chromatography (PE:EtOAc 2:1 →1:4) unless specified otherwise. Note: there is a small amount of leaching of resin occurring during DCM wash.

Experimental procedures and characterisation of products.

Phenyl 2,6-di-*O*-acetyl-1-thio-β-D-galactopyranoside (3)

According to a general procedure with phenyl 1-thio-β-D-galactopyranoside **1** (1.25 mmol, 340 mg), MTBE (22.67 mL), pyridine (5.67 mL), vinyl acetate (2.35 mL, 20.4 equiv), 45 °C and 100% CAL-B (340 mg). Reaction was run for 24 h resulting in white solid 179 mg (40%). TLC – DCM:EtOAc 1:2, R_f = 0.38; column chromatography eluent system DCM:EtOAc 2:3 → 1:1; m.p. 103-106 °C (from DCM); $[\alpha]_D^{20}$ +9.2 (acetone, c 0.11);
^{1}H NMR (400 MHz, $CDCl_3$) δ 7.46-7.53 (m, 2H), 7.26-7.34 (m, 3H), 5.00 (t, *J* = 9.7 Hz, 1H, H-2), 4.62 (d, *J* = 10.0 Hz, 1H, H-1), 4.38 (dd, *J* = 5.8, 11.6 Hz, 1H, H-6a/6b), 4.31 (dd, *J* = 7.0, 11.6 Hz, 1H, H-6a/6b), 3.95 (d, *J* = 3.0 Hz, 1H, H-4), 3.72 (dt, *J* = 0.5, 6.4 Hz, 1H, H-5), 3.68 (dd, *J* = 3.4, 9.4 Hz, 1H, H-3), 2.86 (br.s, 2H, OH-3,4), 2.16 (s, 3H), 2.08 (s, 3H); ^{13}C{^{1}H} NMR (101 MHz, $CDCl_3$) δ 171.4, 171.3, 133.0, 132.4 (2xC), 129.0 (2xC), 128.1, 86.2 (C-1), 76.1 (C-5), 73.6 (C-3), 71.3 (C-2), 69.0 (C-4), 63.0 (C-6), 21.2, 21.0; HRMS (AJS-ESI): $[M+Na]^+$ for $C_{16}H_{20}O_7SNa^+$ 379.0822 found 379.0817.

4-chlorophenyl 2,6-di-*O*-acetyl-1-thio-β-D-galactopyranoside (4)

According to general procedure with *p*-chlorophenyl 1-thio-β-D-galactopyranoside **2** (0.20 mmol, 60 mg), MeCN (1.2 mL), vinyl acetate (1.2 mL), 45 °C and 60% CAL-B (36 mg). Reaction was run for 24 h resulting in white solid 61 mg (80%). TLC – DCM:EtOAc 1:2, R_f = 0.34; column chromatography eluent system PE:EtOAc 2:3 → 1:9; m.p. 134-137 °C (from DCM); $[\alpha]_D^{20}$ +7.1 (acetone, c 0.08);
^{1}H NMR (400 MHz, $CDCl_3$) δ 7.41-7.46 (m, 2H), 7.24-7.30 (m, 2H), 4.98 (t, *J* = 9.7 Hz, 1H, H-2), 4.56 (d, *J* = 10.0 Hz, 1H, H-1), 4.36 (dd, *J* = 5.6, 11.7 Hz, 1H, H-6a/6b), 4.29 (dd, *J* = 7.0, 11.7 Hz, 1H, H-6a/6b), 3.95 (d, *J* = 3.2 Hz, 1H, H-4), 3.70 (t, *J* = 6.7 Hz, 1H, H-5), 3.68 (dd, *J* = 3.3, 9.4 Hz, 1H, H-3), 3.07 (s, 2H, OH-3,4), 2.15 (s, 3H), 2.08 (s, 3H); ^{13}C{^{1}H} NMR (101 MHz, $CDCl_3$) δ 171.3, 171.3, 134.4, 134.0 (2xC), 131.1, 129.1 (2xC), 85.9 (C-1), 76.2 (C-5), 73.4 (C-3), 71.0 (C-2), 68.9 (C-4), 63.1 (C-6), 21.2, 21.0; HRMS (AJS-ESI): $[M+Na]^+$ for $C_{16}H_{19}ClO_7SNa^+$ 413.0432 found 413.0426.

Phenyl 3,6-di-*O*-acetyl-1-thio-β-D-galactopyranoside (5)

From the same reaction as phenyl 2,6-di-*O*-acetyl-1-thio-β-D-galactopyranose (**3**). Reaction was run for 24 h, resulting in white solid 106 mg (24%); TLC – DCM:EtOAc 1:2, R_f = 0.52; column chromatography eluent system DCM:EtOAc 2:3 → 1:1; m.p. 105-108 °C (from DCM); $[\alpha]_D^{20}$ -6.1 (acetone, c 0.10);
^{1}H NMR (400 MHz, $CDCl_3$) δ 7.53-7.59 (m, 2H), 7.29-7.35 (m, 3H), 4.88 (dd, *J* = 3.2, 9.6 Hz, 1H, H-3), 4.58 (d, *J* = 9.7 Hz, 1H, H-1), 4.32 (dd, *J* = 6.3, 11.7 Hz, 1H, H-6a/6b), 4.29 (dd, *J* = 6.5, 11.6 Hz,

1H, H-6a/6b), 4.05 (dd, *J* = 3.5, 4.7 Hz, 1H, H-5), 3.85 (td, *J* = 9.7, 2.8 Hz, 1H, H-2), 3.78 (t, *J* = 6.4 Hz, 1H, H-5), 2.51 (d, *J* = 2.0 Hz, 1H, OH-2), 2.29 (dd, *J* = 5.2, 7.4 Hz, 1H, OH-4), 2.15 (s, 3H), 2.08 (s, 3H); $^{13}C\{^{1}H\}$ NMR (101 MHz, $CDCl_3$) δ 171.1, 170.7, 132.9 (Cx2), 131.8, 129.2 (Cx2), 128.5, 89.1 (C-1), 76.1 (C-3), 76.1 (C-5), 67.4 (C-4), 67.3 (C-2), 62.7 (C-6), 21.2, 21.0; HRMS (AJS-ESI): $[M+Na]^+$ for $C_{16}H_{20}O_7SNa^+$ 379.0822 found 379.0815.

4-chlorophenyl 3,6-di-*O*-acetyl-1-thio-β-D-galactopyranoside (6)

According to general procedure with *p*-chlorophenyl 1-thio-β-D-galactopyranoside **2** (0.20 mmol, 60 mg), MeCN (5 mL), acetic anhydride (372 µL), 45 °C and 100% CAL-B (60 mg). Reaction was run for 48 h resulting in mixture of products. As such, only NMR characterization was carried out. TLC – DCM: EtOAc 1:2, R_f = 0.54;
^{1}H NMR (400 MHz, $CDCl_3$) δ 7.47-7.53 (m, 2H), 7.26-7.32 (m, 2H), 4.87 (dd, *J* = 3.1, 9.6 Hz, 1H, H-3), 4.54 (d, *J* = 9.7 Hz, 1H, H-1), 4.31 (dd, *J* = 6.1, 11.6 Hz, 1H, H-6a/6b), 4.28 (dd, *J* = 6.5, 11.5 Hz, 1H, H-6a/6b), 4.04 (d, *J* = 2.6 Hz, 1H, H-4), 3.83 (t, *J* = 9.7 Hz, 1H, H-2), 3.77 (t, *J* = 6.7 Hz, 1H, H-5), 2.70 (br.s, 2H, OH-2,4), 2.15 (s, 3H), 2.08 (s, 3H); $^{13}C\{^{1}H\}$ NMR (101 MHz, $CDCl_3$) δ 171.1, 170.7, 134.8, 134.4 (Cx2), 130.1, 129.3 (Cx2), 88.7 (C-1), 76.1 (C), 76.1 (C), 67.3 (C), 67.2 (C), 62.7 (C-6), 21.2, 21.0.

Phenyl 4,6-*O*-ethylidene-1-thio-β-D-galactopyranoside (7)

According to a general procedure with phenyl 1-thio-β-D-galactopyranoside **1** (0.22 mmol, 60 mg), chloroform (5 mL), vinyl acetate (0.4 mL, 20.4 equiv), 45 °C and 100% CAL-B (60 mg). Reaction was run for 72 h resulting in white solid 33.5 mg (51%). TLC – DCM:EtOAc 1:2, R_f = 0.10; column chromatography eluent system DCM:EtOAc 2:3 → 0:1; m.p. 52-55 °C (from MeOH); $[\alpha]_D^{20}$ -54.2 (acetone, c 0.08);
^{1}H NMR (400 MHz, MeOD) δ 7.58-7.63 (m, 2H), 7.23-7.32 (m, 2H), 4.76 (q, *J* = 5.0 Hz, 1H, <u>CH</u>CH_3), 4.58 (d, *J* = 9.3 Hz, 1H, H-1), 4.04 (dd, *J* = 1.5, 12.4 Hz, 1H, H-6a/6b), 3.99 (dd, *J* = 0.8, 3.4 Hz, 1H, H-4), 3.86 (dd, *J* = 1.7, 12.4 Hz, 1H, H-6a/6b), 3.62 (t, *J* = 9.3 Hz, 1H, H-2), 3.56 (dd, *J* = 3.5, 9.3 Hz, 1H, H-3), 3.47 (d, *J* = 1.0 Hz, 1H, H-5), 1.32 (d, *J* = 5.0 Hz, 3H, CH<u>CH_3</u>); $^{13}C\{^{1}H\}$ NMR (101 MHz, MeOD) δ 135.0, 133.1 (Cx2), 129.8 (Cx2), 128.3, 100.1 (<u>CH</u>CH_3), 89.3 (C-1), 77.1 (C-4), 74.9 (C-3), 71.2 (C-5), 70.0 (C-2), 69.9 (C-6), 21.1; HRMS (AJS-ESI): $[M+Na]^+$ for $C_{16}H_{20}O_7SNa^+$ 379.0822 found 379.0817.

2,6-dimethylphenyl 2,6-di-*O*-acetyl-1-thio-β-D-galactopyranoside (9)

According to general procedure with 2,6-dimethylphenyl 1-thio-β-D-galactopyranoside **8** (1.78 mmol, 534 mg), MeCN (11 mL), vinyl acetate (11 mL), 60 °C and 200% CAL-B (1068 mg). Reaction was run for 48 h resulting in white solid 581 mg (85%). TLC – DCM:EtOAc 1:4, R_f = 0.52; column chromatography eluent system DCM:acetone 0% → 15% acetone; m.p. 190-194 °C (from DCM); $[\alpha]_D^{20}$ +41.5 (acetone, c 0.09);
^{1}H NMR (400 MHz, MeOD) δ 7.09-7.16 (m, 3H), 5.14 (t, *J* = 9.8 Hz, 1H, H-2), 4.35 (d, *J* = 10.2 Hz, 1H, H-1), 4.31 (dd, *J* = 8.2, 11.5 Hz, 1H, H-6a/6b), 4.08 (dd, *J* = 4.2, 11.5 Hz, 1H, H-6a/6b), 3.84 (ap.d, *J* = 3.0 Hz, 1H, H-4), 3.65 (dd, *J* = 3.4, 9.5 Hz, 1H, H-3), 3.58 (ddd, *J* = 0.8, 4.2, 8.1 Hz, 1H, H-5), 2.53 (s, 6H, 2xCH_3), 2.16 (s, 3H), 1.94 (s, 3H); $^{13}C\{^{1}H\}$ NMR (101 MHz, MeOD) δ 172.4, 172.1, 145.20 (2xC), 133.0, 130.2, 129.2 (2xC), 90.4 (C-1), 77.6 (C-5), 74.0 (C-3), 72.7 (C-2), 70.5 (C-4), 64.9 (C-6), 22.7 (2xC), 21.1, 20.7; HRMS (AJS-ESI): $[M+Na]^+$ for $C_{18}H_{24}O_7SNa^+$ 407.1135 found 407.1131.

4-chlorophenyl 6-*O*-acetyl-1-thio-β-D-glucopyranoside (11)

According to general procedure with 4-chlorophenyl 1-thio-β-D-glucopyranoside **10** (0.33 mmol, 25 mg), MeCN (0.5 mL), vinyl acetate (0.5 mL), 45 °C and 100% CAL-B (25 mg). Reaction was run

for 30 min resulting in white solid 23 mg (80%). TLC – DCM:EtOAc 1:2, R_f = 0.14; column chromatography eluent system DCM:MeOH 2% → 5% MeOH; m.p. 143-146 °C (from MeOH); $[\alpha]_D^{20}$ -54.9 (acetone, c 0.10);
^{1}H NMR (400 MHz, MeOD) δ 7.48-7.54 (m, 2H), 7.28-7.34 (m, 2H), 4.57 (d, *J* = 9.7 Hz, 1H, H-1), 4.40 (dd, *J* = 2.0, 11.9 Hz, 1H, H-6a/6b), 4.18 (dd, *J* = 6.6, 11.9 Hz, 1H, H-6a/6b), 3.49 (ddd, *J* = 2.1, 6.6, 9.7 Hz, 1H, H-5), 3.38 (t, *J* = 8.8 Hz, 1H, H-3), 3.26 (t, *J* = 9.4 Hz, 1H, H-4), 3.19 (t, *J* = 9.2 Hz, 1H, H-2), 2.05 (s, 3H); ^{13}C{^{1}H} NMR (101 MHz, MeOD) δ 172.6, 134.7 (2xC), 134.7, 133.5, 129.8 (2xC), 88.7 (C-1), 79.4 (C-3), 79.1 (C-5), 73.7 (C-2), 71.4 (C-4), 64.9 (C-6), 20.8; HRMS (AJS-ESI): [M+Na]$^+$ for $C_{14}H_{17}ClO_6SNa^+$ 371.0327 found 371.0318.

4-chlorophenyl 2-acetamido-6-*O*-acetyl-2-deoxy-β-D-glucopyranoside (13)

According to a general procedure with 4-chlorophenyl 2-acetamido-2-deoxy-1-thio-β-D-glucopyranose **12** (0.29 mmol, 100 mg), MeCN (1.9 mL), vinyl acetate (1.9 mL), 45 °C and 100% CAL-B (100 mg). Reaction was run for 4 h resulting in white solid 79.4 mg (71%); TLC – 9:1 DCM:MeOH, R_f = 0.21; column chromatography eluent system DCM:MeOH 2% → 20% MeOH; m.p. 226-229 °C (from MeOH); $[\alpha]_D^{20}$ -37.7 (acetone, c 0.10);
^{1}H NMR (400 MHz, MeOD) δ 7.44-7.49 (m, 2H), 7.28-7.33 (m, 2H), 4.73 (d, *J* = 10.4 Hz, 1H, H-1), 4.42 (dd, *J* = 2.0, 11.9 Hz, 1H, H-6a/6b), 4.19 (dd, *J* = 6.6, 11.9 Hz, 1H, H-6a/6b), 3.74 (t, *J* = 10.1 Hz, 1H, H-2), 3.49 (ddd, *J* = 2.1, 6.7, 9.9 Hz, 1H, H-5), 3.47 (t, *J* = 9.3 Hz, 1H, H-3), 3.29-3.33 (m, 1H, H-4), 2.05 (s, 3H), 2.00 (s, 3H); ^{13}C{^{1}H} NMR (101 MHz, MeOD) δ 173.5, 172.6, 134.6, 134.4 (2xC), 133.9, 129.9 (2xC), 87.6 (C-1), 79.2 (C-5), 77.1 (C-3), 71.9 (C-4), 64.9 (C-6), 56.1 (C-2), 22.9, 20.8; HRMS (AJS-ESI): [M+Na]$^+$ for $C_{16}H_{20}ClNO_6SNa^+$ 412.0592 found 412.0585.

4-chlorophenyl 6-*O*-acetyl-2-deoxy-2-phthalimido-β-D-glucopyranoside (15)

According to a general procedure with 4-chlorophenyl 2-deoxy-2-phthalimido-1-thio-β-D-glucopyranose **14** (0.23 mmol, 100 mg), MeCN (1.5 mL), vinyl acetate (1.5 mL), 45 °C and 100% CAL-B (100 mg). Reaction was run for 2 h resulting in white solid 100.2 mg (91%); product was purified by cristallization with MeOH in 3 batches; TLC – DCM:MeOH 9:1, R_f = 0.5; m.p. 198-200 °C (from MeOH); $[\alpha]_D^{20}$ +56.2 (acetone, c 0.10);
^{1}H NMR (400 MHz, MeOD:CHCl$_3$ 7:3, 1 mL) δ 7.78-7.91 (m, 4H), 7.35 (ap.d, *J* = 8.4 Hz, 2H), 7.22 (ap.d, *J* = 8.4 Hz, 2H), 5.52 (d, *J* = 10.4 Hz, 1H, H-1), 4.47 (dd, *J* = 1.3, 11.9 Hz, 1H, H-6a/6b), 4.27 (dd, *J* = 5.8, 11.4 Hz, 1H, H-6a/6b), 4.23 (t, *J* = 9.2 Hz, 1H, H-3), 4.08 (t, *J* = 10.3 Hz, 1H, H-2), 3.66 (ddd, *J* = 1.7, 6.5, 9.5 Hz, 1H, H-5), 3.41 (t, *J* = 9.3 Hz, 1H, H-4), 2.09 (s, 3H); ^{13}C{^{1}H} NMR (101 MHz, MeOD:CHCl$_3$ 7:3, 1 mL) δ 172.3, 169.3 (Phth-CO), 168.8 (Phth-CO), 135.2 (Phth-ArC), 135.2 (Phth-ArC), 134.9 (S-ArC), 134.7 (2xS-ArC), 132.5 (2xPhth-ArC), 131.6 (S-ArC), 129.6 (2xS-ArC), 124.2 (Phth-ArC), 123.9 (Phth-ArC), 84.1 (C-1), 79.0 (C-5), 73.1 (C-3), 71.8 (C-4), 64.5 (C-6), 56.8 (C-2), 21.0; HRMS (AJS-ESI): [M+Na]$^+$ for $C_{22}H_{20}ClNO_7SNa^+$ 412.0592 found 412.0585.

4-chlorophenyl 1-thio-D-mannopyranoside (16)

Following known procedure[2], 1,2,3,6-tetra-O-acetyl-1-thio-d-mannopyranoside (281 mg, 0.59 mmol) α:β 10:90 was dissolved in dry MeOH (7 mL) equipped with CaCl2 tube. To that solution 0.2 M NaOMe solution in MeOH (0.8 mL, 50 mg Na in 10 mL MeOH) was added. The reaction mixture was stirred at room temperature until full completion was achieved according to TLC, after which dry ice was carefully added. The solvent was removed in vacuo resulting in white solid 173 mg (95%) with α:β 10:90; TLC – DCM:MeOH 9:1, R_f = 0.23;
α - ^{1}H NMR (400 MHz, MeOD) δ 7.48 (d, *J* = 8.6 Hz, 2H), 7.29 (d, *J* = 8.7 Hz, 2H), 4.99 (d, *J* = 0.4 Hz, 1H, H-1), 3.98-4.10 (m, 1H, H-2), 3.89 (dd, *J* = 2.2, 12.0 Hz, 1H, H-6a/6b), 3.71-3.79 (m, 1H, H-6a/6b), 3.63 (t, *J* = 9.5 Hz, 1H, H-4), 3.52 (dd, *J* = 3.3, 9.5 Hz, 1H, H-3), 3.28-3.38 (m, 1H, H-5); ^{13}C{^{1}H} NMR (101 MHz, MeOD) δ 135.1, 133.7, 132.4 (2xC), 130.0 (2xC), 88.6 (C-1), 82.4 (C-5),

76.1 (C-3), 74.1 (C-2), 68.3 (C-4), 62.9 (C-6);
β - ^{1}H NMR (400 MHz, MeOD) δ 7.51 (d, *J* = 8.5 Hz, 1H), 7.31 (d, *J* = 8.5 Hz, 1H), 5.43 (d, *J* = 0.6 Hz, 1H), 4.07 (dd, *J* = 1.4, 2.9 Hz, 1H), 4.01 (ddd, *J* = 2.5, 6.0, 8.8 Hz, 1H), 3.83 (dd, *J* = 2.3, 12.0 Hz, 1H), 3.75 (dd, *J* = 5.6, 12.0 Hz, 1H), 3.72 (dd, *J* = 9.3, 9.3 Hz, 1H), 3.67 (dd, *J* = 3.1, 9.4 Hz, 1H); ^{13}C{^{1}H} NMR (101 MHz, MeOD) δ 134.7, 134.6, 134.3 (2xC), 130.1 (2xC), 90.3 (C-1), 75.8 (C-5), 73.6 (C-2), 73.1 (C-3), 68.7 (C-4), 62.6 (C-6); HRMS (AJS-ESI): [M+Na]$^+$ for $C_{12}H_{15}ClO_5SNa^+$ 329.0221 found 329.0212.

4-chlorophenyl 6-*O*-acetyl-1-thio-β-D-mannopyranoside (17)

According to a general procedure with 4-chlorophenyl 1-thio-D-mannopyranose **16** (0.33 mmol, 100 mg), MeCN (2 mL), vinyl acetate (2 mL), 45 °C and 100% CAL-B (100 mg). Reaction was run for 20 h resulting in white solid 86.6 mg (76%); TLC – DCM:MeOH 9:1, R_f = 0.36; column chromatography eluent system DCM:EtOAc 4:1 → 0:1; m.p. 83-86 °C (from MeOH); $[\alpha]_D^{20}$ +216.5 (acetone, c 0.10);
^{1}H NMR (400 MHz, MeOD) δ 7.47-7.51 (m, 2H), 7.31-7.36 (m, 2H), 5.44 (d, *J* = 1.4 Hz, 1H, H-1), 4.39 (dd, *J* = 1.5, 11.1 Hz, 1H, H-6a/6b), 4.22 (dd, *J* = 7.2, 16.5 Hz, 1H, H-6a/6b), 4.20 (ddd, *J* = 1.5, 7.0, 14.1 Hz, 1H, H-5), 4.07 (t, *J* = 1.9 Hz, 1H, H-2), 3.67-3.70 (m, 2H, H-3,4), 1.99 (s, 3H); ^{13}C{^{1}H} NMR (101 MHz, MeOD) δ 172.7, 134.6, 134.3, 134.2 (2xC), 130.1 (2xC), 89.7 (C-1), 73.2 (C-2), 73.1 (C-3/4/5), 73.0 (C-3/4/5), 68.9 (C-3/4), 65.0 (C-6), 20.7; HRMS (AJS-ESI): [M+Na]$^+$ for $C_{14}H_{17}ClO_6SNa^+$ 371.0327 found 371.0319.

4-chlorophenyl 6-deoxy-1-thio-α-L-mannopyranoside (18)

Following known procedure[2], 4-chlorophenyl 2,3,4-tetra-*O*-acetyl-6-deoxy-1-thio-α-L-mannopyranoside (580 mg, 1.39 mmol) was dissolved in dry MeOH (15 mL) equipped with $CaCl_2$ tube. To that solution, 0.2 M NaOMe solution in MeOH (1.75 mL, 50 mg Na in 10 mL MeOH) was added. The reaction mixture was stirred at room temperature upon reaching completion according to TLC, after which dry ice was carefully added. The reaction mixture was concentrated in vacuo resulting in white solid 404 mg (99%). TLC – DCM:MeOH 9:1, R_f = 0.35; m.p. 62-65 °C (from MeOH), $[\alpha]_D^{20}$ -281.6 (MeOH, c 0.12);
^{1}H NMR (400 MHz, MeOD) δ 7.42-7.47 (m, 2H), 7.29-7.34 (m, 2H), 5.39 (d, *J* = 1.1 Hz, 1H, H-1), 4.07 (dd, *J* = 1.5, 3.3 Hz, 1H, H-2), 4.02 (ddd, *J* = 6.2, 9.4, 12.4 Hz, 1H, H-5), 3.64 (dd, *J* = 3.3, 9.5 Hz, 1H, H-3), 3.47 (t, *J* = 9.4 Hz, 1H, H-4), 1.27 (d, *J* = 6.2 Hz, 3H); ^{13}C{^{1}H} NMR (101 MHz, MeOD) δ 134.8, 134.4, 133.9 (2xC), 130.1 (2xC), 90.0 (C-1), 74.0 (C-4), 73.7 (C-2), 72.9 (C-3), 71.0 (C-5), 17.8; HRMS (AJS-ESI): [M+Na]$^+$ for $C_{12}H_{15}ClO_4SNa^+$ 313.0272 found 313.0265.

4-chlorophenyl 2-*O*-acetyl-6-deoxy-1-thio-α-L-mannopyranoside (19), 4-chlorophenyl 3-*O*-acetyl-6-deoxy-1-thio-α-L-mannopyranoside (20) and 4-chlorophenyl 4-*O*-acetyl-6-deoxy-1-thio-α-L-mannopyranoside (21)

According to a general procedure with 4-chlorophenyl 6-deoxy-1-thio-L-mannopyranoside **18** (0.34 mmol, 100 mg), MeCN (2.2 mL), vinyl acetate (2.2 mL), 45 °C and 100% CAL-B (100 mg). Reaction was run for 20 h resulting in two fractions. Fraction 1 is a mixture of 4-chlorophenyl 2-*O*-acetyl-6-deoxy-1-thio-α-L-mannopyranoside (**19**) (57%) and 4-chlorophenyl 3-*O*-acetyl-6-deoxy-1-thio-α-L-mannopyranoside (**20**) (43%), gel-like 42 mg (36%), and fraction 2 is 4-chlorophenyl 4-*O*-acetyl-6-deoxy-1-thio-α-L-mannopyranoside (**21**), white solid 31 mg (27%), with total yield of 72 mg (63%); TLC – DCM:MeOH 9:1, Fraction 1 R_f = 0.51, Fraction 2 R_f = 0.49 ; column chromatography eluent system PE:EtOAc 4:1 → 1:4; 4-chlorophenyl 4-*O*-acetyl-6-deoxy-1-thio-α-L-mannopyranoside (**21**) - m.p. 138-141 °C (from MeOH); $[\alpha]_D^{20}$ -221.4 (acetone, c 0.09);
4-chlorophenyl 2-*O*-acetyl-6-deoxy-1-thio-α-L-mannopyranoside (**19**) - ^{1}H NMR (400 MHz, MeOD) δ 7.42-7.48 (m, 2H), 7.30-7.35 (m, 2H), 5.40 (d, *J* = 1.1 Hz, 1H, H-1), 5.24 (dd, *J* = 1.4, 3.5 Hz, 1H, H-2), 4.04 (ddd, *J* = 6.2, 9.4, 12.4 Hz, 1H, H-5), 3.81 (dd, *J* = 3.5, 9.6 Hz, 1H, H-3), 3.44

(t, *J* = 9.5 Hz, 1H, H-4), 2.10 (s, 3H), 1.28 (d, *J* = 4.9 Hz, 3H); $^{13}C\{^1H\}$ NMR (101 MHz, MeOD) δ 172.2, 134.8, 134.3 (2xC), 134.1, 130.3 (2xC), 87.2 (C-1), 75.5 (C-2), 74.2 (C-4), 71.2 (C-3/5), 71.2 (C-3/5), 20.9, 17.9;
4-chlorophenyl 3-*O*-acetyl-6-deoxy-1-thio-α-L-mannopyranoside (**20**) - ^1H NMR (400 MHz, MeOD) δ 7.42-7.48 (m, 2H), 7.30-7.35 (m, 2H), 5.39 (d, *J* = 1.3 Hz, 1H, H-1), 4.91 (dd, *J* = 3.2, 9.9 Hz, 1H, H-3), 4.23 (dd, *J* = 1.6, 3.1 Hz, 1H, H-2), 4.12 (ddd, *J* = 6.2, 9.5, 12.4 Hz, 1H, H-5), 3.67 (t, *J* = 9.7 Hz, 1H, H-4), 2.12 (s, 3H), 1.30 (d, *J* = 4.8 Hz, 3H); $^{13}C\{^1H\}$ NMR (101 MHz, MeOD) δ 172.5, 134.6, 134.1 (2xC), 134.1, 130.2 (2xC), 89.6 (C-1), 75.7 (C-3), 71.3 (C-2/4/5), 71.2 (C-2/4/5), 71.1 (C-2/4/5), 21.0, 17.9;
HRMS (AJS-ESI): Fraction 1 $[M+Na]^+$ for $C_{14}H_{17}ClO_5SNa^+$ 355.0377 found 355.0373.
4-chlorophenyl 4-*O*-acetyl-6-deoxy-1-thio-α-L-mannopyranoside (**21**) - ^1H NMR (400 MHz, MeOD) δ 7.43-7.47 (m, 2H), 7.31-7.36 (m, 2H), 5.42 (d, *J* = 1.2 Hz, 1H, H-1), 5.01 (t, *J* = 9.7 Hz, 1H, H-4), 4.16 (ddd, *J* = 6.2, 9.7, 12.4 Hz, 1H, H-5), 4.10 (dd, *J* = 1.5, 3.3 Hz, 1H, H-2), 3.79 (dd, *J* = 3.3, 9.7 Hz, 1H, H-3), 2.11 (s, 3H), 1.15 (d, *J* = 6.3 Hz, 3H); $^{13}C\{^1H\}$ NMR (101 MHz, MeOD) δ 172.4, 134.6, 134.4, 134.0 (2xC), 130.2 (2xC), 89.8 (C-1), 75.5 (C-4), 73.6 (C-2), 70.9 (C-3), 68.9 (C-5), 21.0, 17.7; HRMS (AJS-ESI): for $C_{14}H_{17}ClO_5SNa^+$ 355.0377 found 355.0370.

4-chlorophenyl (β-D-galactopyranosyl)-(1→4)-1-thio-β-D-glucopyranoside (22)

Following known procedure[2], 4-chlorophenyl (2,3,4,6-tetra-*O*-acetyl-β-D-galactopyranosyl-(1→4))-2,3,6-tri-*O*-acetyl-1-thio-D-glucosylpyranoside (350 mg, 0.46 mmol) α:β 20:80 was dissolved in dry MeOH (5 mL) equipped with $CaCl_2$ tube. To that solution, 0.2 M NaOMe solution in MeOH (1 mL, 50 mg Na in 10 mL MeOH) was added. The reaction mixture was stirred at room temperature upon reaching completion according to TLC, after which dry ice was carefully added. The white solid precipitation was filtered resulting in 4-chlorophenyl (β-D-galactopyranosyl)-(1→4)-1-thio-β-D-glucopyranoside (**36**) 70 mg (32%). The filtrate was concentrated in vacuo resulting in white solid 165 mg (77%) with α:β 23:77; TLC – DCM:MeOH 4:1, R_f = 0.21; β-m.p. 199-201 °C (from H_2O), β-$[\alpha]_D^{20}$ -42.7 (H_2O, c 0.08);
β - ^1H NMR (400 MHz, MeOD) δ 7.53 (d, *J* = 8.5 Hz, 2H), 7.34 (d, *J* = 8.5 Hz, 2H), 4.67 (d, *J* = 9.9 Hz, 1H, H-1), 4.40 (d, *J* = 7.1 Hz, 1H, H'-1), 3.92 (dd, *J* = 1.6, 12.2 Hz, 1H), 3.86 (d, *J* = 2.5 Hz, 1H), 3.75-3.85 (m, 2H), 3.71 (dd, *J* = 4.1, 11.7 Hz, 1H), 3.49-3.68 (m, 6H), 3.29-3.34 (m, 1H); $^{13}C\{^1H\}$ NMR (101 MHz, MeOD) δ 134.6, 134.4 (2xC), 132.6, 130.0 (2xC), 104.3 (C), 88.5 (C), 80.1, 79.4, 77.3, 76.7, 74.1, 72.9, 72.2, 69.9, 62.2 (C-6/C'-6), 61.4 (C-6/C'-6); HRMS (AJS-ESI): for $C_{18}H_{25}ClO_{10}SNa^+$ 491.0749 found 491.0743.

3,6-di-*O*-acetyl-D-glucopyranose (**24**)

According to a general procedure with D-glucose **23** (0.56 mmol, 100 mg), MeCN (3.5 mL), vinyl acetate (3.5 mL), 45 °C and 100% CAL-B (100 mg). Reaction was run for 72 h resulting in white solid 133 mg (91%) α:β 61:39. TLC – DCM:EtOAc 1:4, R_f = 0.12; column chromatography eluent system DCM:MeOH 2% → 10% MeOH.
α - ^1H NMR (400 MHz, MeOD) δ 5.20 (t, *J* = 9.6 Hz, 1H, H-3), 5.11 (d, *J* = 3.6 Hz, 1H, H-1), 4.33 (dd, *J* = 2.2, 11.9 Hz, 1H, H-6a/6b), 4.21 (dd, *J* = 5.5, 11.6 Hz, 1H, H-6a/6b), 4.05 (ddd, *J* = 2.1, 5.3, 10.0 Hz, 1H, H-5), 3.50 (dd, *J* = 3.7, 9.9 Hz, 1H, H-2), 3.43 (t, *J* = 9.7 Hz, 1H, H-4), 2.11 (s, 3H), 2.05 (s, 3H).
β - ^1H NMR (400 MHz, MeOD) δ 4.92 (t, *J* = 9.4 Hz, 1H, H-3), 4.56 (d, *J* = 7.8 Hz, 1H, H-1), 4.38 (dd, *J* = 2.0, 11.8 Hz, 1H, H-6a/6b), 4.19 (dd, *J* = 5.8, 11.5 Hz, 1H, H-6a/6b), 3.57 (ddd, *J* = 2.1, 5.7, 9.9 Hz, 1H, H-5), 3.45 (t, *J* = 9.5 Hz, 1H, H-4), 3.25 (dd, *J* = 7.9, 9.6 Hz, 1H, H-2), 2.11 (s, 3H), 2.05 (s, 3H).
NMR matches with previously reported values.[10]

2-acetomido-6-*O*-acetyl-2-deoxy-D-glucopyranose (**27**)

According to a general procedure with 2-acetomido-2-deoxy-D-glucopyranose **26** (0.45 mmol, 100 mg), MeCN (3 mL), vinyl acetate (3 mL), 60 °C and 200% CAL-B (200 mg). Reaction was run for 72 h resulting in white solid 48 mg (40%) α:β 93:7. TLC – DCM:MeOH 9:1, R_f = 0.12; column chromatography eluent system DCM:MeOH 9:1 → 3:1.
α - ^{1}H NMR (400 MHz, D_2O) δ 8.10 (d, *J* = 8.8 Hz, 1H, NH), 5.17 (d, *J* = 3.5 Hz, 1H, H-1), 4.34 (dd, *J* = 2.8, 12.3 Hz, 1H, H-6a/6b), 4.30 (dd, *J* = 4.6, 12.3 Hz, 1H, H-6a/6b), 4.03 (ddd, *J* = 2.7, 4.4, 10.1 Hz, 1H, H-5), 3.87 (dd, *J* = 3.5, 10.6 Hz, 1H, H-2), 3.74 (dd, *J* = 9.0, 10.6 Hz, 1H, H-3), 3.51 (dd, *J* = 9.1, 10.0 Hz, 1H, H-4), 2.11 (s, 3H), 2.02 (s, 3H).
Only α-NMR shown, β-NMR was too weak. α-NMR matches with previously reported values.[11]

6-*O*-acetyl-2-deoxy-2-(2,2,2-trichloroethoxycarbonylamino)-D-glucopyranose (29)

According to a general procedure with phenyl 2-deoxy-2-(2,2,2-trichloroethoxycarbonylamino)-D-glucopyranose **28** α:β:Fur 79:11:10 (0.28 mmol, 100 mg), MeCN (1.8 mL), vinyl acetate (1.8 mL), 45 °C and 100% CAL-B (100 mg). Reaction was run for 2 h resulting in white solid 91.6 mg (82%) α:β:Fur-α 79:11:10; TLC – DCM:MeOH 9:1, R_f = 0.38; column chromatography eluent system DCM:MeOH 1:0 → 9:1;
α - ^{1}H NMR (400 MHz, MeOD) δ 5.12 (d, *J* = 3.4 Hz, 1H, H-1), 4.84 (d, *J* = 12.1 Hz, 1H, Troc$\underline{CH_2}$), 4.72 (d, *J* = 12.1 Hz, 1H, Troc$\underline{CH_2}$), 4.35 (dd, *J* = 2.1, 11.8 Hz, 1H, H-6a/6b), 4.21 (dd, *J* = 5.5, 11.8 Hz, 1H, H-6a/6b), 3.99 (ddd, *J* = 2.1, 5.5, 10.0 Hz, 1H, H-5), 3.69 (dd, *J* = 8.8, 10.5 Hz, 1H, H-3), 3.58 (dd, *J* = 3.4, 10.6 Hz, 1H, H-2), 3.35 (dd, *J* = 8.9, 9.9 Hz, 1H, H-4), 2.06 (s, 3H); $^{13}C\{^1H\}$ NMR (101 MHz, MeOD) δ 172.9, 156.9 (Troc$\underline{CO}$), 97.1 (Troc$\underline{CCl_3}$) 92.8 (C-1), 75.6 (Troc$\underline{CH_2}$), 72.5 (C-3), 72.5 (C-4), 70.7 (C-5), 65.0 (C-6), 57.8 (C-2), 20.7.
β - ^{1}H NMR (400 MHz, MeOD) δ 5.00 (d, *J* = 12.0 Hz, 1H, Troc$\underline{CH_2}$), 4.64 (d, *J* = 12.0 Hz, 1H, Troc$\underline{CH_2}$), 4.60 (d, *J* = 8.3 Hz, 1H, H-1), 4.40 (dd, *J* = 1.9, 11.9 Hz, 1H, H-6a/6b), 4.18 (dd, *J* = 5.8, 11.6 Hz, 1H, H-6a/6b), 3.47 (ddd, *J* = 2.0, 5.9, 9.7 Hz, 1H, H-5), 3.46 (t, *J* = 9.7 Hz, 1H, H-4), 3.29-3.36 (m, 2H, H-2,3), 2.06 (s, 3H); $^{13}C\{^1H\}$ NMR (101 MHz, MeOD) δ 172.8, 156.9 (Troc$\underline{CO}$), 97.1 (Troc$\underline{CCl_3}$), 97.0 (C-1), 75.7 (Troc$\underline{CH_2}$), 75.2 (C-4,5,3), 65.0 (C-6), 60.5 (C-2), 20.7.
Fur-α ^{1}H NMR (400 MHz, MeOD) δ 5.20 (d, *J* = 3.07, H-1). Rest of the structure was not characterized.
HRMS (AJS-ESI): $[M+Na]^+$ for $C_{11}H_{16}Cl_3NO_8Na^+$ 417.9834 found 417.9824.

1,6-di-*O*-acetyl-D-mannopyranoside (**31**), 2,6-di-*O*-acetyl-D-mannopyranose (**32**) and **3,6-di-*O*-acetyl-D-mannopyranose (33)**

According to a general procedure with D-mannopyranose **30** (0.56 mmol, 100 mg), MeCN (3.5 mL), vinyl acetate (3.5 mL), 45 °C and 100% CAL-B (100 mg). Reaction was run for 20 h resulting in white solid 119.3 mg (81%), a mixture of 1,6-di-*O*-acetyl-D-mannopyranoside **31** (13%), 2,6-di-*O*-acetyl-D-mannopyranose **32** α:β 12:88 (73%) and 3,6-di-*O*-acetyl-D-mannopyranose **33** (24%); TLC – DCM:MeOH 9:1, R_f = 0.34; column chromatography eluent system DCM:EtOH 1:0 → 3:17;
1,6-di-*O*-acetyl-D-mannopyranoside (**31**) α - ^{1}H NMR (400 MHz, MeOD) δ 5.96 (d, *J* = 1.8 Hz, 1H, H-1), 4.32-4.41 (m, 1H, H-6a/6b), 4.17-4.26 (m, 1H, H-6a/6b), 3.82 (dd, *J* = 1.9, 2.8 Hz, 1H, H-2), 3.77 (ddd, *J* = 1.8, 5.4, 10.4 Hz, 1H, H-5), 3.69-3.71 (m, 2H, H-3,4), 2.10 (s, 3H), 2.06 (s, 3H); $^{13}C\{^1H\}$ NMR (101 MHz, MeOD) δ 172.8, 170.6, 94.3 (C-1), 74.3 (C-5), 72.1 (C-3/4), 70.9 (C-2), 68.0 (C-3/4), 65.1 (C-6), 20.8, 20.7.
2,6-di-*O*-acetyl-D-mannopyranose (**32**) α - ^{1}H NMR (400 MHz, MeOD) δ 5.03 (d, *J* = 1.7 Hz, 1H, H-1), 4.98 (dd, *J* = 1.8, 3.5 Hz, 1H, H-2), 4.38 (dd, *J* = 2.1, 11.8 Hz, 1H, H-6a/6b), 4.21 (dd, *J* = 6.0, 11.8 Hz, 1H, H-6a/6b), 3.92-3.98 (m, *J* = 3.0 Hz, 2H, H-3,5), 3.61 (t, *J* = 9.8 Hz, 1H, H-4), 2.10 (s, 3H), 2.06 (s, 3H); $^{13}C\{^1H\}$ NMR (101 MHz, MeOD) δ 172.8, 172.4, 93.1 (C-1), 74.8 (C-2), 71.6 (C-5), 70.2 (C-3/5), 69.0 (C-4), 65.1 (C-6), 20.9, 20.7.

2,6-di-*O*-acetyl-D-mannopyranose (**32**) β - ^{1}H NMR (400 MHz, MeOD) δ 5.28 (dd, *J* = 1.1, 3.4 Hz, 1H, H-2), 4.89 (d, *J* = 1.0 Hz, 1H, H-1), 4.43 (dd, *J* = 1.8, 11.8 Hz, 1H, H-6a/6b), 4.17-4.26 (m, 1H, H-6a/6b), 3.66 (dd, *J* = 3.4, 9.0 Hz, 1H, H-3), 3.45-3.55 (m, 2H, H-4,5), 2.13 (s, 3H), 2.06 (s, 3H); ^{13}C{^{1}H} NMR (101 MHz, MeOD) δ 172.8, 172.4, 94.3 (C-1), 75.7 (C-4/5), 74.2 (C-2), 73.5 (C-3), 68.6 (C-4/5), 65.1 (C-6), 20.9, 20.7.
3,6-di-*O*-acetyl-D-mannopyranose (**33**) α - ^{1}H NMR (400 MHz, MeOD) δ 5.05 (dd, *J* = 3.3, 10.0 Hz, 1H, H-3), 5.04 (d, *J* = 1.6 Hz, 1H, H-1), 4.34 (dd, *J* = 1.9, 13.1 Hz, 1H, H-6a/6b), 4.17-4.26 (m, 1H, H-6a/6b), 4.03 (ddd, *J* = 2.0, 5.9, 10.0 Hz, 1H, H-5), 3.92-3.98 (m, 1H, H-2), 3.85 (t, *J* = 10.1 Hz, 1H, H-4), 2.11 (s, 3H), 2.06 (s, 3H); ^{13}C{^{1}H} NMR (101 MHz, MeOD) δ 172.9, 172.5, 95.8 (C-1), 75.6 (C-3), 71.6 (C-5), 70.5 (C-2), 66.1 (C-4), 65.0 (C-6), 21.0, 20.7.
HRMS (AJS-ESI): $[M+Na]^+$ for $C_{10}H_{16}O_8Na^+$ 287.0737 found 287.0731.

4-*O*-acetyl-6-deoxy-L-mannopyranose (**35**)

According to a general procedure with 6-deoxy-L-mannopyranose hydrate **34** (0.55 mmol, 100 mg), MeCN (3.5 mL), vinyl acetate (3.5 mL), 45 °C and 100% CAL-B (100 mg). Reaction was run for 72 h resulting in white solid 106 mg (96%) α:β 72:28. TLC – DCM:MeOH 9:1, R_f = 0.26; column chromatography eluent system DCM:MeOH 2% → 16% MeOH;
α - ^{1}H NMR (400 MHz, MeOD) δ 5.02 (d, *J* = 1.4 Hz, 1H, H-1), 4.91 (t, *J* = 9.7 Hz, 1H, H-4), 3.93 (ddd, *J* = 6.2, 9.8, 12.5 Hz, 1H, H-5), 3.86 (dd, *J* = 3.3, 9.7 Hz, 1H, H-3), 3.81 (dd, *J* = 1.7, 3.3 Hz, 1H, H-2), 2.08 (s, 3H), 1.12 (d, *J* = 6.3 Hz, 3H), ^{13}C NMR (101 MHz, MeOD) δ 95.71 (s, 1C), 75.85 (s, 1C), 73.04 (s, 1C), 70.08 (s, 1C), 67.05 (s, 1C), 21.0, 18.0.
β - ^{1}H NMR (400 MHz, MeOD) δ 4.87 (t, *J* = 8.2 Hz, 1H, H-4), 4.74 (d, *J* = 0.8 Hz, 1H, H-1), 3.82-3.86 (m, 1H, H-2), 3.62 (dd, *J* = 3.3, 9.8 Hz, 1H, H-3), 3.43 (ddd, *J* = 6.2, 9.6, 12.4 Hz, 1H, H-5), 2.08 (s, 3H), 1.17 (d, *J* = 6.2 Hz, 3H).

NMR Spectra

Figure S1: Phenyl 1-thio-β-D-glucopyranoside (**1**) ^{1}H NMR (400 MHz) in MeOD

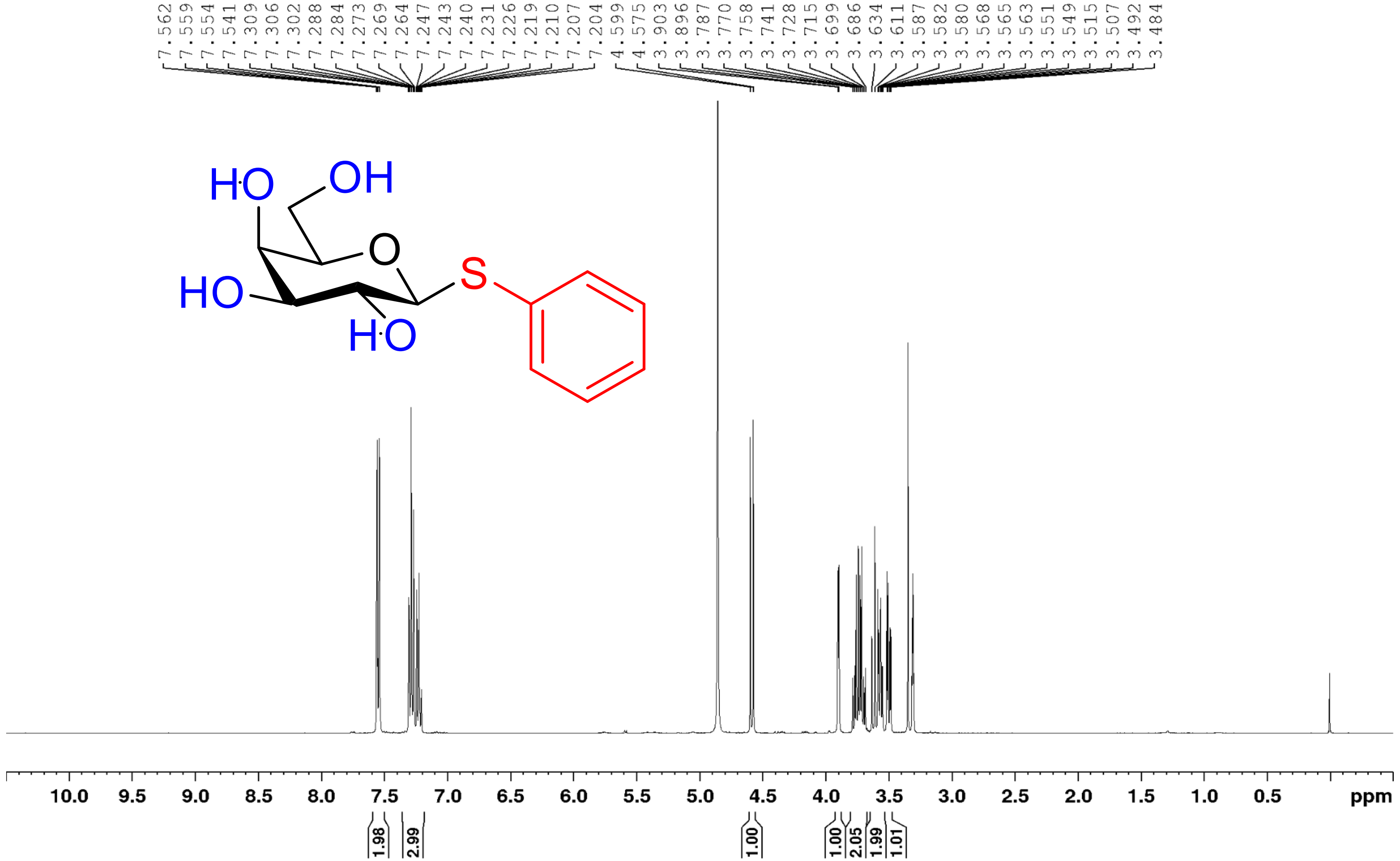

Figure S2: 4-chlorophenyl 1-thio-β-D-glucopyranoside (**2**) ^{1}H NMR (400 MHz) in $CDCl_3$

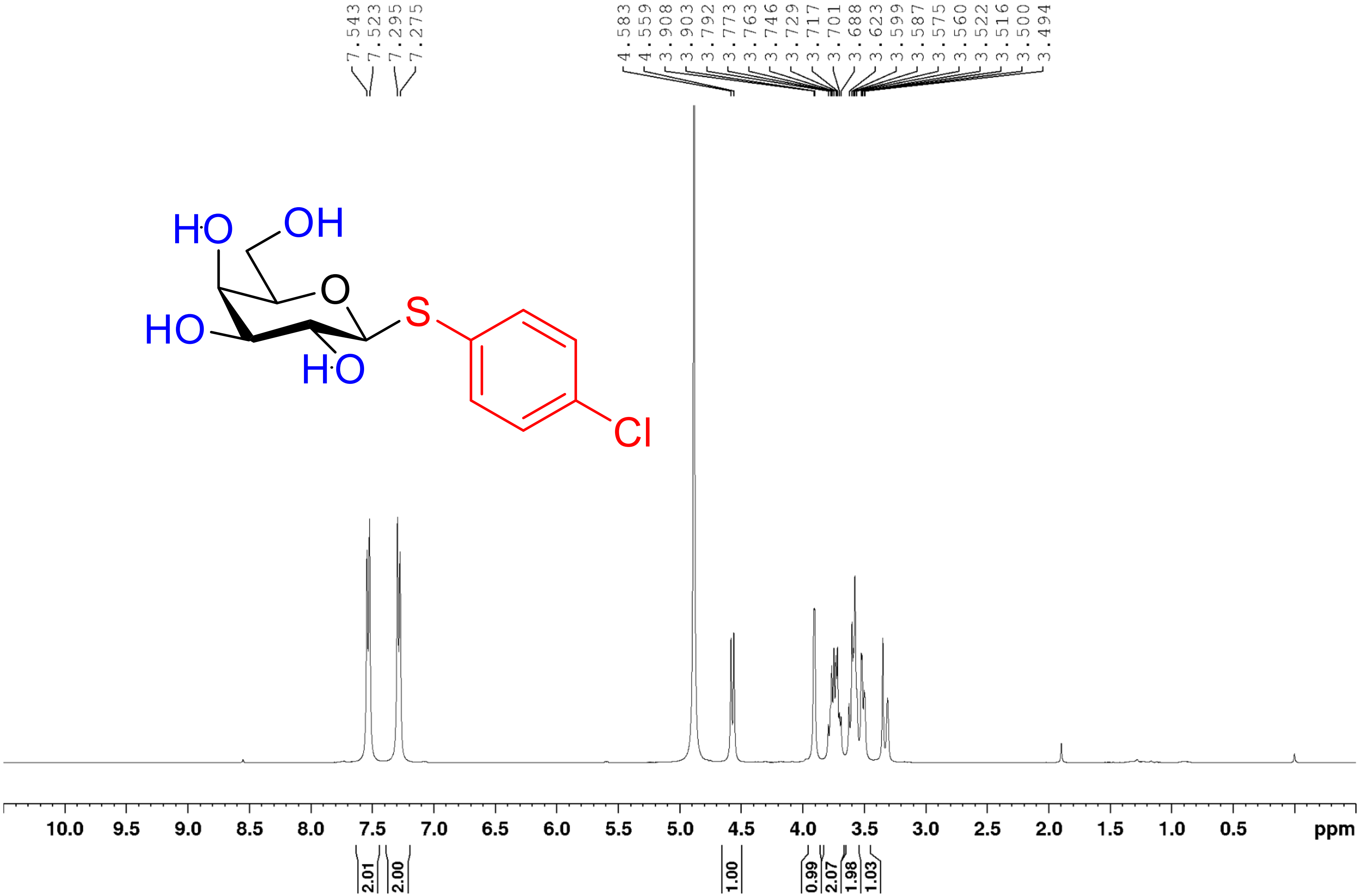

Figure S3: Phenyl 2,6-di-*O*-acetyl-1-thio-β-D-galactopyranoside (3) ^{1}H NMR (400 MHz) in $CDCl_3$

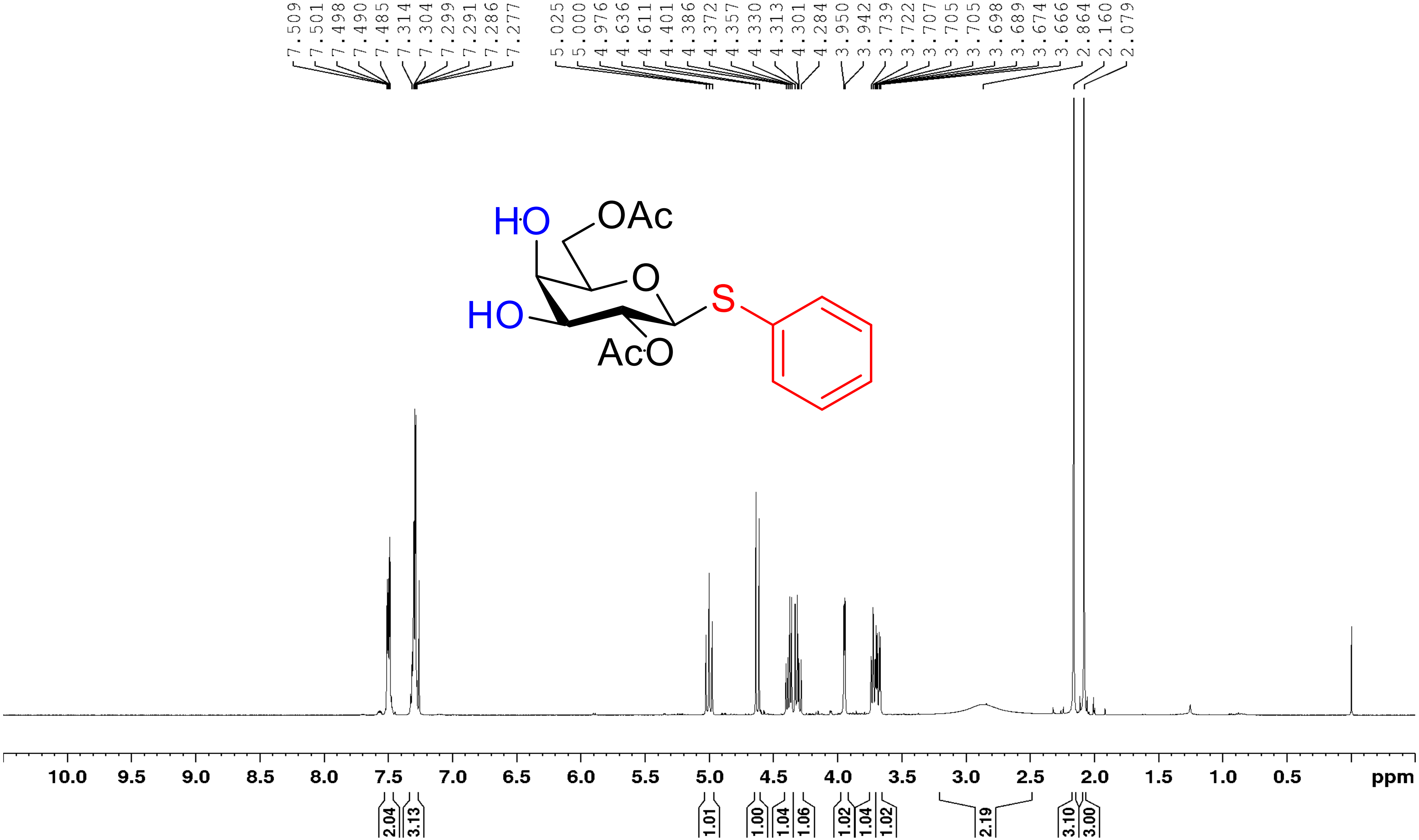

Figure S4: Phenyl 2,6-di-*O*-acetyl-1-thio-β-D-galactopyranoside (3) ^{1}H-^{1}H COSY NMR (400 MHz) in $CDCl_3$

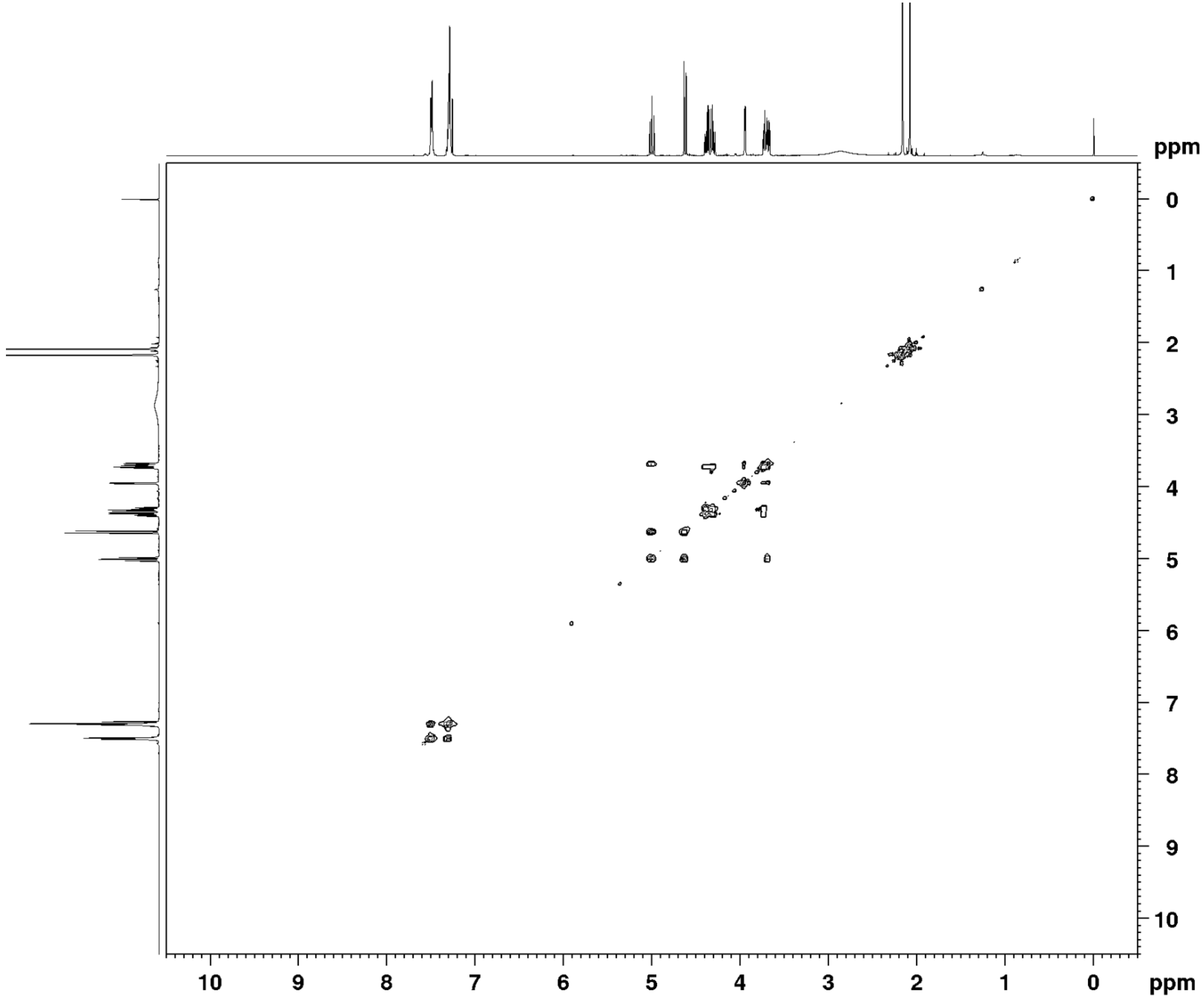

Figure S5: Phenyl 2,6-di-*O*-acetyl-1-thio-β-D-galactopyranoside (3) ^{1}H-^{13}C{^{1}H} HSQC NMR (400 & 101 MHz) in $CDCl_3$

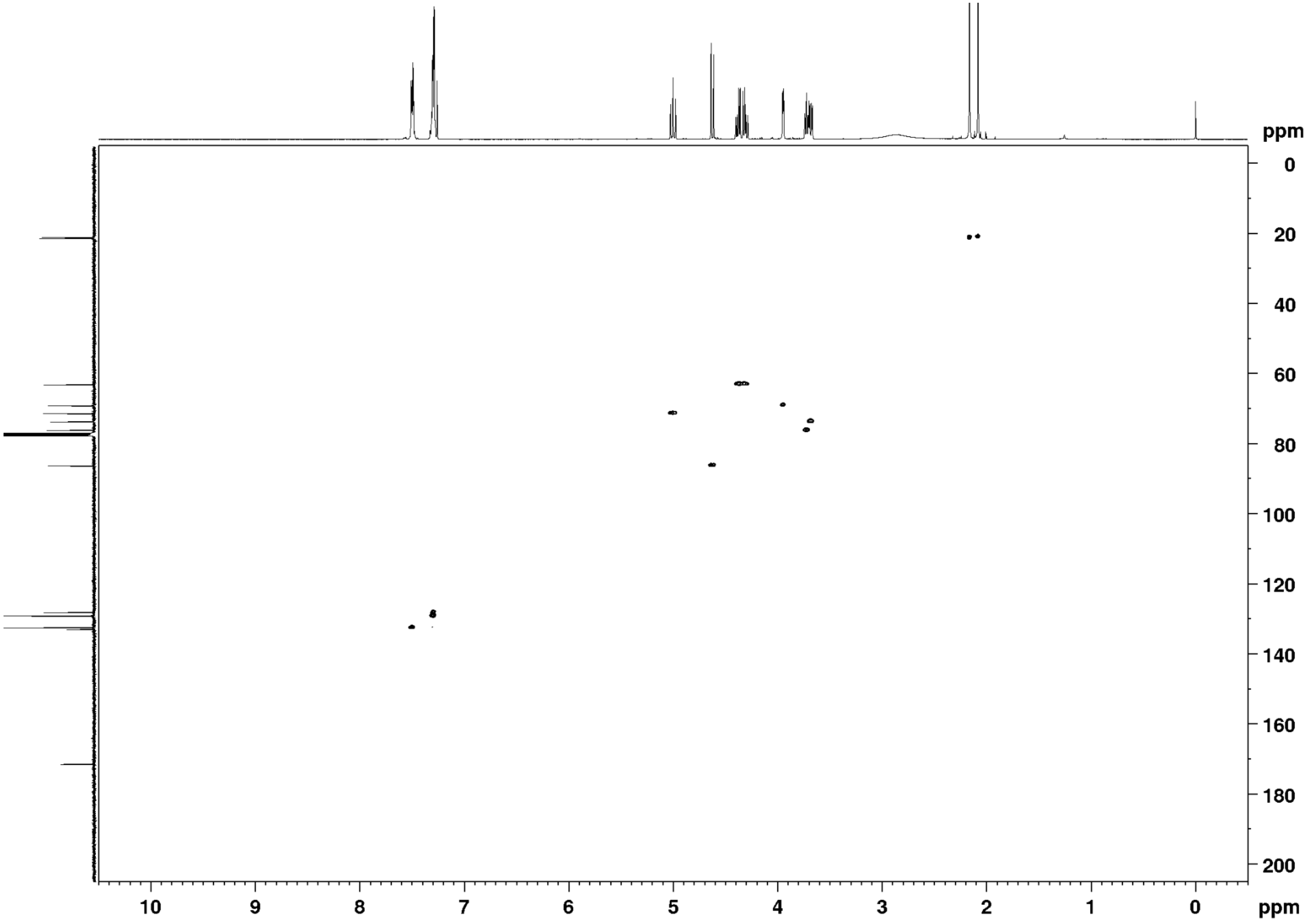

Figure S6: Phenyl 2,6-di-*O*-acetyl-1-thio-β-D-galactopyranoside (3) ^{1}H-$^{13}C\{^{1}H\}$ HMBC NMR (400 & 101 MHz) in $CDCl_3$

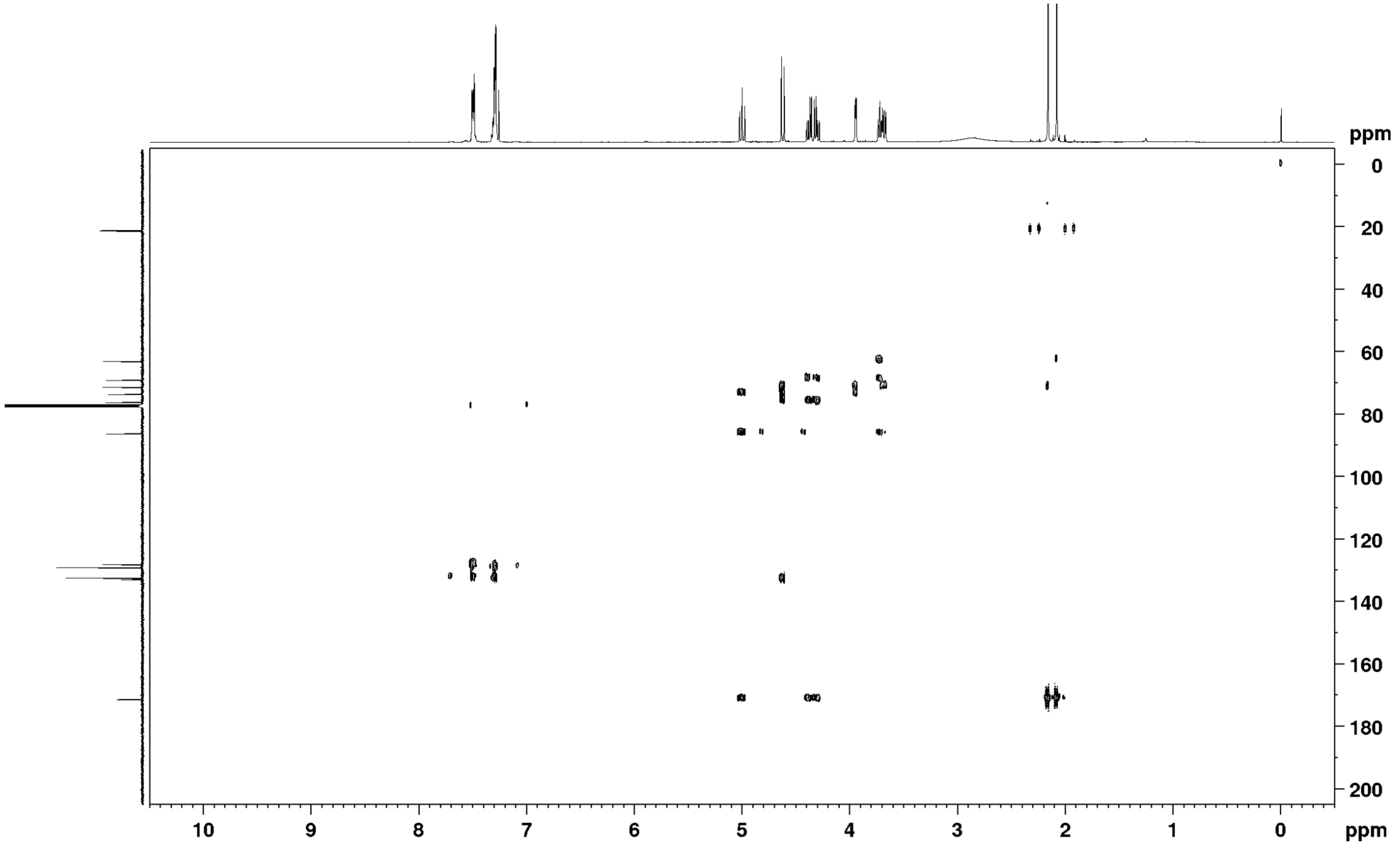

Figure S7: Phenyl 2,6-di-*O*-acetyl-1-thio-β-D-galactopyranoside (3) $^{13}C\{^{1}H\}$ NMR (101 MHz) in $CDCl_3$

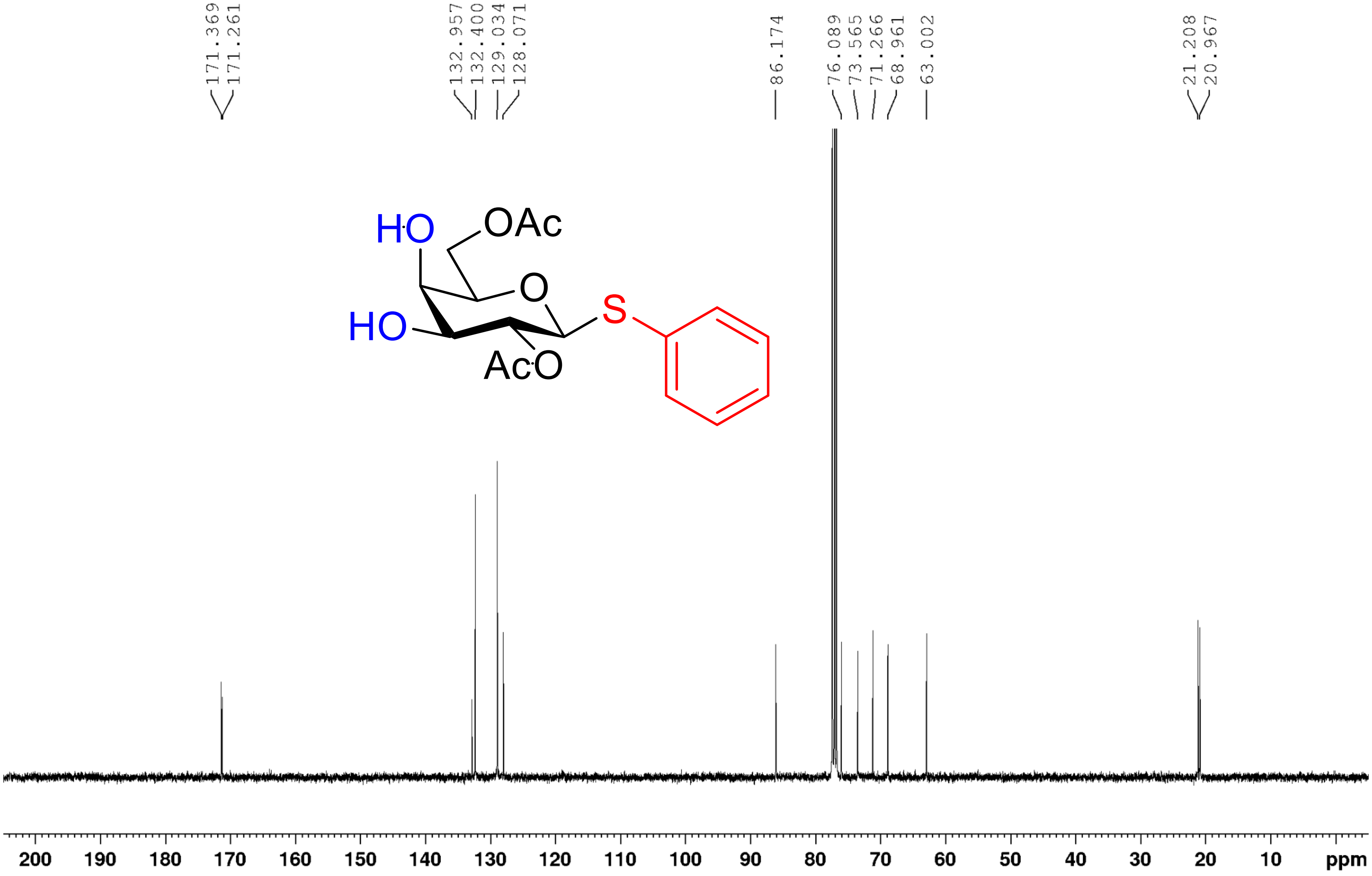

Figure S8: 4-chlorophenyl 2,6-di-*O*-acetyl-1-thio-β-D-galactopyranoside (4) ^{1}H NMR (400 MHz) in $CDCl_3$

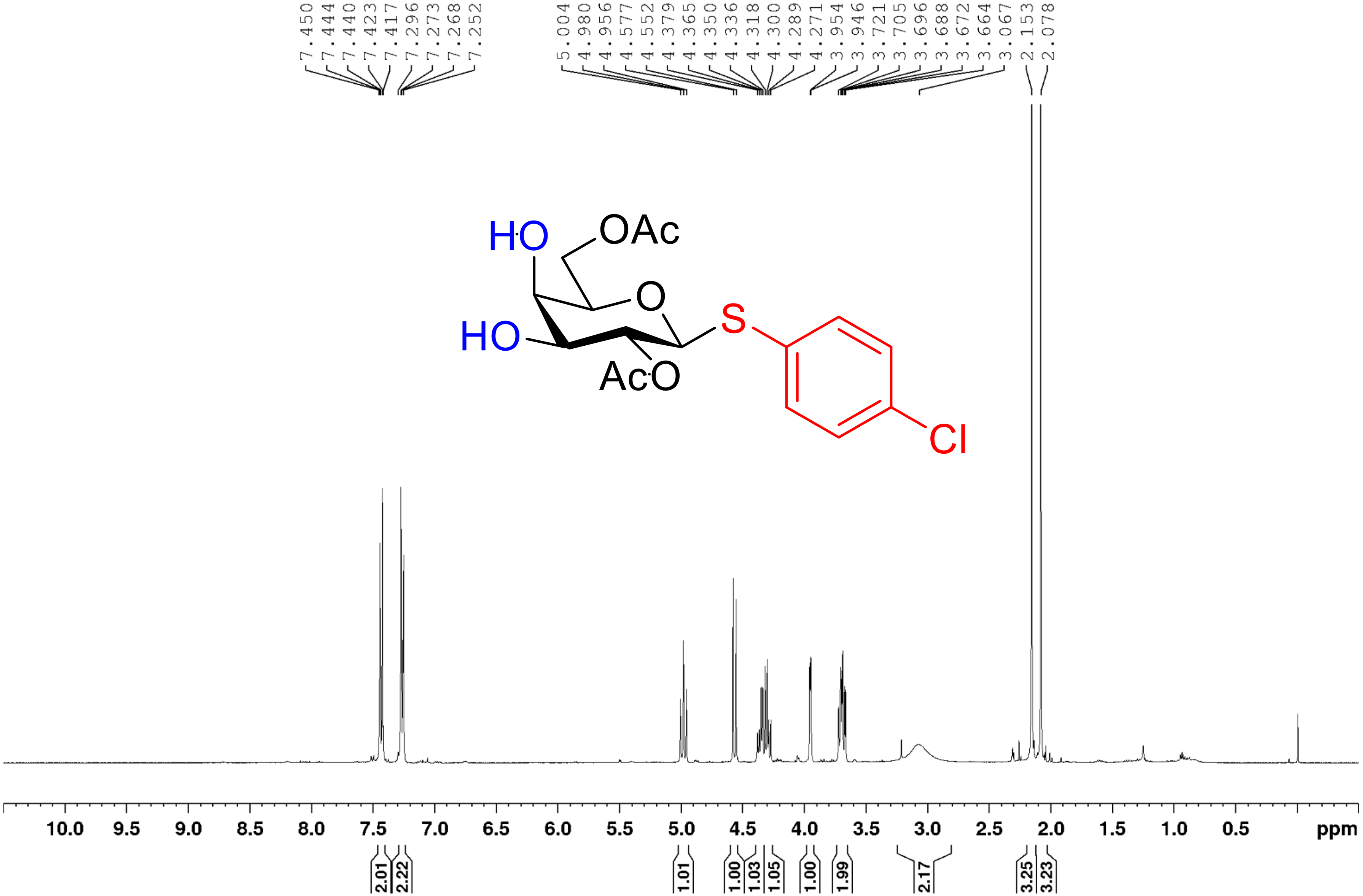

Figure S9: 4-chlorophenyl 2,6-di-*O*-acetyl-1-thio-β-D-galactopyranoside (4) ^{1}H-^{1}H COSY NMR (400 MHz) in $CDCl_3$

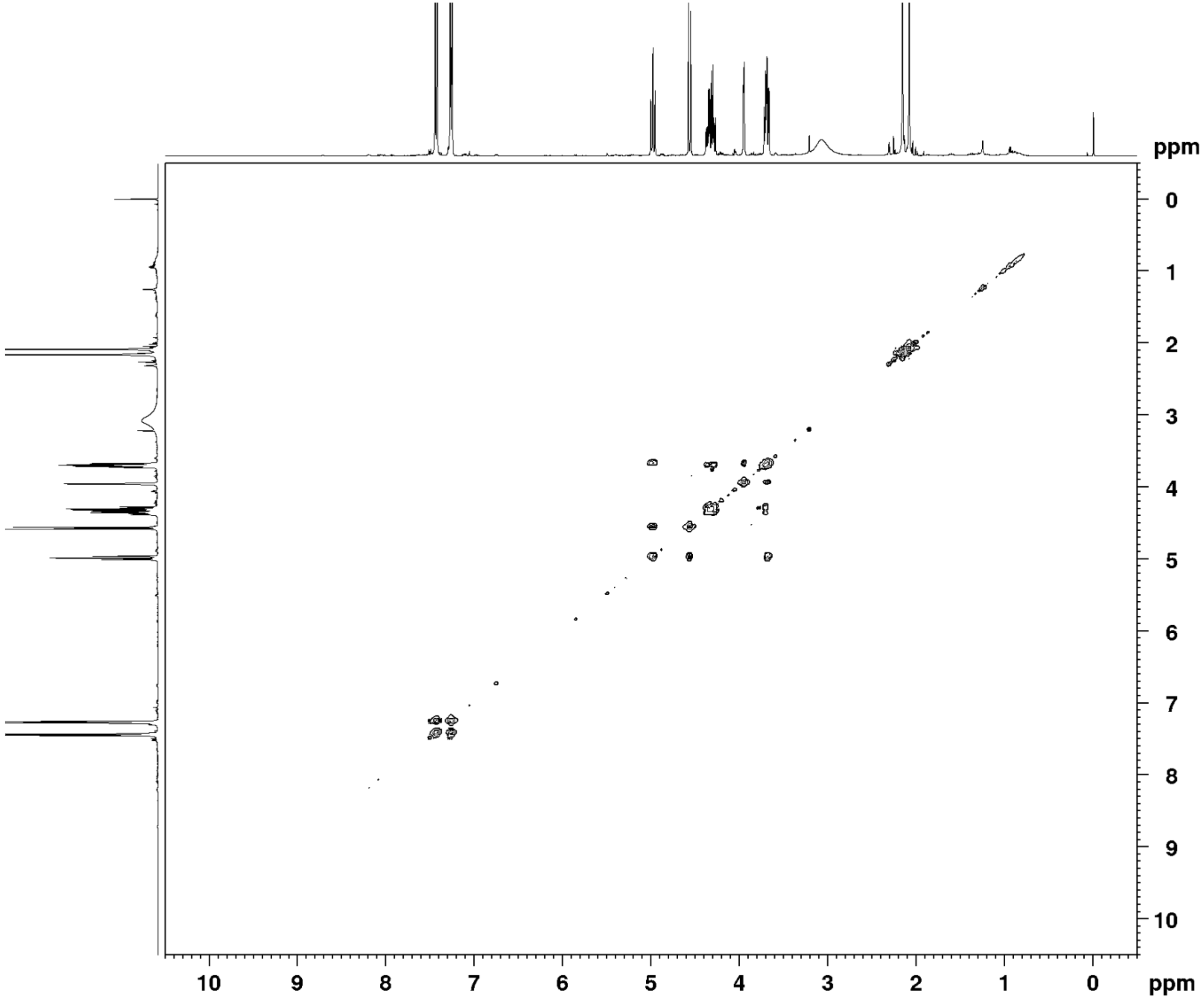

Figure S10: 4-chlorophenyl 2,6-di-*O*-acetyl-1-thio-β-D-galactopyranoside (4) ^{1}H-$^{13}C\{^{1}H\}$ HSQC NMR (400 & 101 MHz) in $CDCl_3$

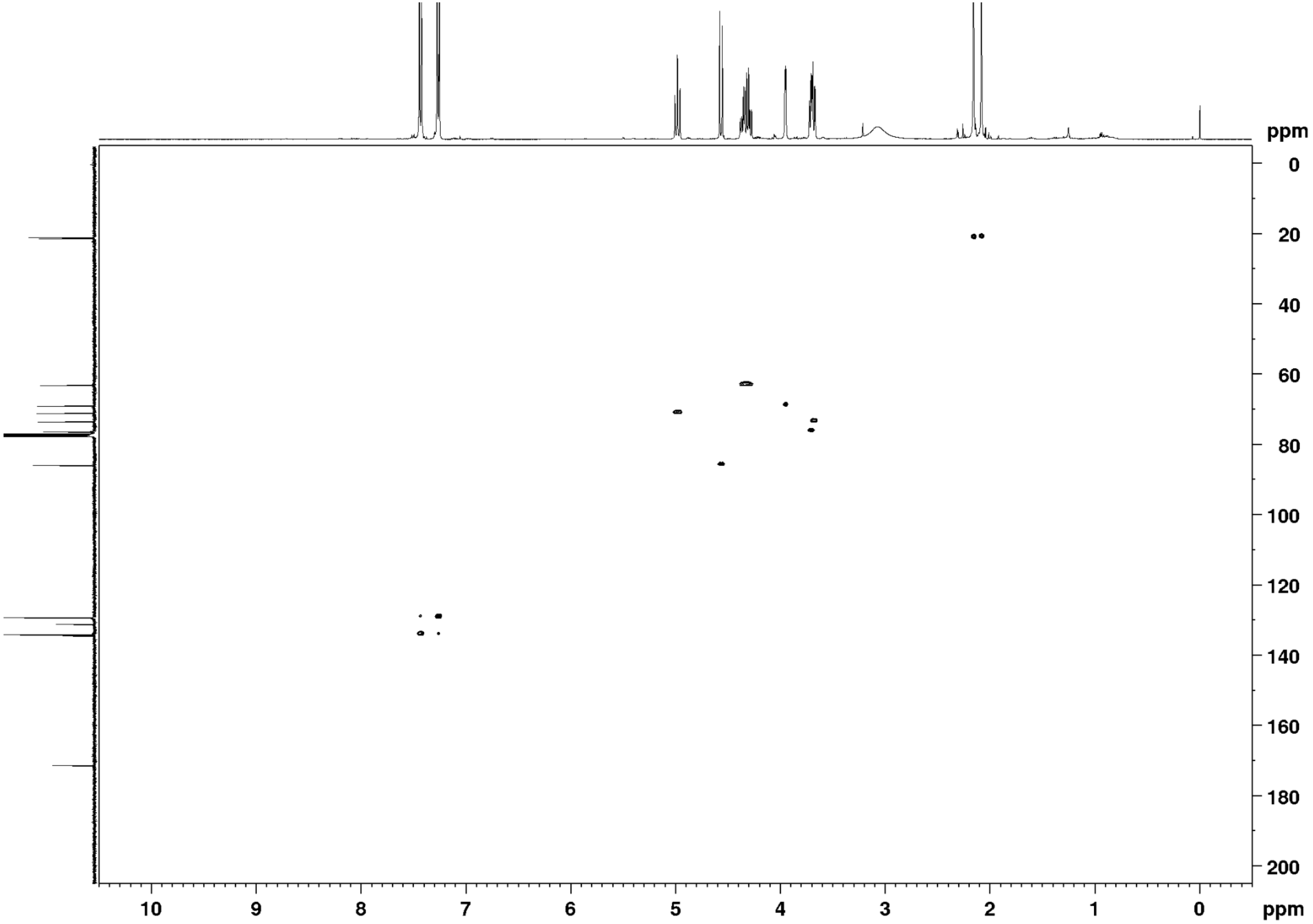

Figure S11: 4-chlorophenyl 2,6-di-*O*-acetyl-1-thio-β-D-galactopyranoside (4) ^{1}H-$^{13}C\{^{1}H\}$ HMBC NMR (400 & 101 MHz) in $CDCl_3$

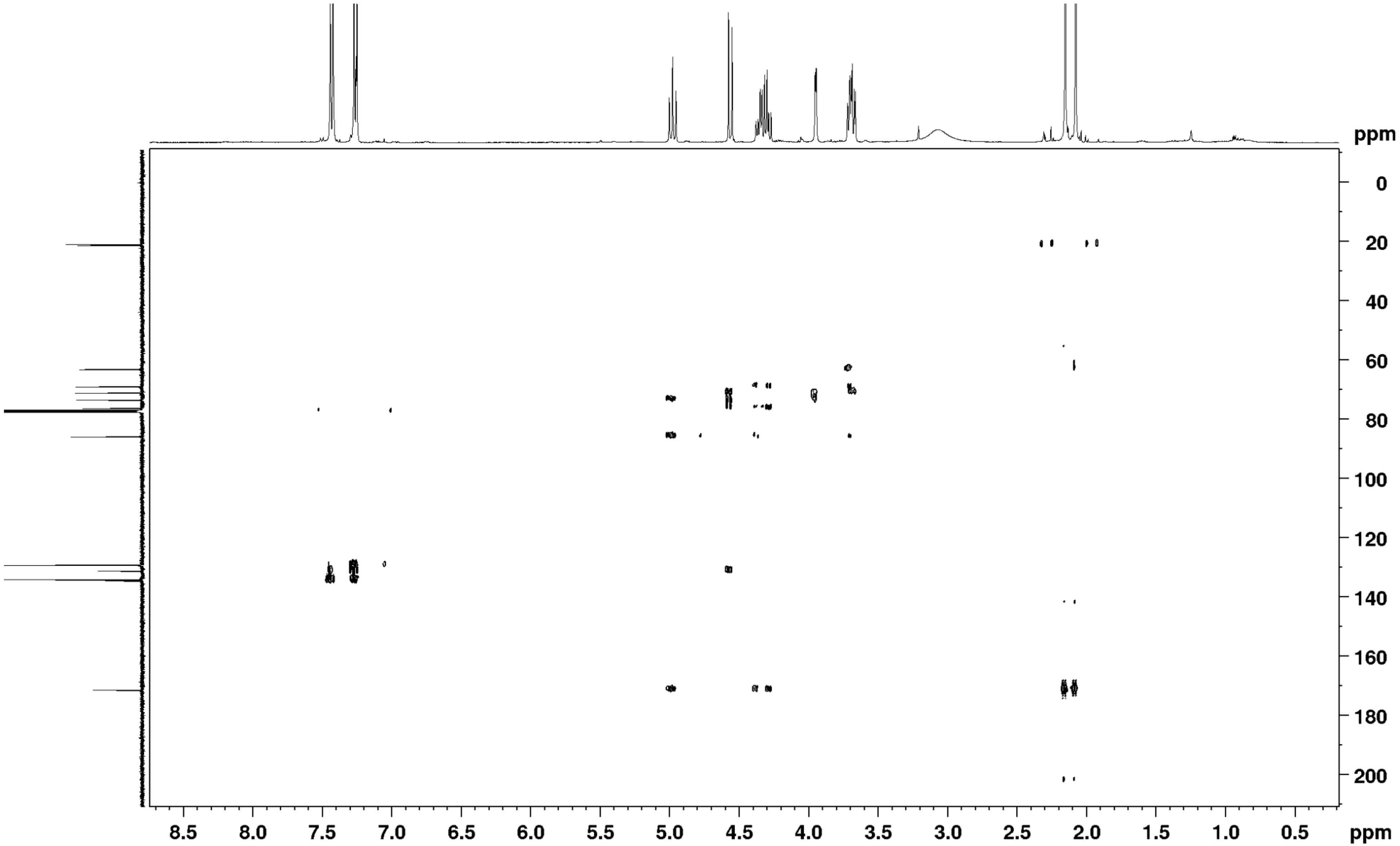

Figure S12: 4-chlorophenyl 2,6-di-*O*-acetyl-1-thio-β-D-galactopyranoside (4) $^{13}C\{^{1}H\}$ NMR (101 MHz) in $CDCl_3$

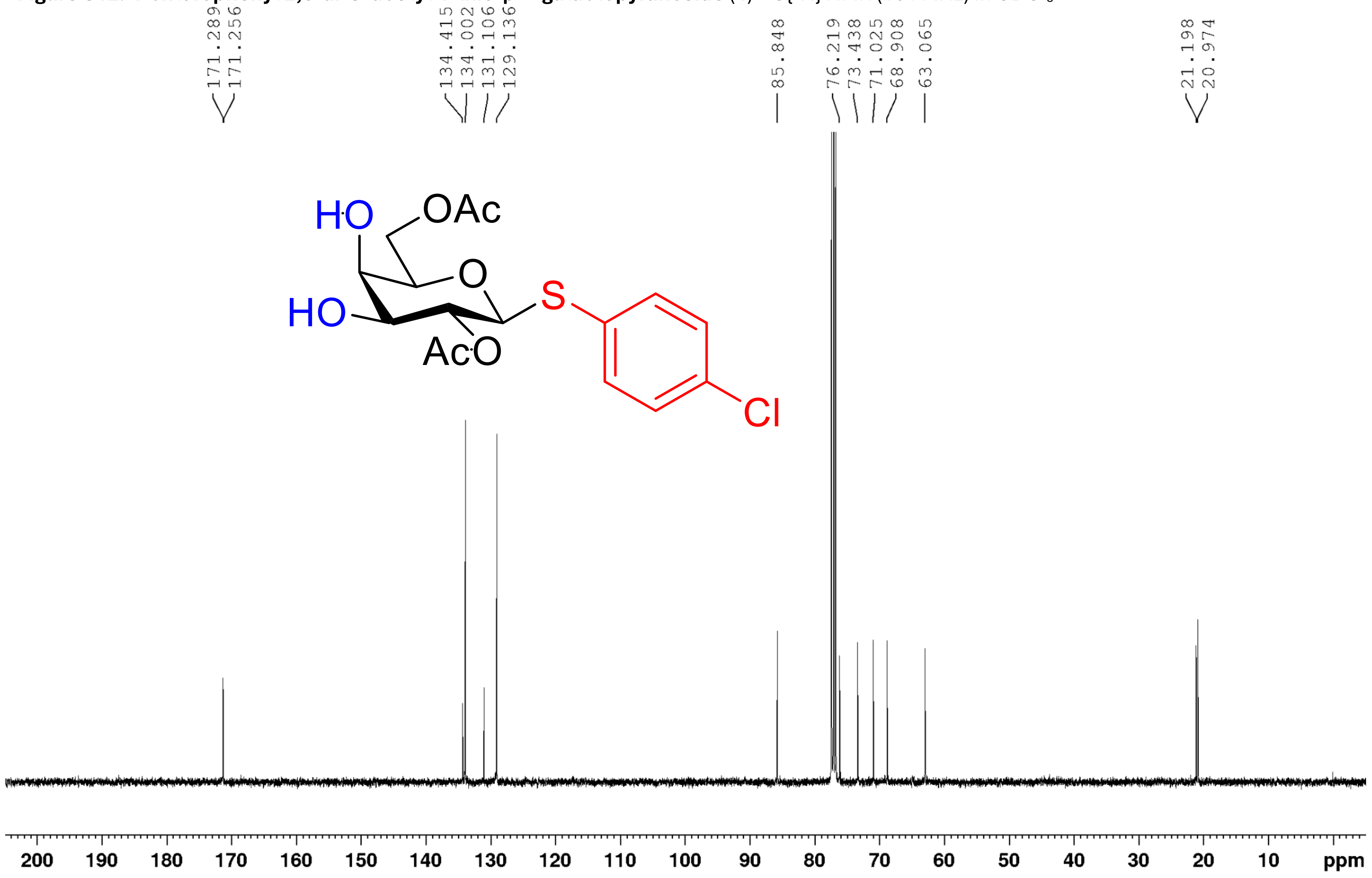

Figure S13: Phenyl 3,6-di-*O*-acetyl-1-thio-β-D-galactopyranoside (5) ^{1}H NMR (400 MHz) in $CDCl_3$

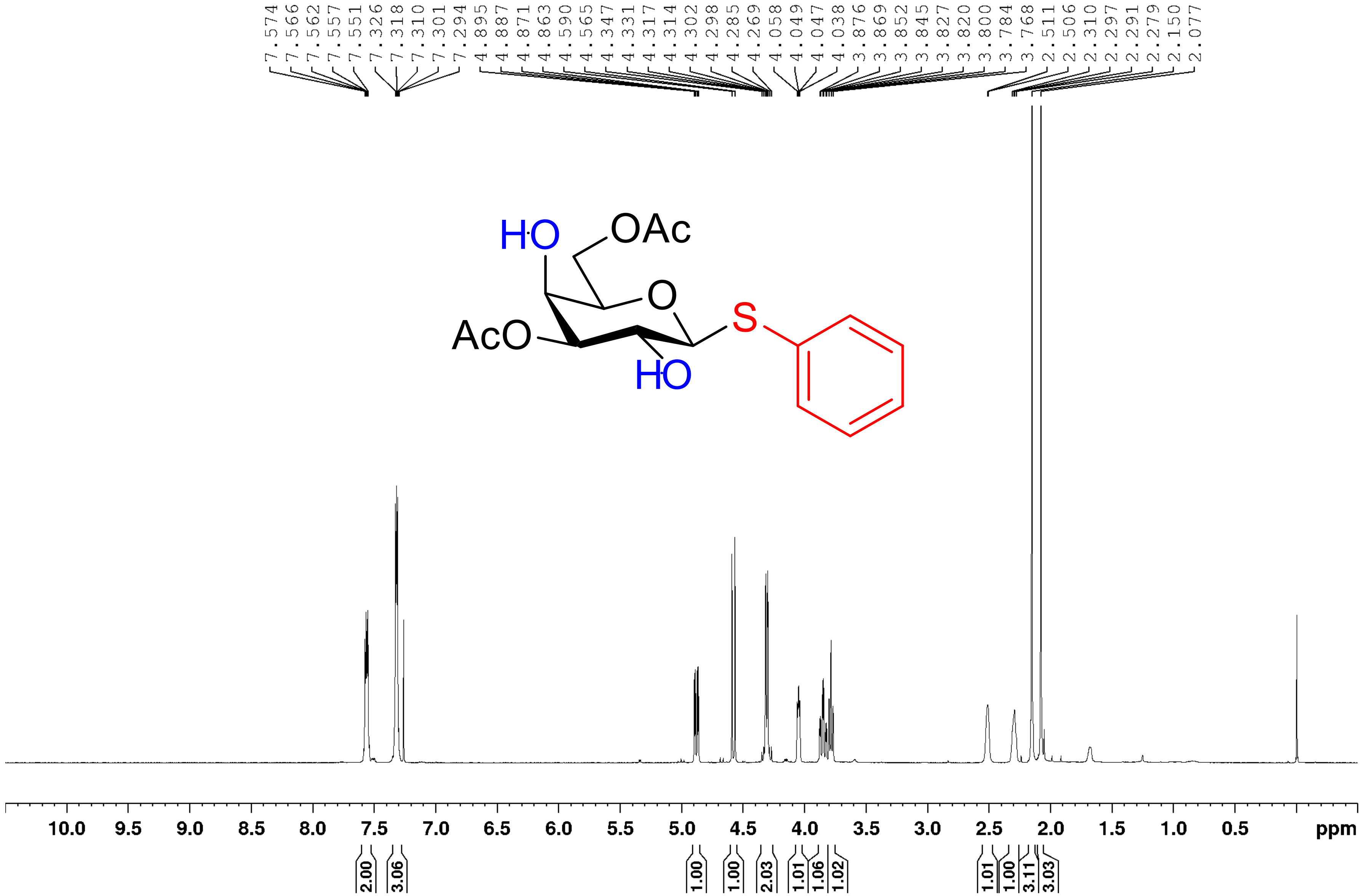

Figure S14: Phenyl 3,6-di-*O*-acetyl-1-thio-β-D-galactopyranoside (5) ^{1}H-^{1}H COSY NMR (400 MHz) in $CDCl_3$

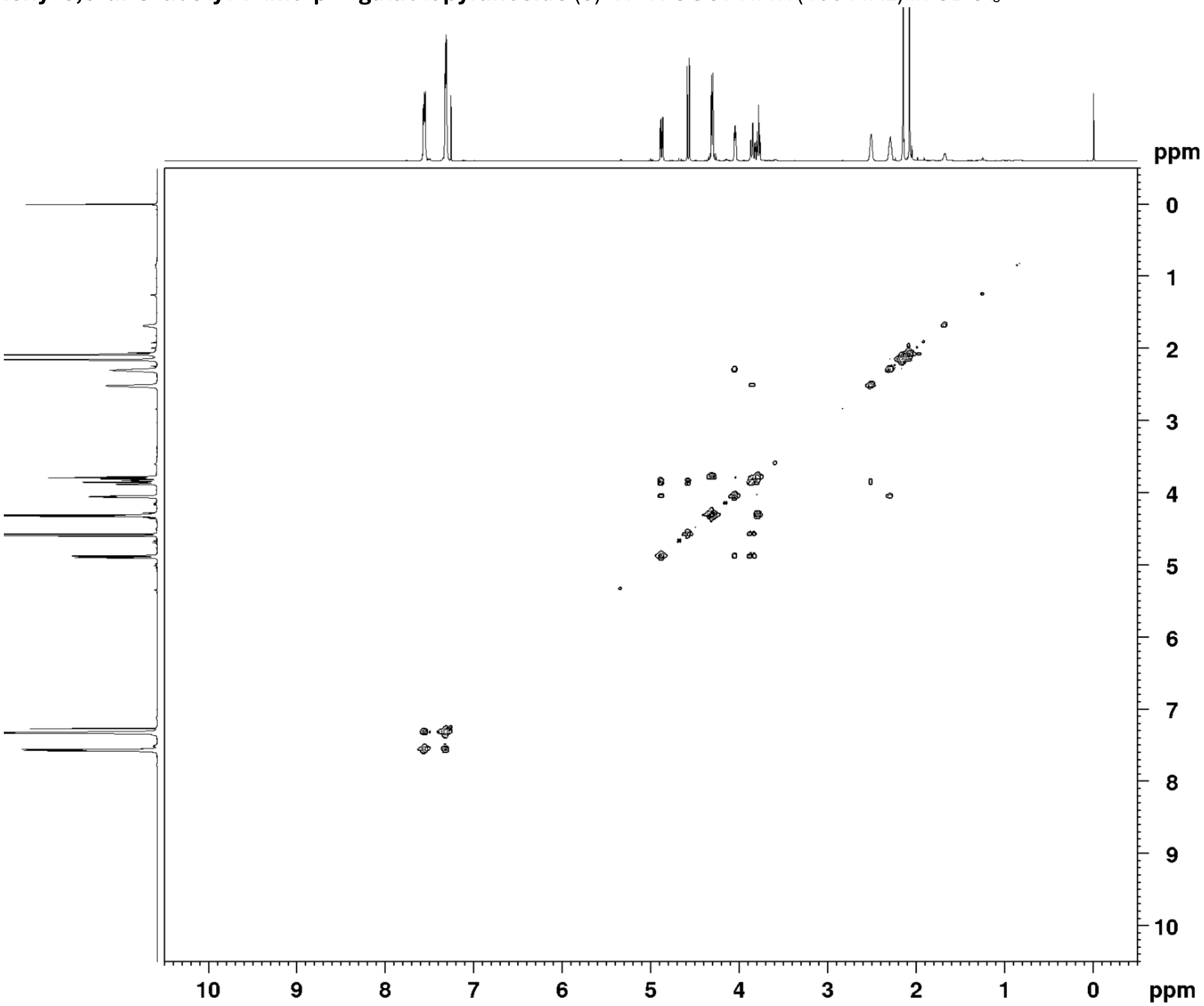

Figure S15: Phenyl 3,6-di-*O*-acetyl-1-thio-β-D-galactopyranoside (5) ^{1}H-$^{13}C\{^{1}H\}$ HSQC NMR (400 & 101 MHz) in $CDCl_3$

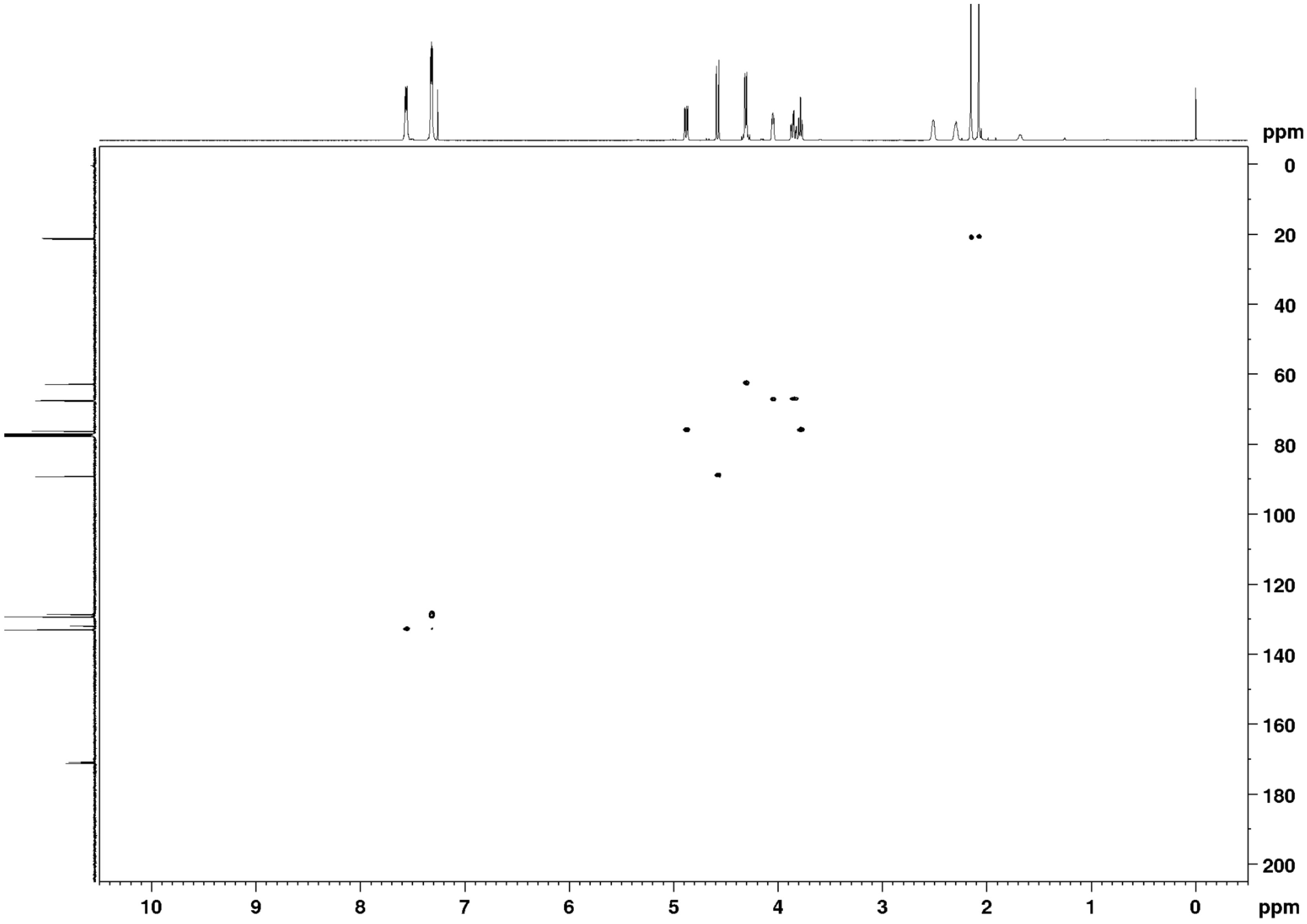

Figure S16: Phenyl 3,6-di-*O*-acetyl-1-thio-β-D-galactopyranoside (5) ^{1}H-$^{13}C\{^{1}H\}$ HMBC NMR (400 & 101 MHz) in $CDCl_3$

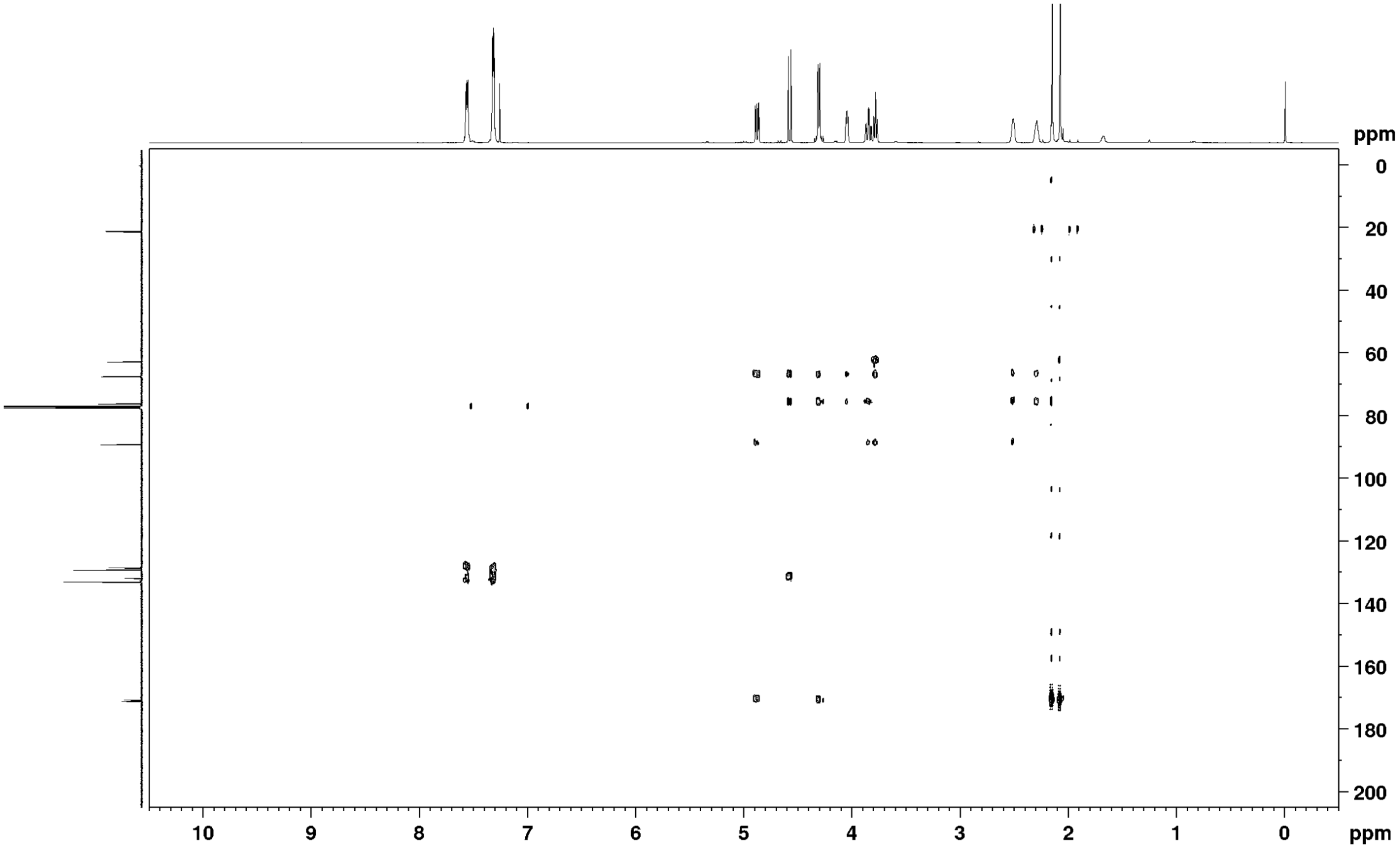

Figure S17: Phenyl 3,6-di-*O*-acetyl-1-thio-β-D-galactopyranoside (5) $^{13}C\{^{1}H\}$ NMR (101 MHz) in $CDCl_3$

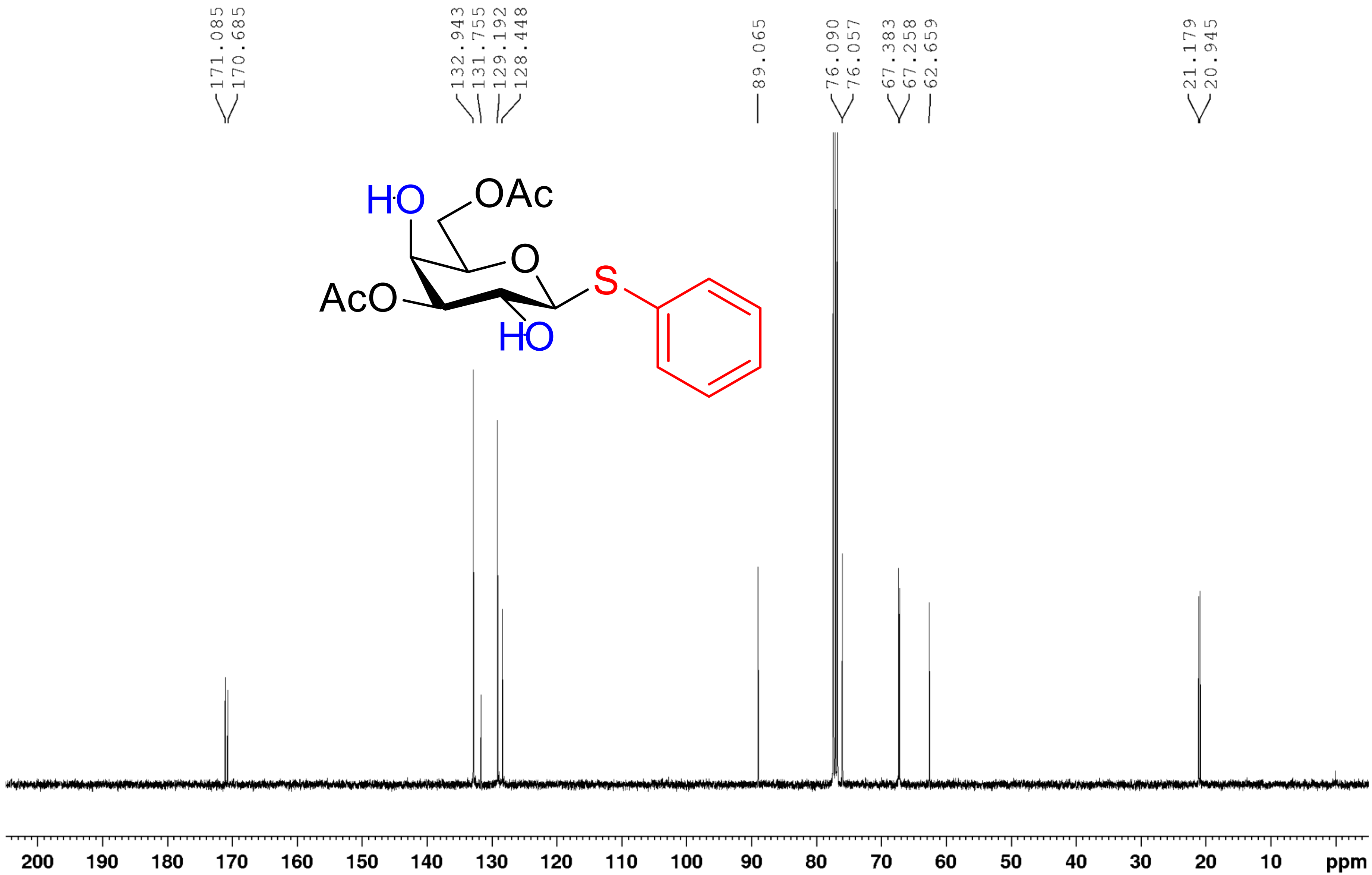

Figure S18: Mixture, where dominant is **4-chlorophenyl 3,6-di-*O*-acetyl-1-thio-β-D-galactopyranoside** (**6**) ^{1}H NMR (400 MHz) in $CDCl_3$

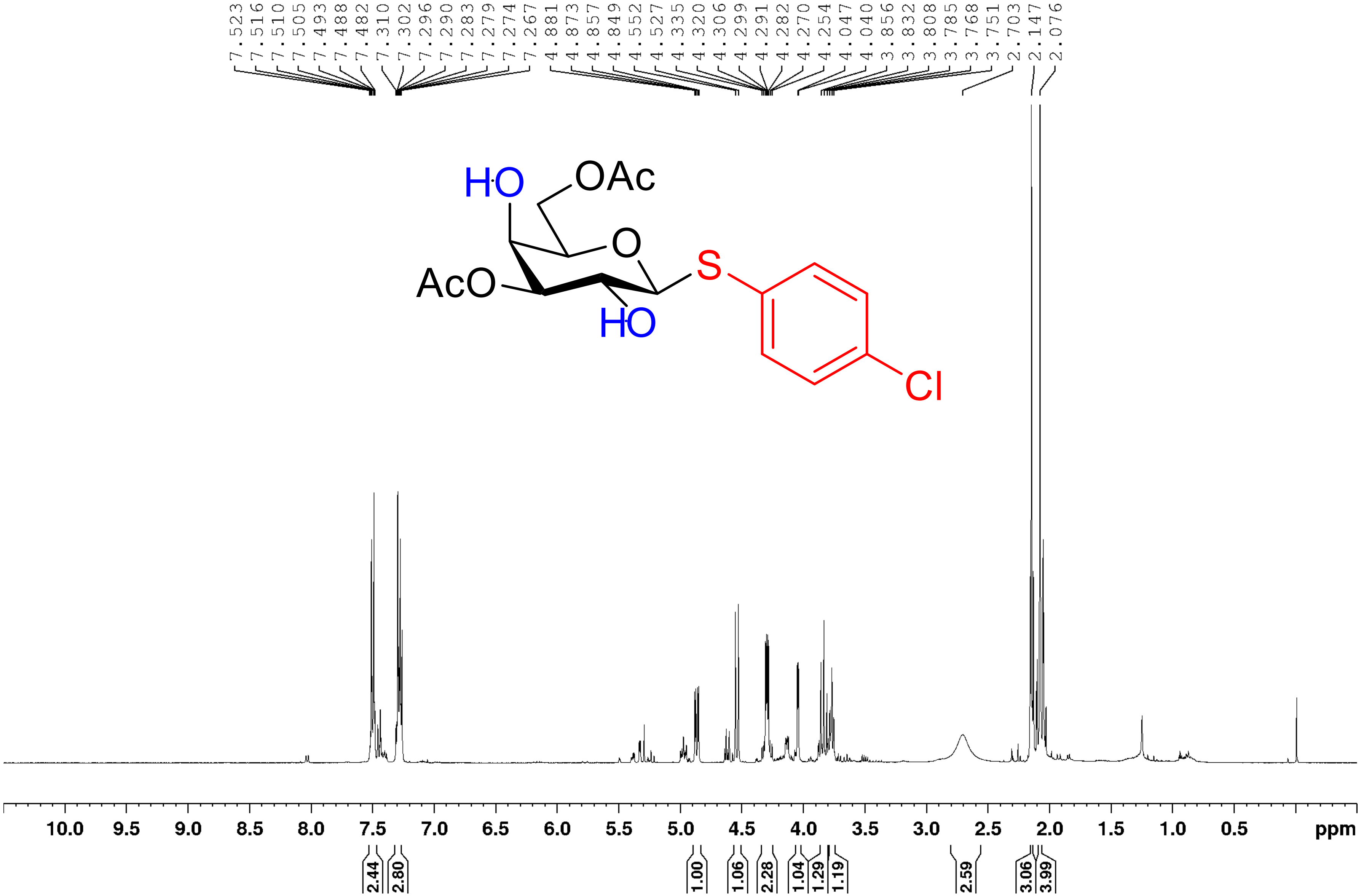

Figure S19: Mixture, where dominant is **4-chlorophenyl 3,6-di-*O*-acetyl-1-thio-β-D-galactopyranoside** (**6**) ^{1}H-^{1}H COSY NMR (400 MHz) in $CDCl_3$

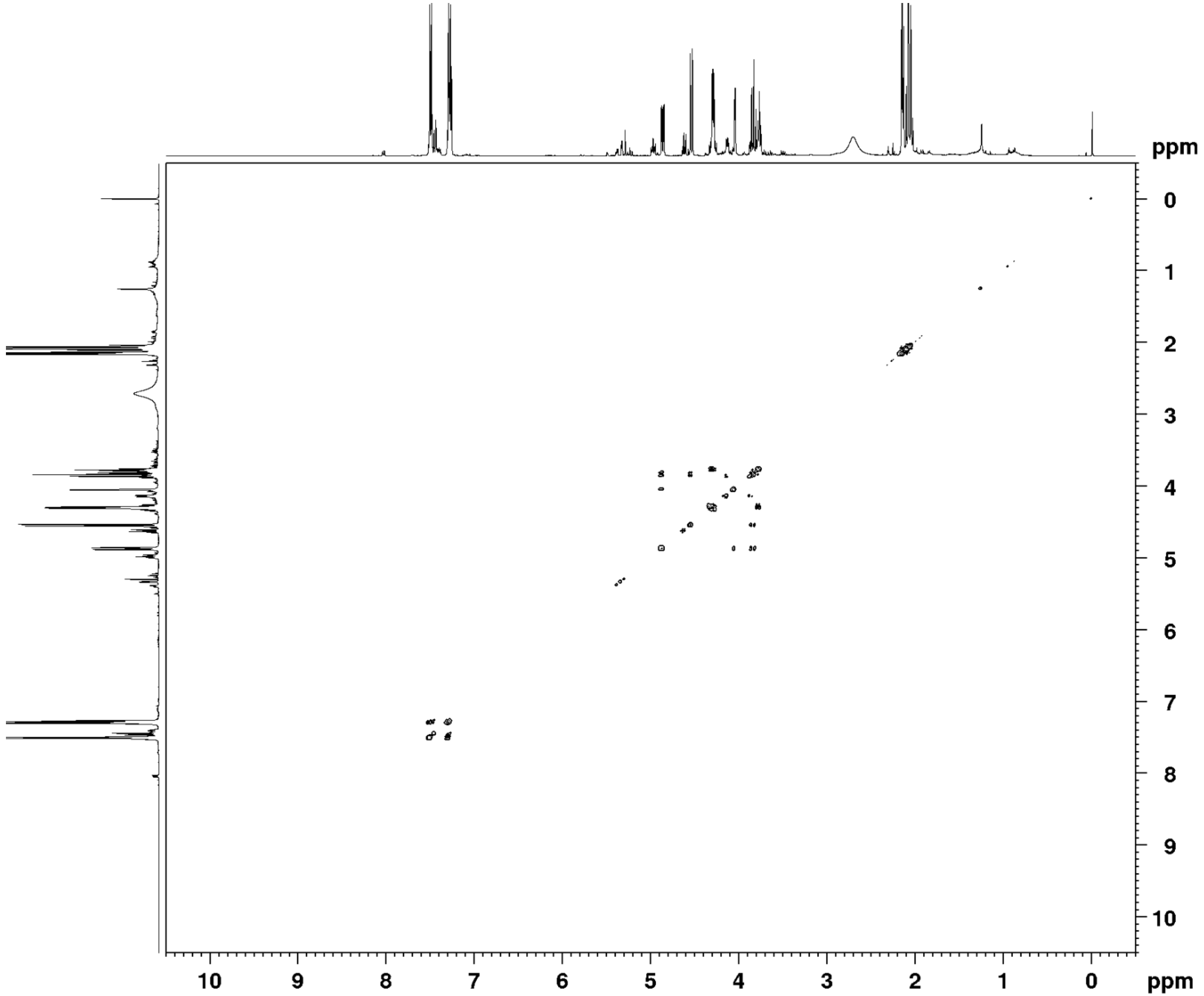

Figure S20: Mixture, where dominant is **4-chlorophenyl 3,6-di-*O*-acetyl-1-thio-β-D-galactopyranoside** (**6**) ^{1}H-$^{13}C\{^{1}H\}$ HSQC NMR (400 & 101 MHz) in $CDCl_3$

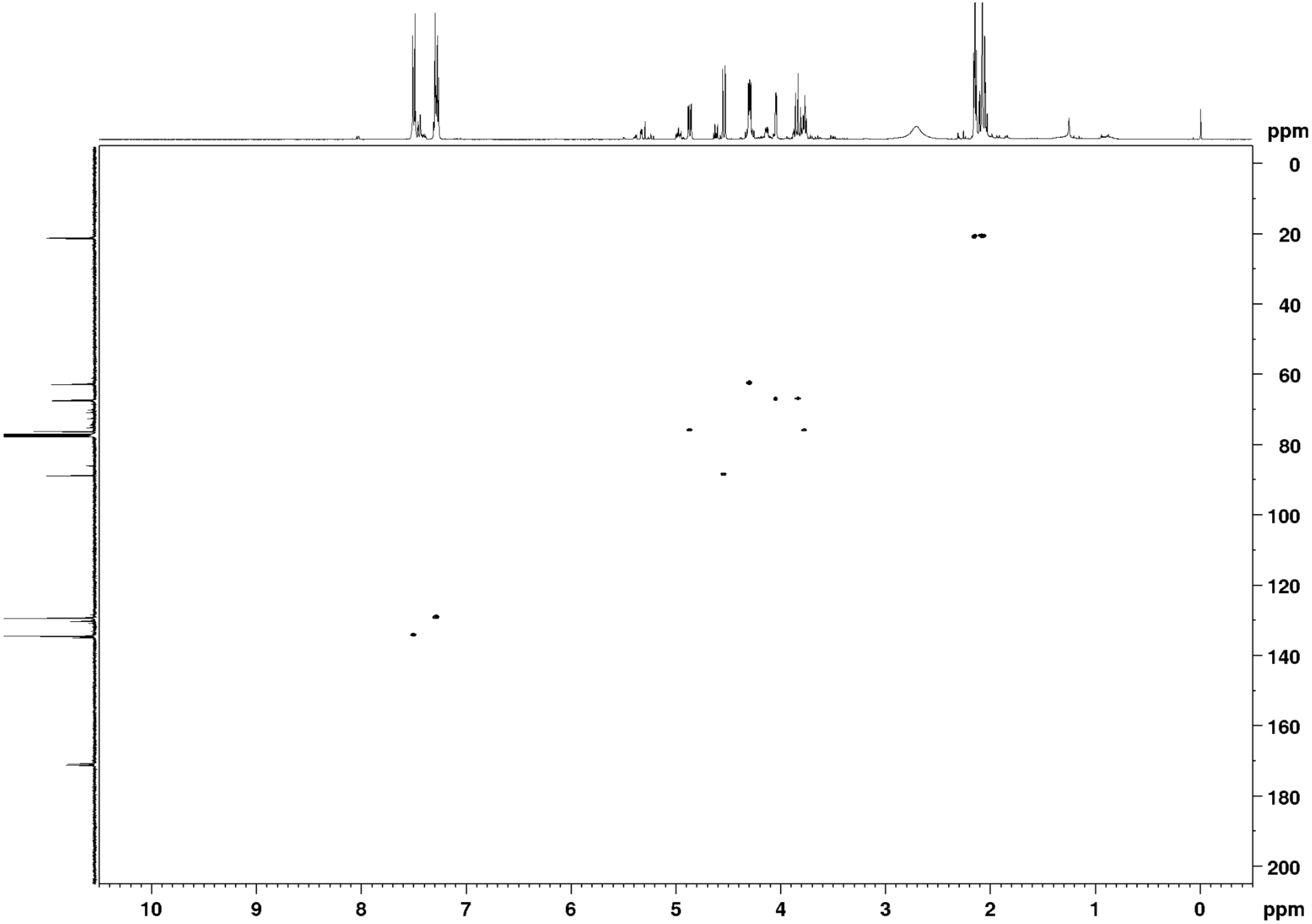

Figure S21: Mixture, where dominant is **4-chlorophenyl 3,6-di-*O*-acetyl-1-thio-β-D-galactopyranoside** (**6**) ^{1}H-$^{13}C\{^{1}H\}$ HMBC NMR (400 & 101 MHz) in $CDCl_3$

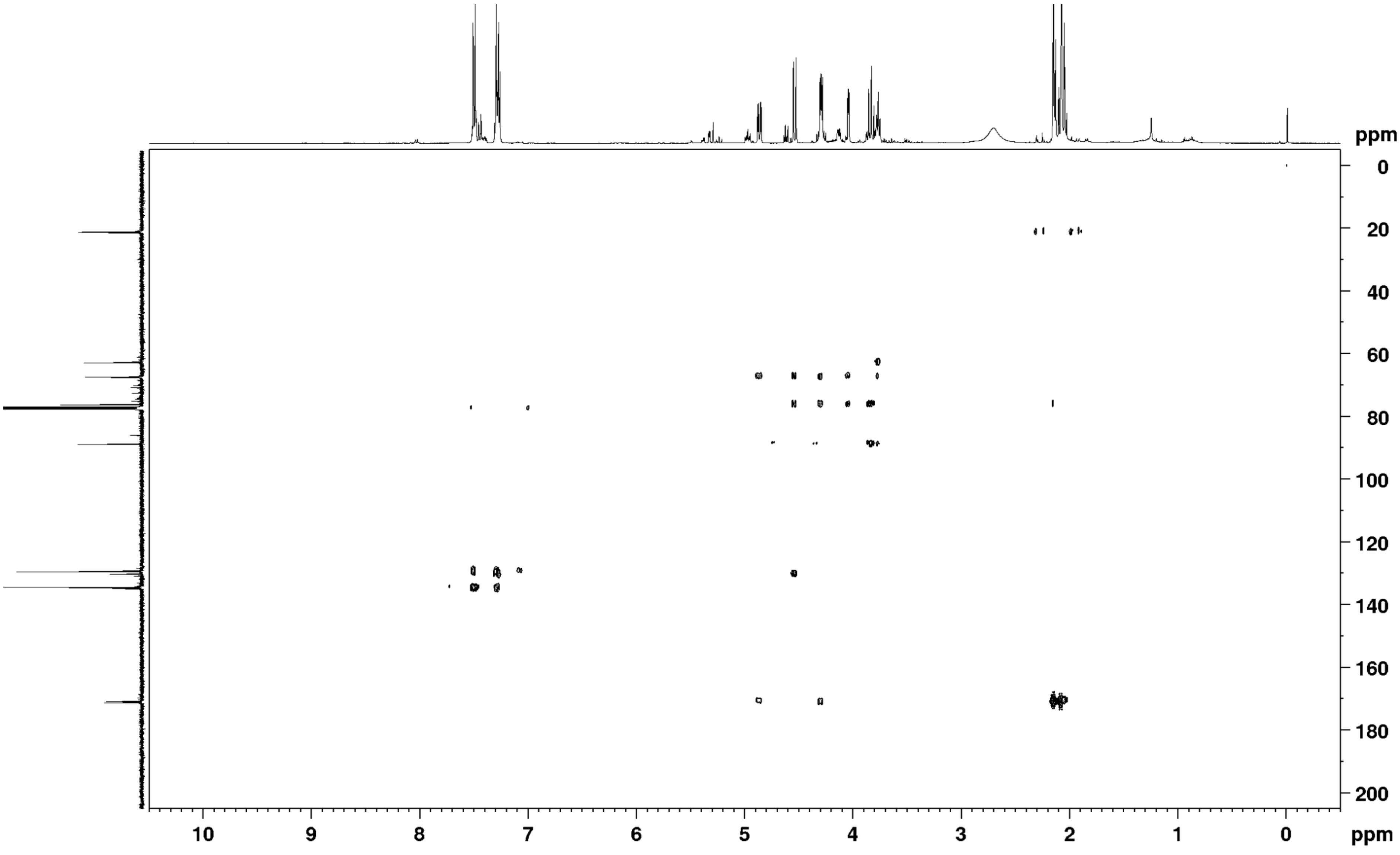

Figure S22: Mixture, where dominant is **4-chlorophenyl 3,6-di-*O*-acetyl-1-thio-β-D-galactopyranoside** (**6**) $^{13}C\{^{1}H\}$ NMR (101 MHz) in $CDCl_3$

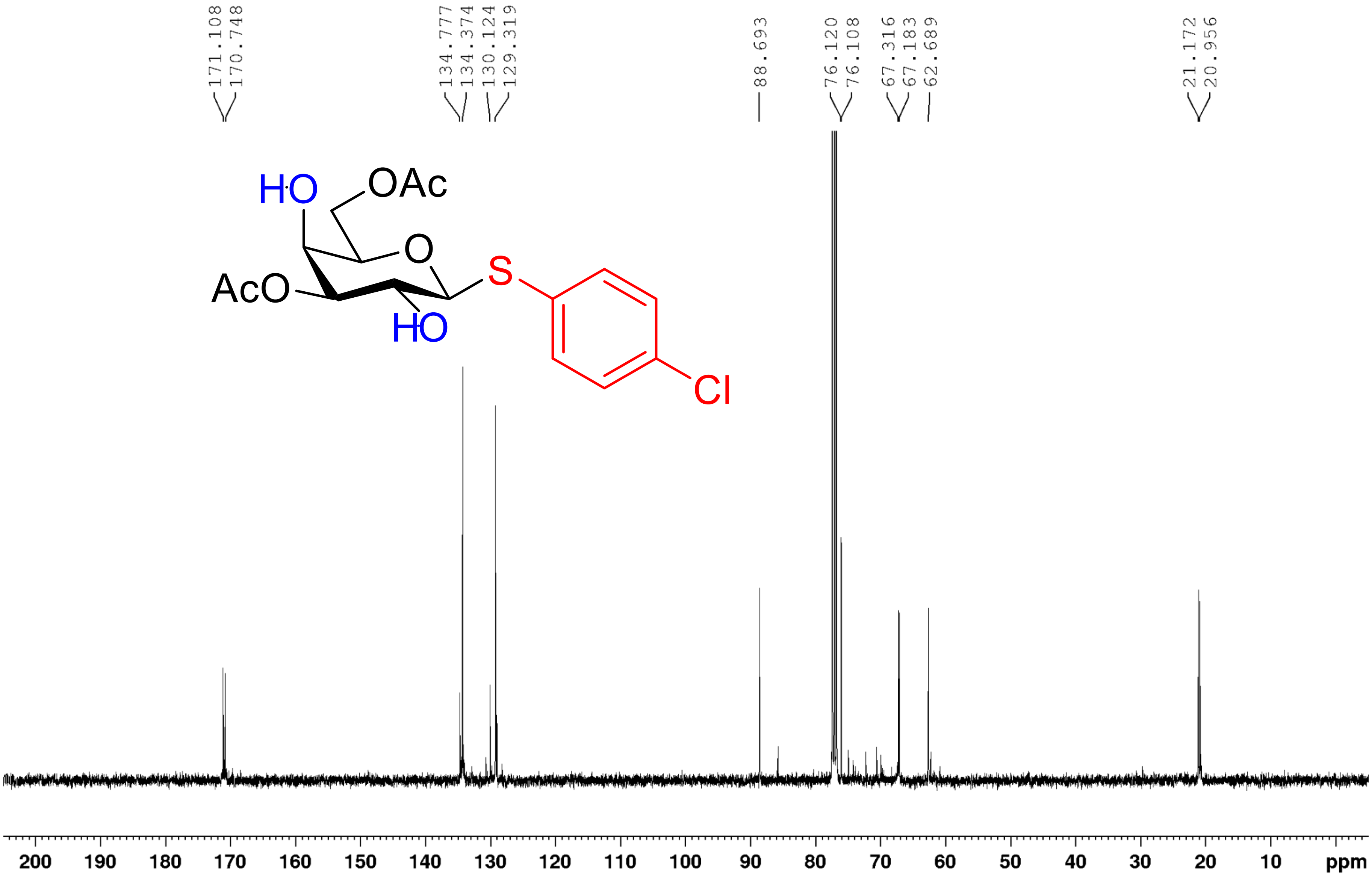

Figure S23: Phenyl 4,6-*O*-ethylidene-1-thio-β-D-galactopyranoside (7) ^{1}H NMR (400 MHz) in MeOD

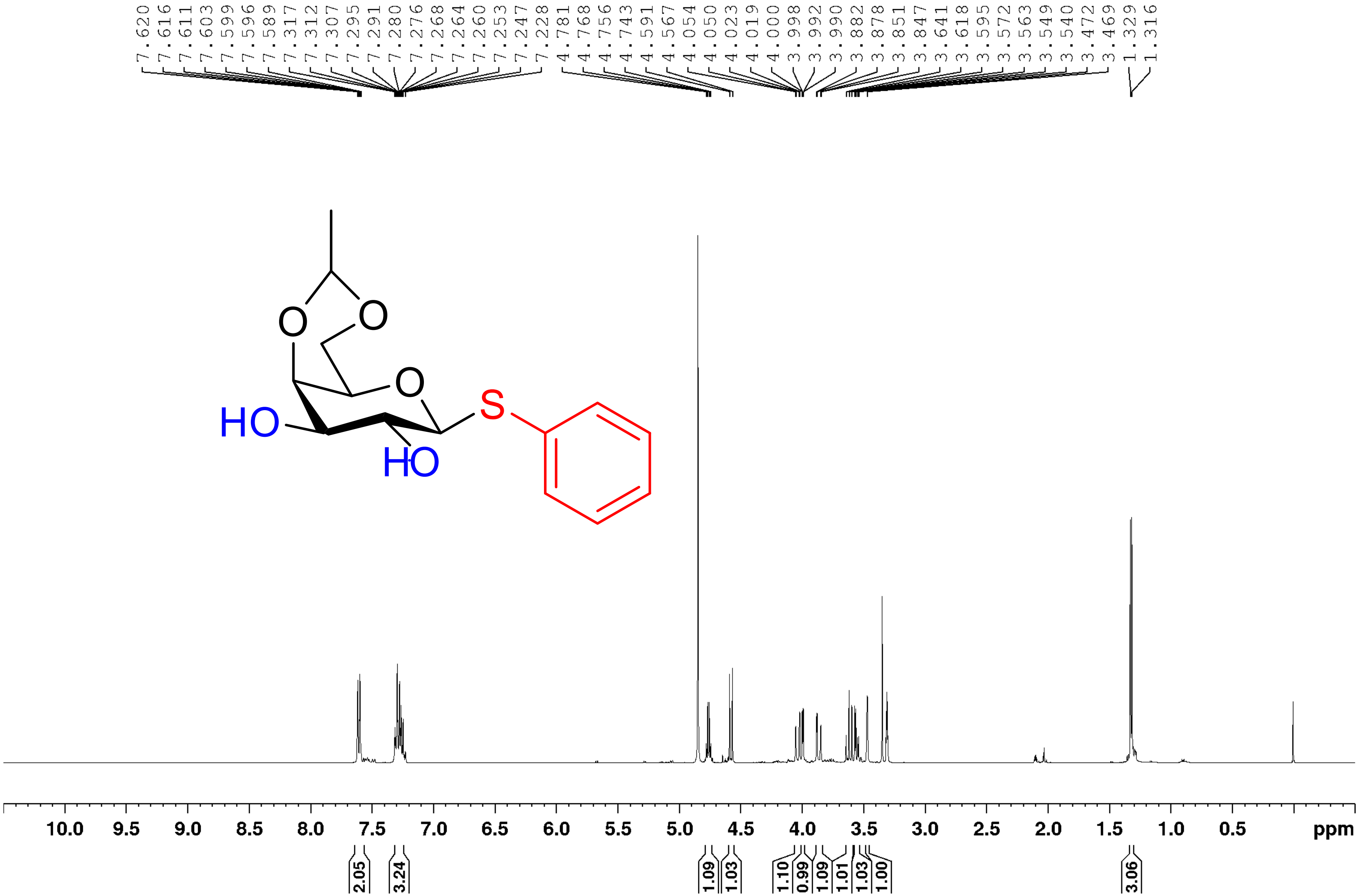

Figure S24: Phenyl 4,6-*O*-ethylidene-1-thio-β-D-galactopyranoside (**7**) ^{1}H-^{1}H COSY NMR (400 MHz) in MeOD

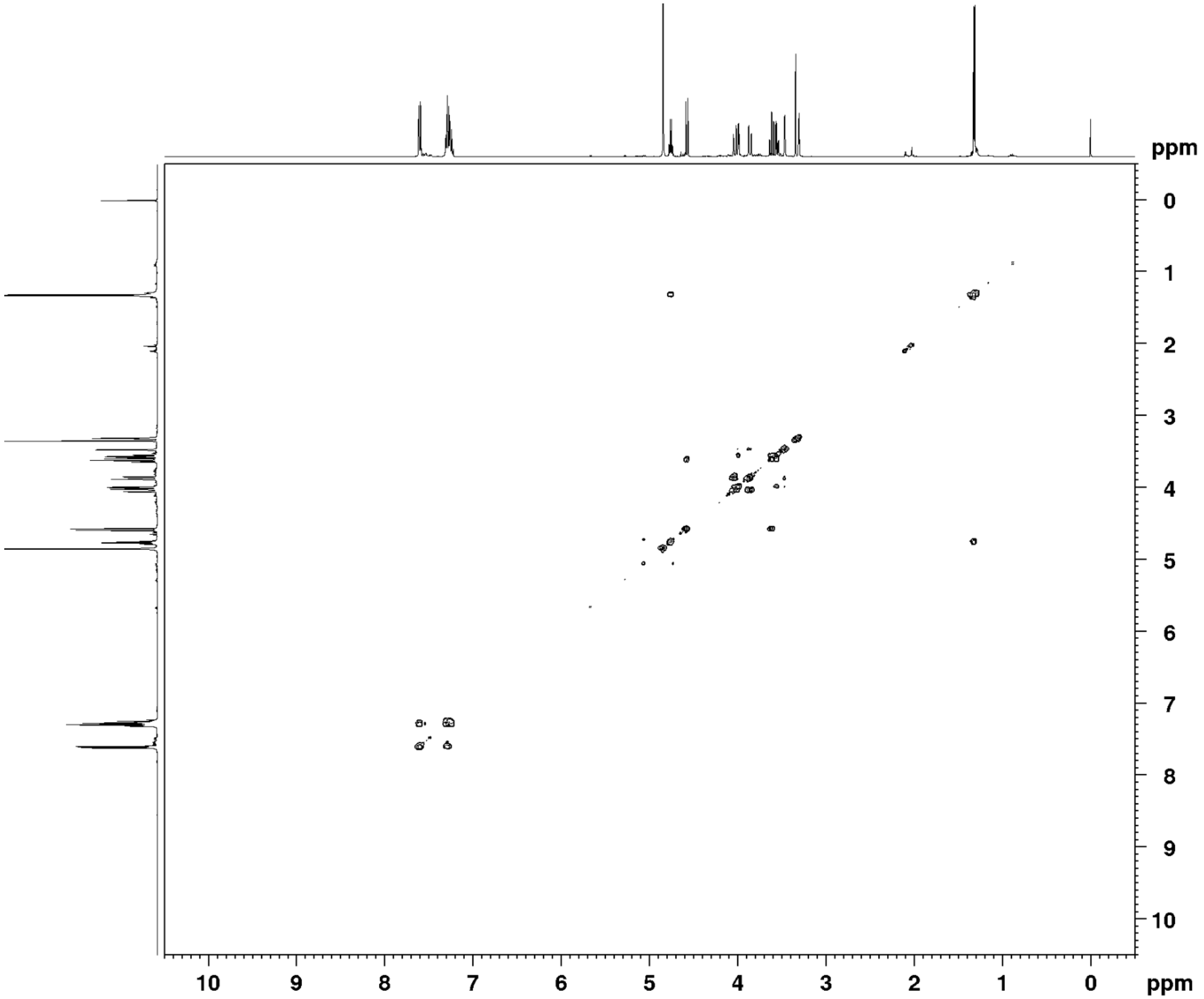

Figure S25: Phenyl 4,6-*O*-ethylidene-1-thio-β-D-galactopyranoside (7) ^{1}H-$^{13}C\{^{1}H\}$ HSQC NMR (400 & 101 MHz) in MeOD

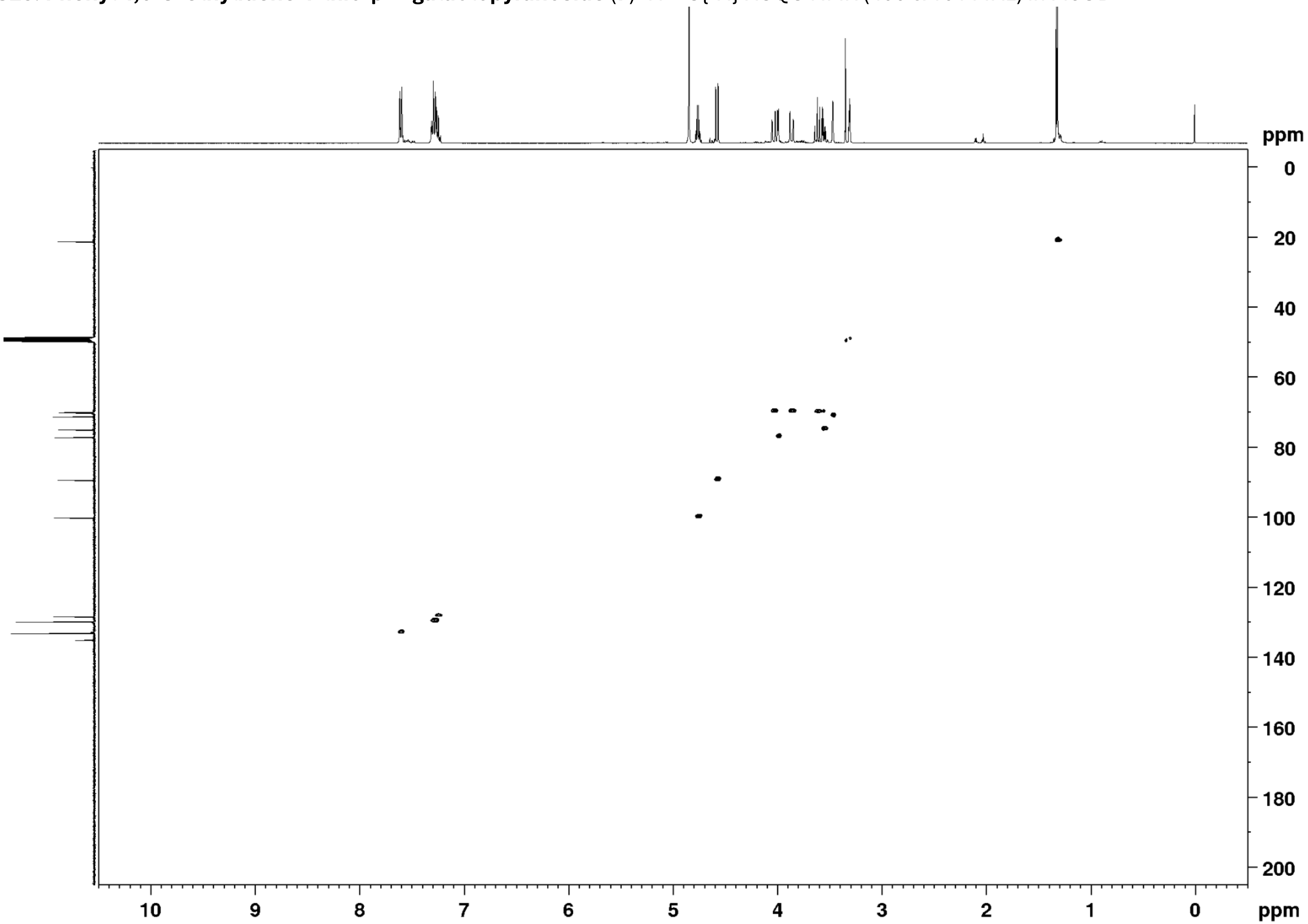

Figure S26: Phenyl 4,6-*O*-ethylidene-1-thio-β-D-galactopyranoside (7) ^{1}H-$^{13}C\{^{1}H\}$ HMBC NMR (400 & 101 MHz) in MeOD

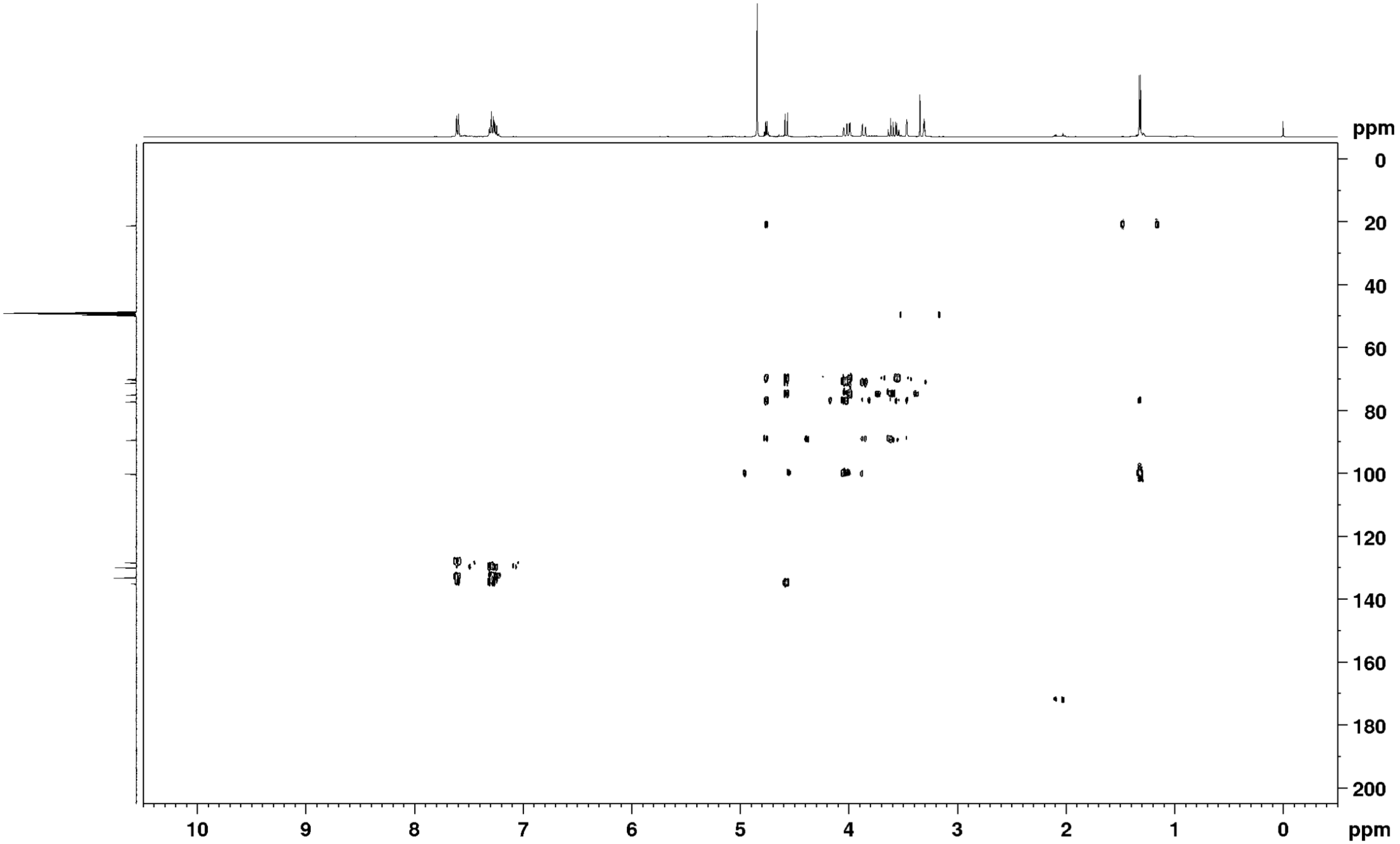

Figure S27: Phenyl 4,6-*O*-ethylidene-1-thio-β-D-galactopyranoside (7) $^{13}C\{^{1}H\}$ NMR (101 MHz) in MeOD

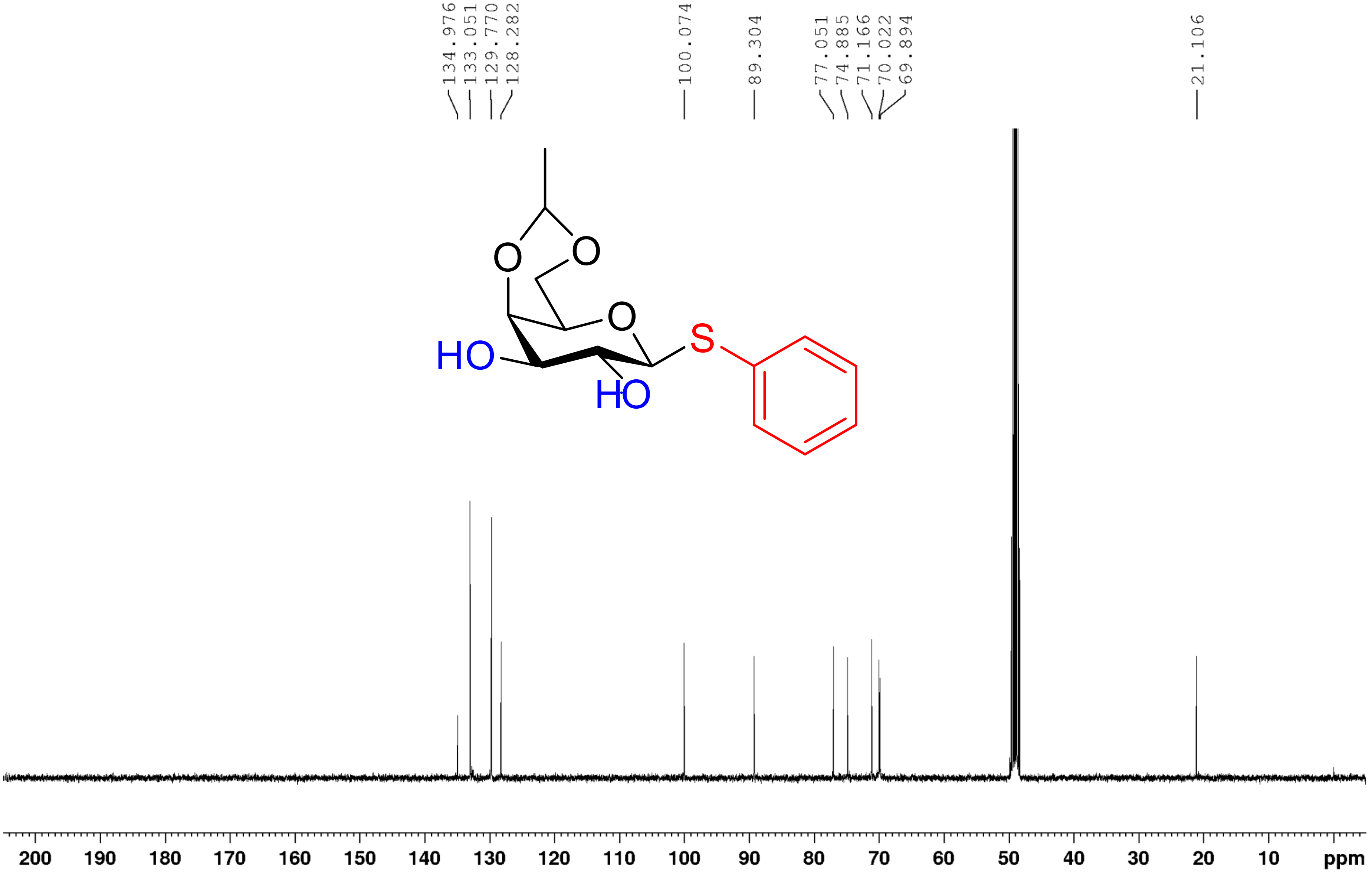

Figure S28: 2,6-dimethylphenyl 1-thio-β-D-galactopyranoside (**8**) ^{1}H NMR (400 MHz) in DMSO-d_6

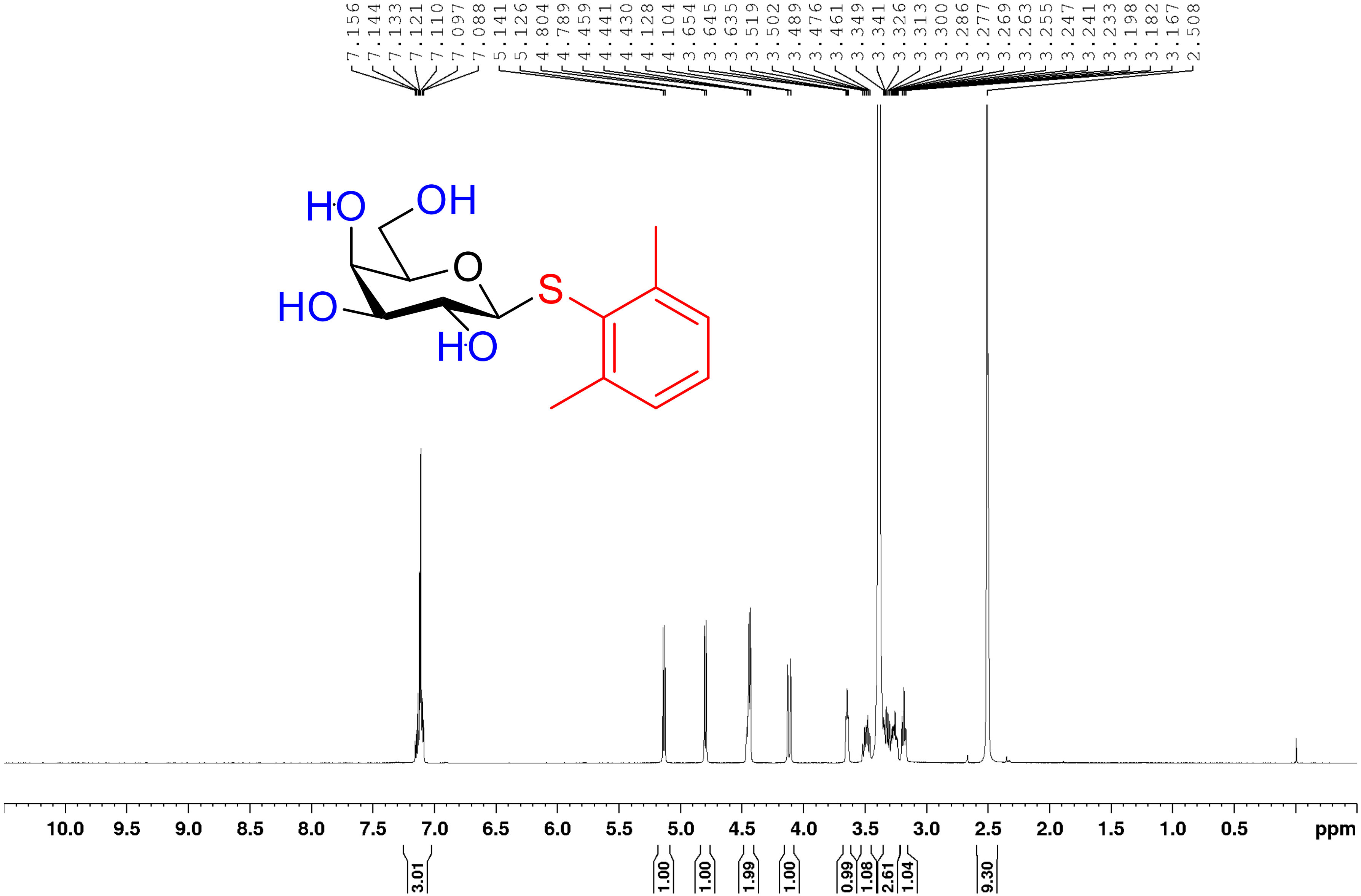

Figure S29: 2,6-dimethylphenyl 2,6-di-*O*-acetyl-1-thio-β-D-galactopyranoside (9) ^{1}H NMR (400 MHz) in MeOD

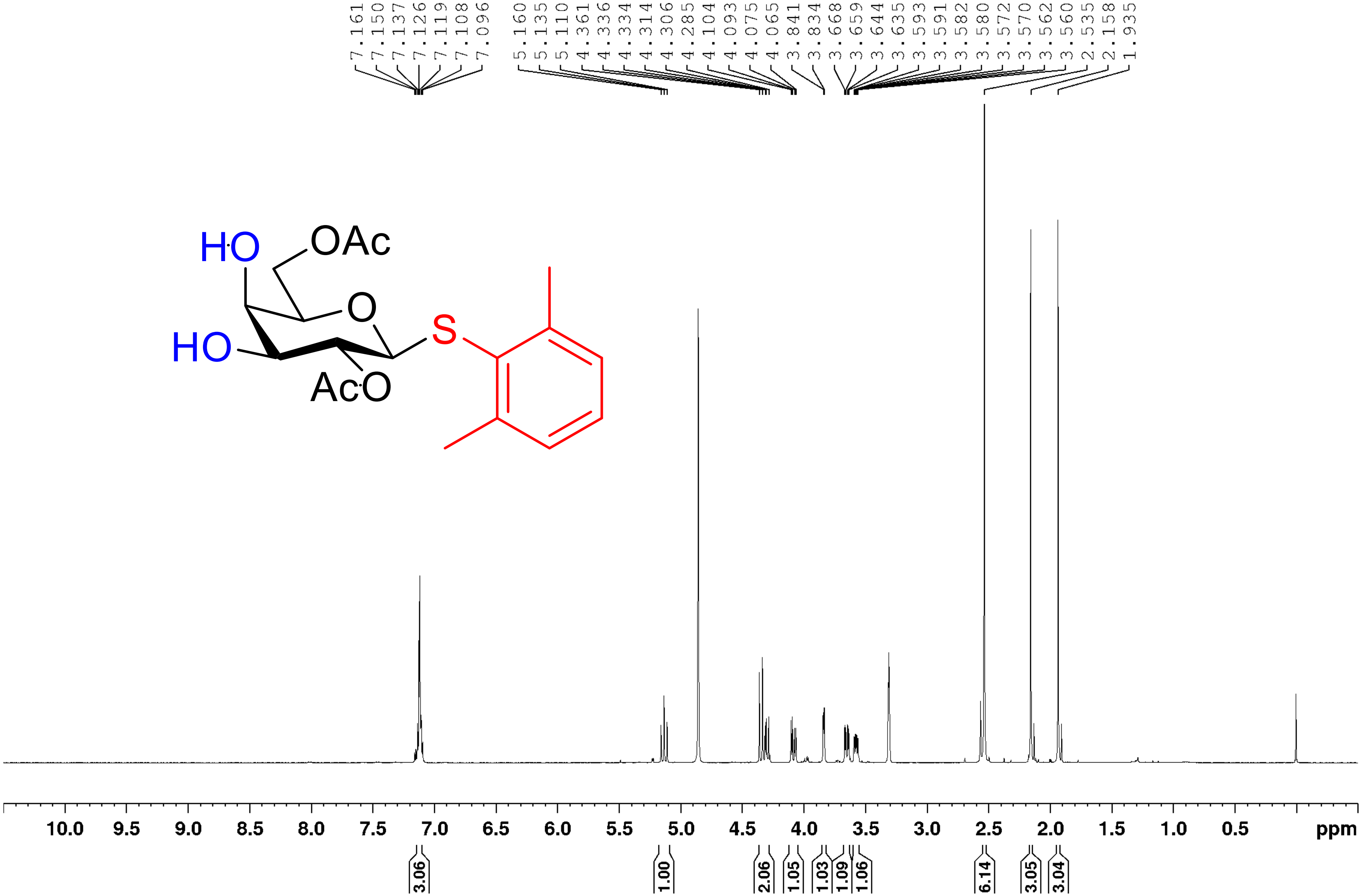

Figure S30: 2,6-dimethylphenyl 2,6-di-*O*-acetyl-1-thio-β-D-galactopyranoside (9) ^{1}H-^{1}H COSY NMR (400 MHz) in MeOD

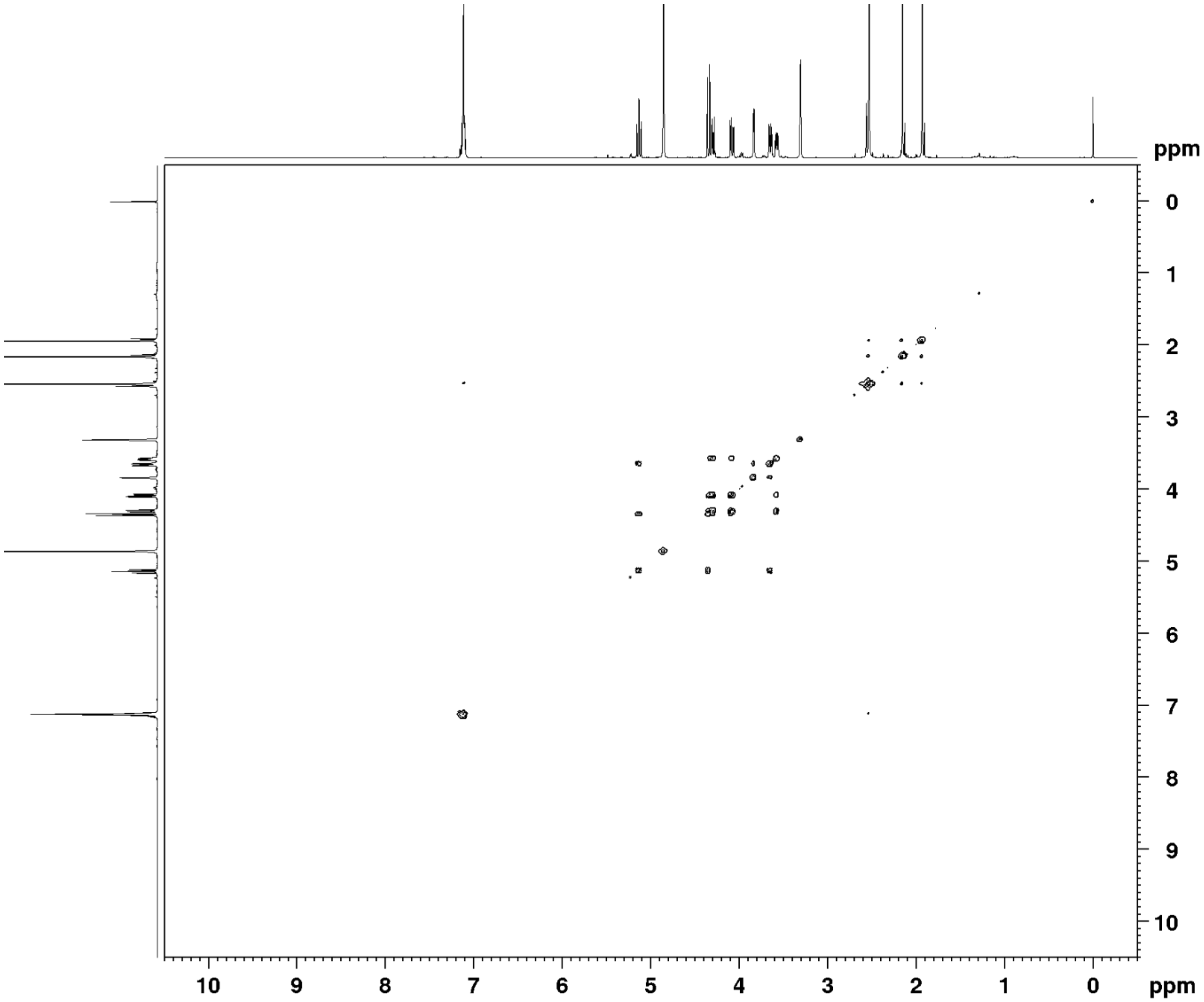

Figure S31: 2,6-dimethylphenyl 2,6-di-*O*-acetyl-1-thio-β-D-galactopyranoside (9) ^{1}H-$^{13}C\{^{1}H\}$ HSQC NMR (400 & 101 MHz) in MeOD

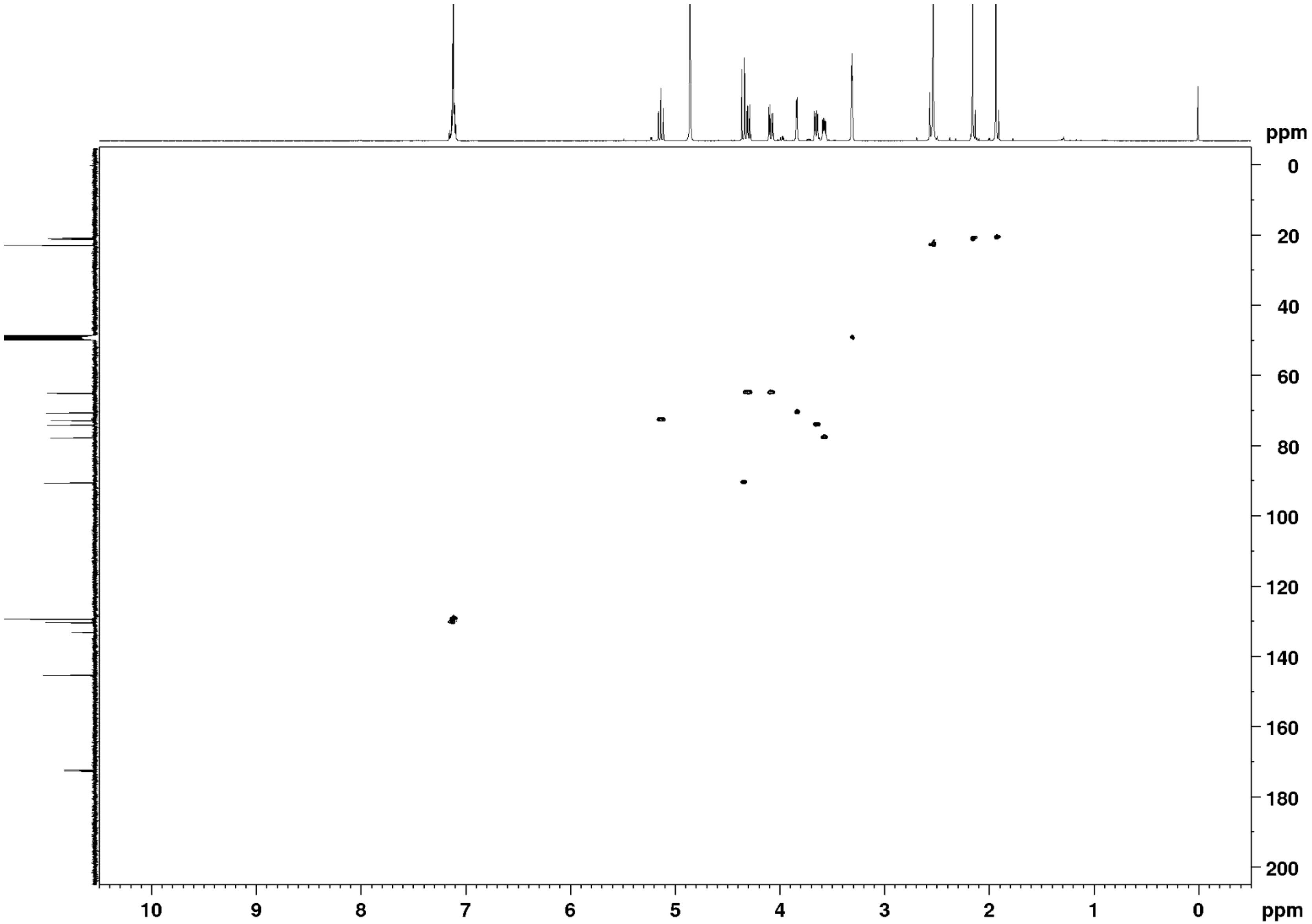

Figure S32: 2,6-dimethylphenyl 2,6-di-*O*-acetyl-1-thio-β-D-galactopyranoside (9) ^{1}H-$^{13}C\{^{1}H\}$ HMBC NMR (400 & 101 MHz) in MeOD

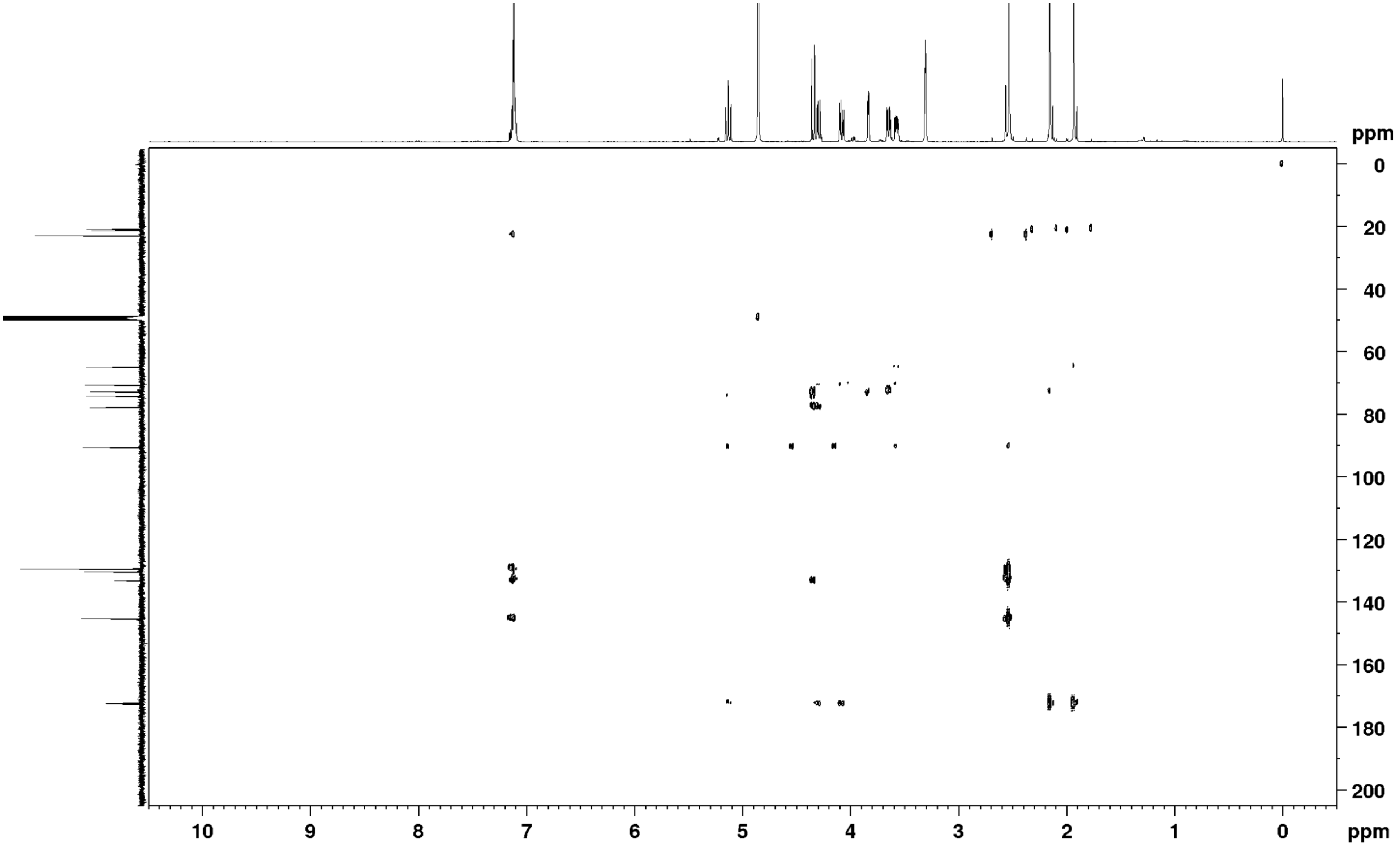

Figure S33: 2,6-dimethylphenyl 2,6-di-*O*-acetyl-1-thio-β-D-galactopyranoside (9) $^{13}C\{^{1}H\}$ NMR (101 MHz) in MeOD

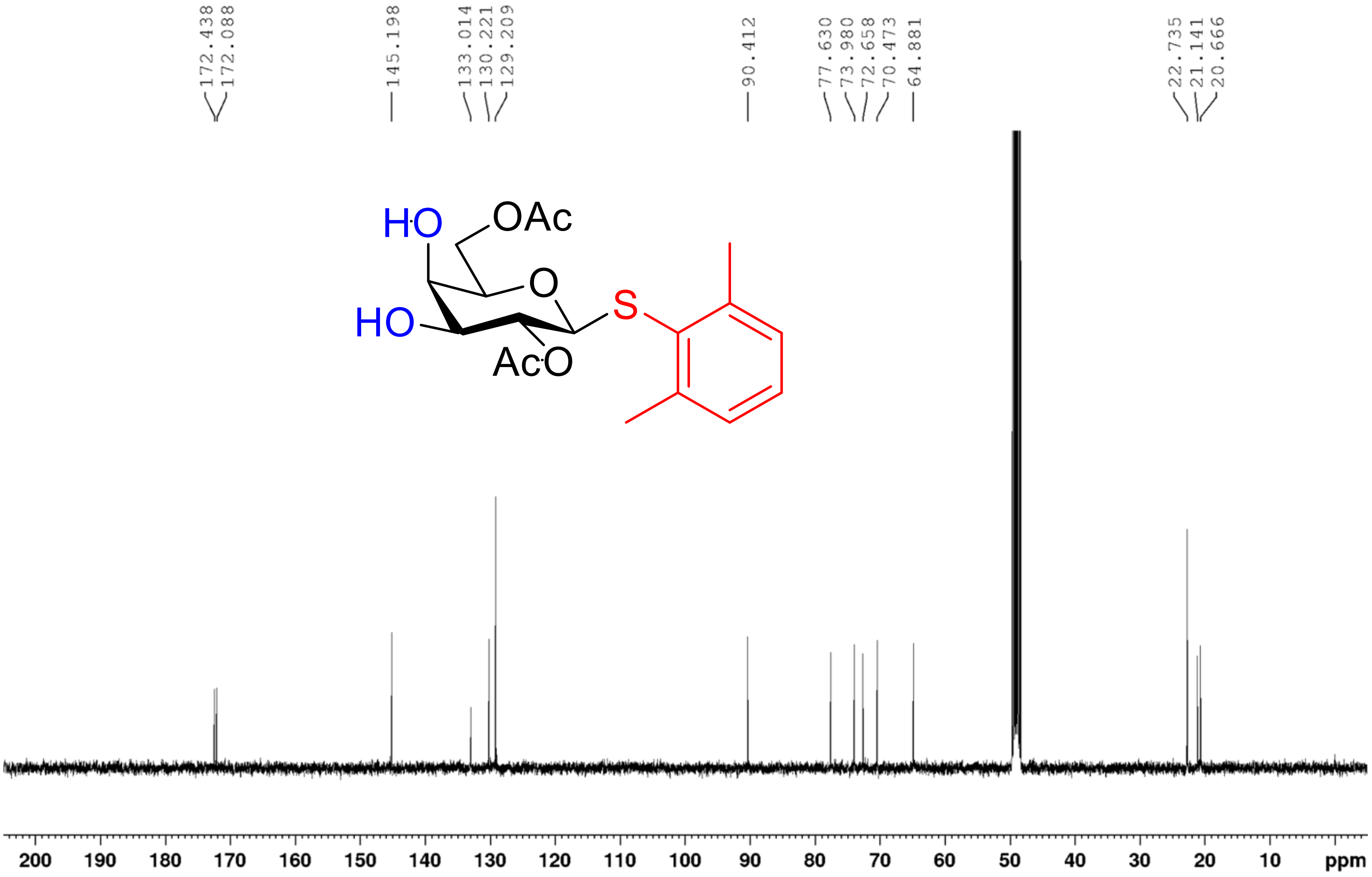

Figure S34: 4-chlorophenyl 1-thio-β-D-glucopyranoside (**10**) ^{1}H NMR (400 MHz) in MeOD

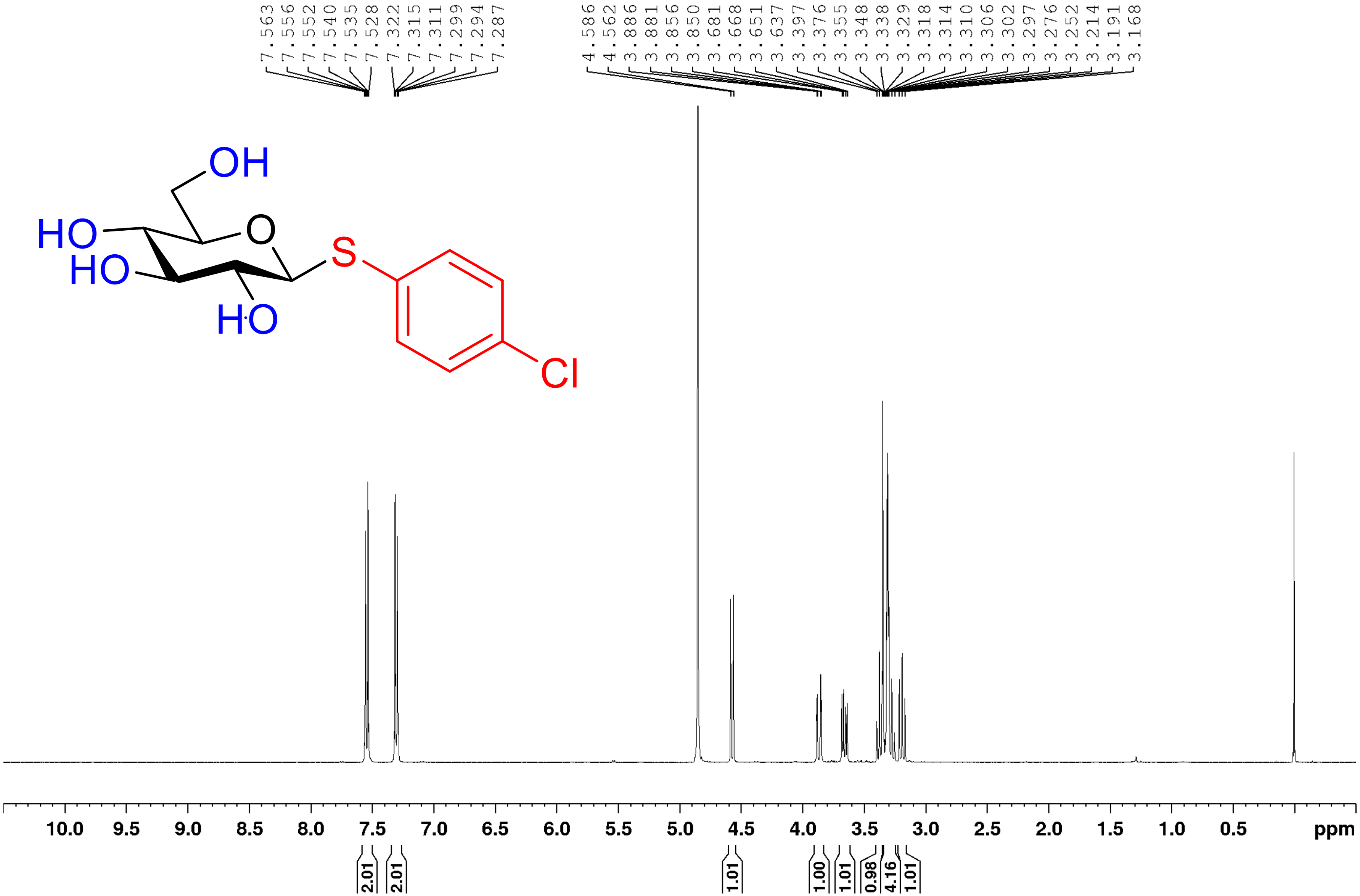

Figure S35: 4-chlorophenyl 6-*O*-acetyl-1-thio-β-D-glucopyranoside (11) ^{1}H NMR (400 MHz) in MeOD

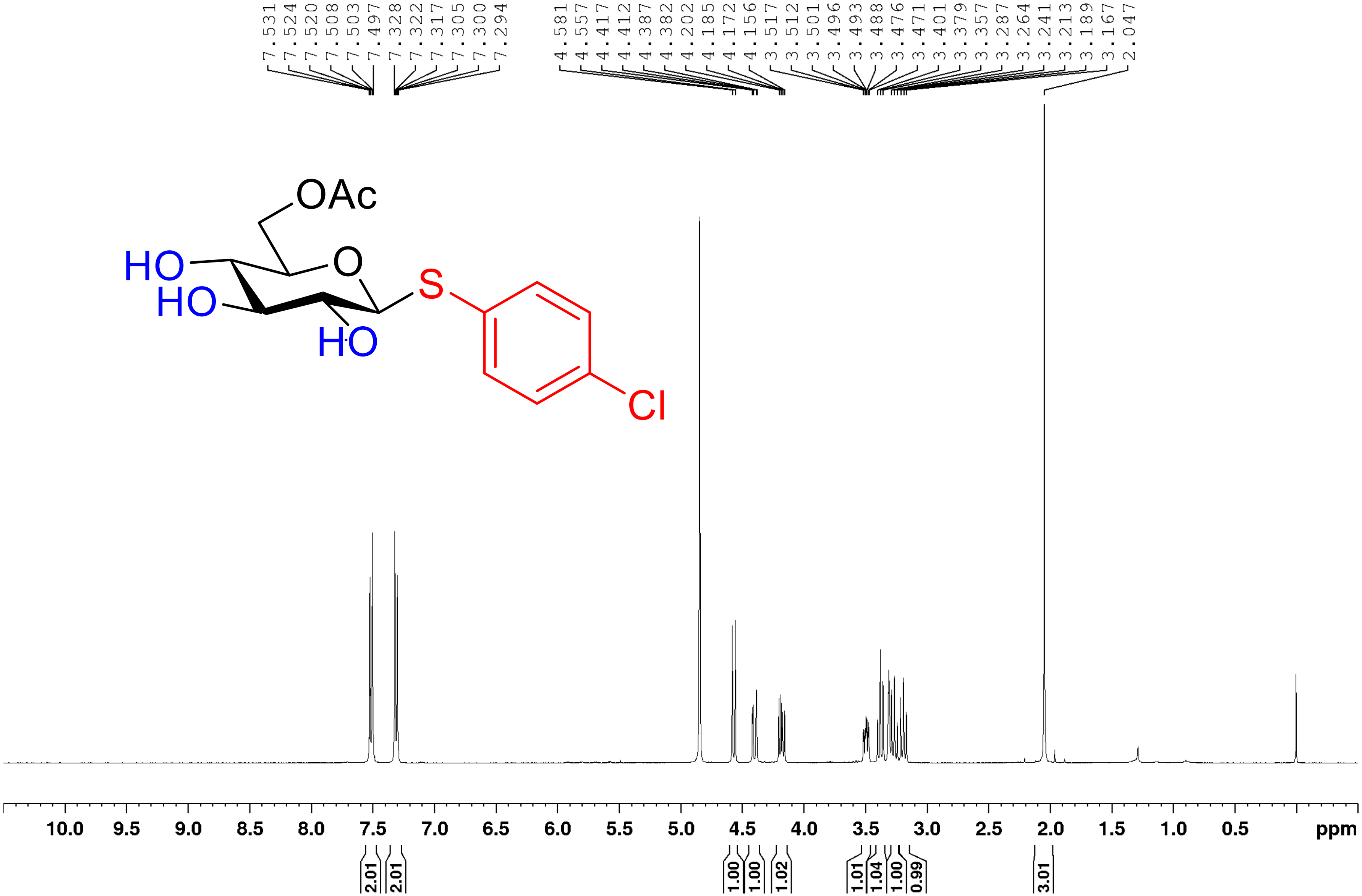

Figure S36: 4-chlorophenyl 6-*O*-acetyl-1-thio-β-D-glucopyranoside (**11**) ^1H-^1H COSY NMR (400 MHz) in MeOD

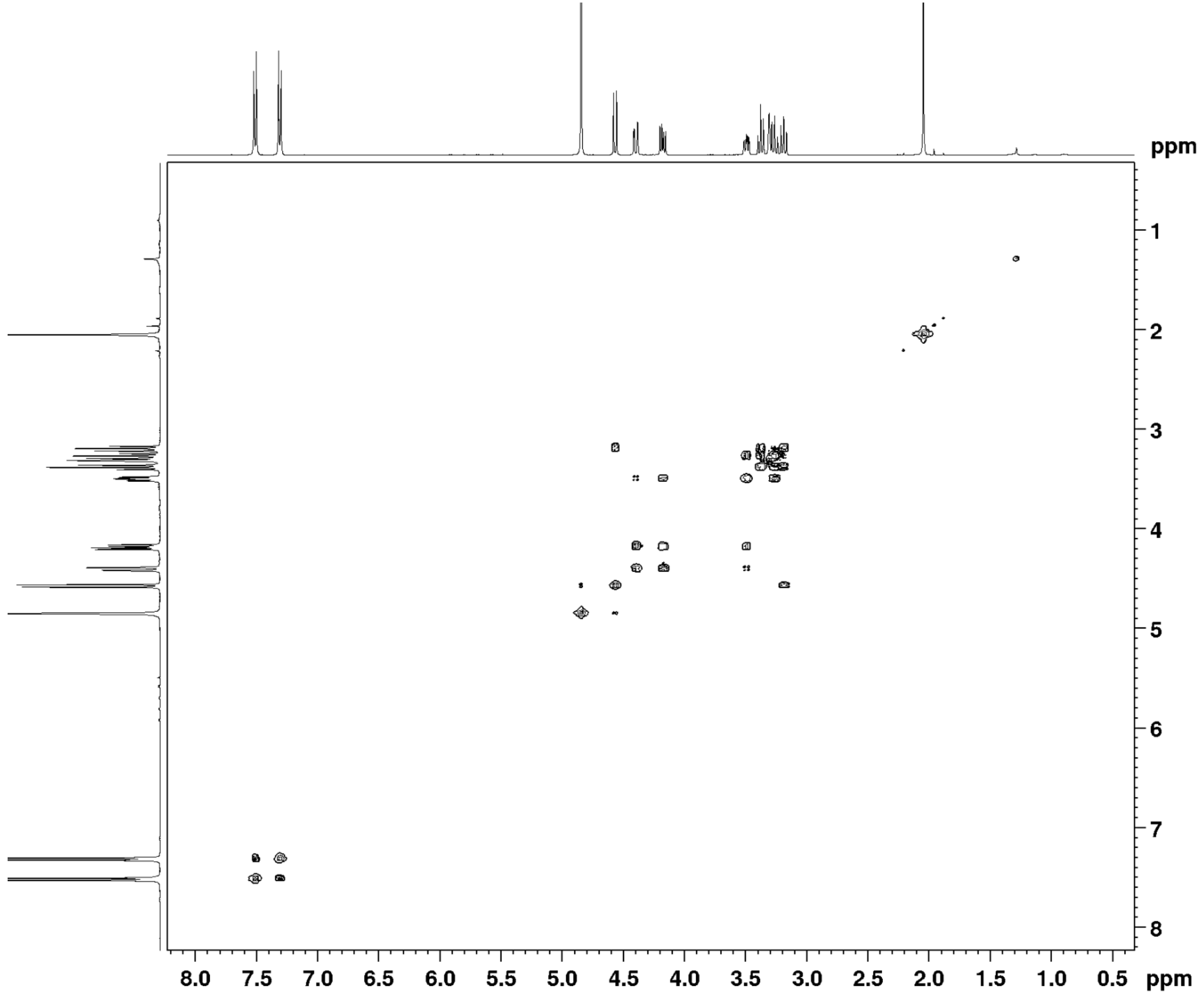

Figure S37: 4-chlorophenyl 6-*O*-acetyl-1-thio-β-D-glucopyranoside (11) ^{1}H-$^{13}C\{^{1}H\}$ HSQC NMR (400 & 101 MHz) in MeOD

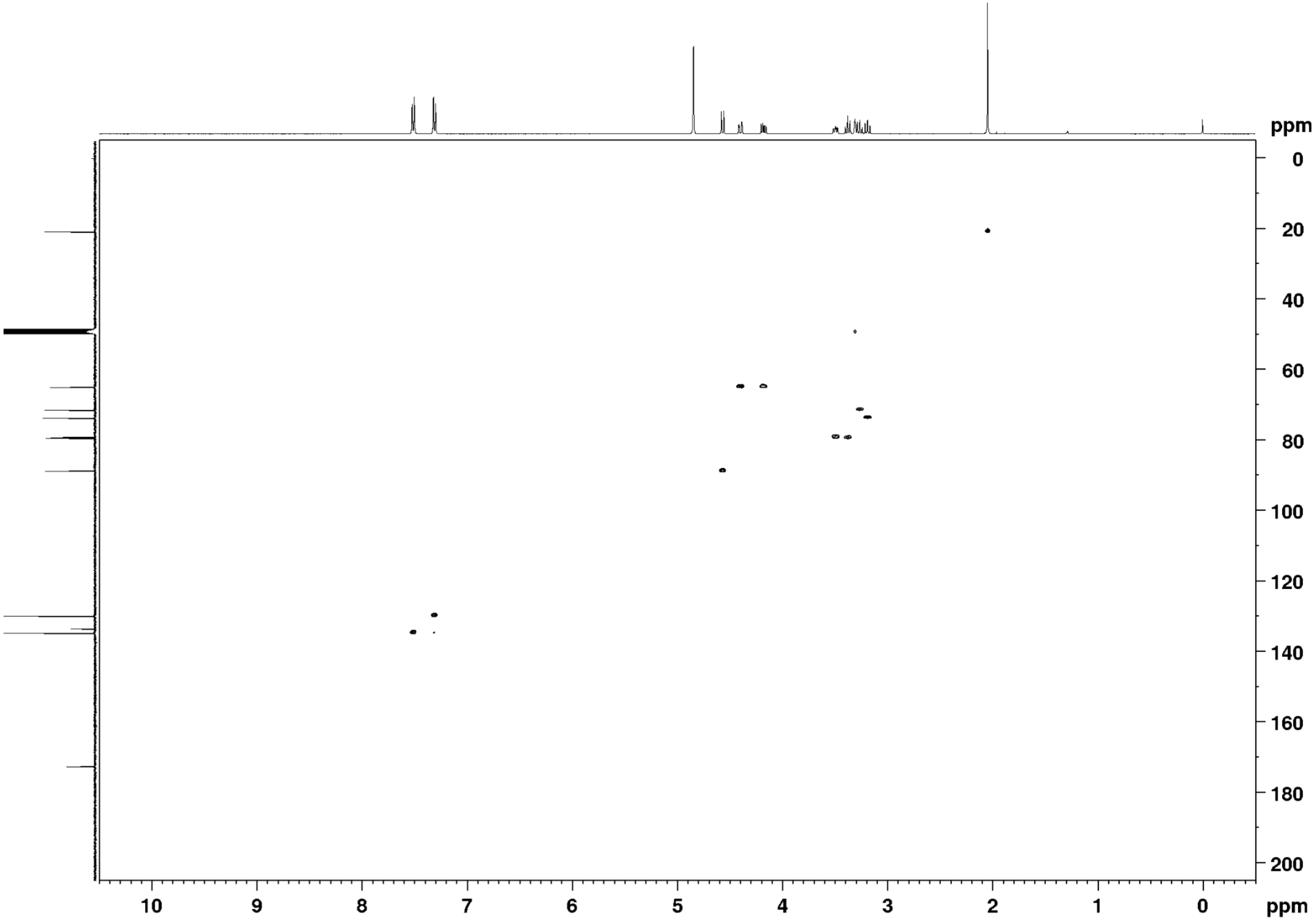

Figure S38: 4-chlorophenyl 6-*O*-acetyl-1-thio-β-D-glucopyranoside (11) ^{1}H-^{13}C{^{1}H} HMBC NMR (400 & 101 MHz) in MeOD

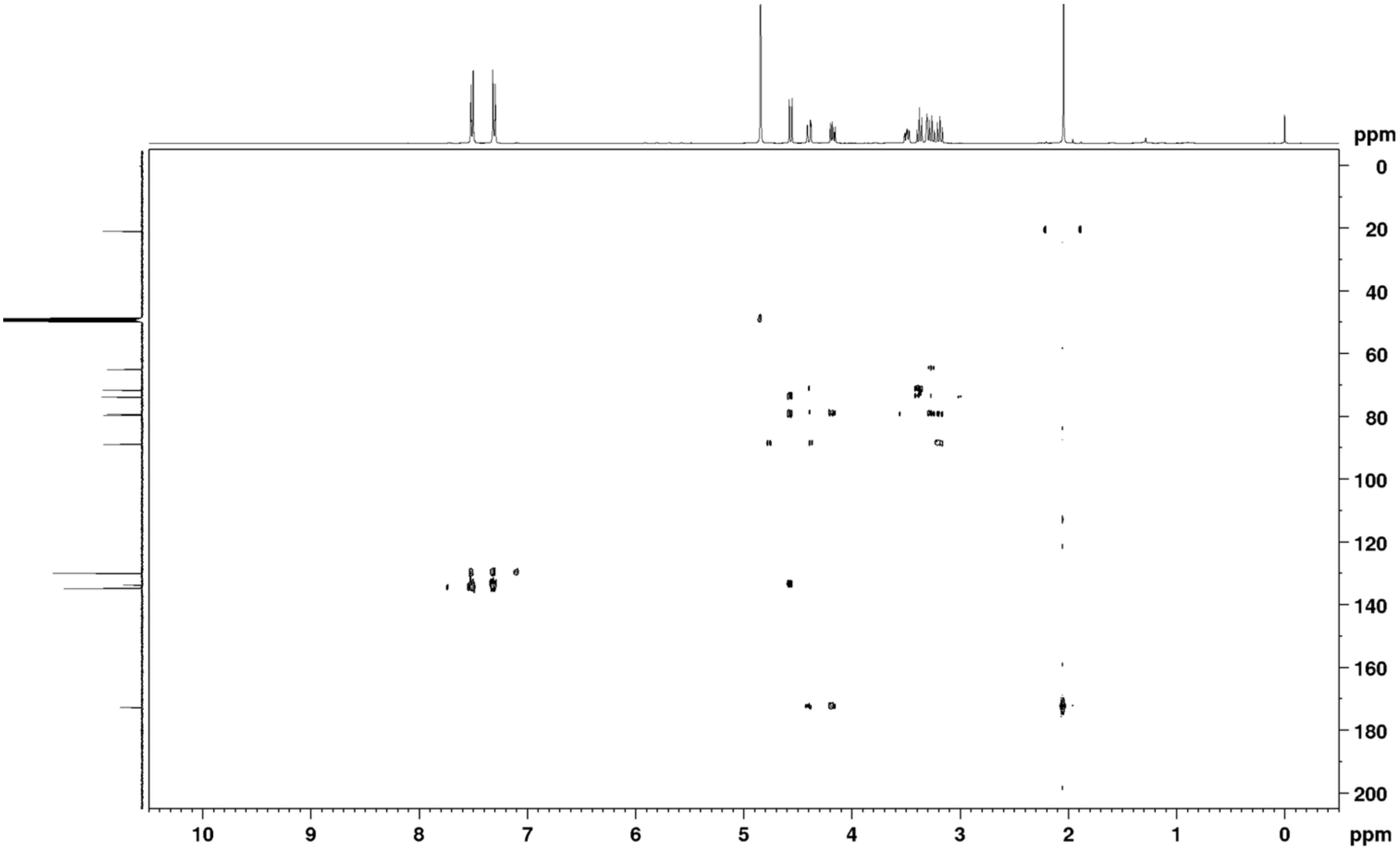

Figure S39: 4-chlorophenyl 6-*O*-acetyl-1-thio-β-D-glucopyranoside (11) $^{13}C\{^{1}H\}$ NMR (101 MHz) in MeOD

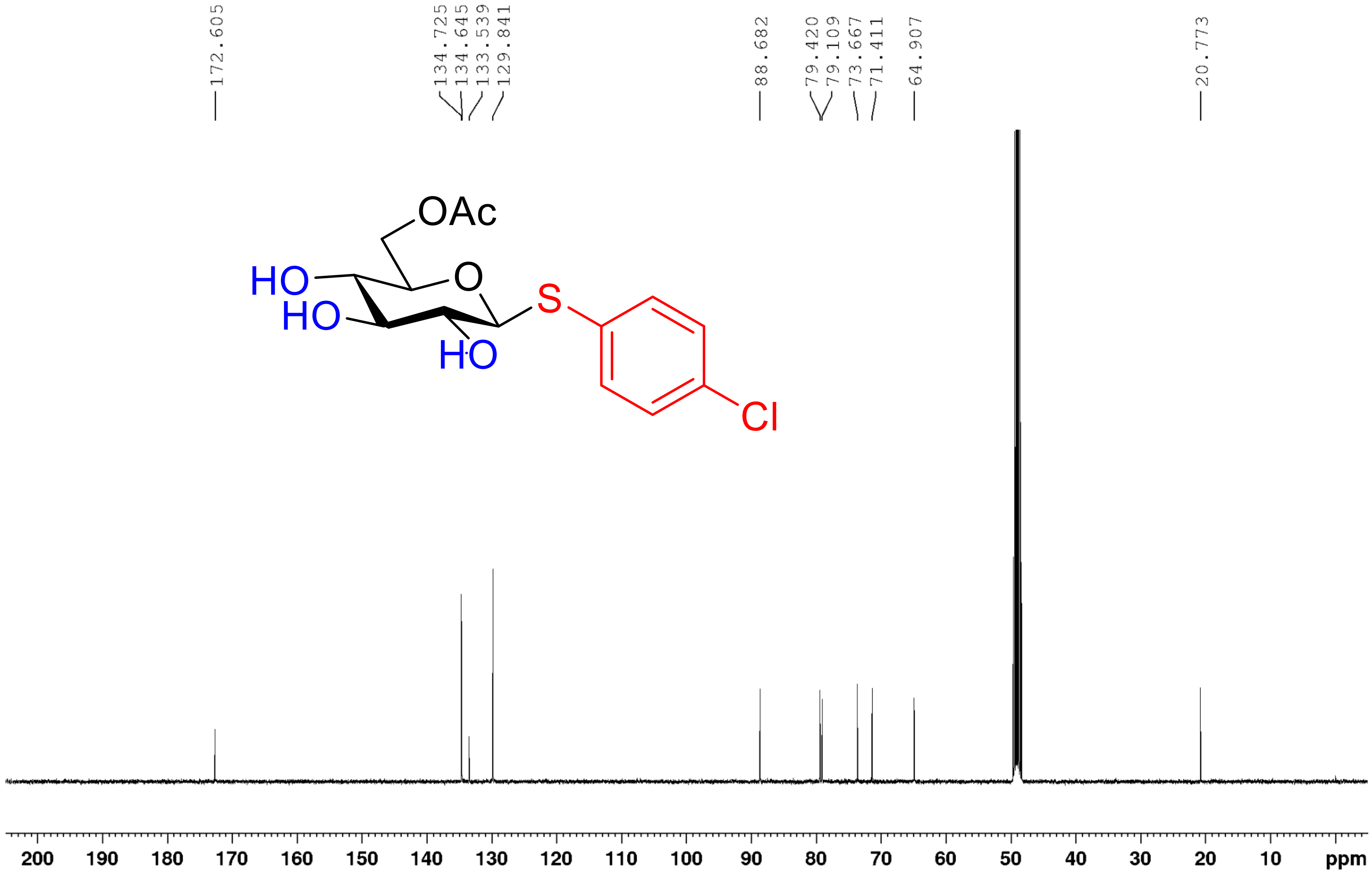

Figure S40: 4-chlorophenyl 2-acetamido-2-deoxy-1-thio-β-D-glucopyranoside (**12**) ^{1}H NMR (400 MHz) in MeOD

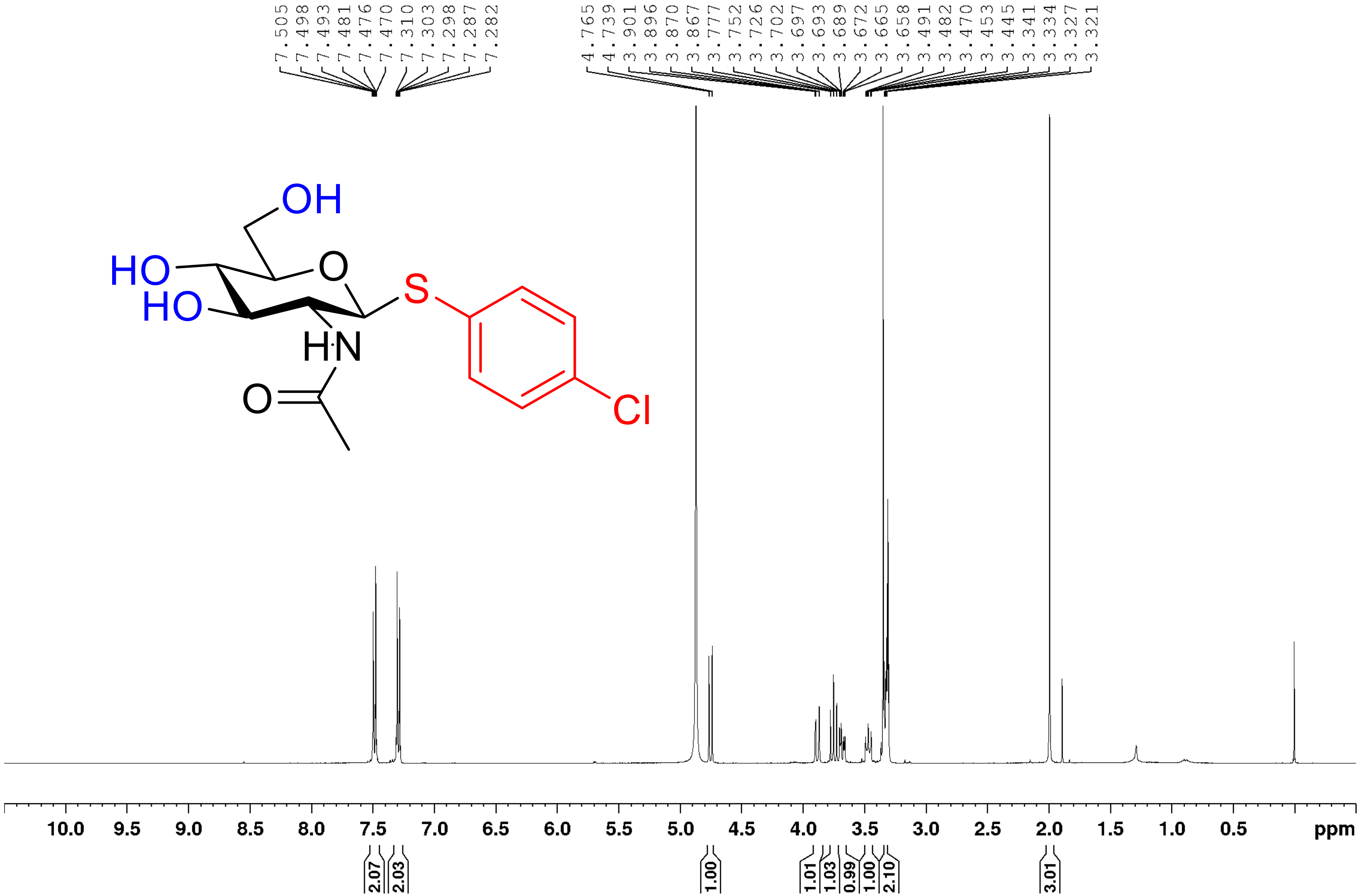

Figure S41: 4-chlorophenyl 2-acetamido-6-*O*-acetyl-2-deoxy-1-thio-β-D-glucopyranoside (13) ^{1}H NMR (400 MHz) in MeOD

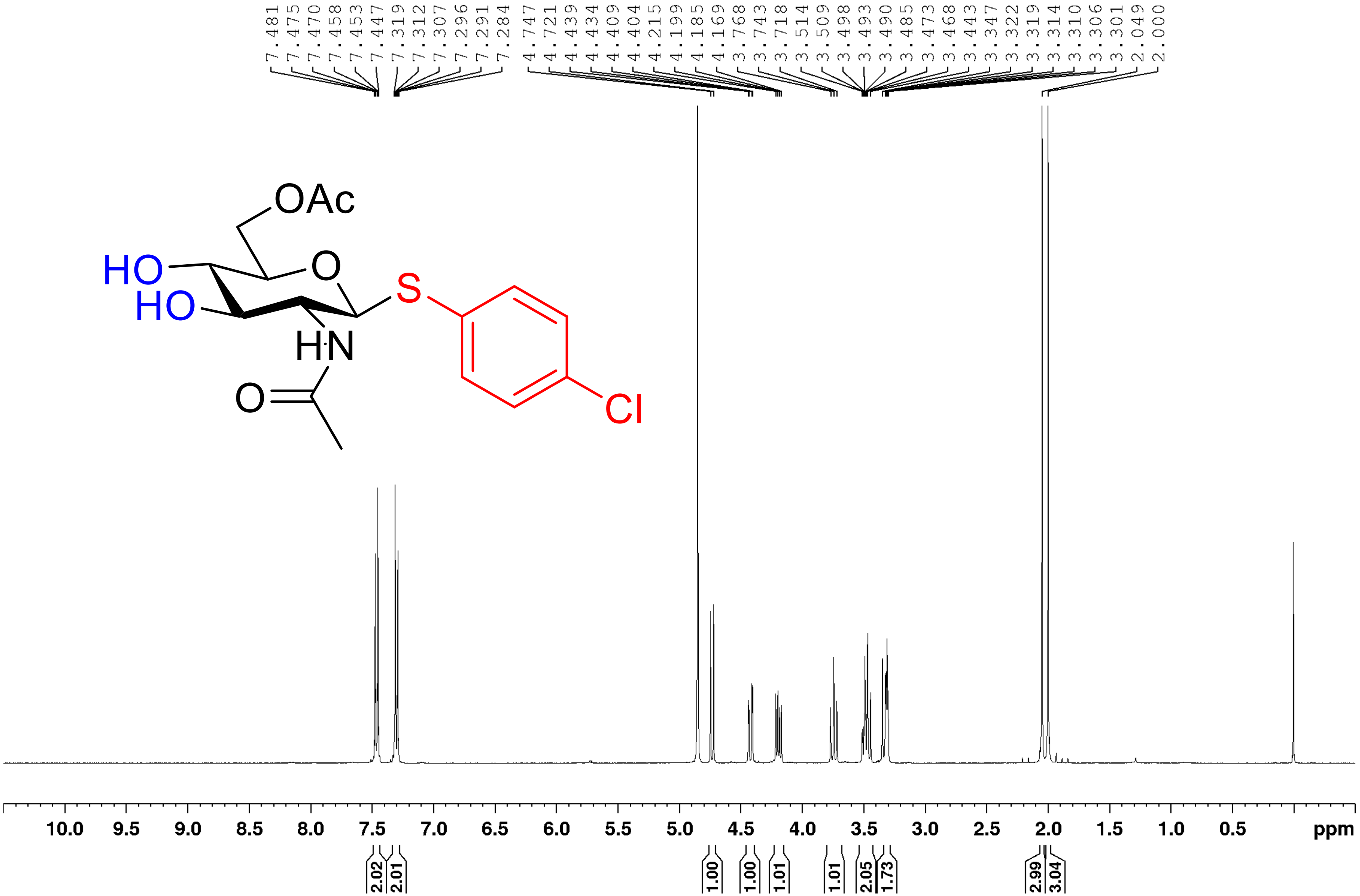

Figure S42: 4-chlorophenyl 2-acetomido-6-*O*-acetyl-2-deoxy-1-thio-β-D-glucopyranoside (13) ^{1}H-^{1}H COSY NMR (400 MHz) in MeOD

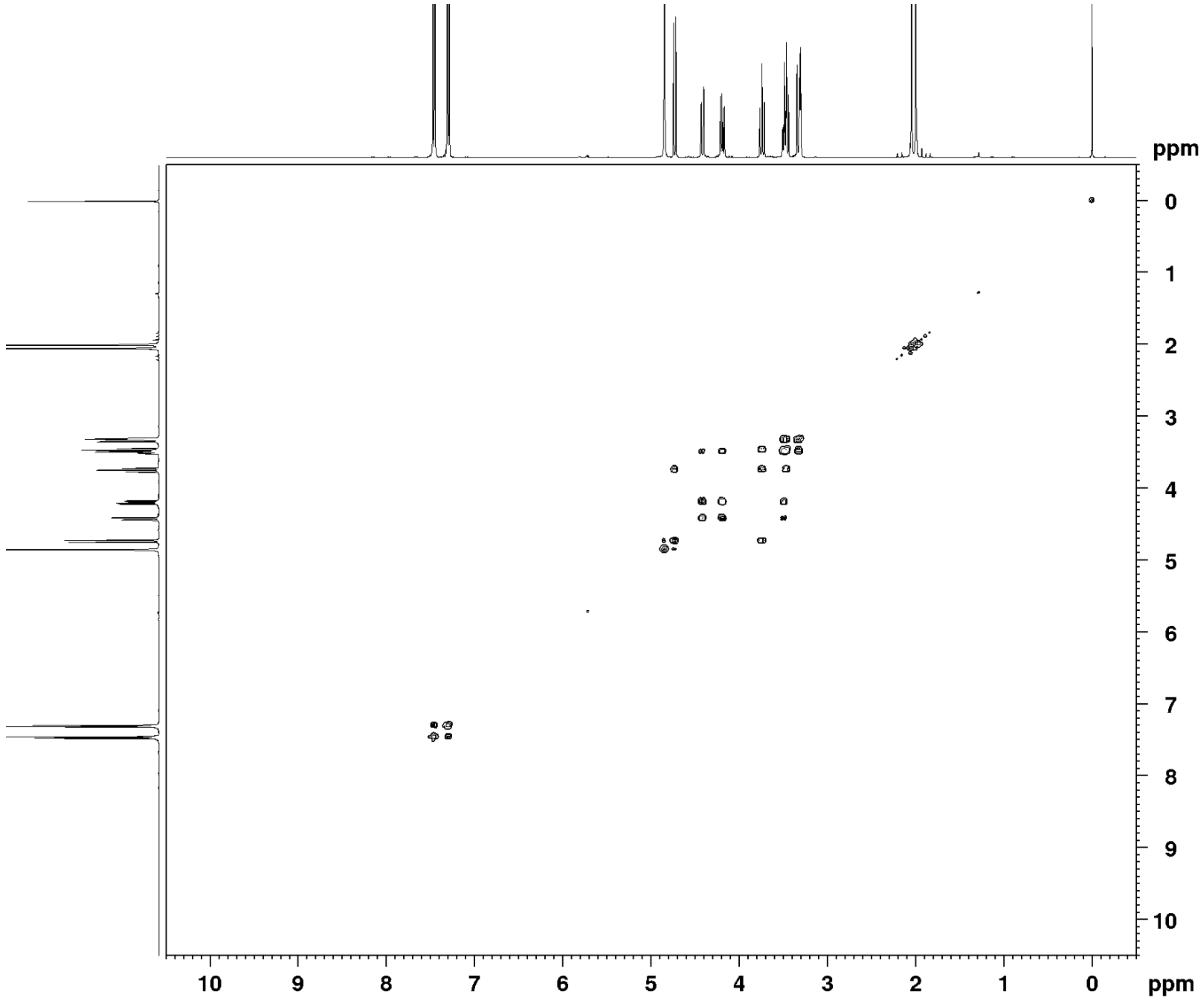

Figure S43: 4-chlorophenyl 2-acetomido-6-*O*-acetyl-2-deoxy-1-thio-β-D-glucopyranoside (**13**) ^{1}H-$^{13}C\{^{1}H\}$ HSQC NMR (400 & 101 MHz) in MeOD

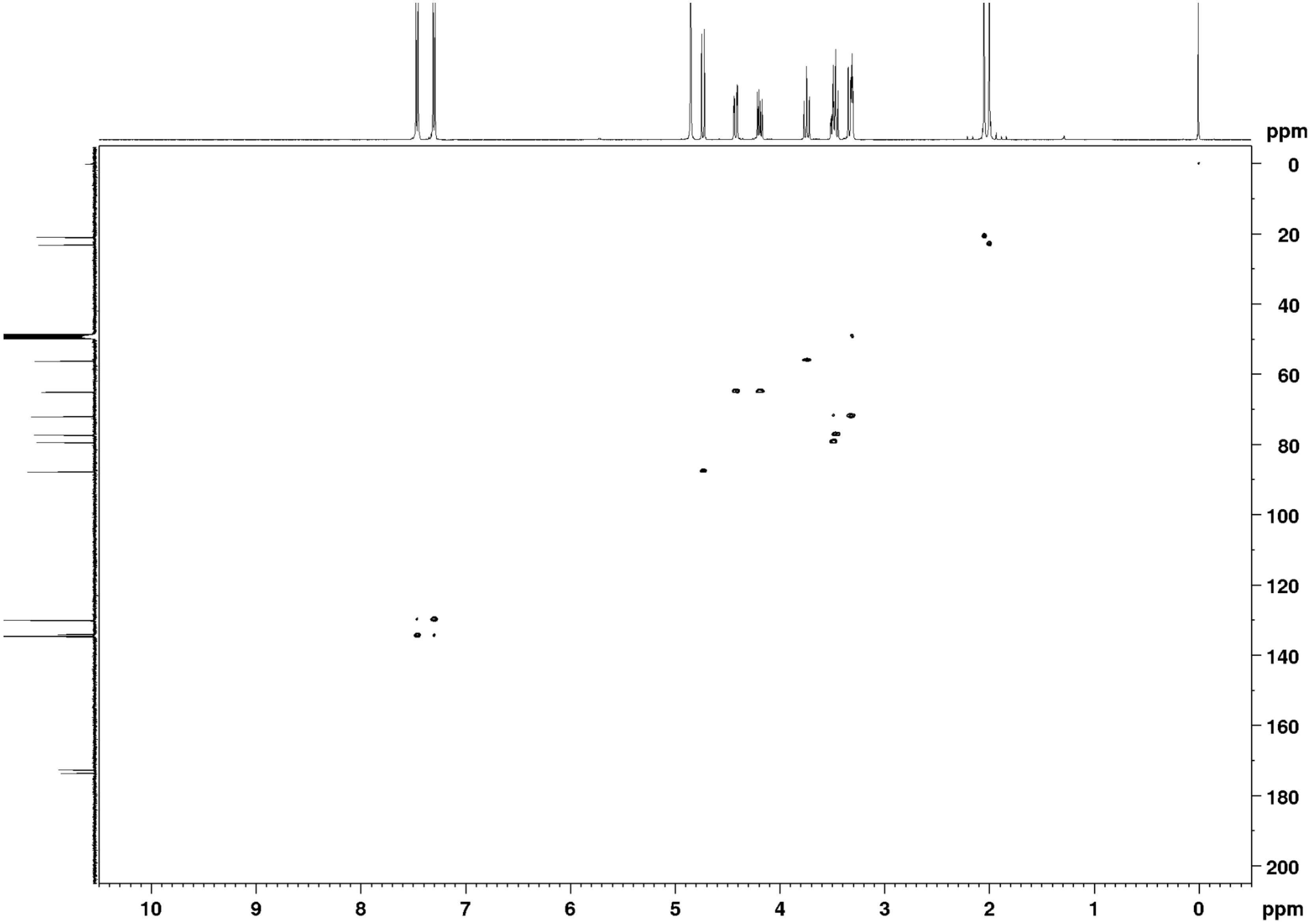

Figure S44: 4-chlorophenyl 2-acetomido-6-*O*-acetyl-2-deoxy-1-thio-β-D-glucopyranoside (**13**) ^{1}H-$^{13}C\{^{1}H\}$ HMBC NMR (400 & 101 MHz) in MeOD

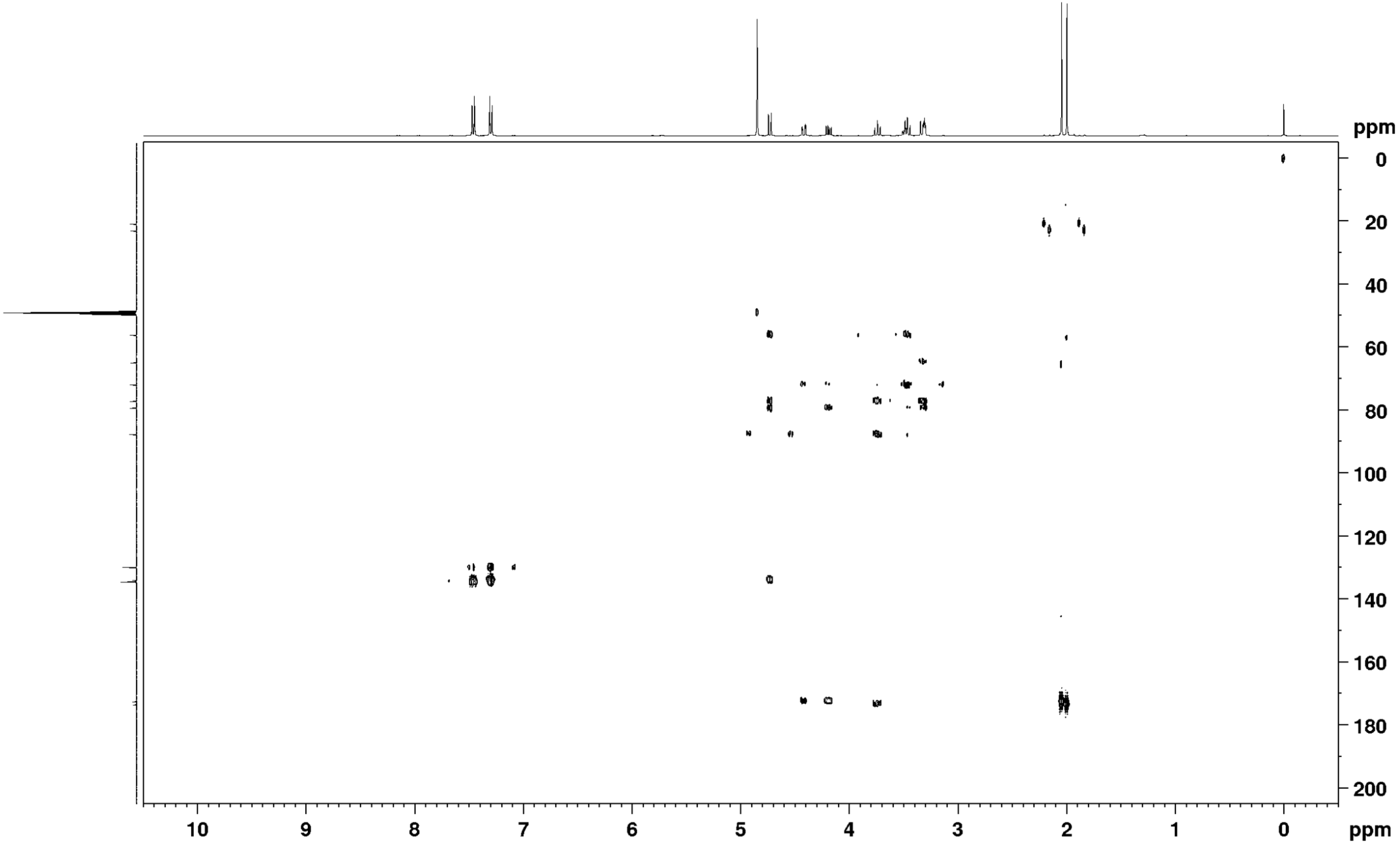

Figure S45: 4-chlorophenyl 2-acetomido-6-*O*-acetyl-2-deoxy-1-thio-β-D-glucopyranoside (13) $^{13}C\{^{1}H\}$ NMR (101 MHz) in MeOD

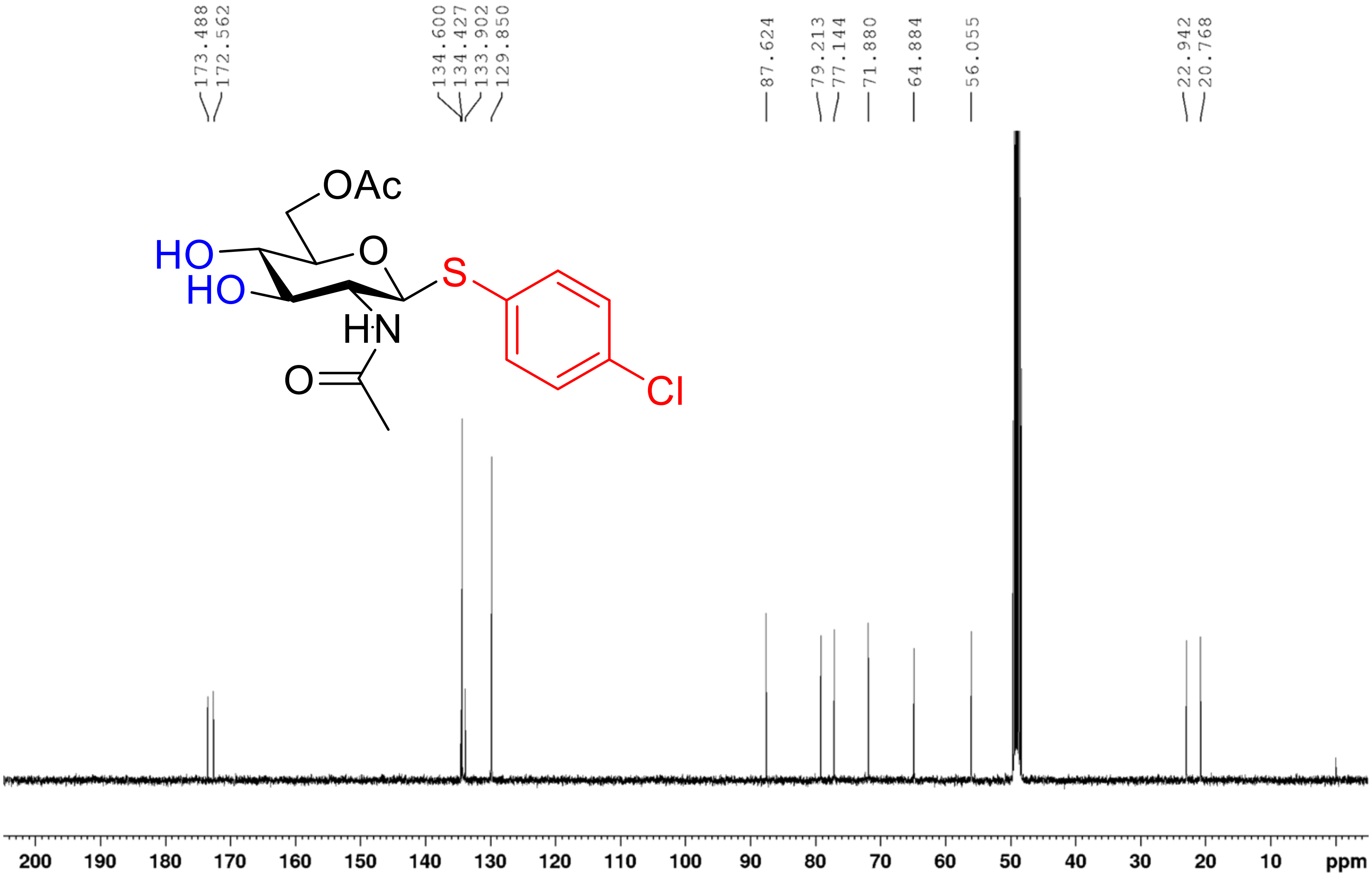

Figure S46: 4-chlorophenyl 6-acetyl-2-deoxy-2-phthalimido-1-thio-β-D-glucopyranoside (**14**) ^{1}H NMR (400 MHz) in DMSO-d_6

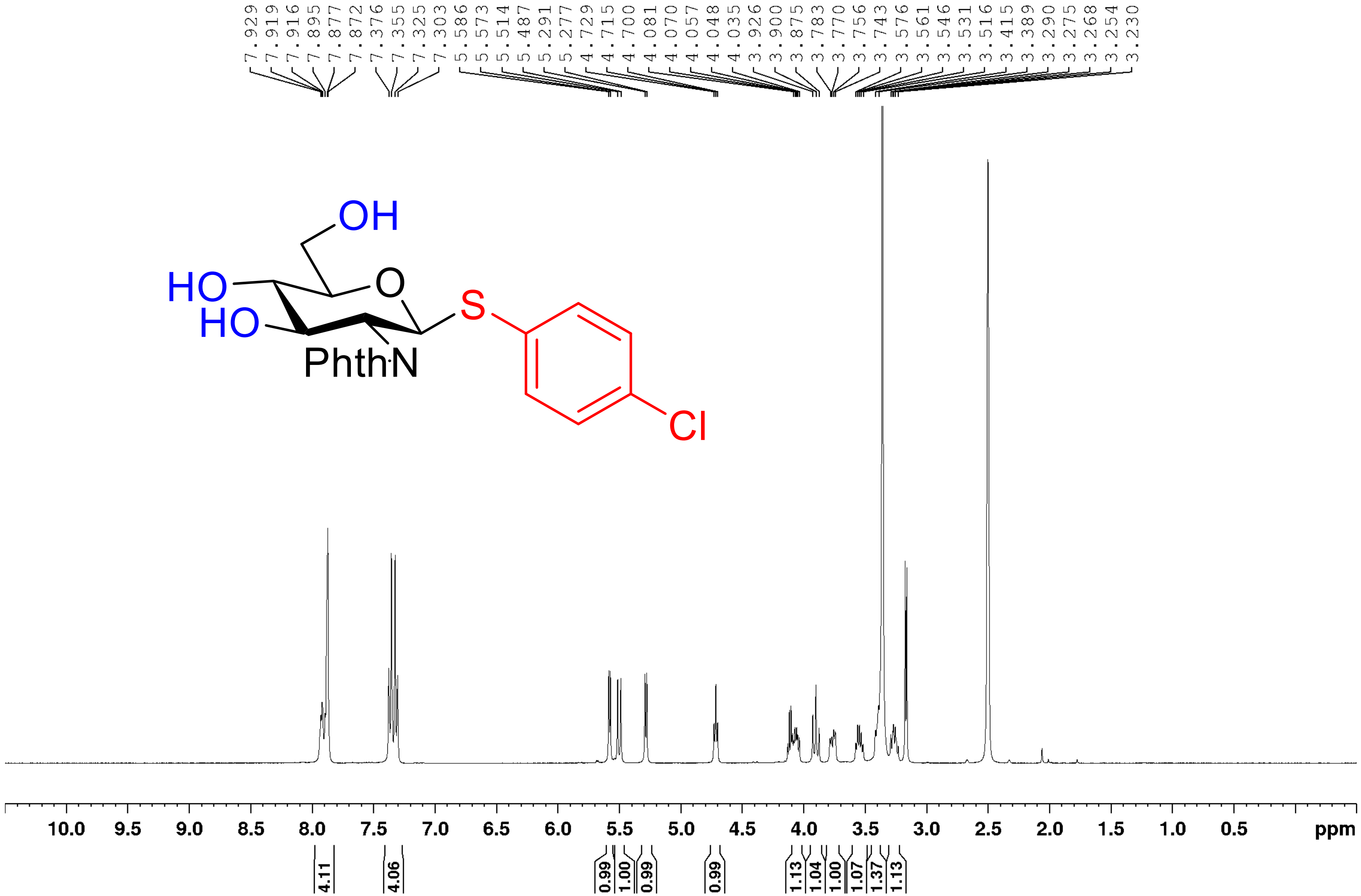

Figure S47: 4-chlorophenyl 6-acetyl-2-deoxy-2-phthalimido-1-thio-β-D-glucopyranoside (15) ^{1}H NMR (400 MHz) in MeOD:$CHCl_3$ 7:3

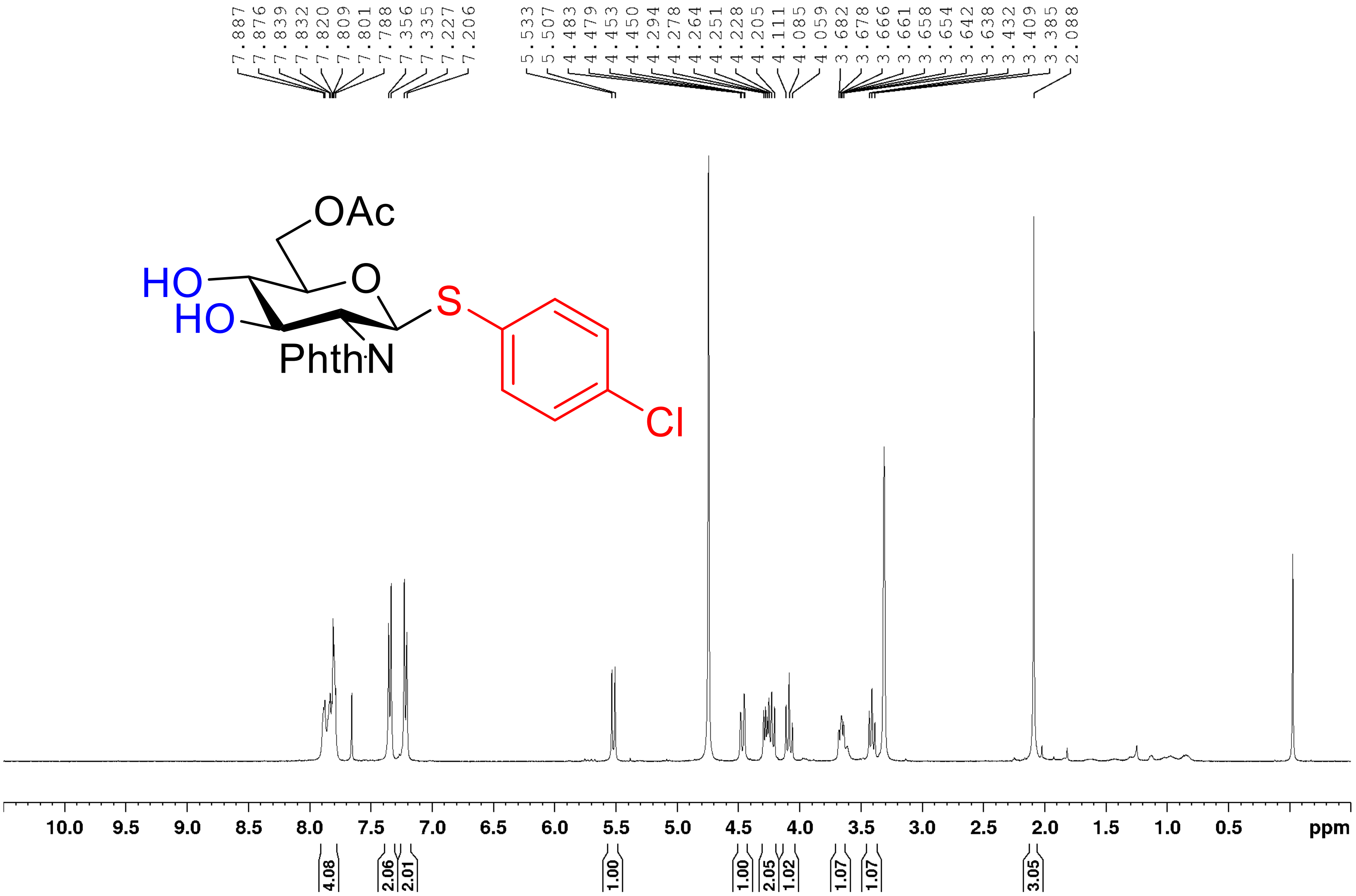

Figure S48: 4-chlorophenyl 6-acetyl-2-deoxy-2-phthalimido-1-thio-β-D-glucopyranoside (15) ^1H-^1H COSY NMR (400 MHz) in MeOD:$CHCl_3$ 7:3

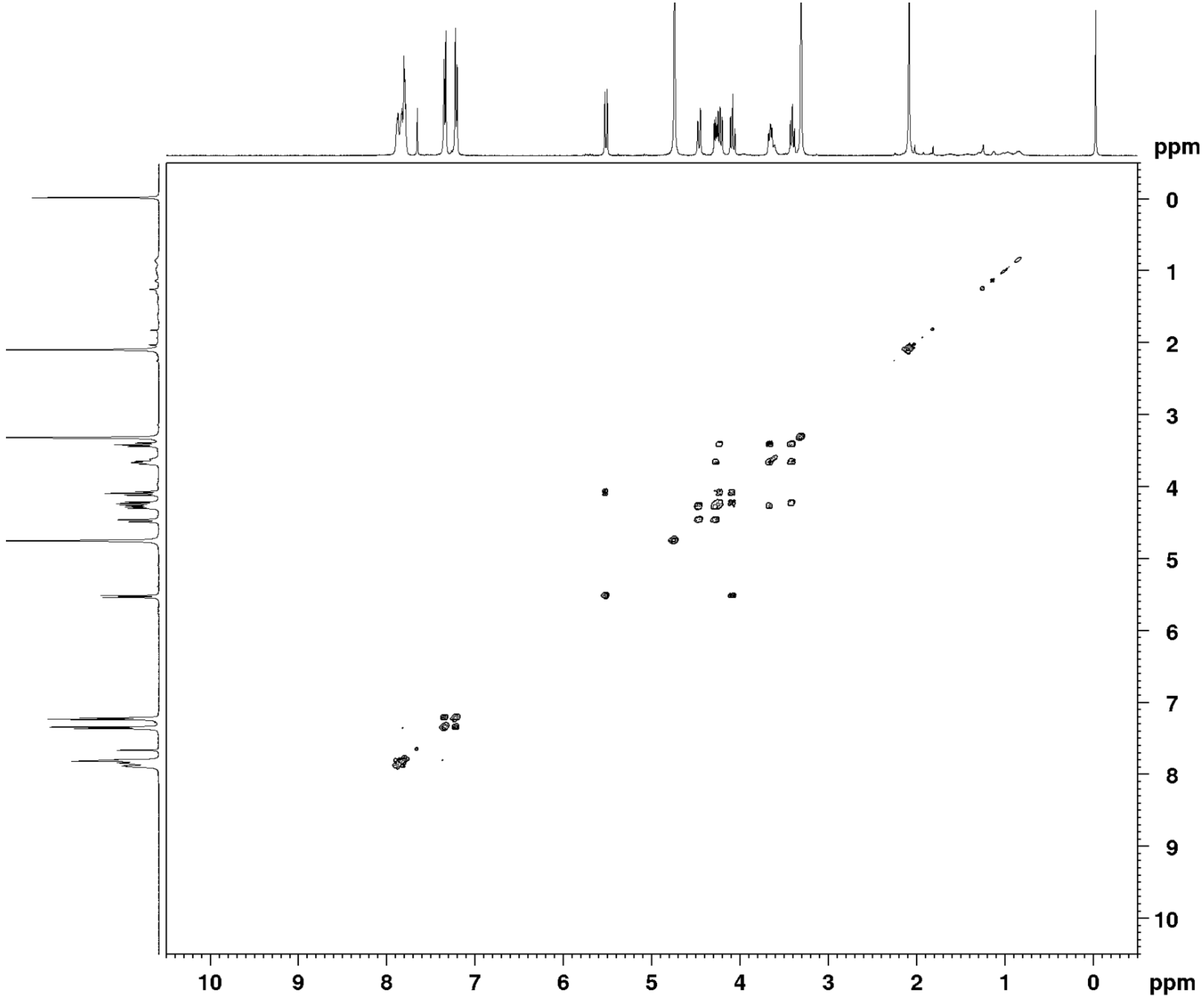

Figure S49: 4-chlorophenyl 6-acetyl-2-deoxy-2-phthalimido-1-thio-β-D-glucopyranoside (**15**) ^{1}H-$^{13}C\{^{1}H\}$ HSQC NMR (400 & 101 MHz) in MeOD:$CHCl_3$ 7:3

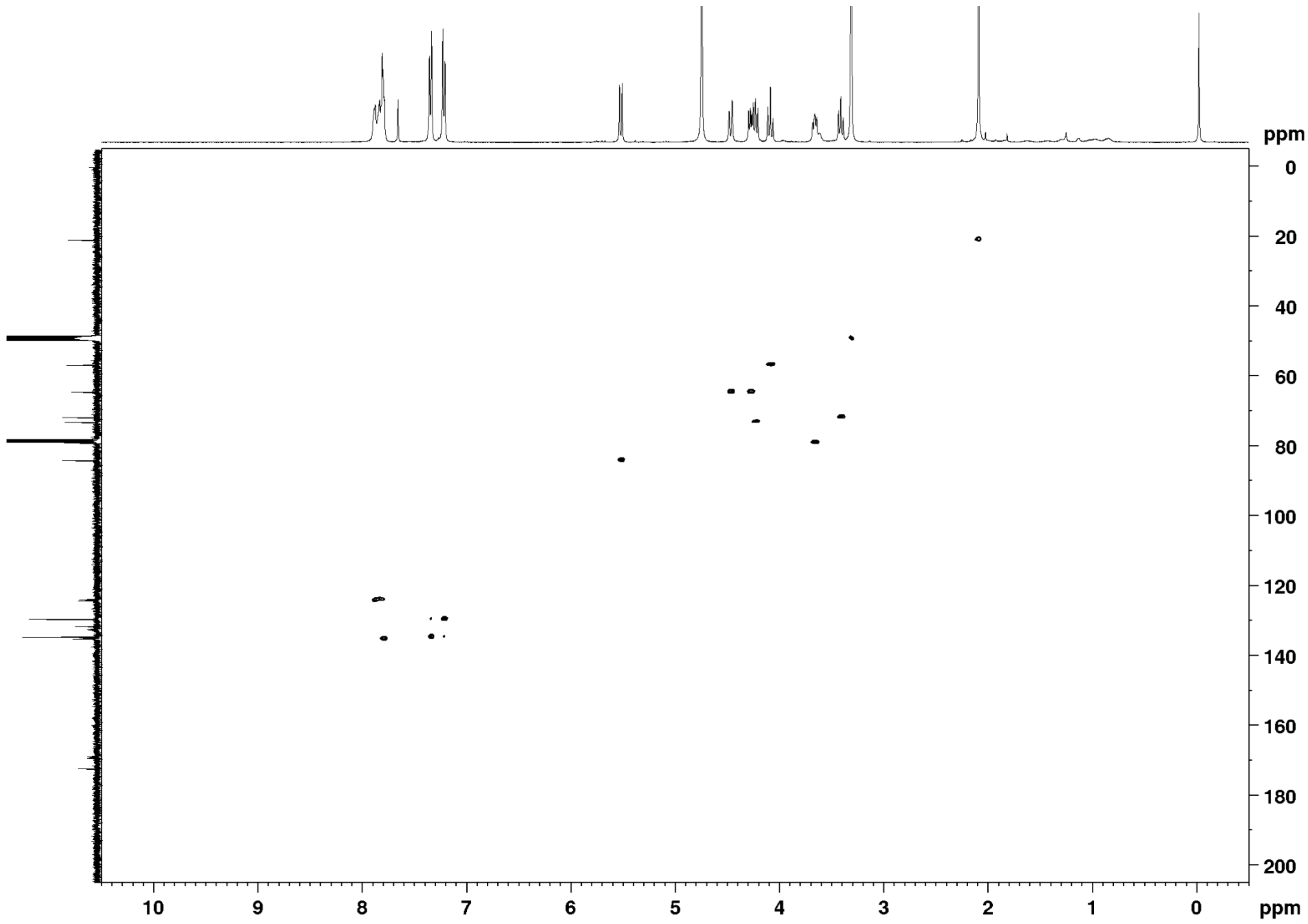

Figure S50: 4-chlorophenyl 6-acetyl-2-deoxy-2-phthalimido-1-thio-β-D-glucopyranoside (15) ^{1}H-$^{13}C\{^{1}H\}$ HMBC NMR (400 & 101 MHz) in MeOD:$CHCl_3$ 7:3

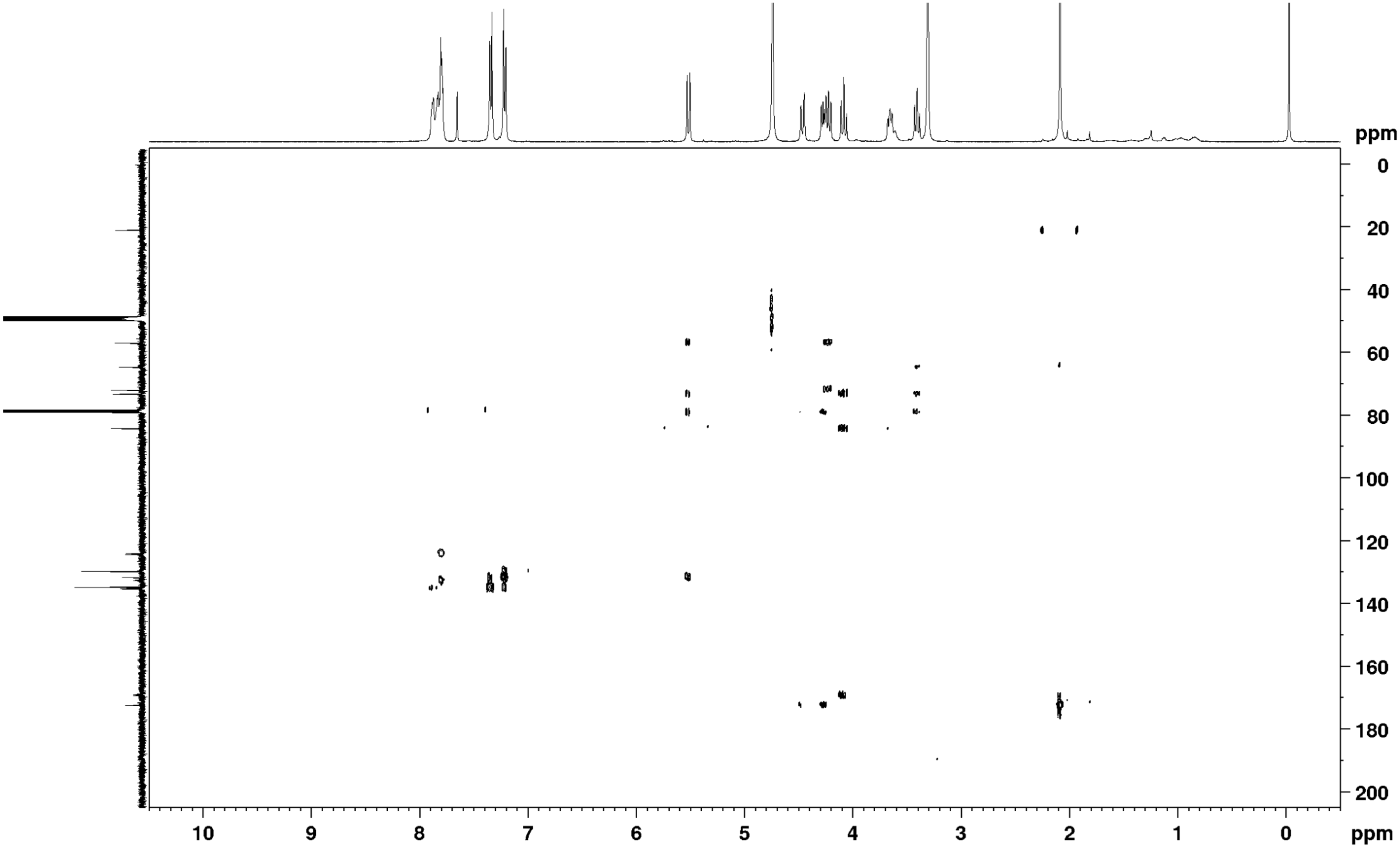

Figure S51: 4-chlorophenyl 6-acetyl-2-deoxy-2-phthalimido-1-thio-β-D-glucopyranoside (15) $^{13}C\{^1H\}$ NMR (101 MHz) in MeOD:$CHCl_3$ 7:3

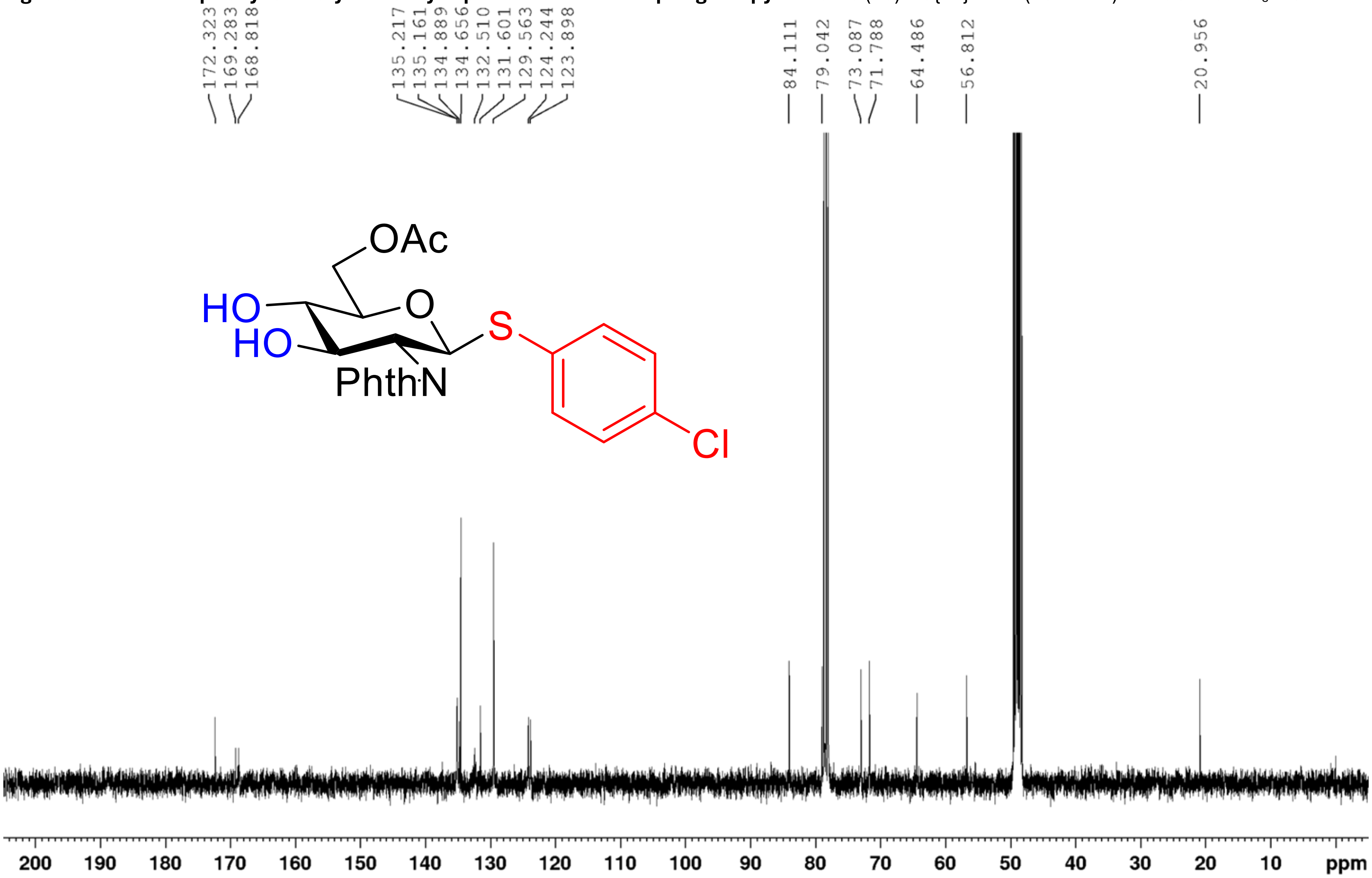

Figure S52: 4-chlorophenyl 1-thio-D-mannopyranoside (16) α:β 10:90 ^{1}H NMR (400 MHz) in MeOD

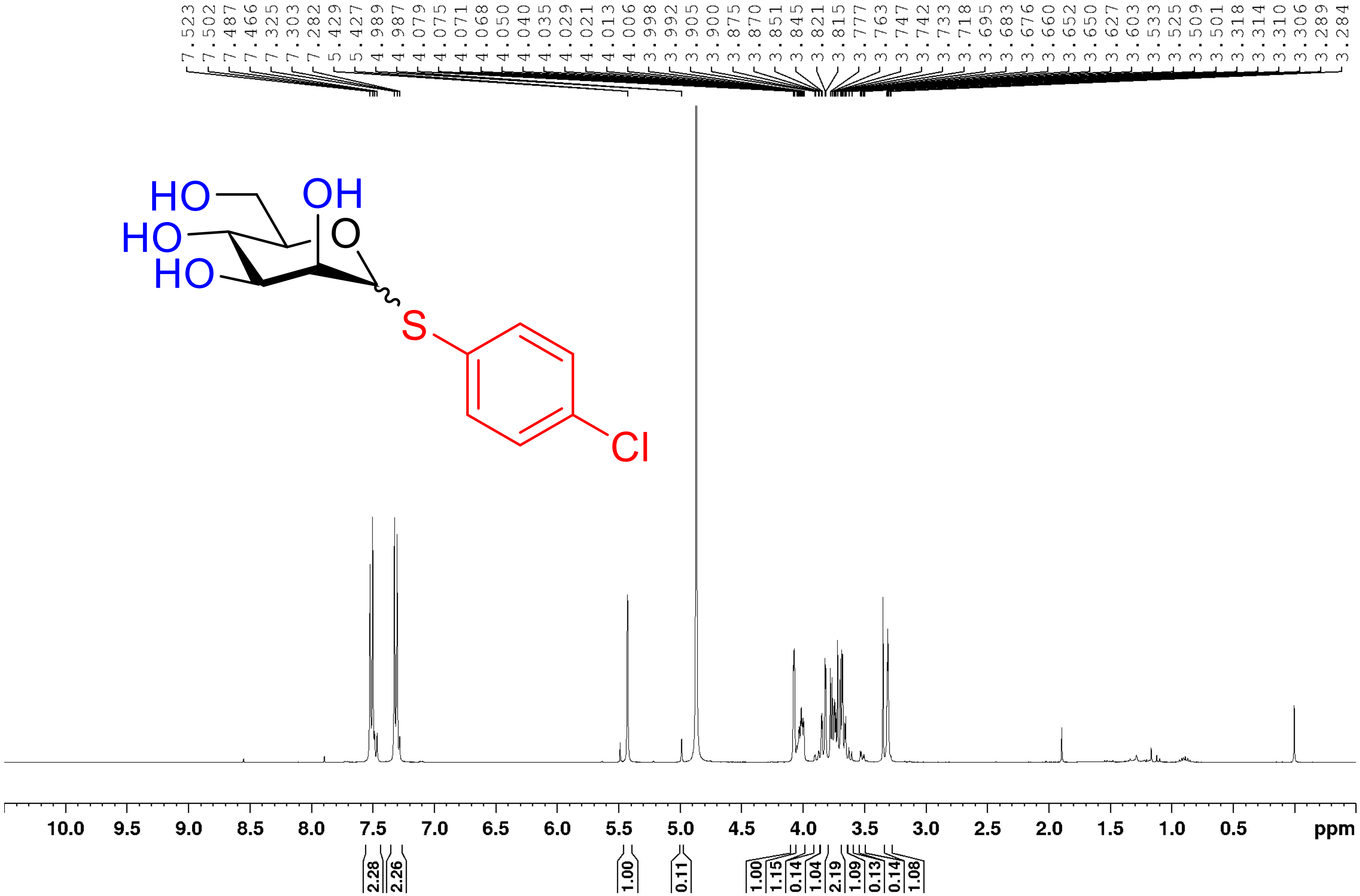

Figure S53: 4-chlorophenyl 1-thio-D-glucopyranoside (**16**) α:β 10:90 ^{1}H-^{1}H COSY NMR (400 MHz) in MeOD

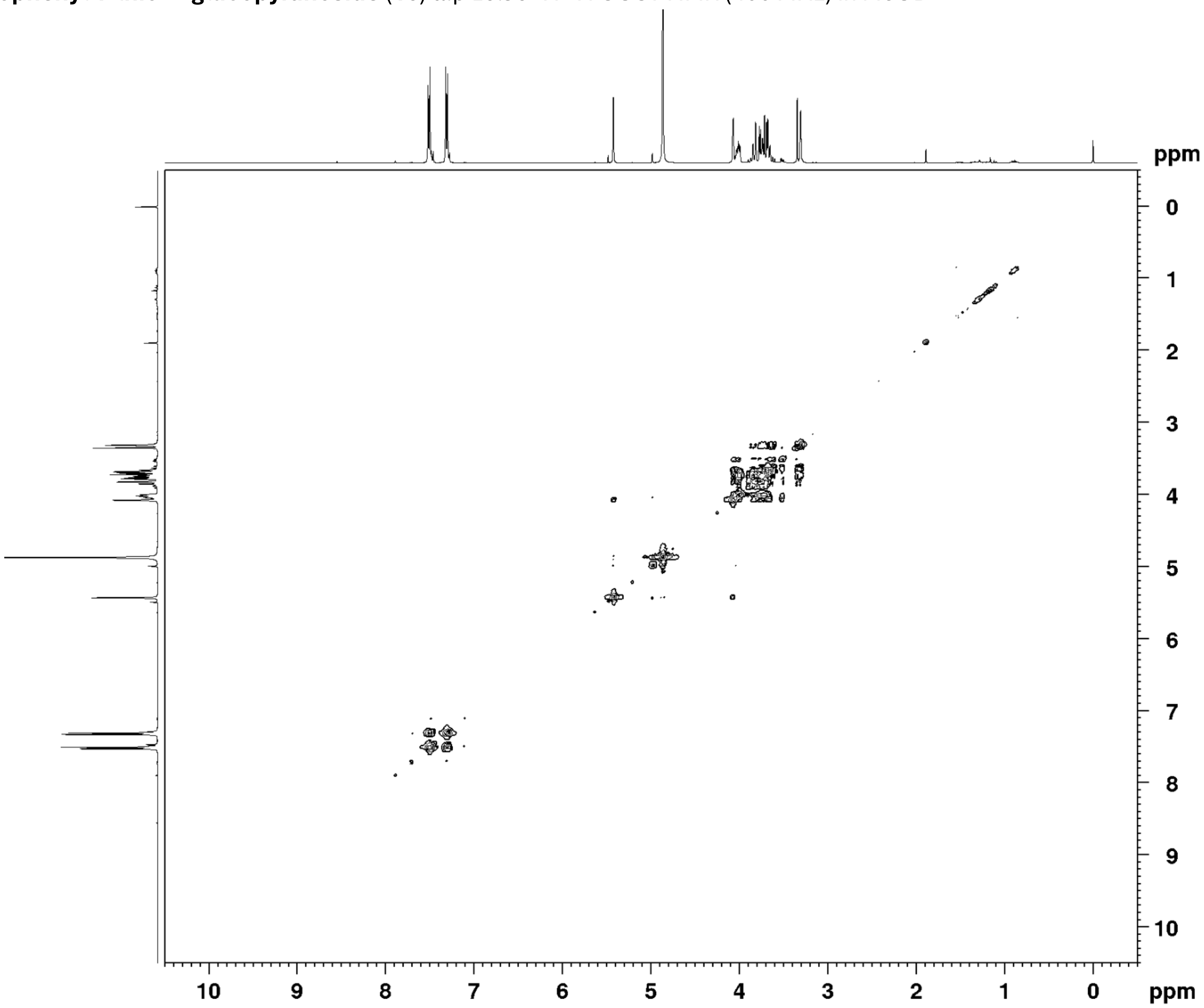

Figure S54: 4-chlorophenyl 1-thio-D-glucopyranoside (16) α:β 10:90 ^{1}H-$^{13}C\{^{1}H\}$ HSQC NMR (400 & 101 MHz) in MeOD

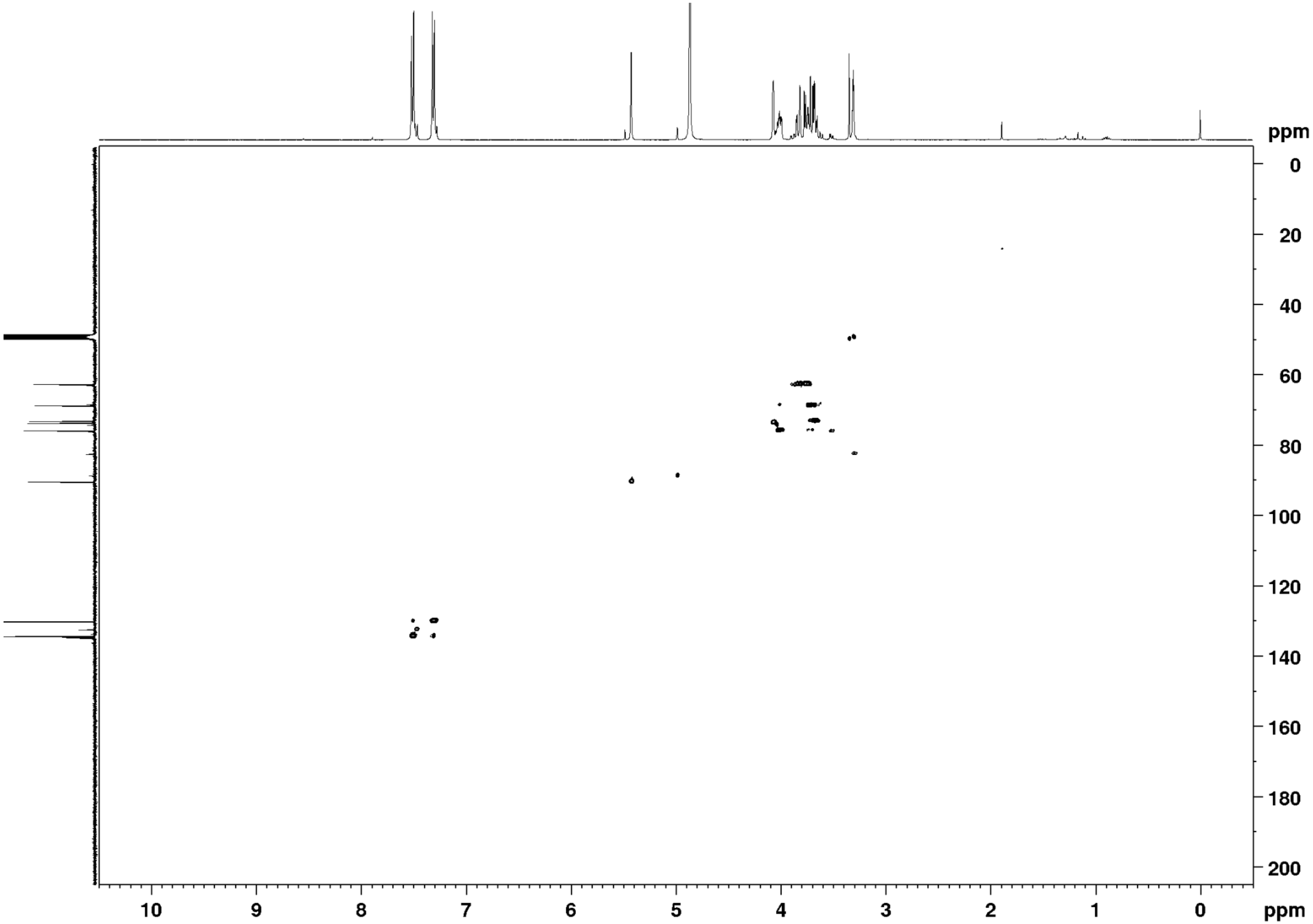

Figure S55: 4-chlorophenyl 1-thio-D-glucopyranoside (16) α:β 10:90 ^{1}H-$^{13}C\{^{1}H\}$ HMBC NMR (400 & 101 MHz) in MeOD

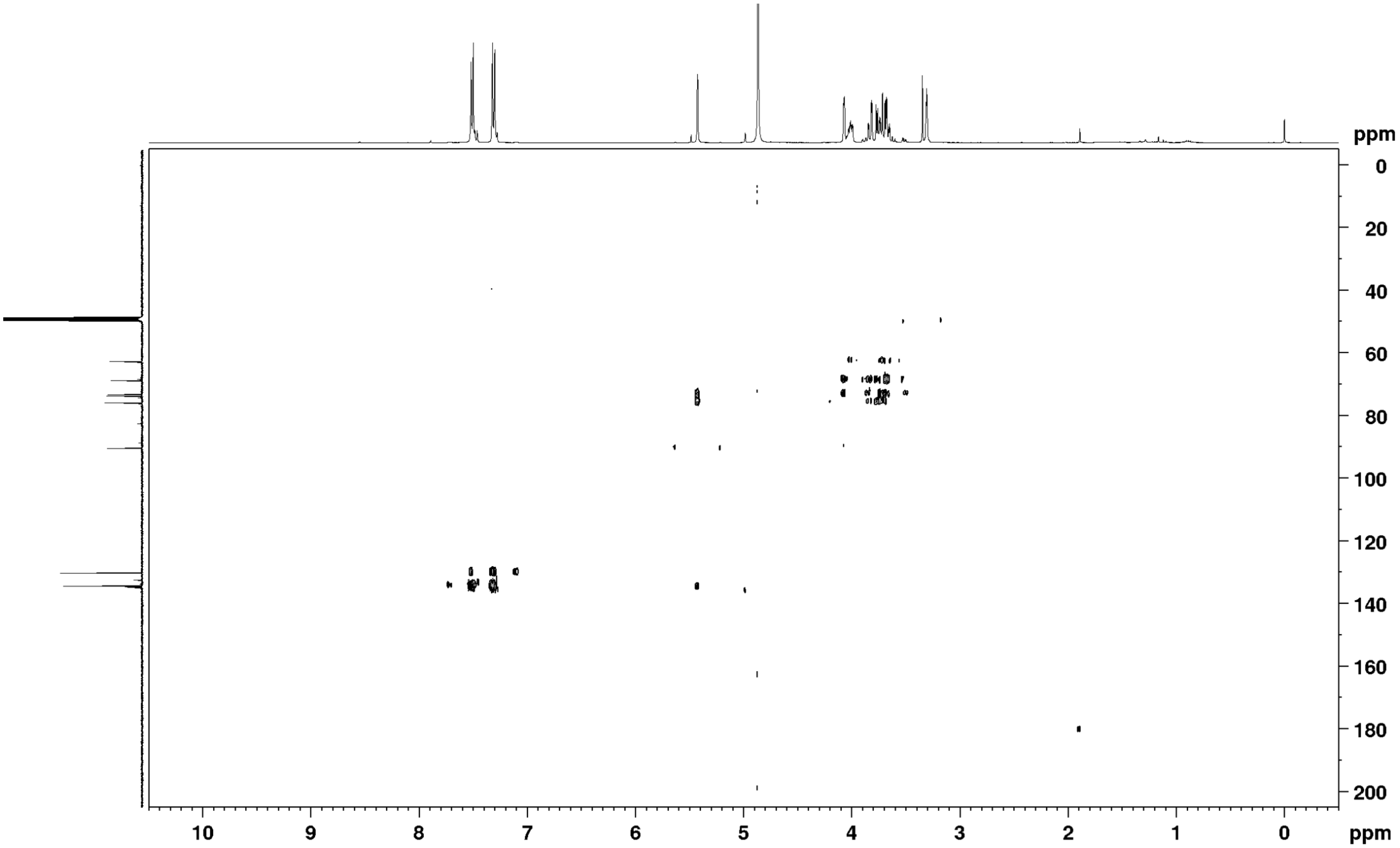

Figure S56: 4-chlorophenyl 1-thio-D-glucopyranoside (16) α:β 10:90 $^{13}C\{^{1}H\}$ NMR (101 MHz) in MeOD

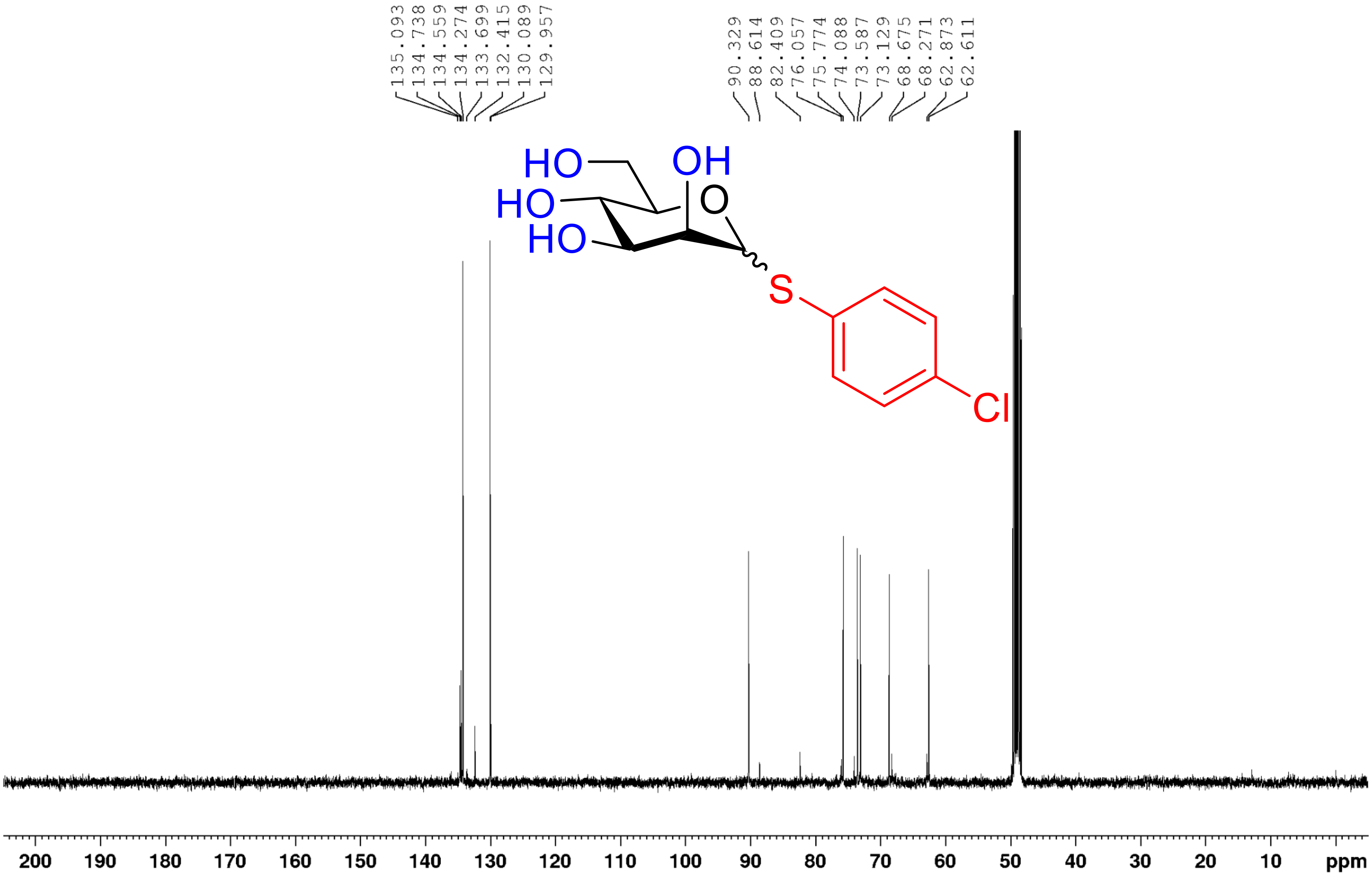

Figure S57: 4-chlorophenyl 6-*O*-acetyl-1-thio-β-D-mannopyranoside (17) ¹H NMR (400 MHz) in MeOD

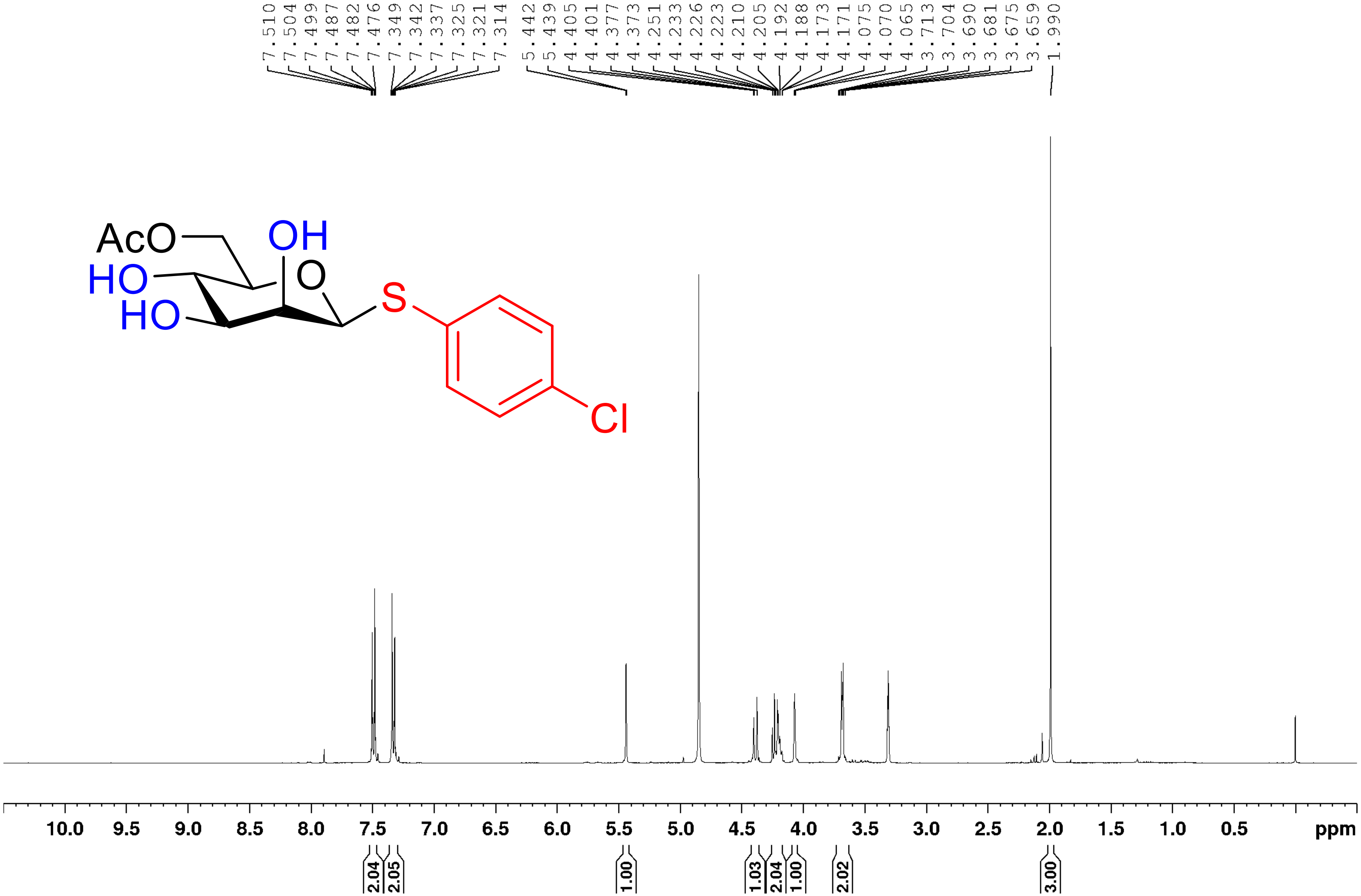

Figure S58: 4-chlorophenyl 6-*O*-acetyl-1-thio-β-D-glucopyranoside (17) ^{1}H-^{1}H COSY NMR (400 MHz) in MeOD

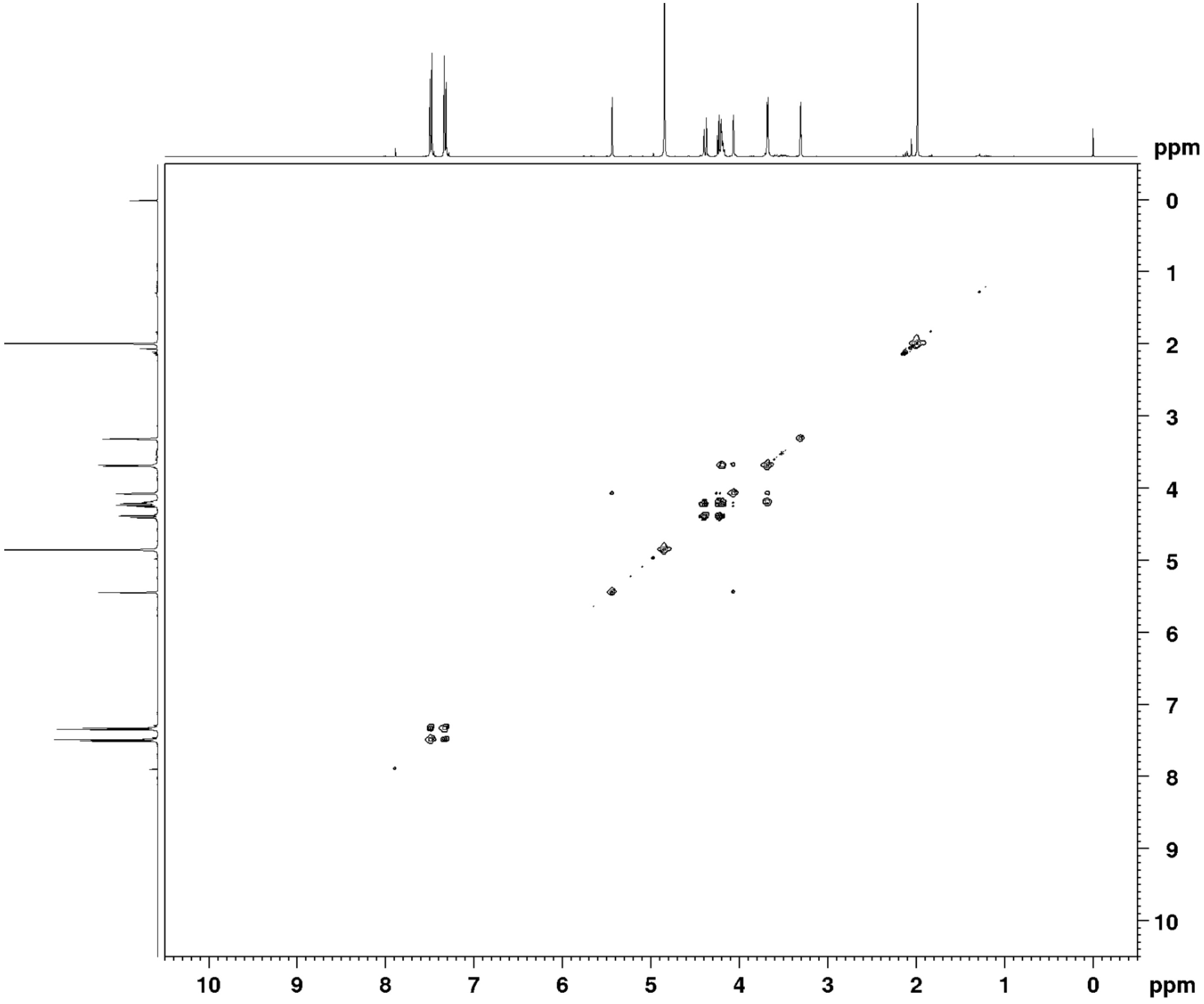

Figure S59: 4-chlorophenyl 6-*O*-acetyl-1-thio-β-D-glucopyranoside (17) ^{1}H-$^{13}C\{^{1}H\}$ HSQC NMR (400 & 101 MHz) in MeOD

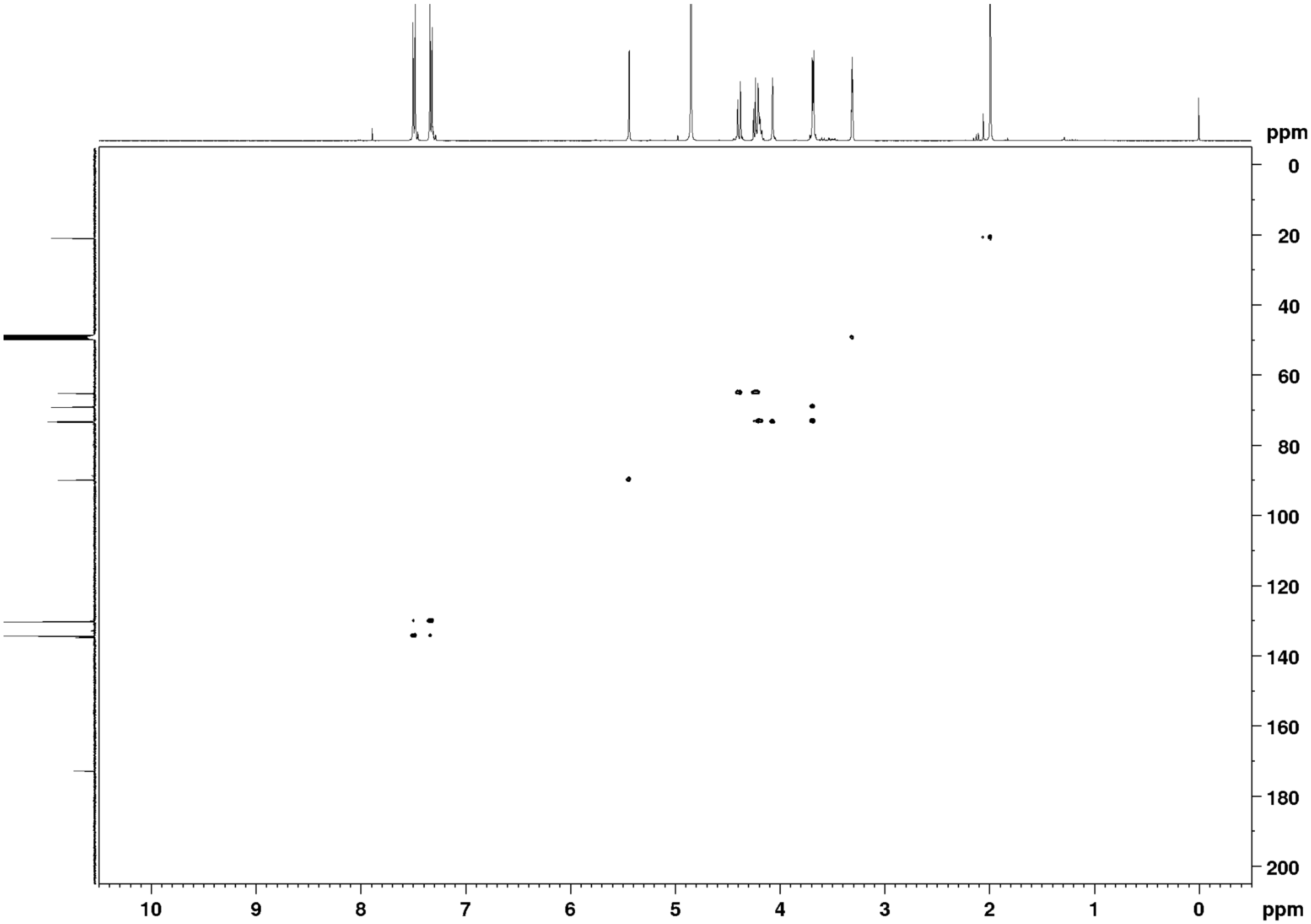

Figure S60: 4-chlorophenyl 6-*O*-acetyl-1-thio-β-D-glucopyranoside (**17**) ^{1}H-^{13}C{^{1}H} HMBC NMR (400 & 101 MHz) in MeOD

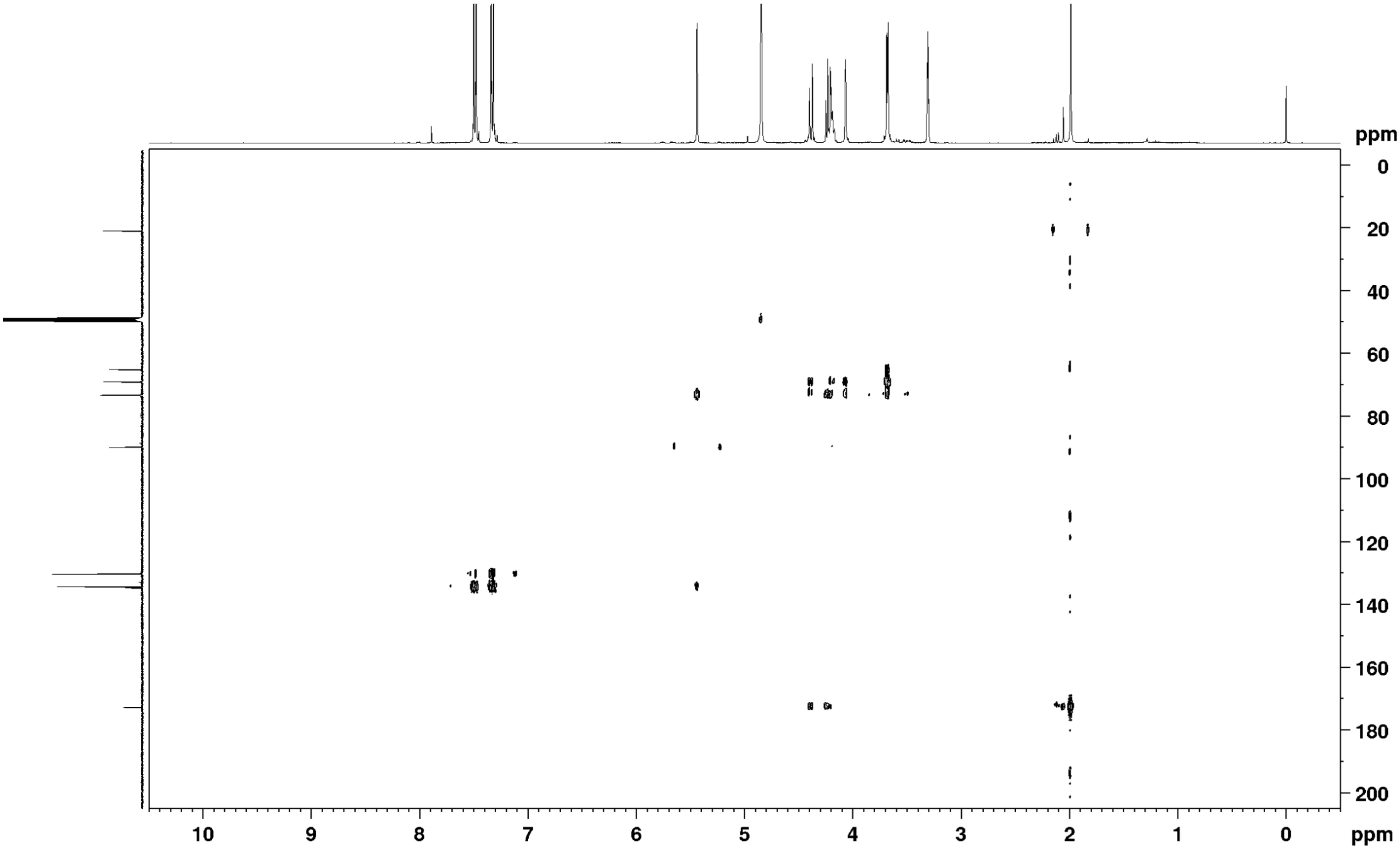

Figure S61: 4-chlorophenyl 6-*O*-acetyl-1-thio-β-D-glucopyranoside (17) $^{13}C\{^{1}H\}$ NMR (101 MHz) in MeOD

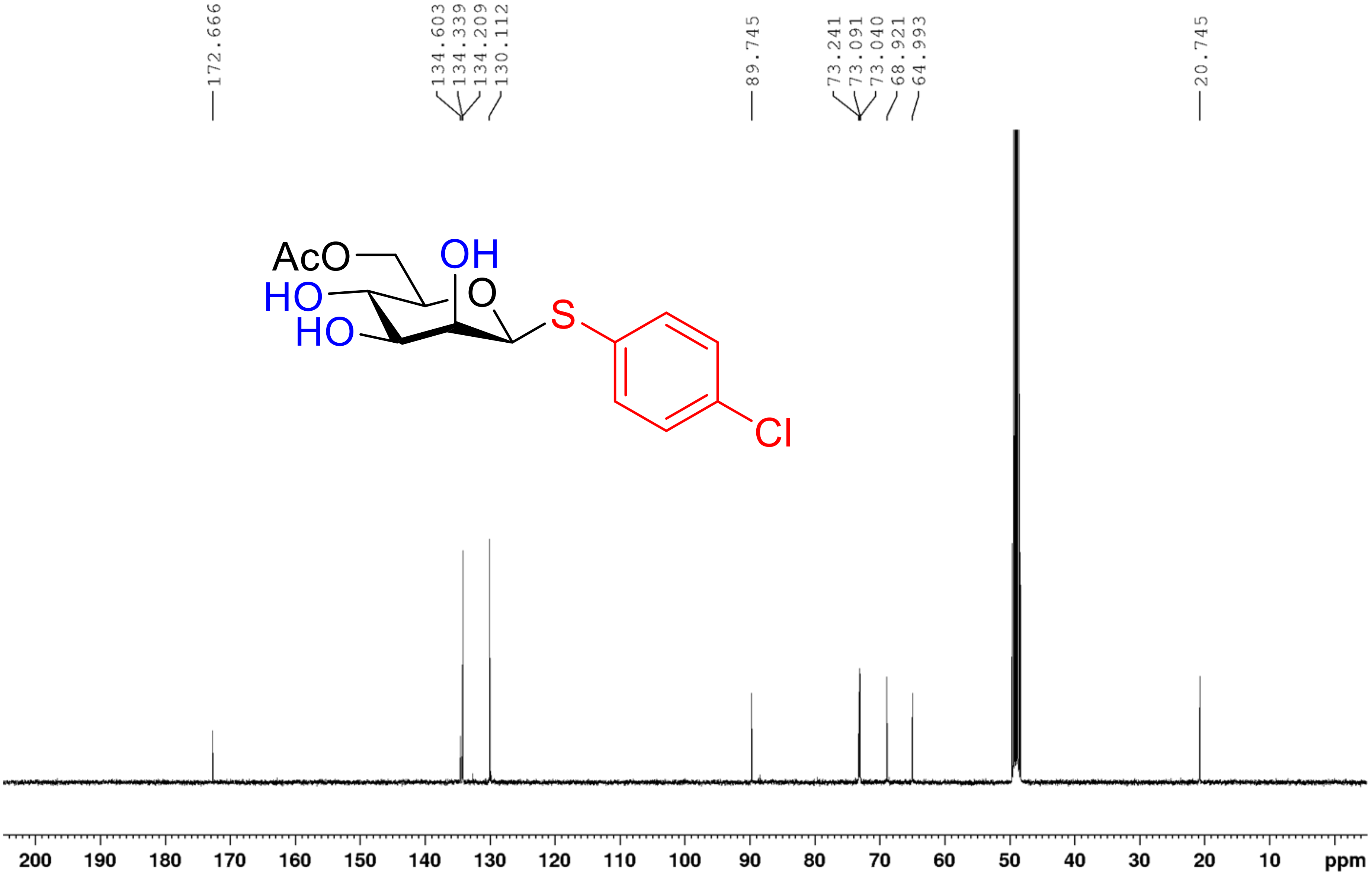

Figure S62: 4-chlorophenyl 6-deoxy-1-thio-α-L-mannopyranoside (18) ^{1}H NMR (400 MHz) in MeOD

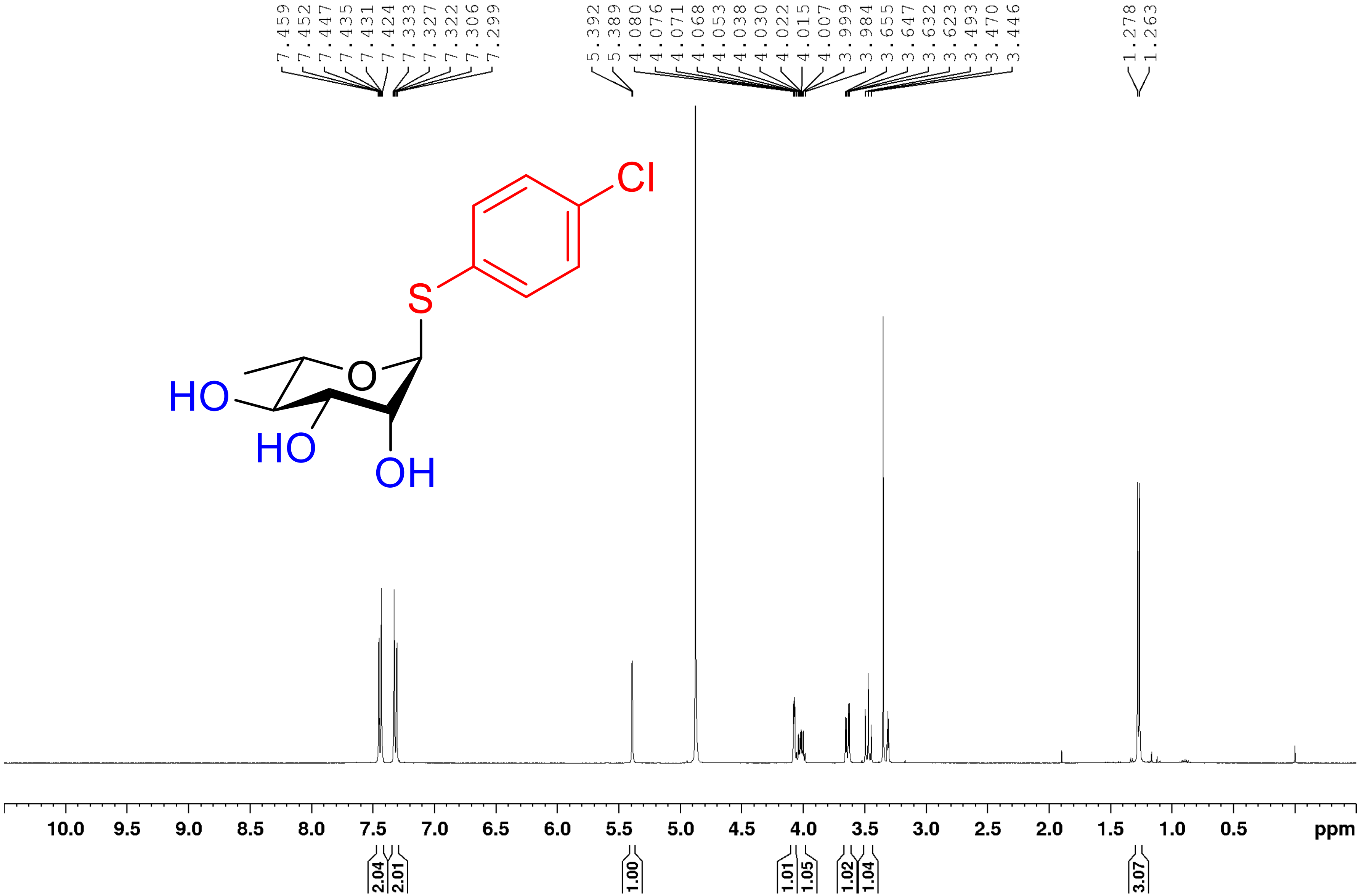

Figure S63: 4-chlorophenyl 6-deoxy-1-thio-α-L-mannopyranoside (18) ^{1}H-^{1}H COSY NMR (400 MHz) in MeOD

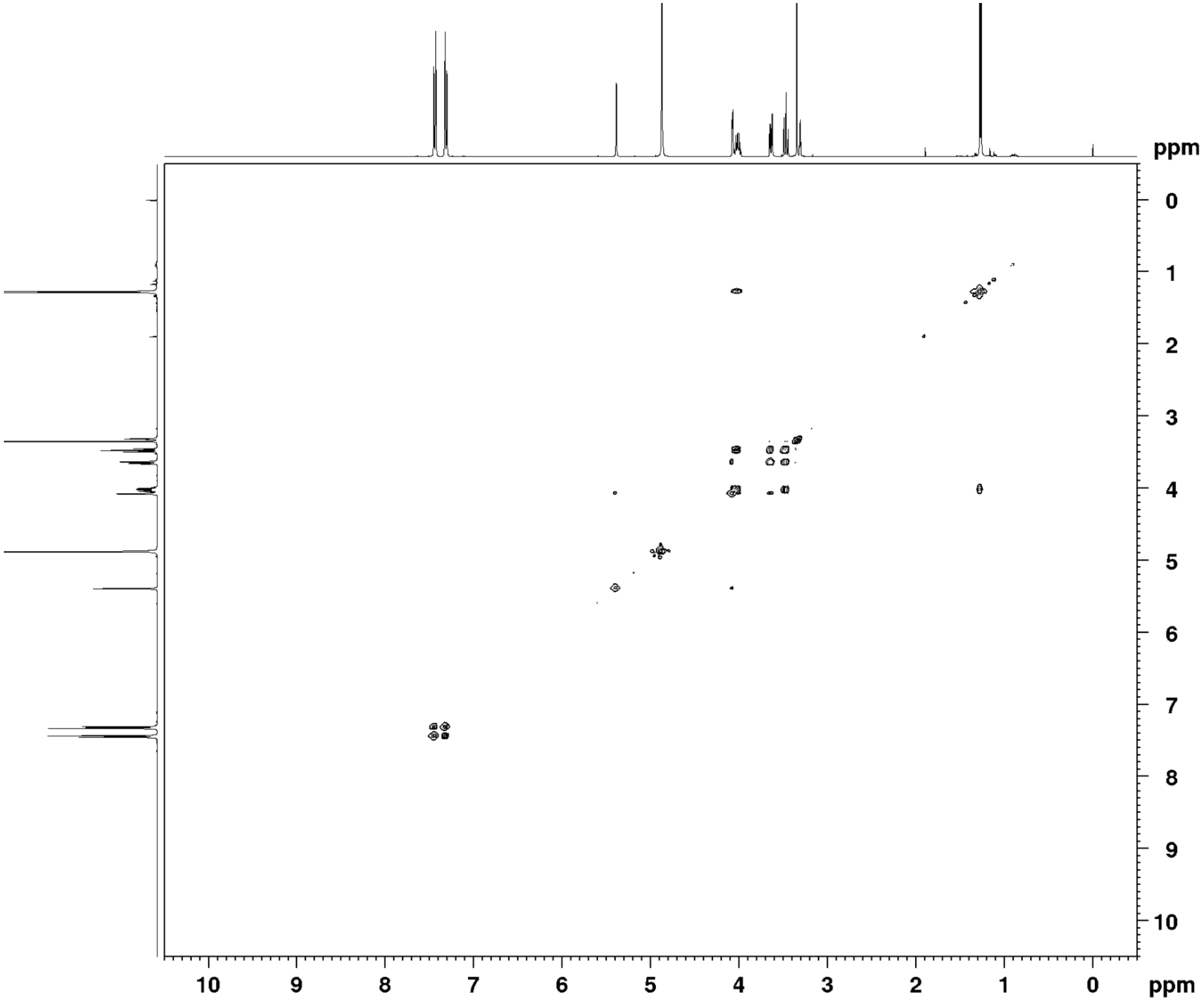

Figure S64: 4-chlorophenyl 6-deoxy-1-thio-α-L-mannopyranoside (**18**) ^{1}H-$^{13}C\{^{1}H\}$ HSQC NMR (400 & 101 MHz) in MeOD

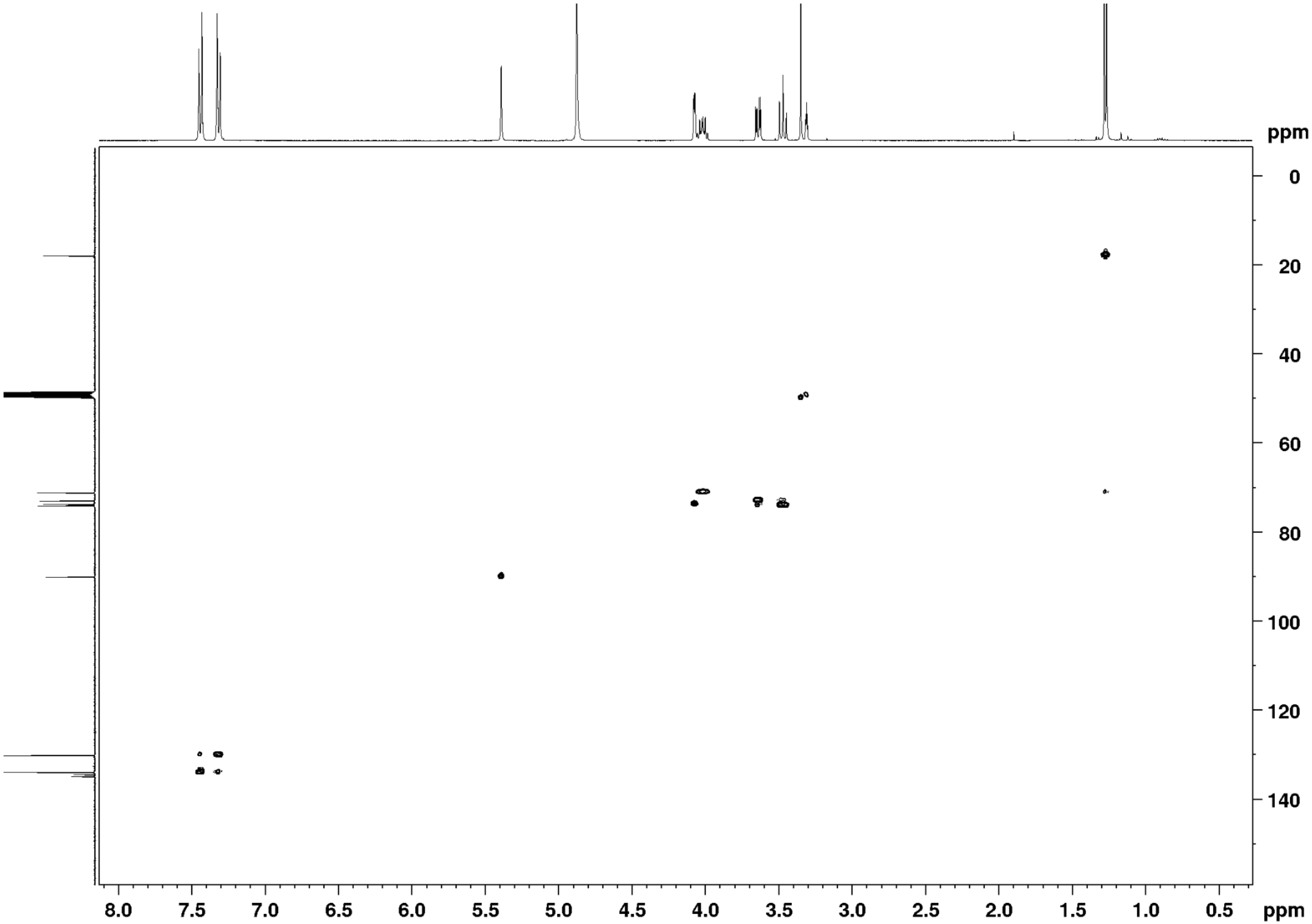

Figure S65: 4-chlorophenyl 6-deoxy-1-thio-α-L-mannopyranoside (**18**) ^{1}H-^{13}C{^{1}H} HMBC NMR (400 & 101 MHz) in MeOD

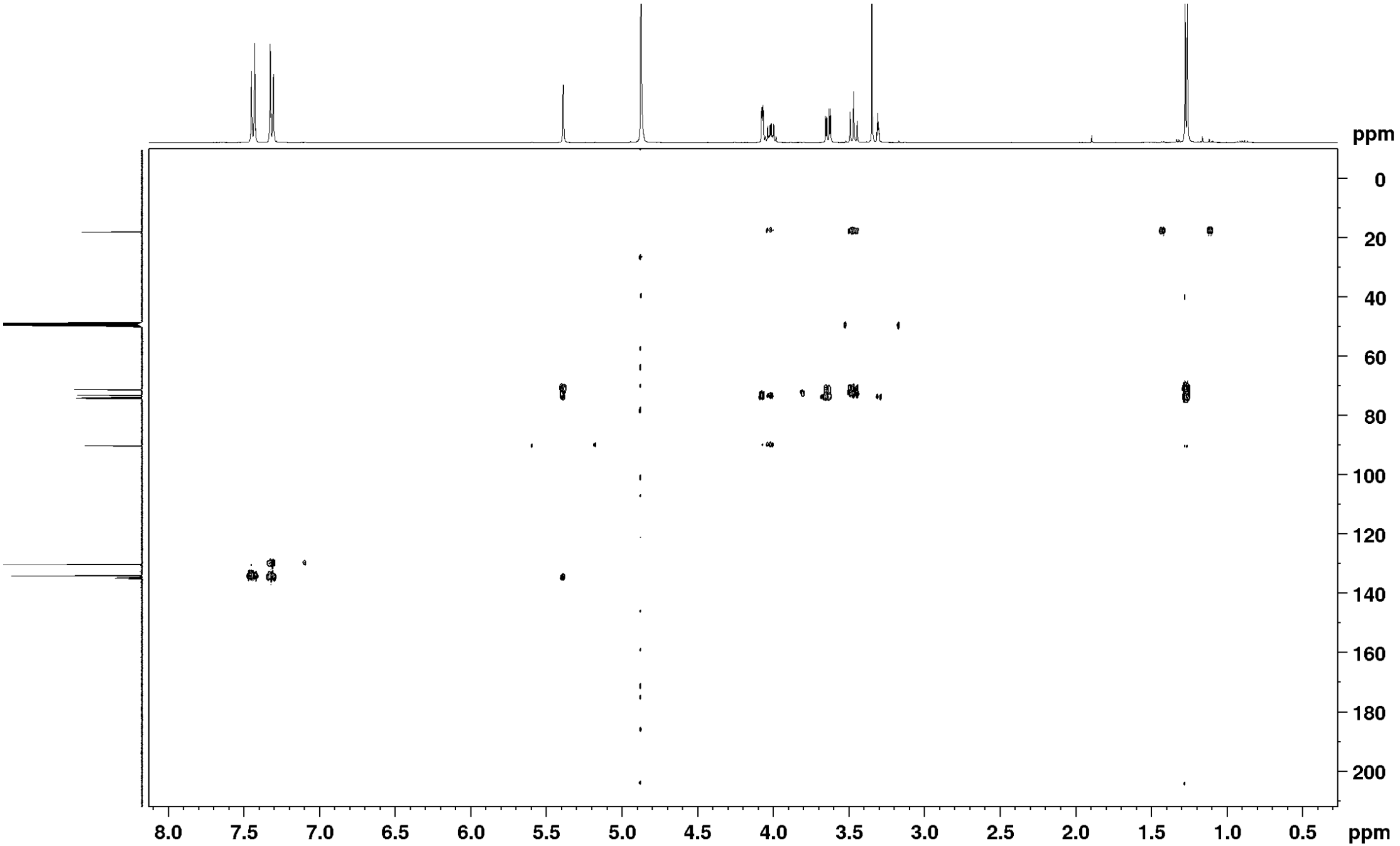

Figure S66: 4-chlorophenyl 6-deoxy-1-thio-α-L-mannopyranoside (18) $^{13}C\{^{1}H\}$ NMR (101 MHz) in MeOD

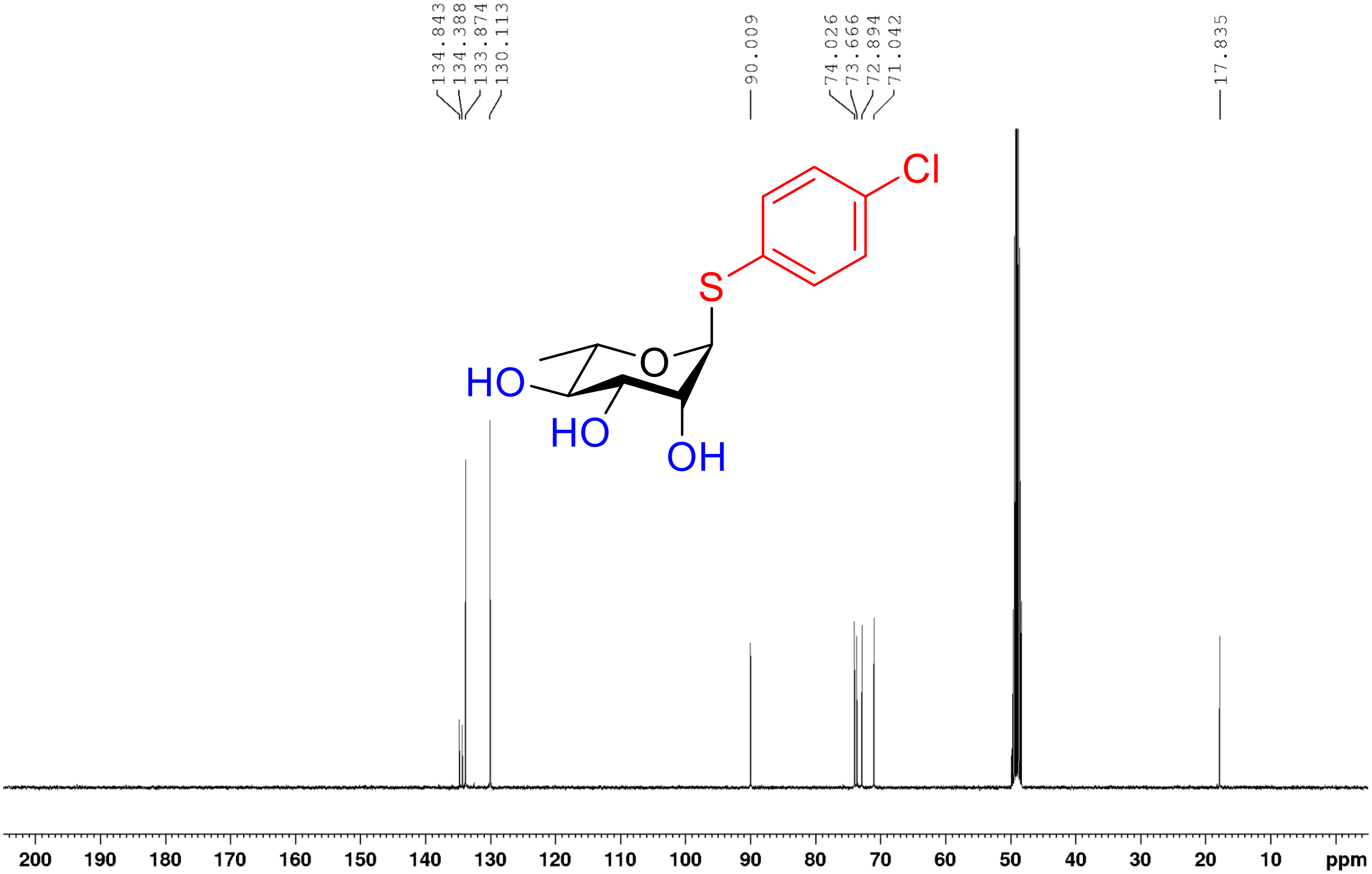

Figure S67: 4-chlorophenyl 2-*O*-acetyl-6-deoxy-1-thio-α-L-mannopyranoside (19) shown from mixture ^{1}H NMR (400 MHz) in MeOD

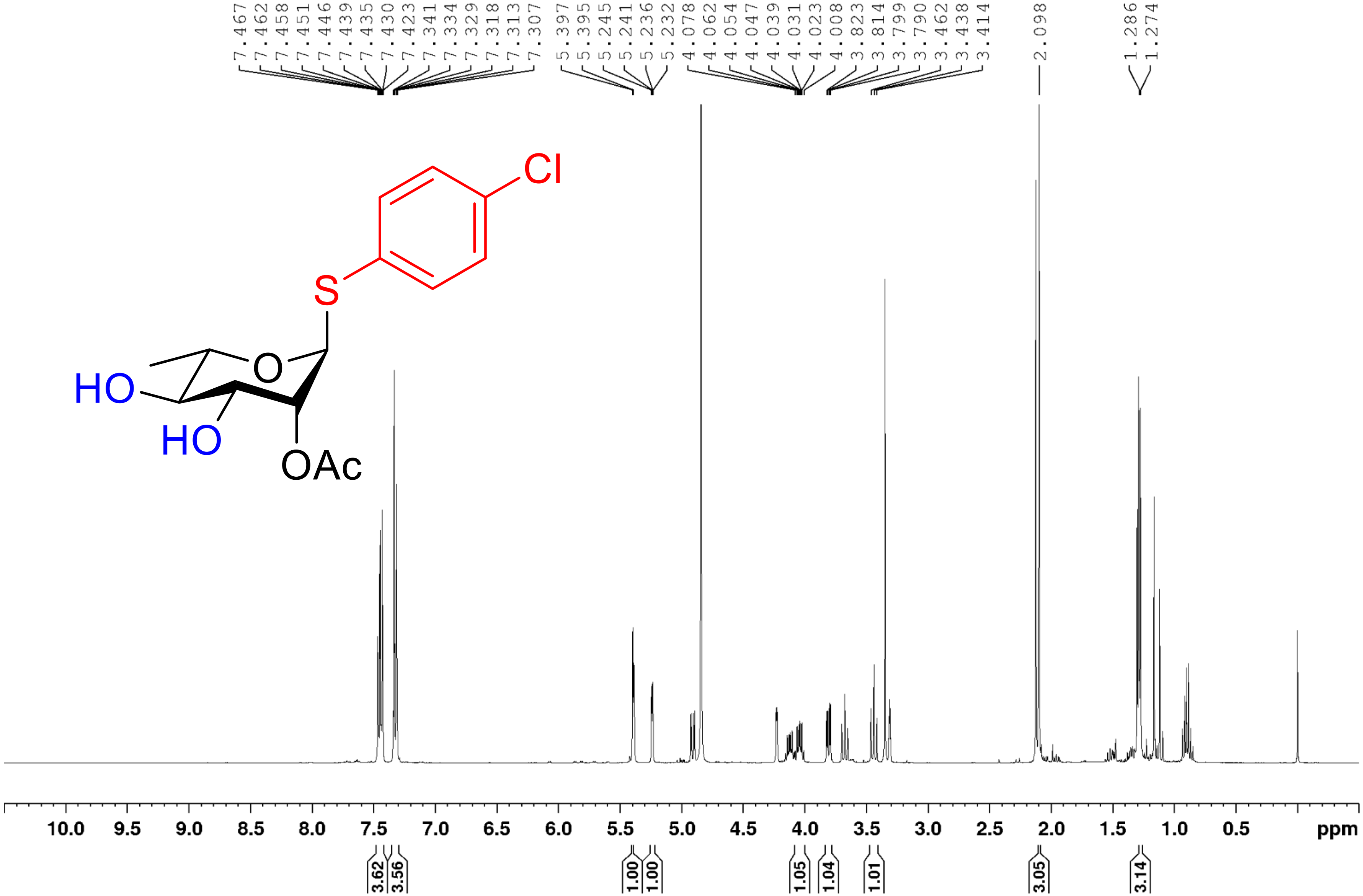

Figure S68: 4-chlorophenyl 2-*O*-acetyl-6-deoxy-1-thio-α-L-mannopyranoside (**19**) and **4-chlorophenyl 2-*O*-acetyl-6-deoxy-1-thio-α-L-mannopyranoside** (**20**) ^{1}H-^{1}H COSY NMR (400 MHz) in MeOD

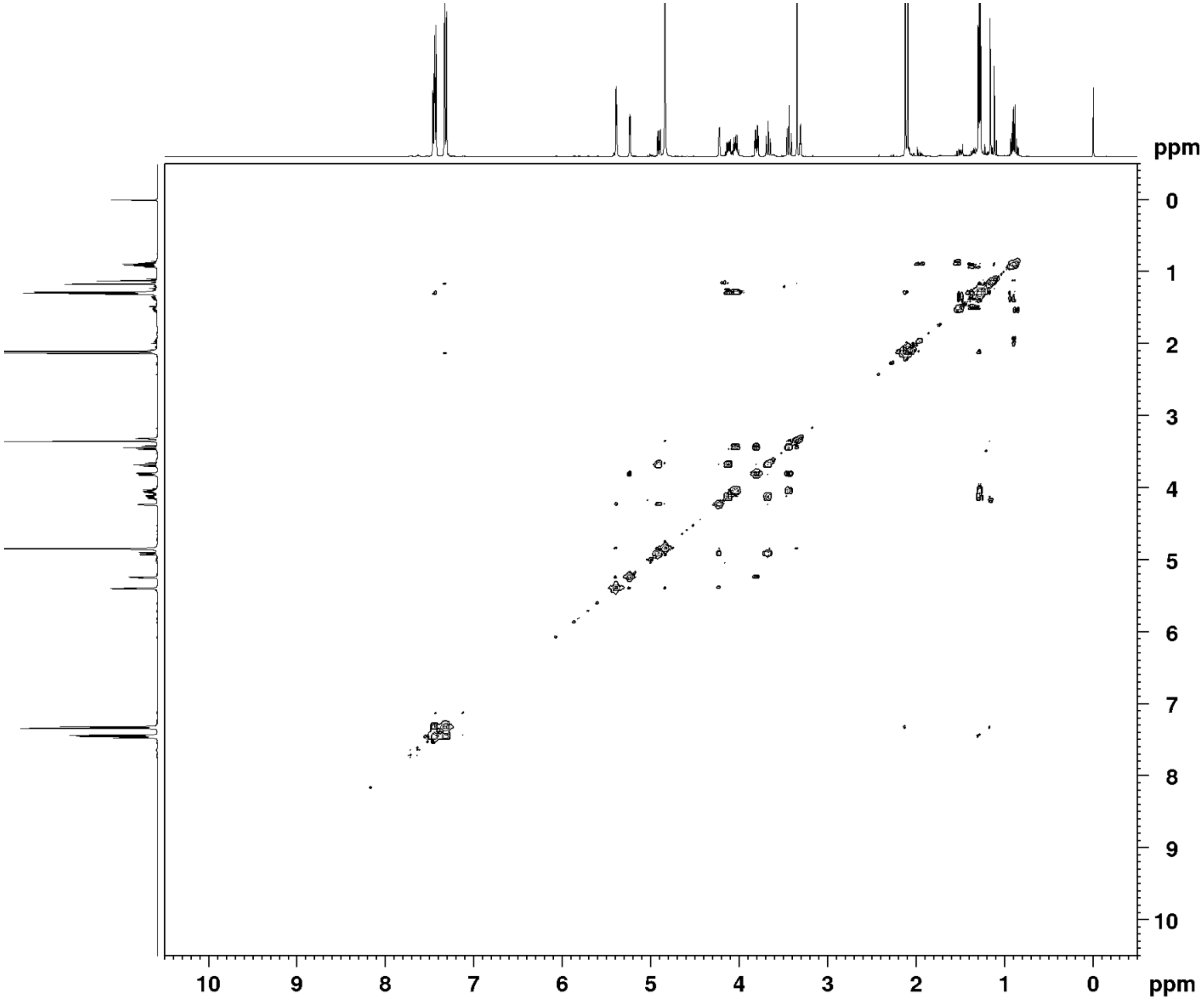

Figure S69: 4-chlorophenyl 2-*O*-acetyl-6-deoxy-1-thio-α-L-mannopyranoside (**19**) and **4-chlorophenyl 2-*O*-acetyl-6-deoxy-1-thio-α-L-mannopyranoside** (**20**) ^{1}H-$^{13}C\{^{1}H\}$ HSQC NMR (400 & 101 MHz) in MeOD

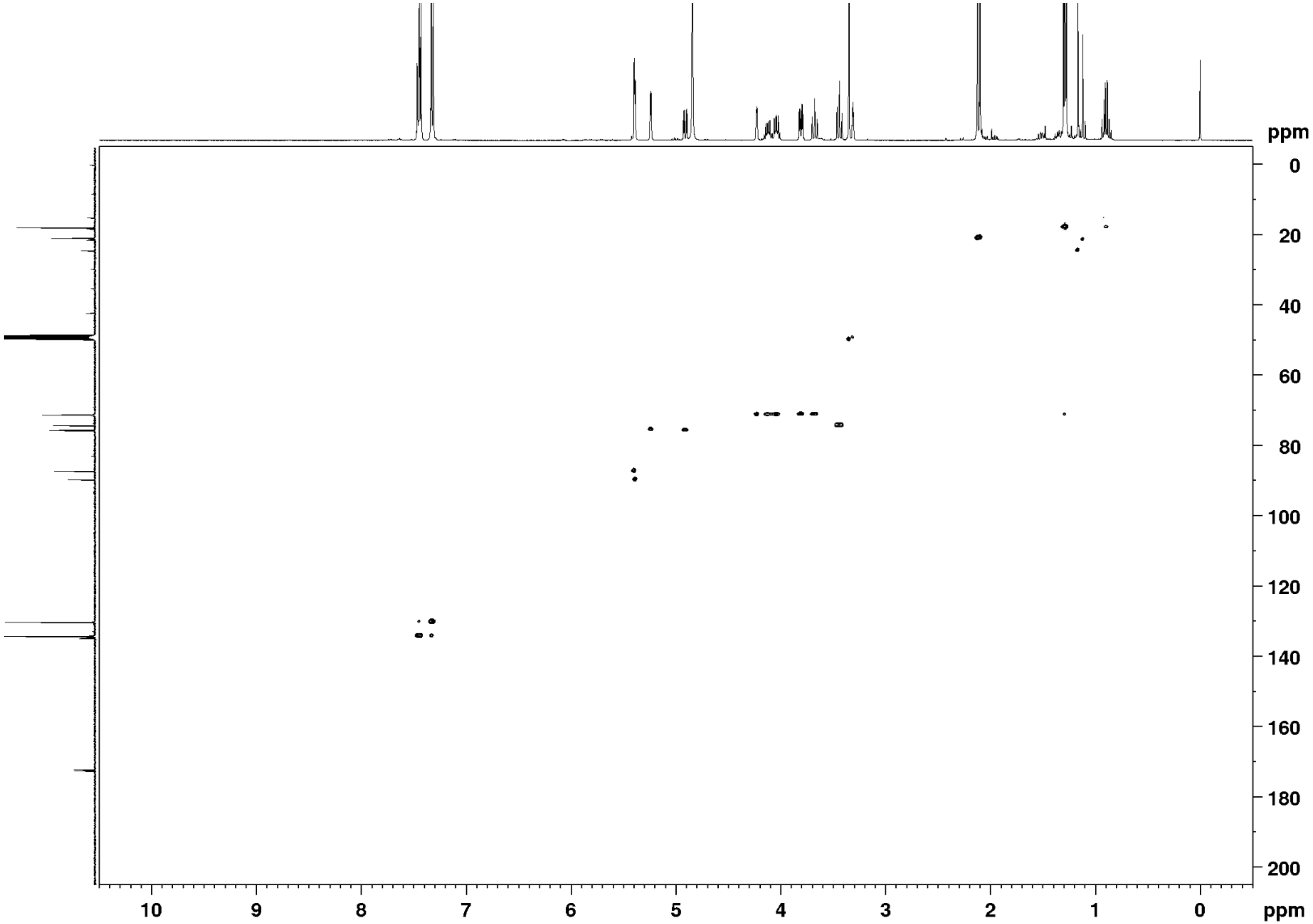

Figure S70: 4-chlorophenyl 2-*O*-acetyl-6-deoxy-1-thio-α-L-mannopyranoside (19) and **4-chlorophenyl 2-*O*-acetyl-6-deoxy-1-thio-α-L-mannopyranoside (20)** ^{1}H-$^{13}C\{^{1}H\}$ HMBC NMR (400 & 101 MHz) in MeOD

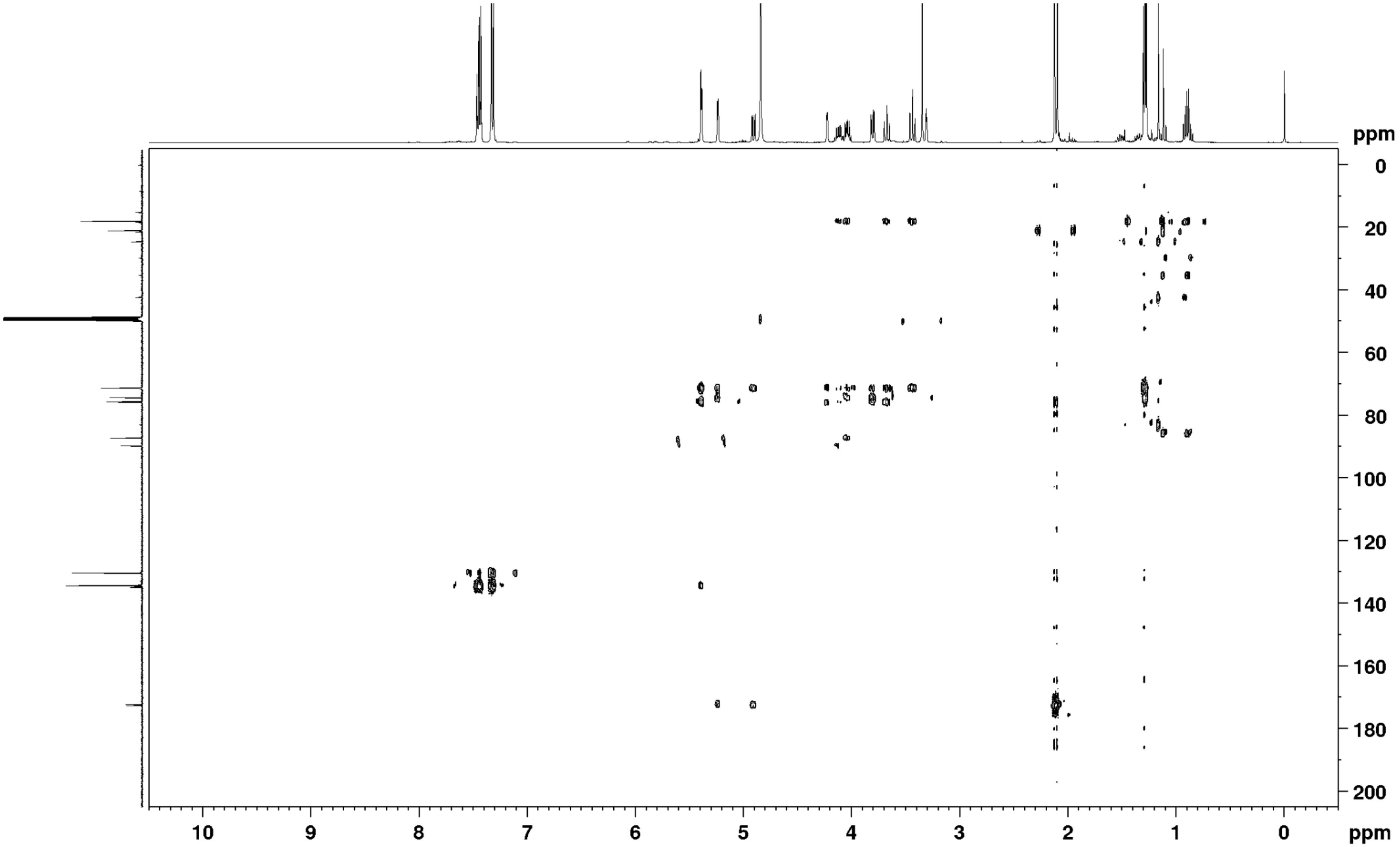

Figure S71: 4-chlorophenyl 2-*O*-acetyl-6-deoxy-1-thio-α-L-mannopyranoside (19) shown from mixture $^{13}C\{^1H\}$ NMR (101 MHz) in MeOD

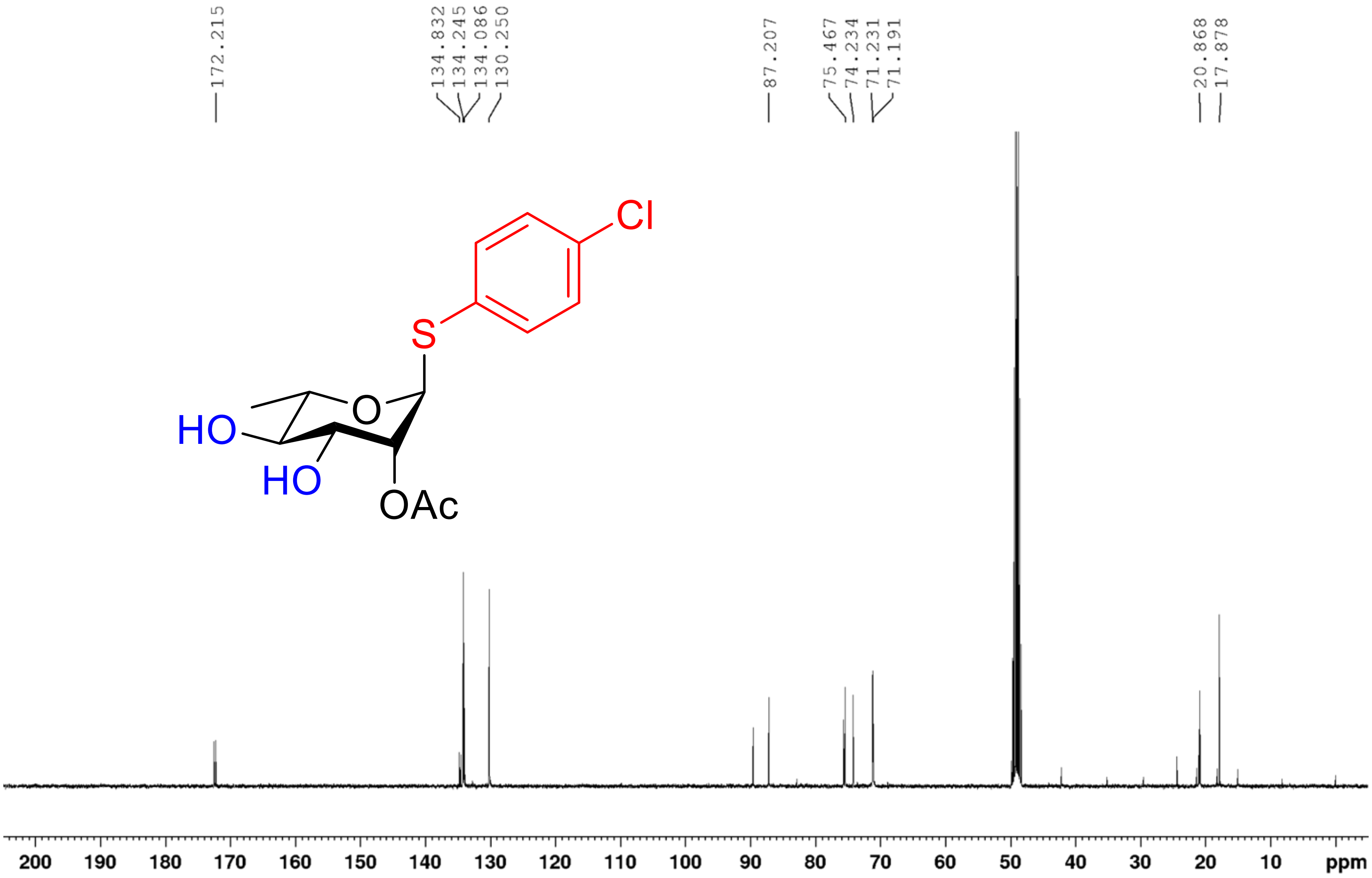

Figure S72: 4-chlorophenyl 3-*O*-acetyl-6-deoxy-1-thio-α-L-mannopyranoside (20) shown from mixture ^{1}H NMR (400 MHz) in MeOD

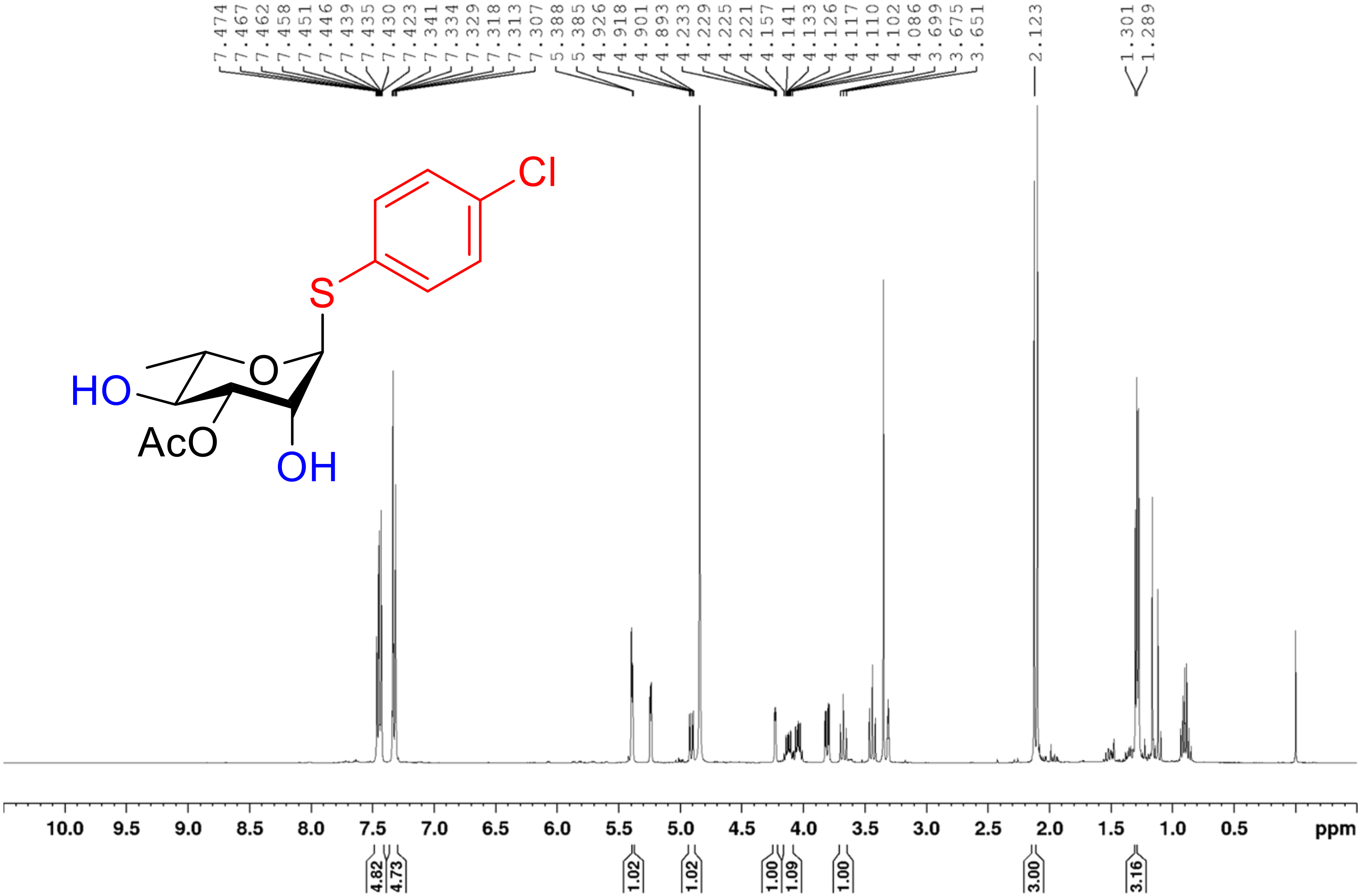

Figure S73: 4-chlorophenyl 3-*O*-acetyl-6-deoxy-1-thio-α-L-mannopyranoside (**20**) shown from mixture $^{13}C\{^{1}H\}$ NMR (101 MHz) in MeOD

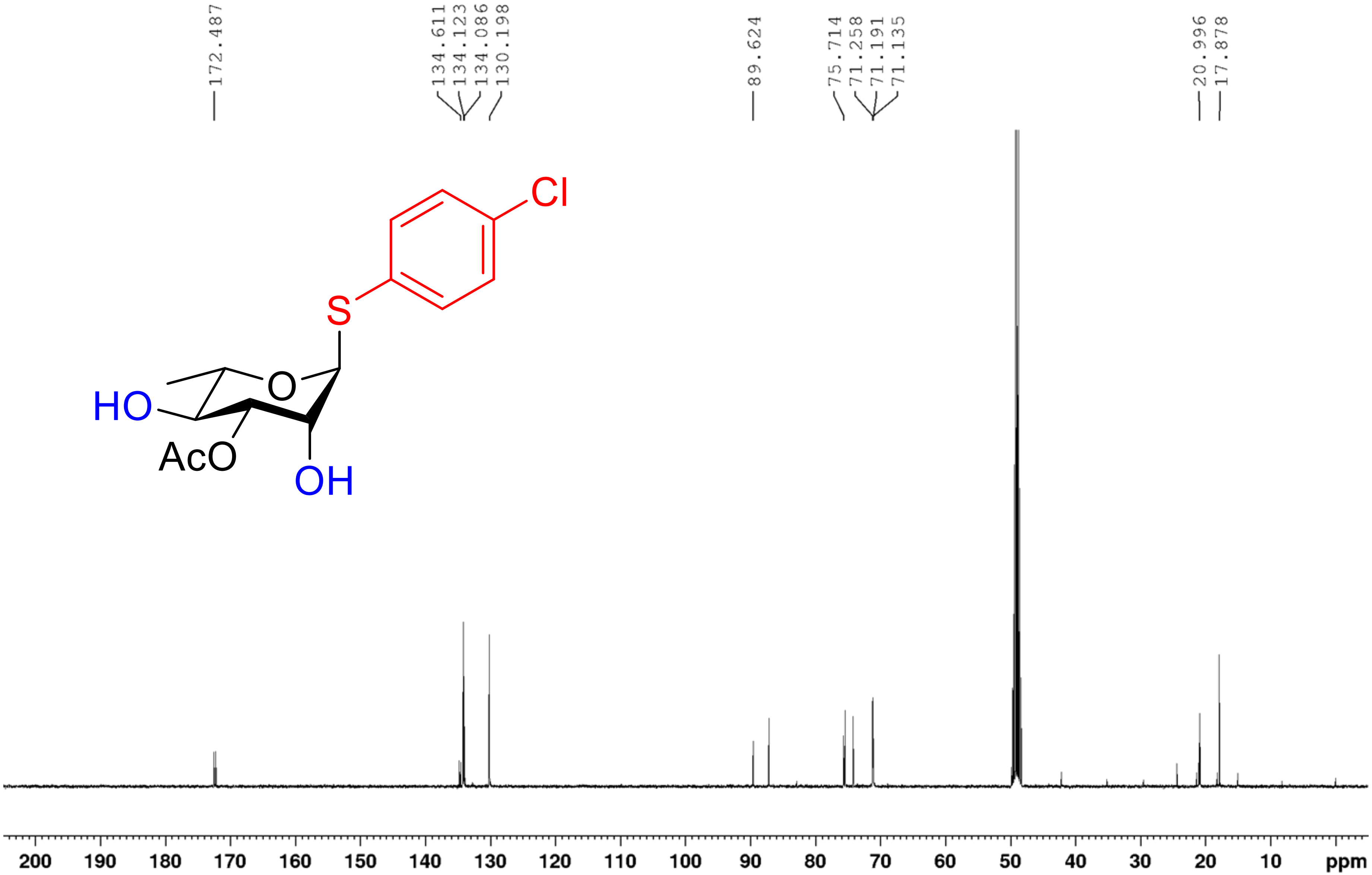

Figure S74: 4-chlorophenyl 4-*O*-acetyl-6-deoxy-1-thio-α-L-mannopyranoside (21) ^{1}H NMR (400 MHz) in MeOD

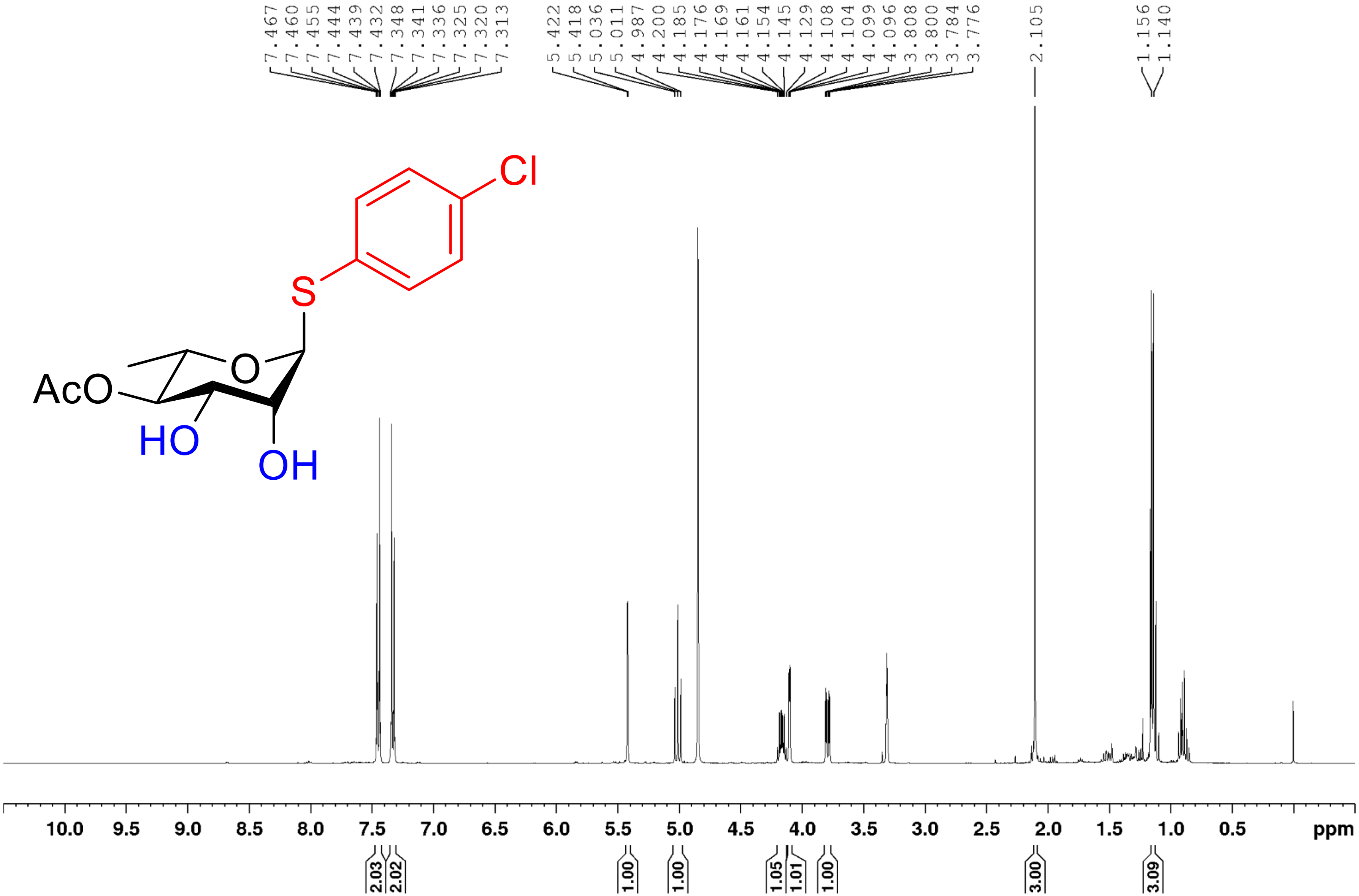

Figure S75: 4-chlorophenyl 4-*O*-acetyl-6-deoxy-1-thio-α-L-mannopyranoside (21) ^{1}H-^{1}H COSY NMR (400 MHz) in MeOD

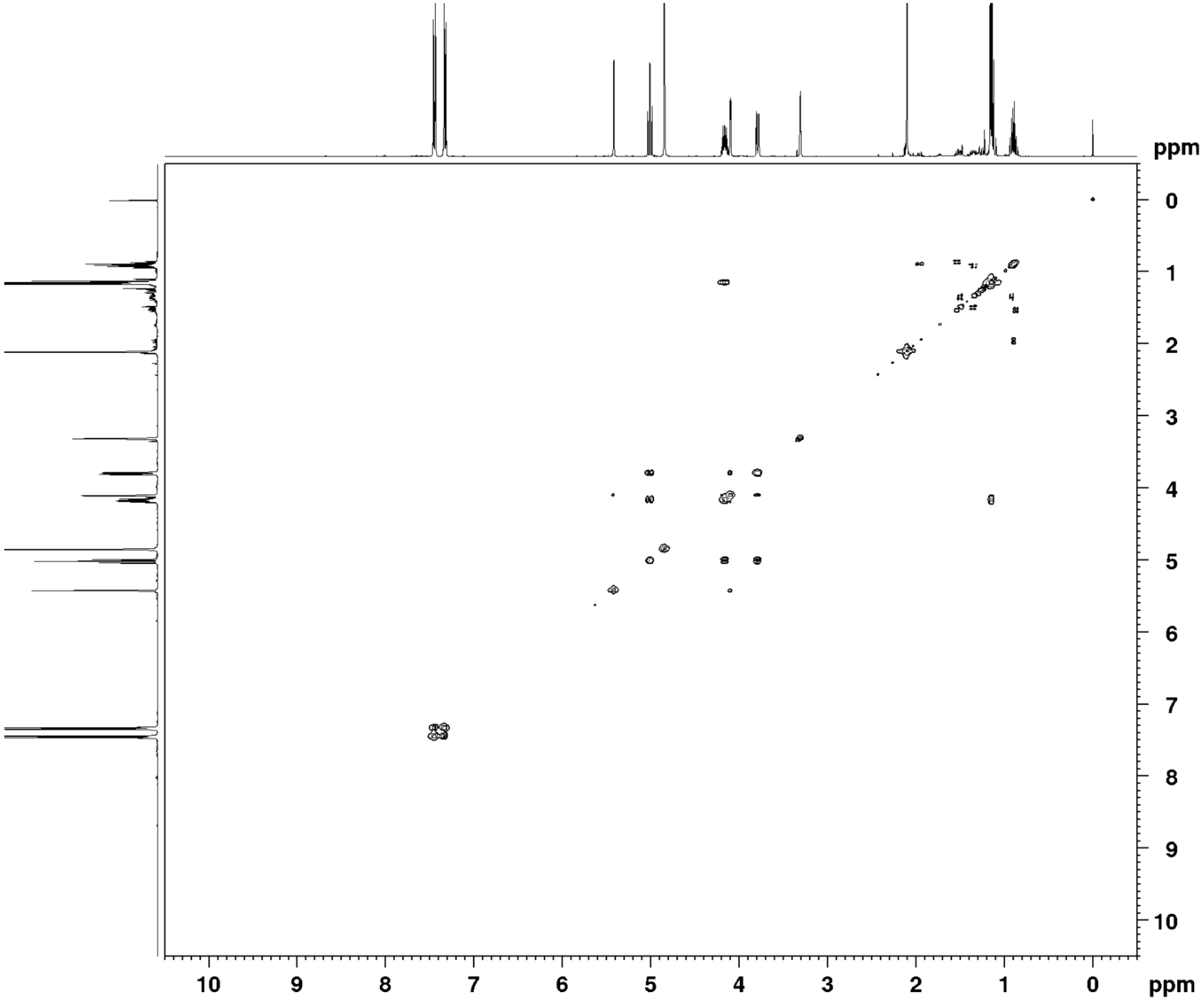

Figure S76: 4-chlorophenyl 4-*O*-acetyl-6-deoxy-1-thio-α-L-mannopyranoside (**21**) ^{1}H-^{13}C{^{1}H} HSQC NMR (400 & 101 MHz) in MeOD

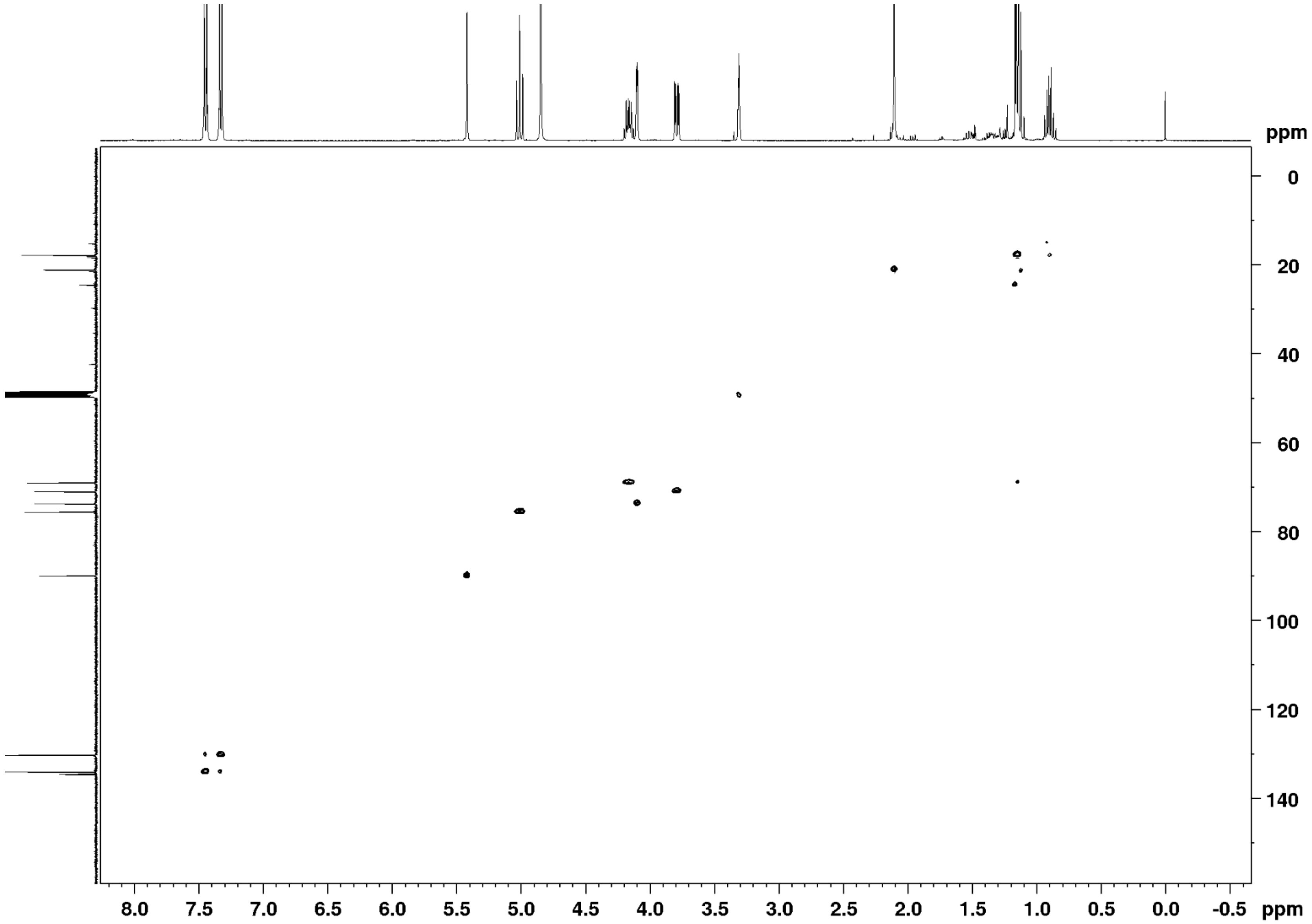

Figure S77: 4-chlorophenyl 4-*O*-acetyl-6-deoxy-1-thio-α-L-mannopyranoside (21) ^{1}H-$^{13}C\{^{1}H\}$ HMBC NMR (400 & 101 MHz) in MeOD

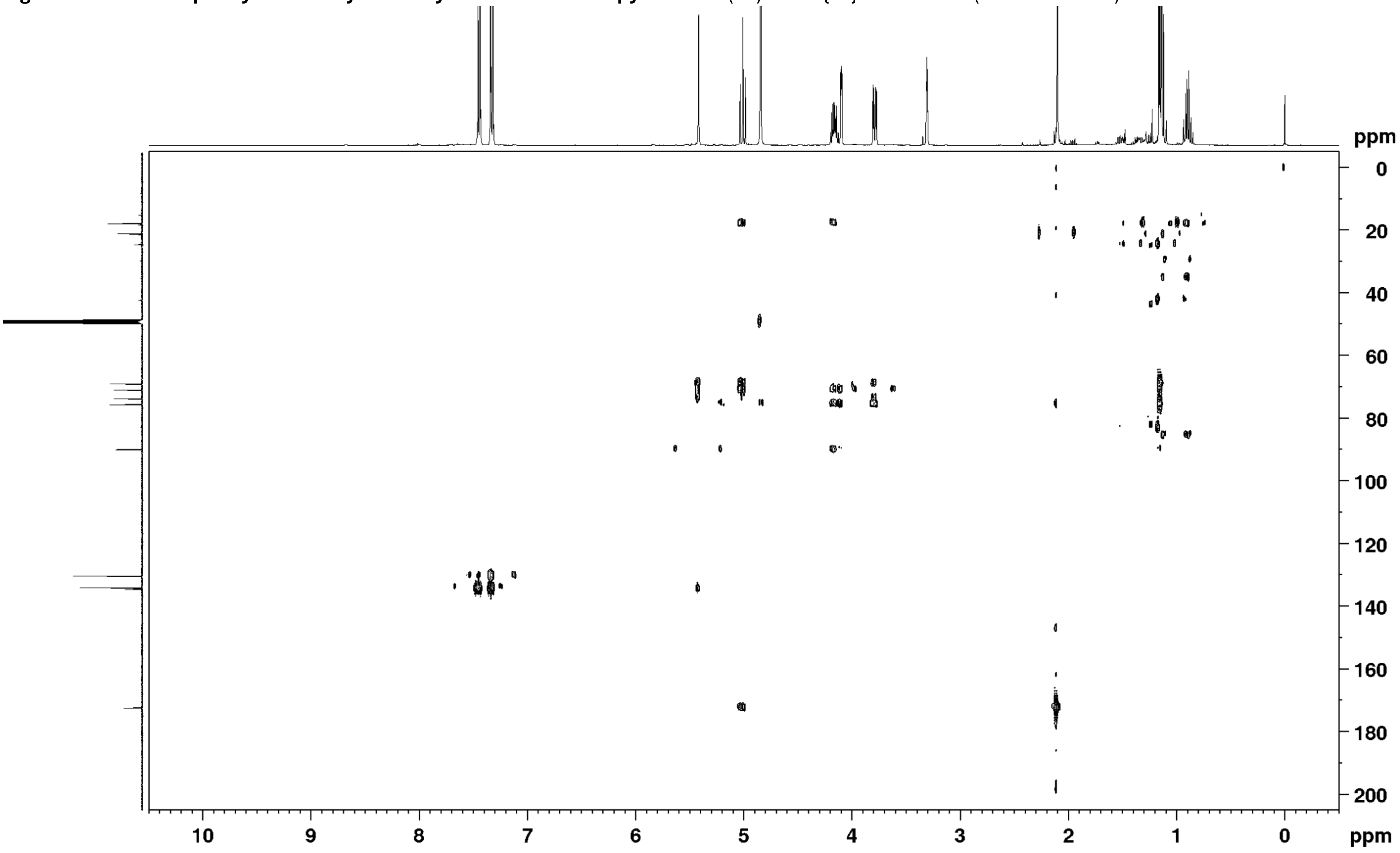

Figure S78: 4-chlorophenyl 4-*O*-acetyl-6-deoxy-1-thio-α-L-mannopyranoside (**21**) $^{13}C\{^{1}H\}$ NMR (101 MHz) in MeOD

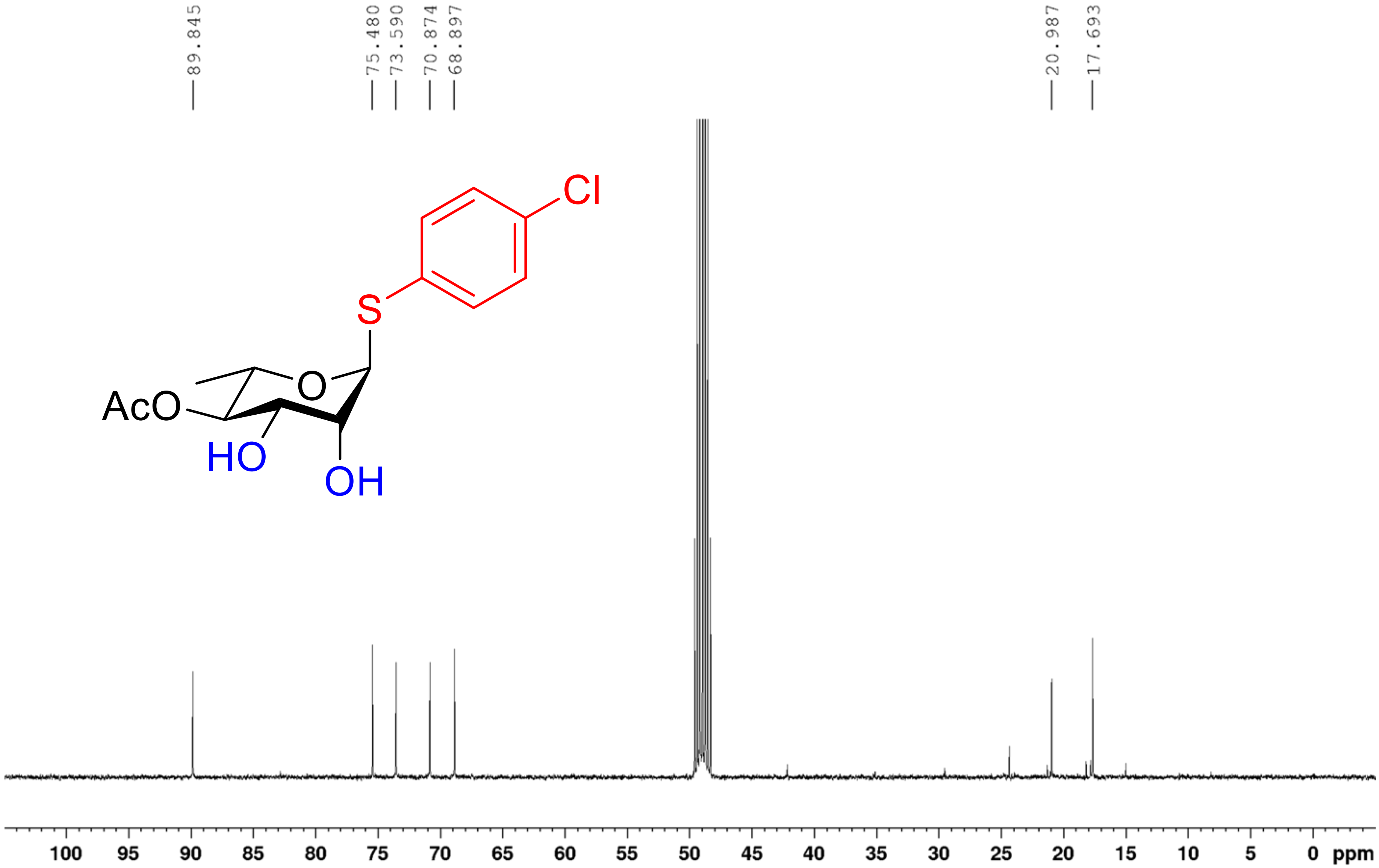

Figure S79: 4-chlorophenyl (β-D-galactopyranosyl)-(1→4)-1-thio-β-D-glucopyranoside (22) ^{1}H NMR (400 MHz) in MeOD+D_2O 0.65+0.2 mL

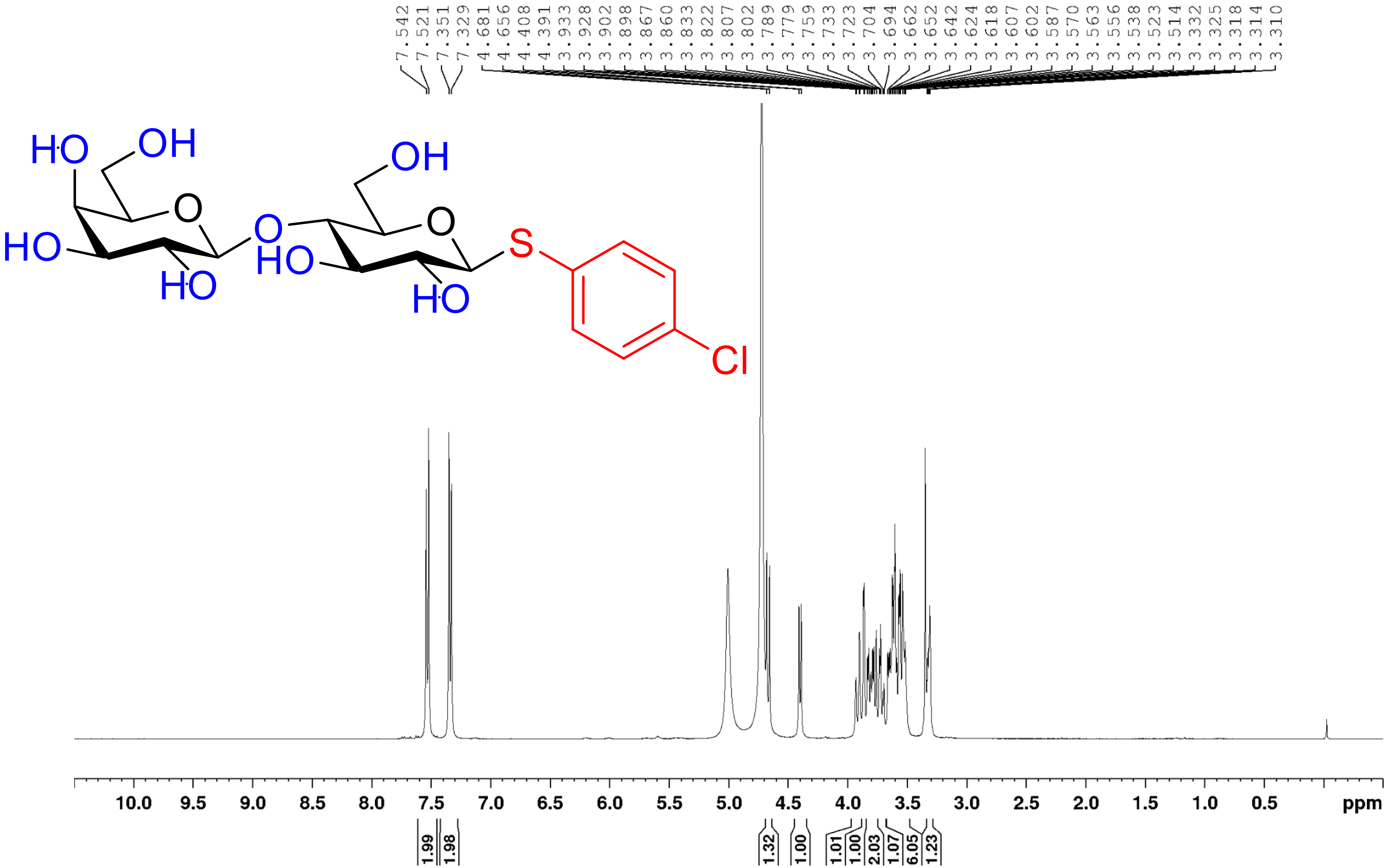

Figure S80: 4-chlorophenyl (β-D-galactopyranosyl)-(1→4)-1-thio-β-D-glycopyranoside (22) ^1H-^1H COSY NMR (400 MHz) in MeOD+D_2O 0.65+0.2 mL

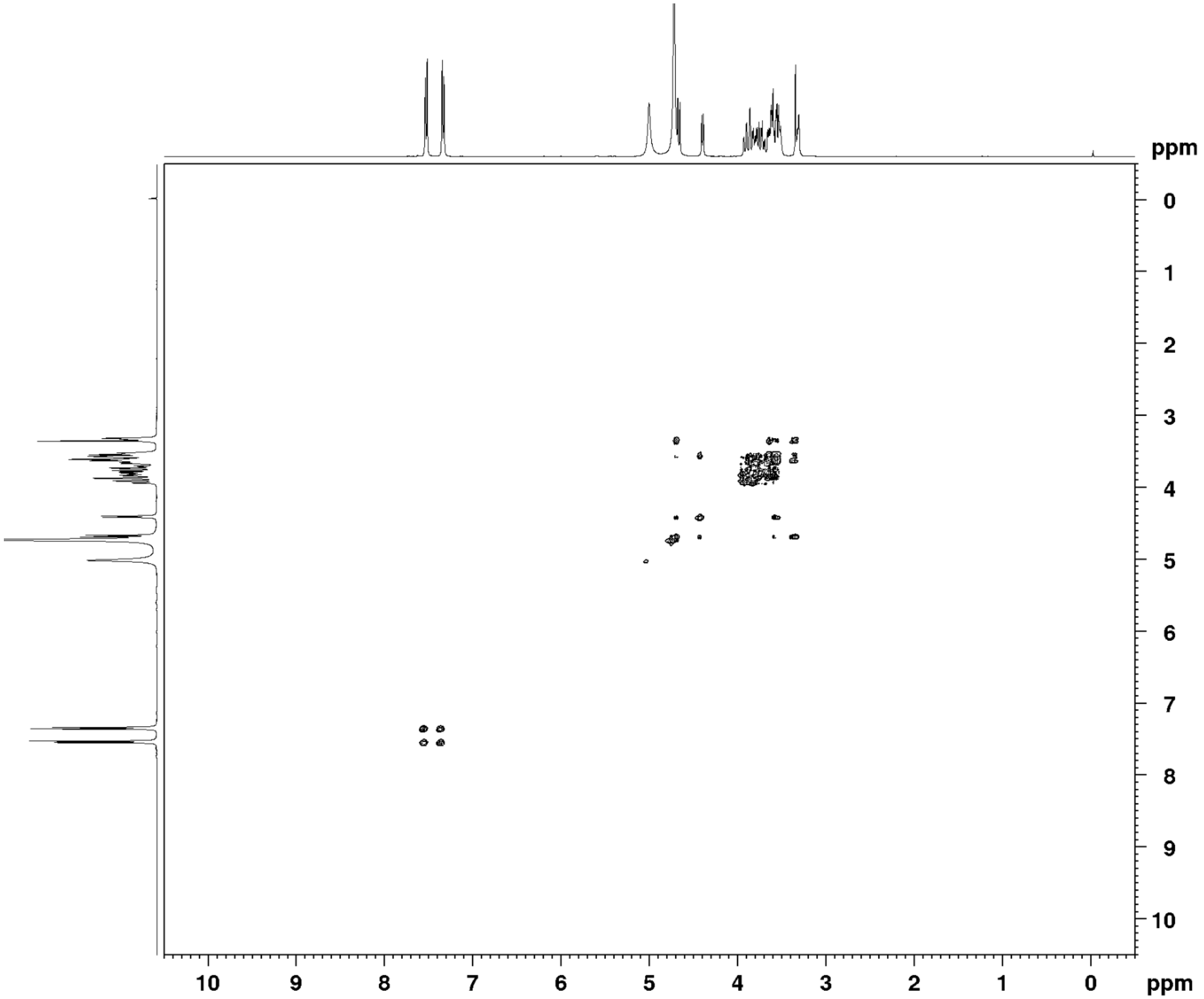

Figure S81: 4-chlorophenyl (β-D-galactopyranosyl)-(1→4)-1-thio-β-D-glycopyranoside (**22**) ^1H-$^{13}C\{^1H\}$ HSQC NMR (400 & 101 MHz) in MeOD+D_2O 0.65+0.2 mL

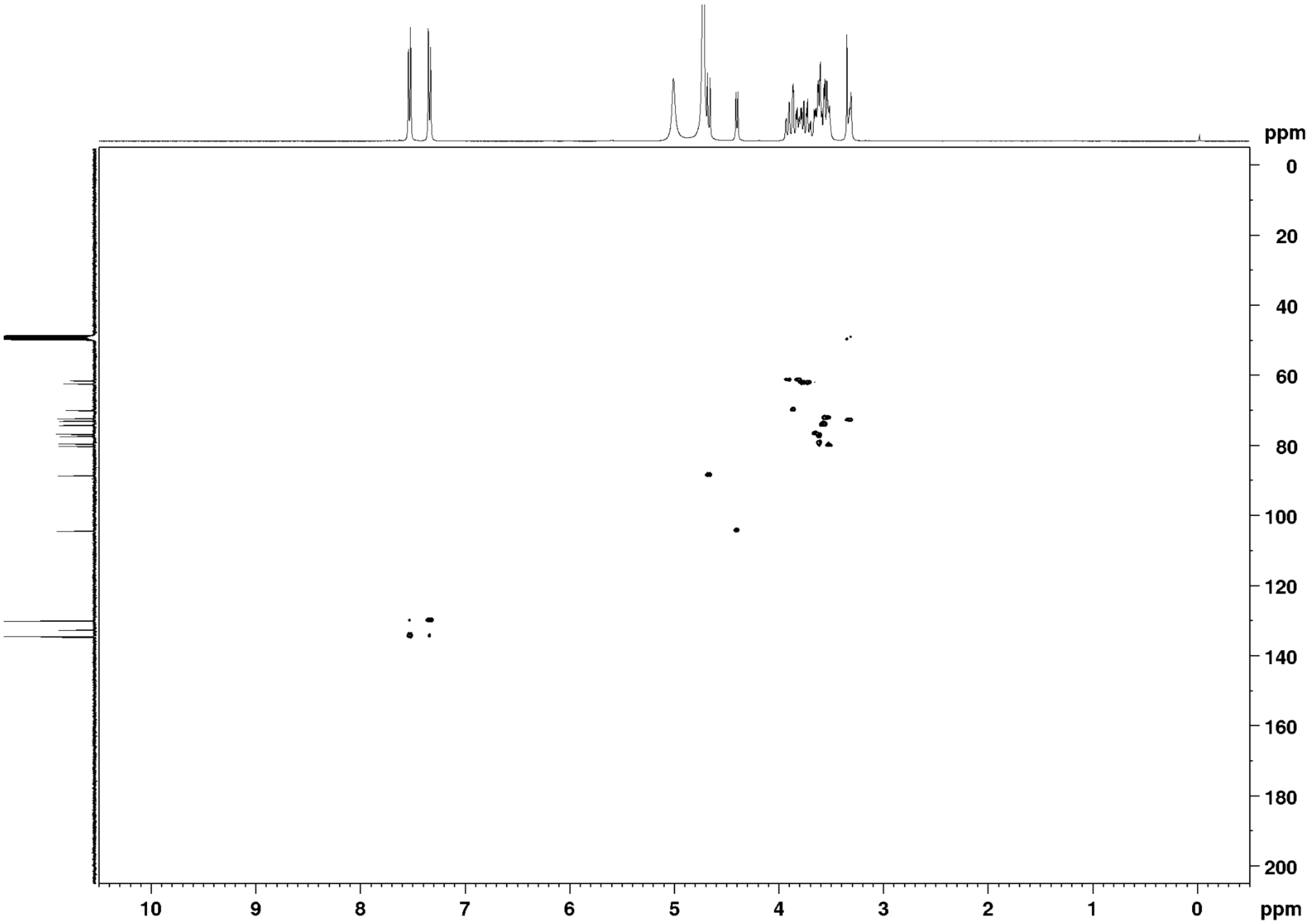

Figure S82: 4-chlorophenyl (β-D-galactopyranosyl)-(1→4)-1-thio-β-D-glycopyranoside (22) ^{1}H-$^{13}C\{^{1}H\}$ HMBC NMR (400 & 101 MHz) in MeOD+D_2O 0.65+0.2 mL

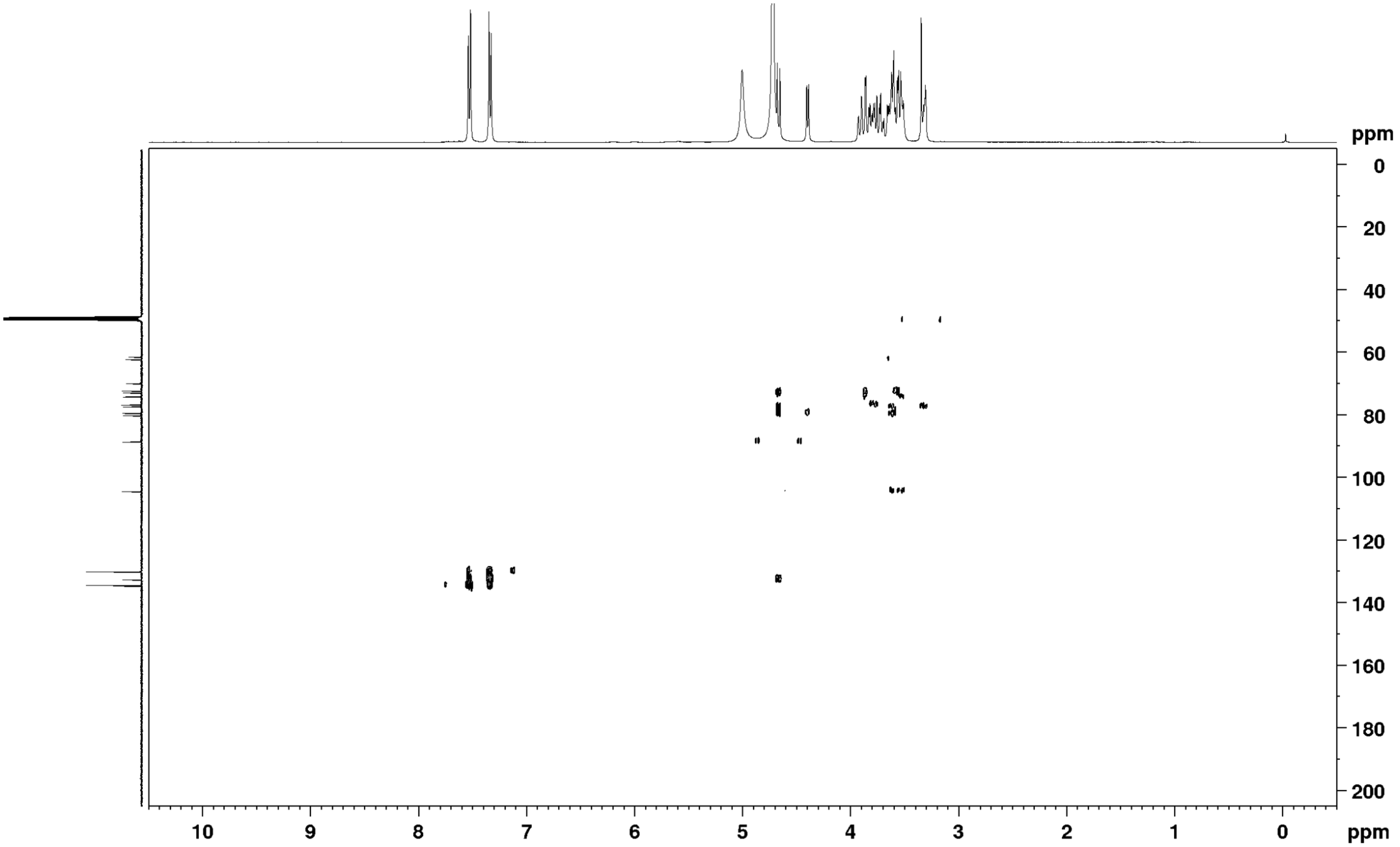

Figure S83: 4-chlorophenyl (β-D-galactopyranosyl)-(1→4)-1-thio-β-D-glycopyranoside (22) $^{13}C\{^1H\}$ NMR (101 MHz) in MeOD+D_2O 0.65+0.2 mL

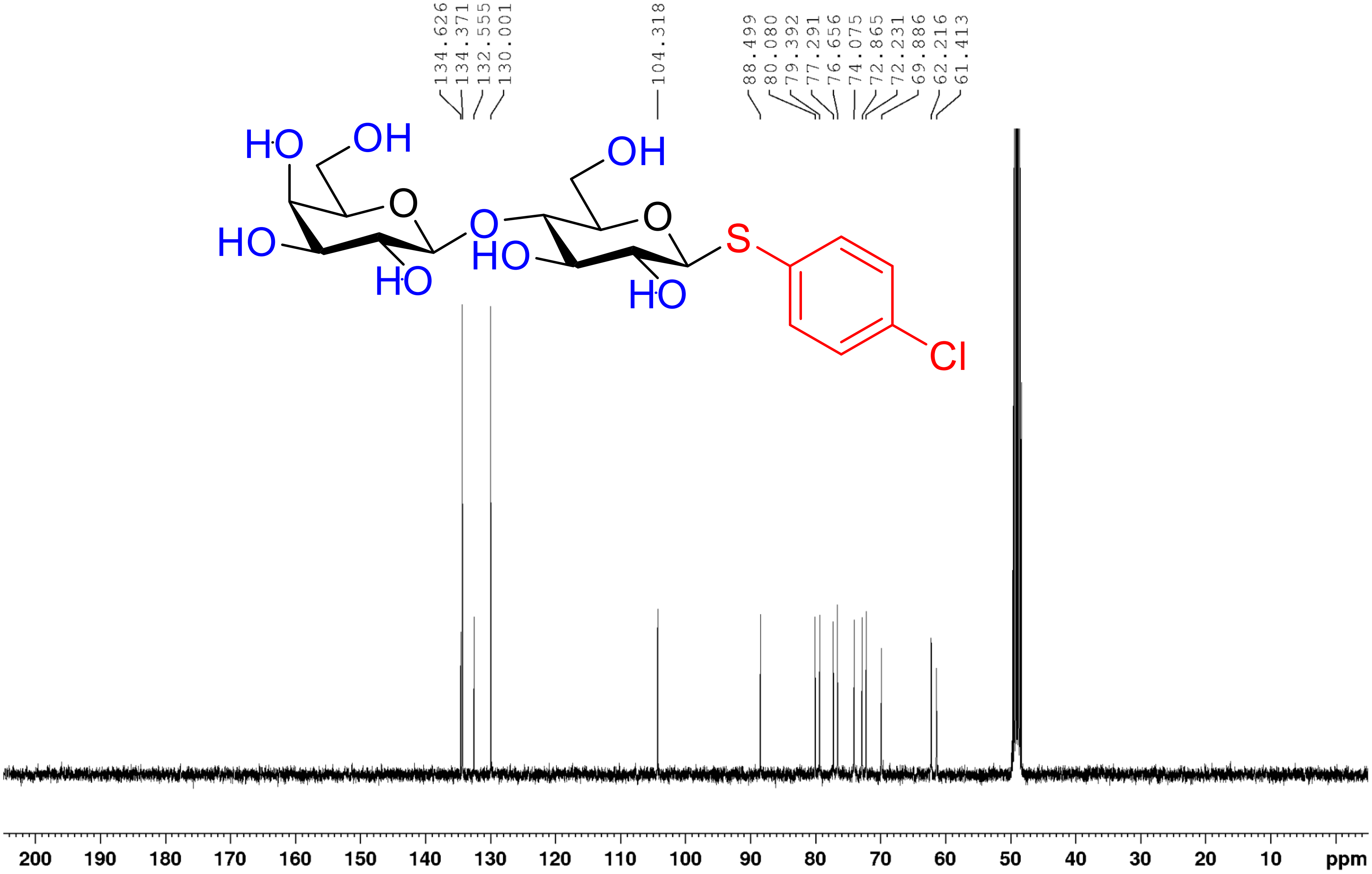

Figure S84: 3,6-di-*O*-acetyl-D-glucopyranose (**24**) α:β 61:39 ^{1}H NMR (400 MHz) in MeOD

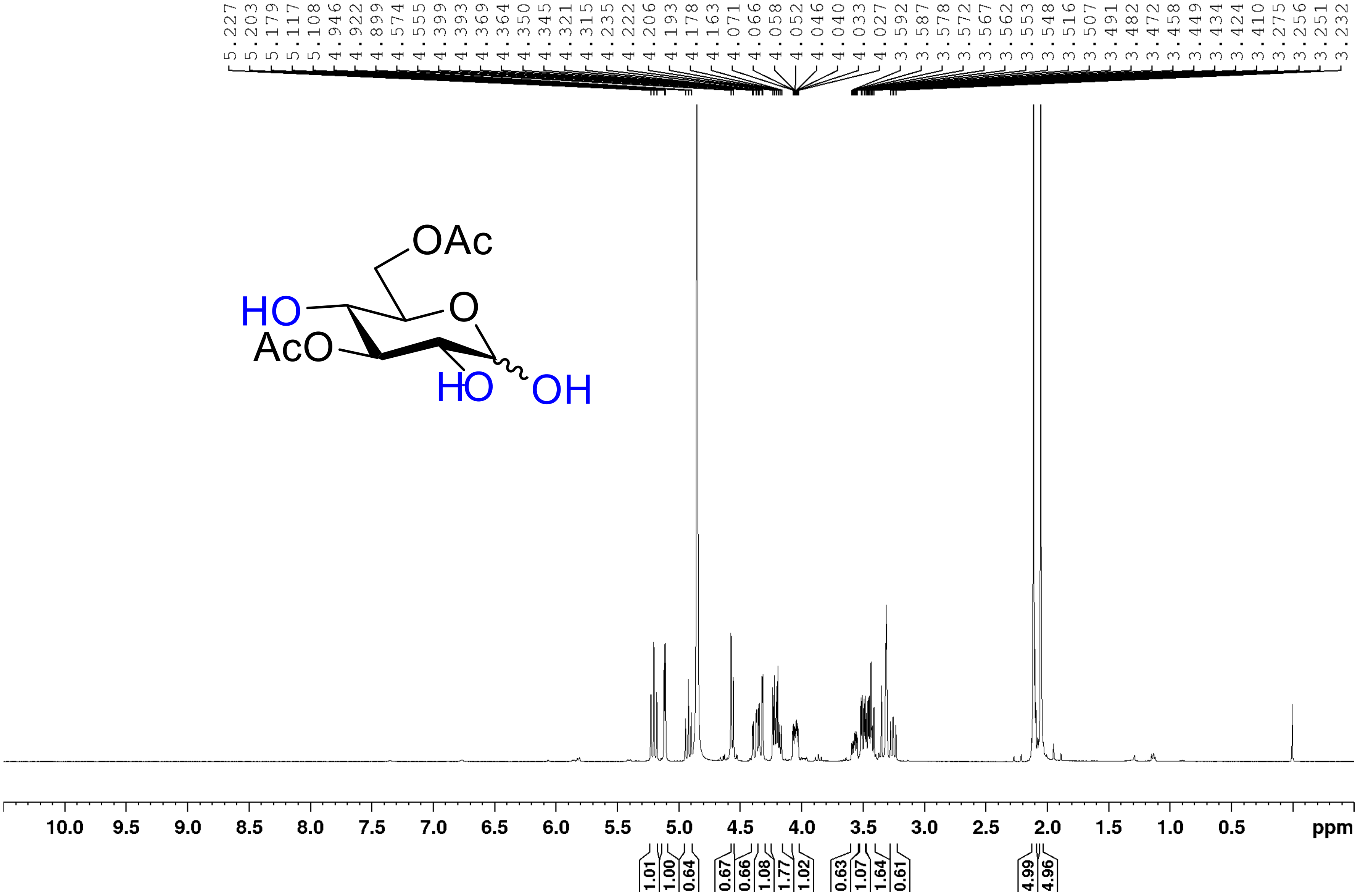

Figure S85: 3,6-di-*O*-acetyl-D-glucopyranose (**24**) α:β 61:39 ^{1}H-^{1}H COSY NMR (400 MHz) in MeOD

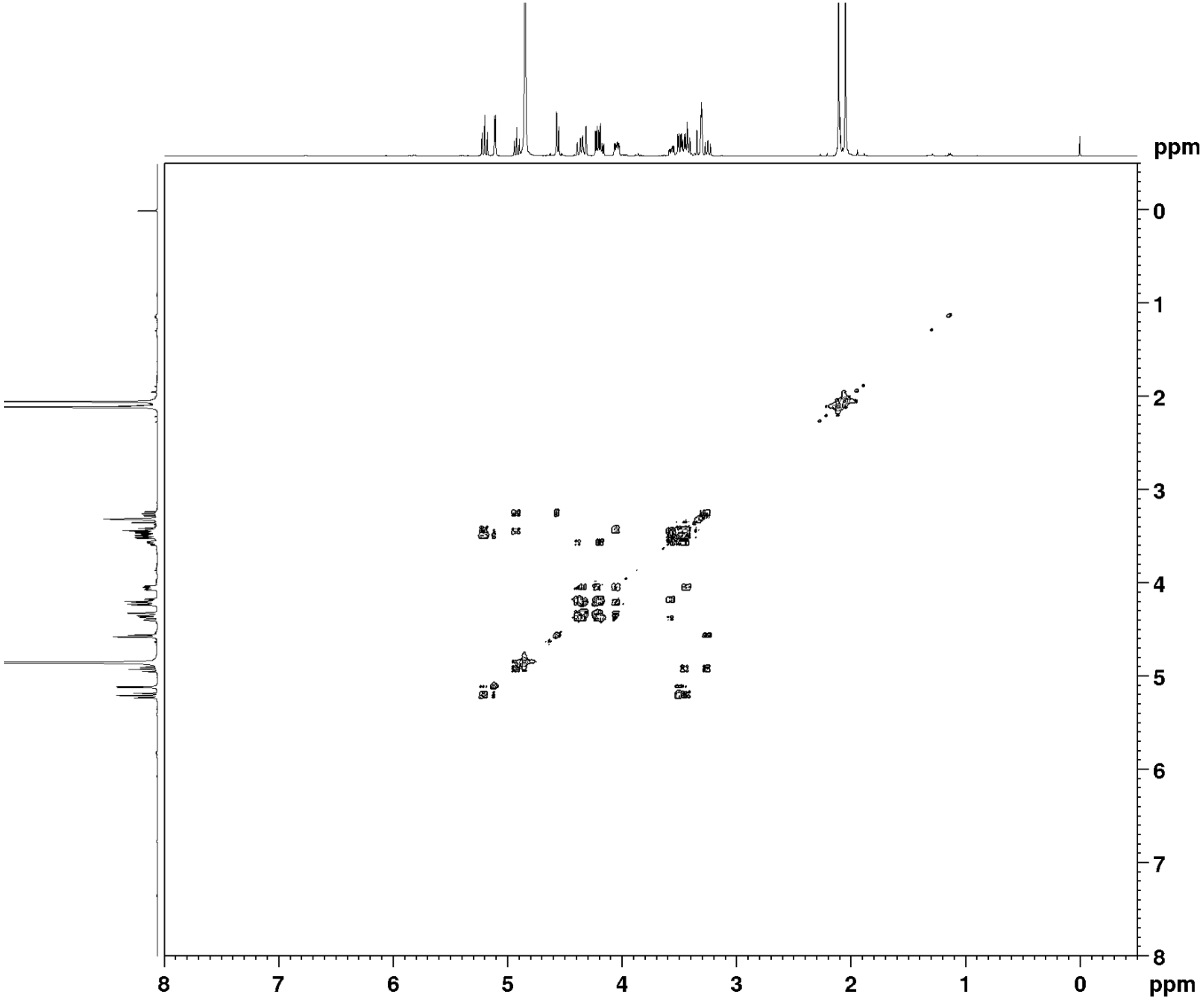

Figure S86: 3,6-di-*O*-acetyl-D-glucopyranose (**24**) α:β 61:39 ^{1}H-^{13}C{^{1}H} HSQC NMR (400 & 101 MHz) in MeOD

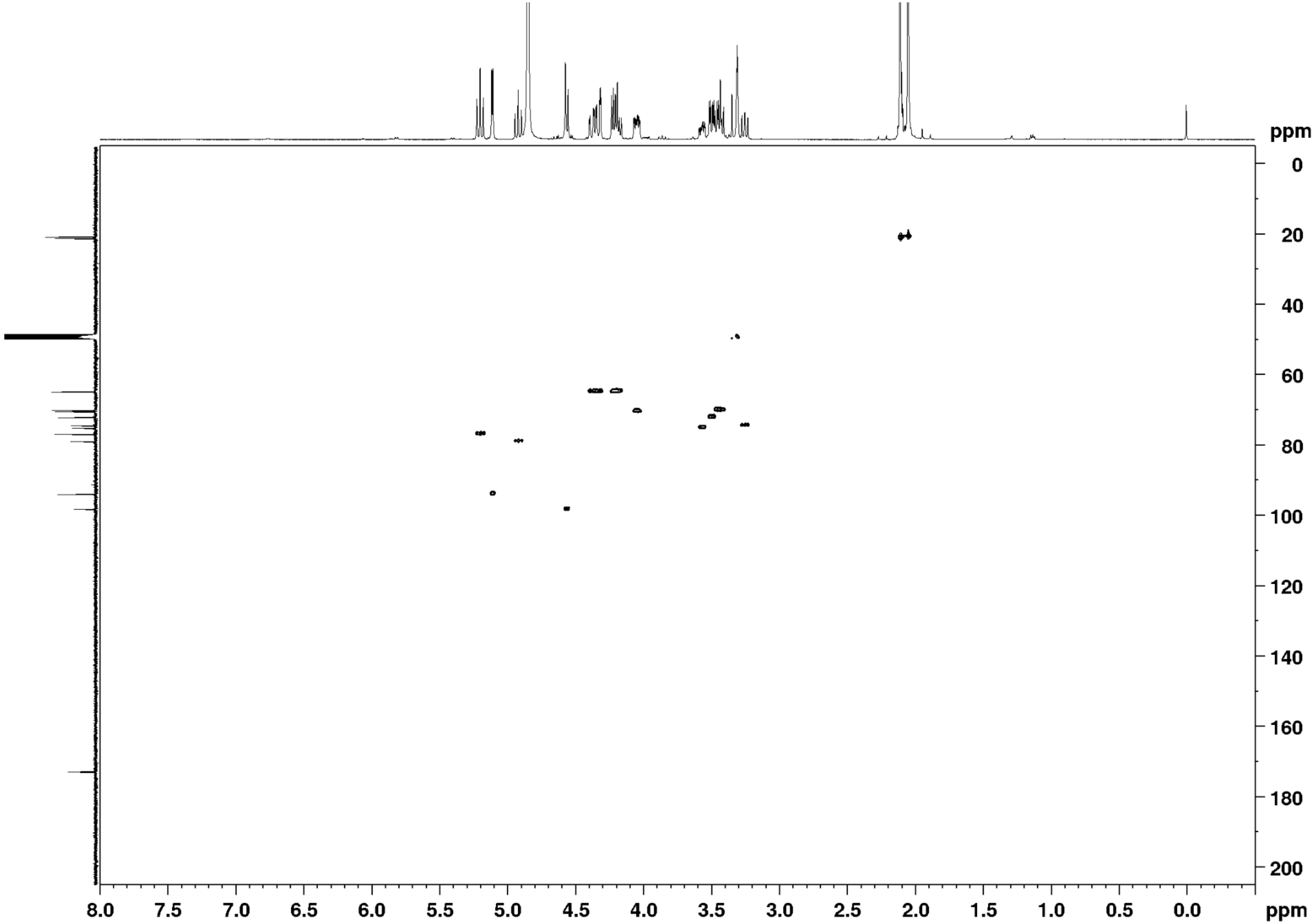

Figure S87: 3,6-di-*O*-acetyl-D-glucopyranose (**24**) α:β 61:39 ^{1}H-$^{13}C\{^{1}H\}$ HMBC NMR (400 & 101 MHz) in MeOD

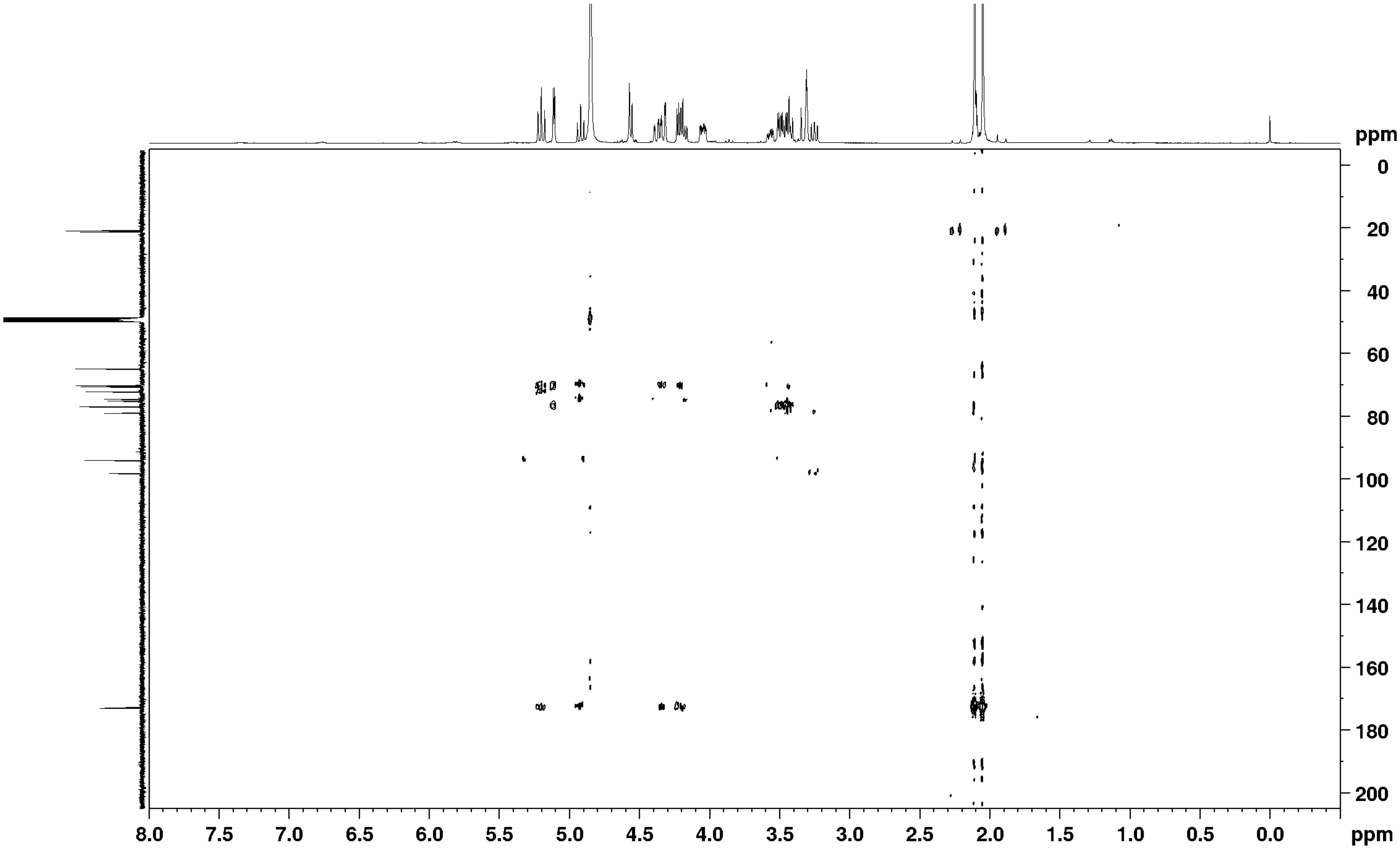

Figure S88: 2-acetomido-6-*O*-acetyl-2-deoxy-D-glucopyranose (**27**) α:β 97:3 ^{1}H NMR (400 MHz) in D_2O

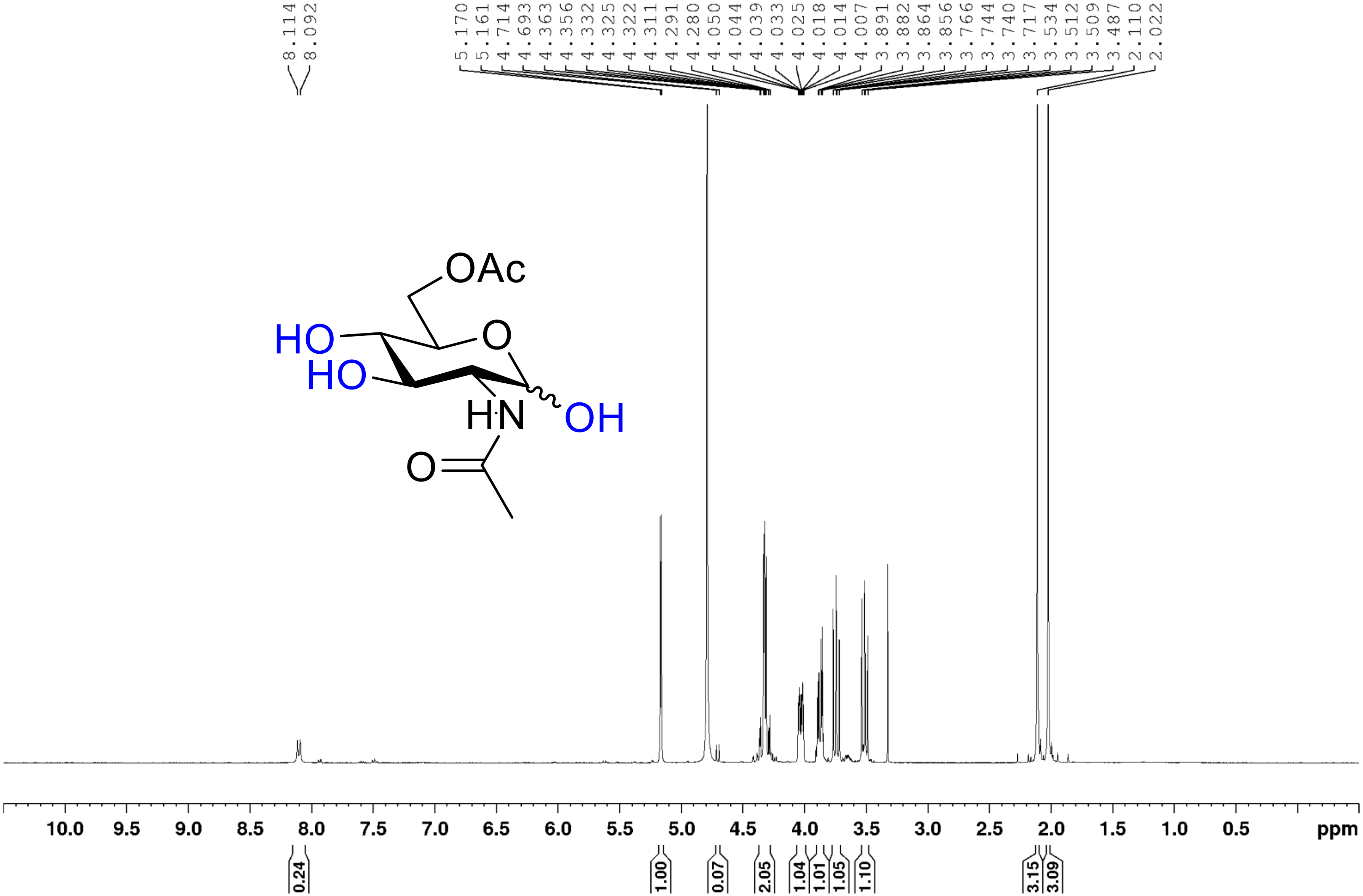

Figure S89: 2-acetomido-6-*O*-acetyl-2-deoxy-D-glucopyranose (**27**) α:β 97:3 ^{1}H-^{1}H COSY NMR (400 MHz) in D_2O

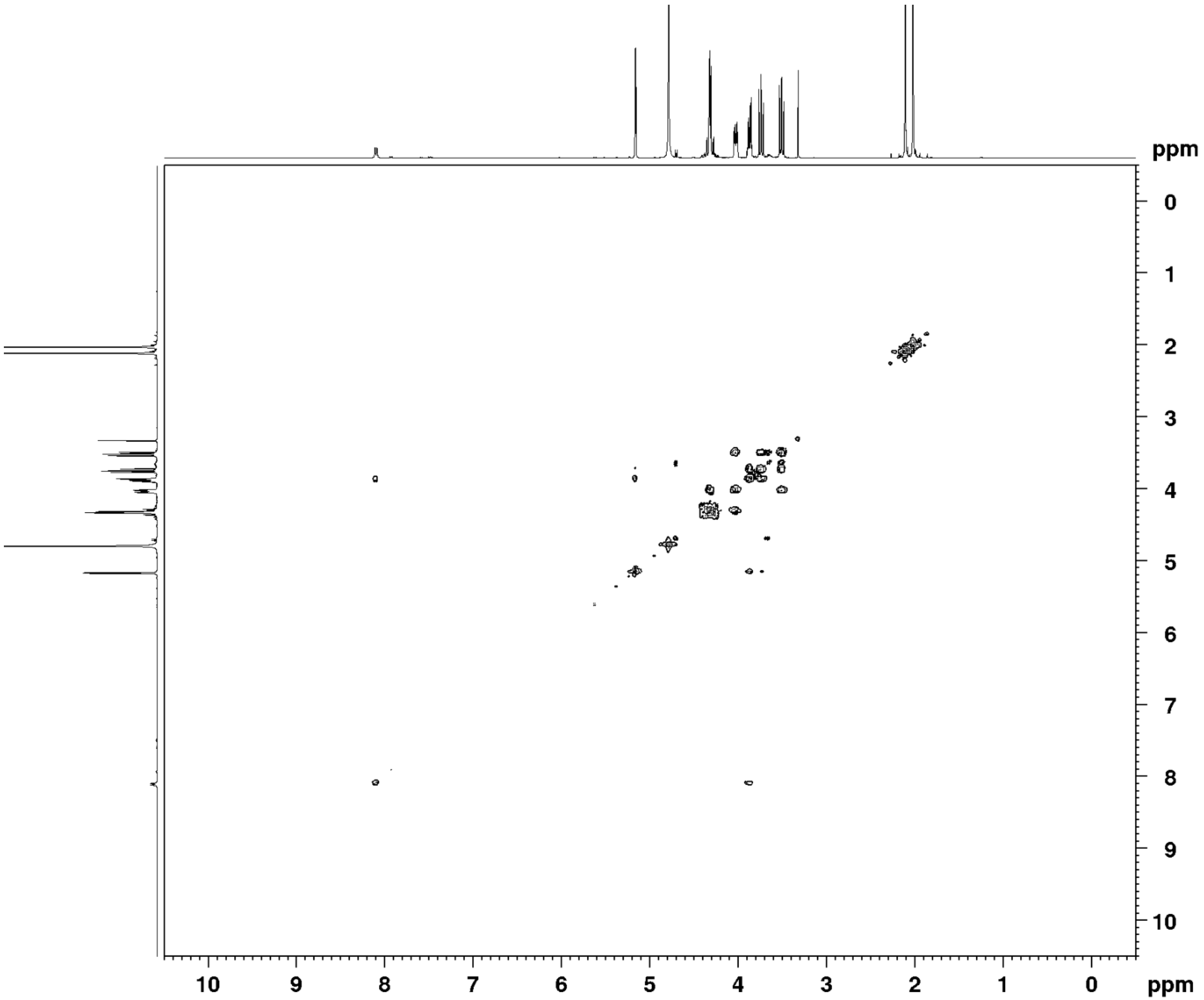

Figure S90: 2-acetomido-6-*O*-acetyl-2-deoxy-D-glucopyranose (**27**) α:β 97:3 ^{1}H-^{13}C{^{1}H} HSQC NMR (400 & 101 MHz) in D_2O

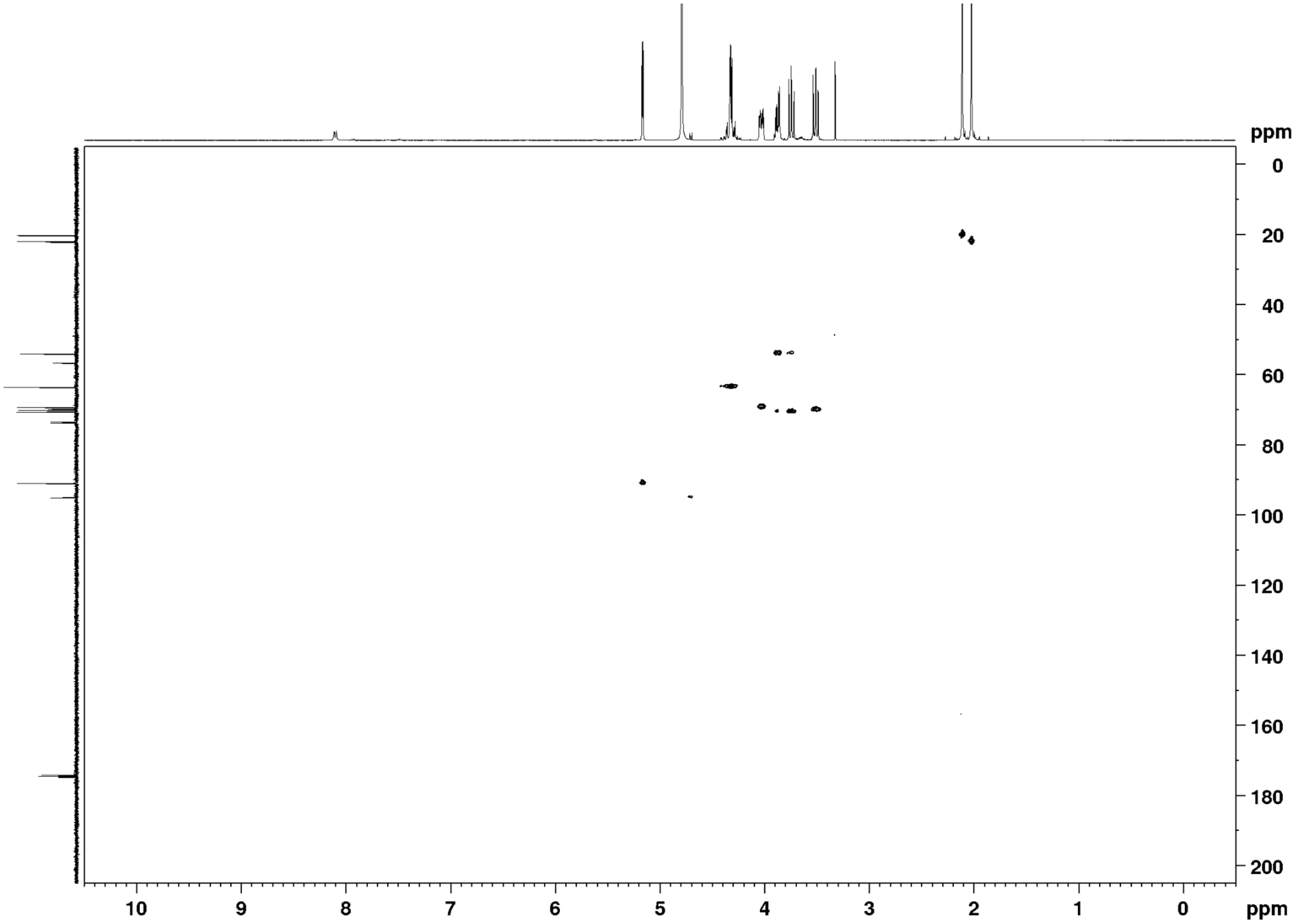

Figure S91: 2-acetomido-6-*O*-acetyl-2-deoxy-D-glucopyranose (**27**) α:β 97:3 ^{1}H-$^{13}C\{^{1}H\}$ HMBC NMR (400 & 101 MHz) in D_2O

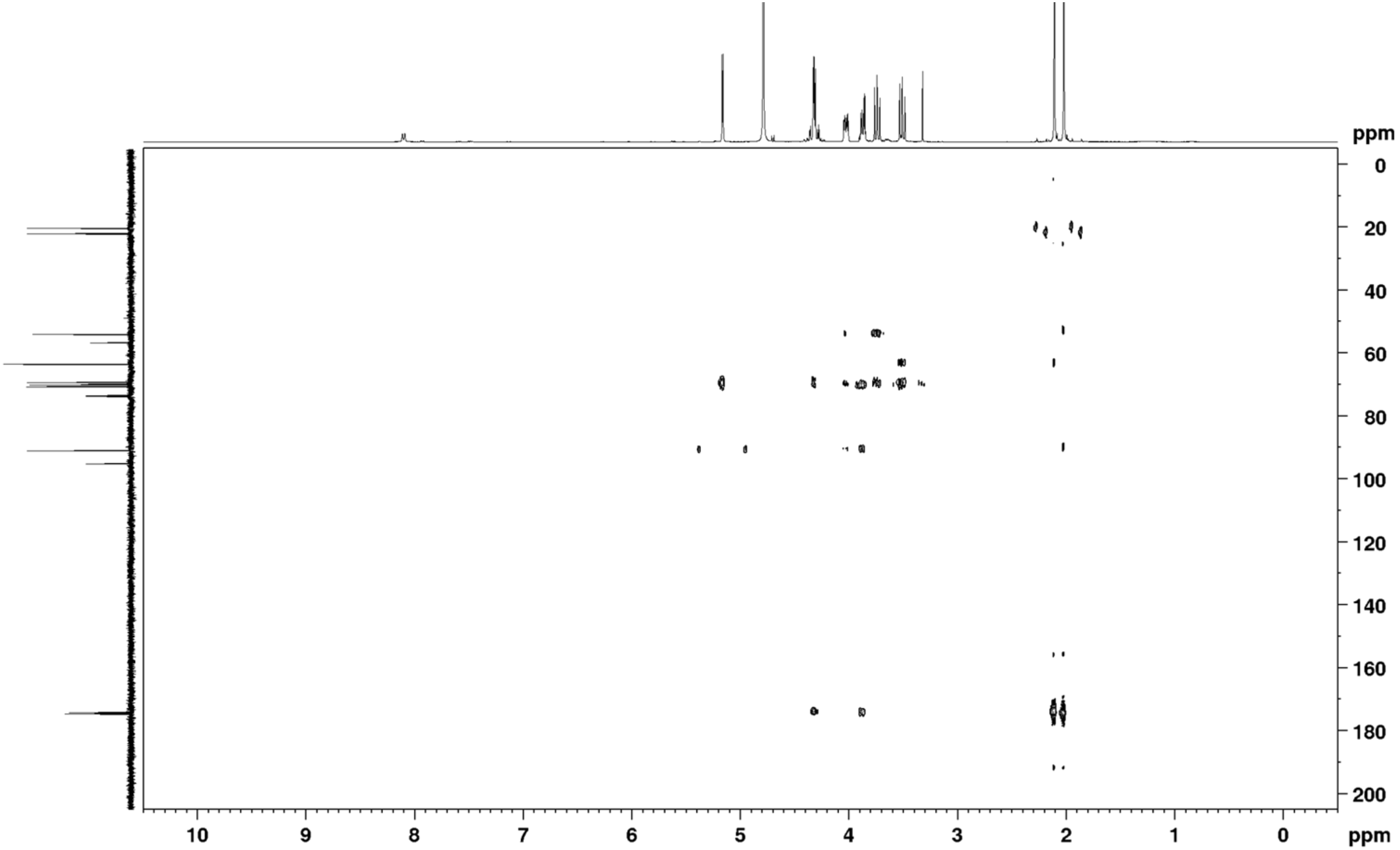

Figure S92: 2-deoxy-2-(2,2,2-trichloroethoxycarbonylamino)-D-glucopyranose (**28**) α:β:Fur 79:11:10 ^{1}H NMR (400 MHz) in MeOD

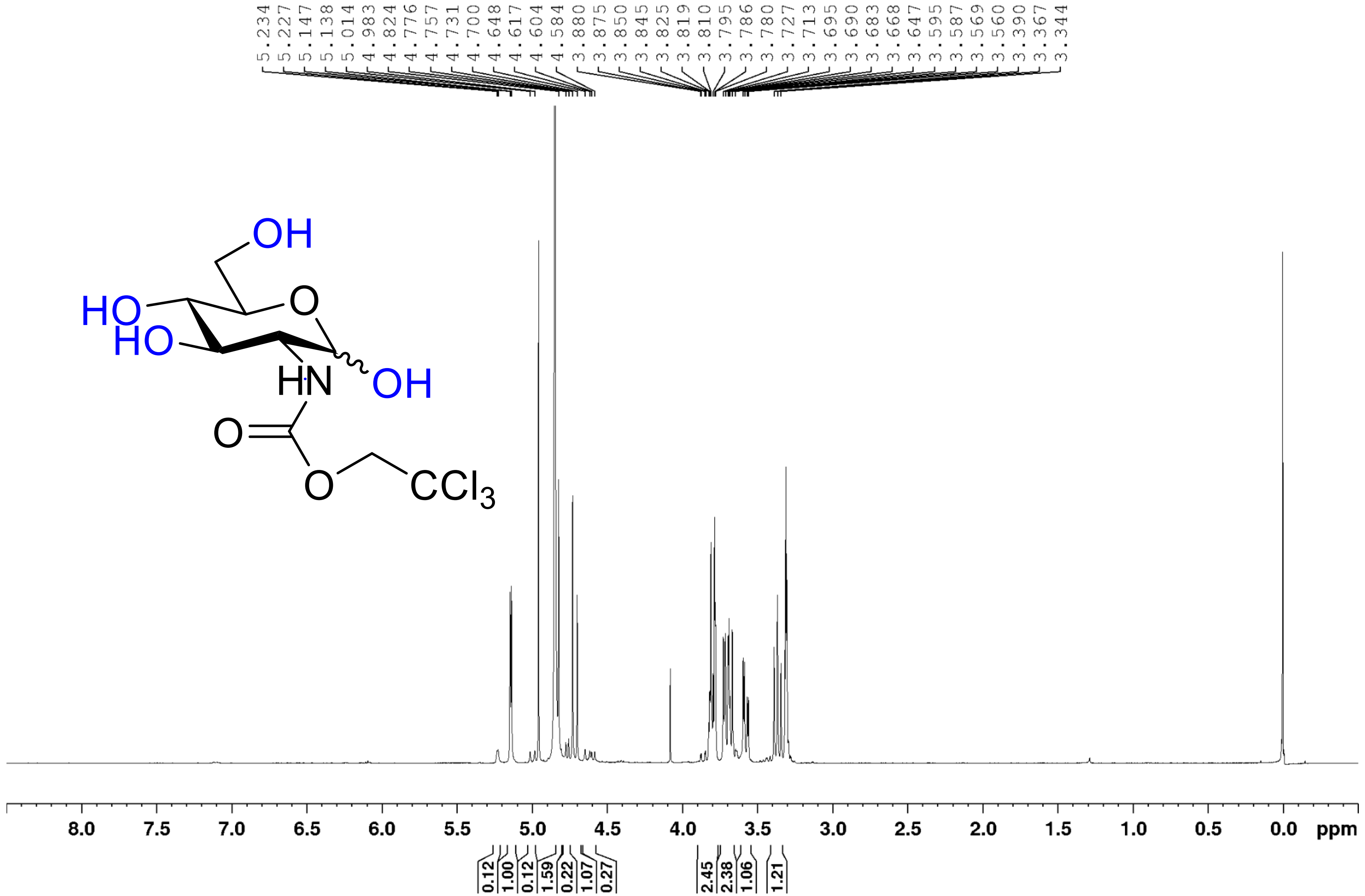

Figure S93: 6-*O*-acetyl-2-deoxy-2-(2,2,2-trichloroethoxycarbonylamino)-D-glucopyranose (29) ^{1}H NMR (400 MHz) in MeOD

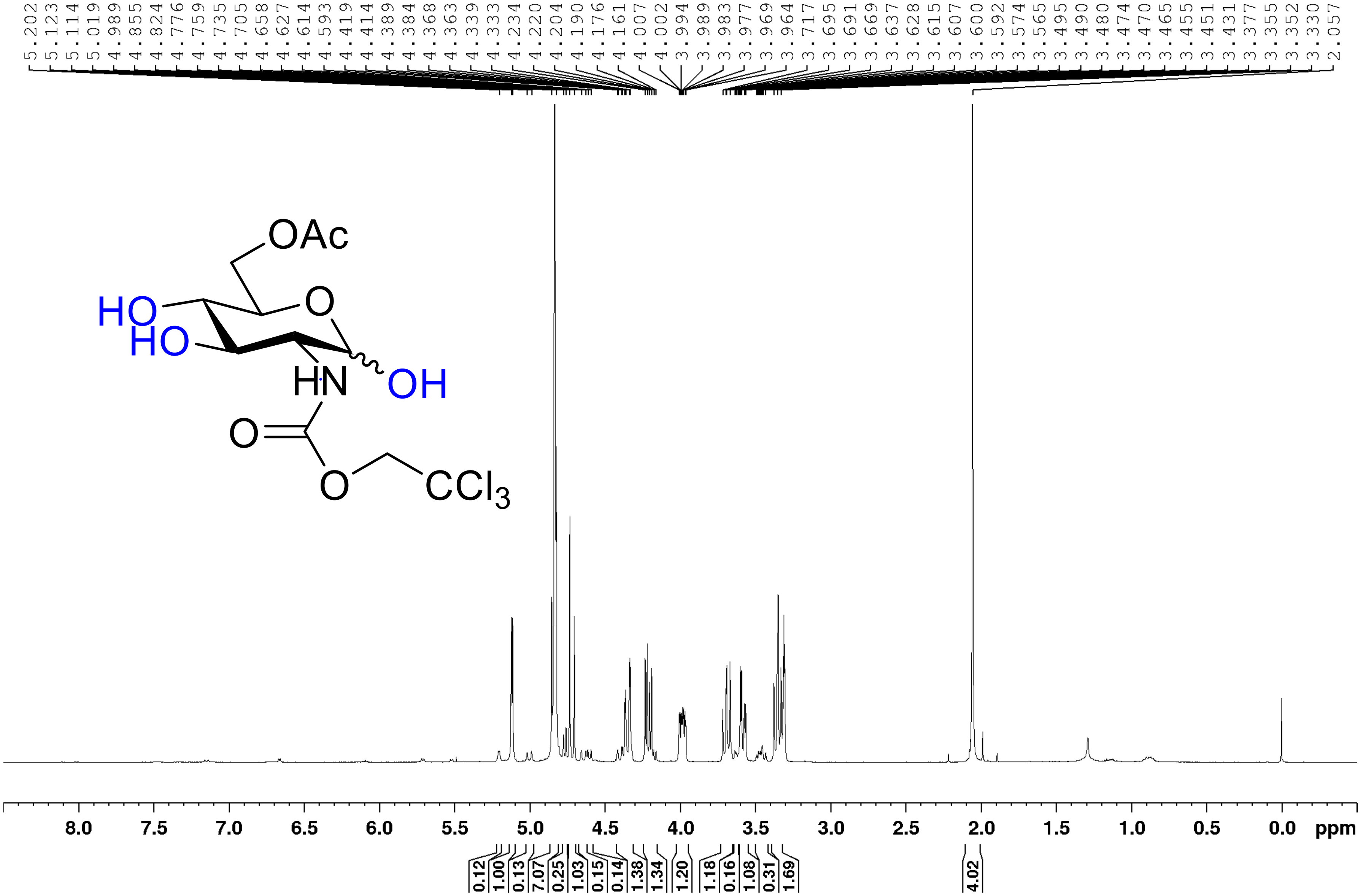

Figure S94: 6-*O*-acetyl-2-deoxy-2-(2,2,2-trichloroethoxycarbonylamino)-D-glucopyranose (29) ^{1}H-^{1}H COSY NMR (400 MHz) in MeOD

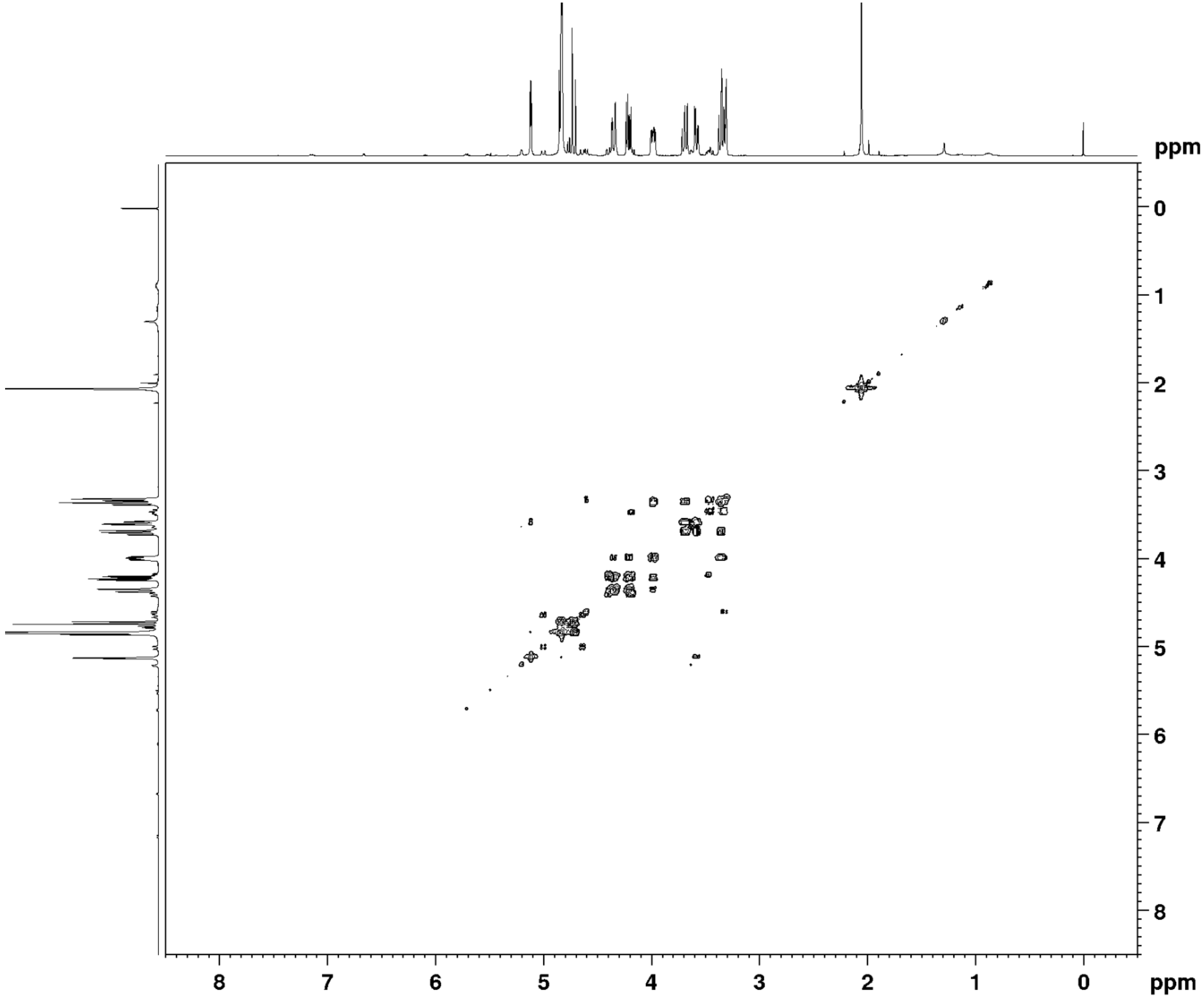

Figure S95: 6-*O*-acetyl-2-deoxy-2-(2,2,2-trichloroethoxycarbonylamino)-D-glucopyranose (**29**) ^{1}H-$^{13}C\{^{1}H\}$ HSQC NMR (400 & 101 MHz) in MeOD

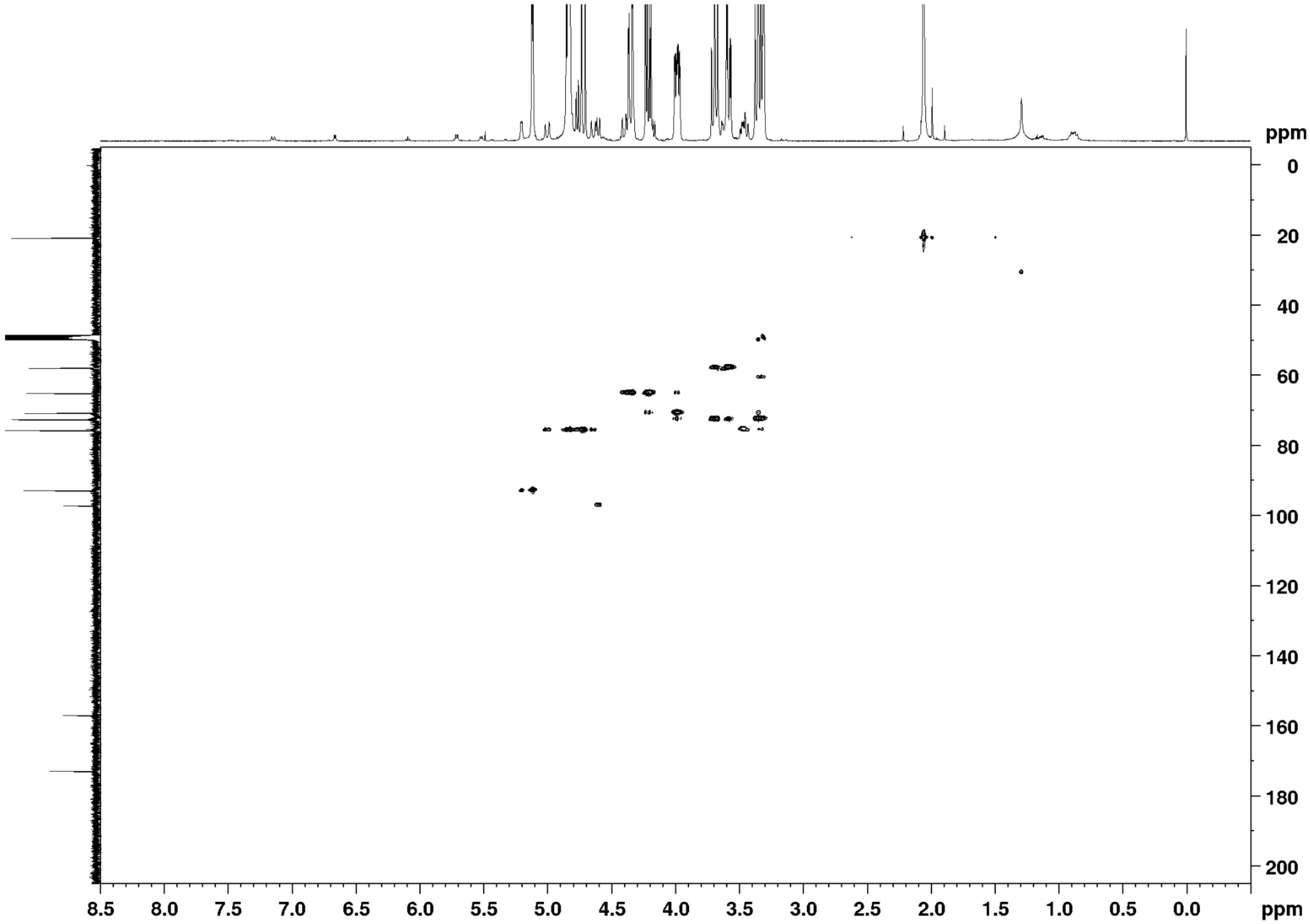

Figure S96: 6-*O*-acetyl-2-deoxy-2-(2,2,2-trichloroethoxycarbonylamino)-D-glucopyranose (29) ^{1}H-$^{13}C\{^{1}H\}$ HMBC NMR (400 & 101 MHz) in MeOD

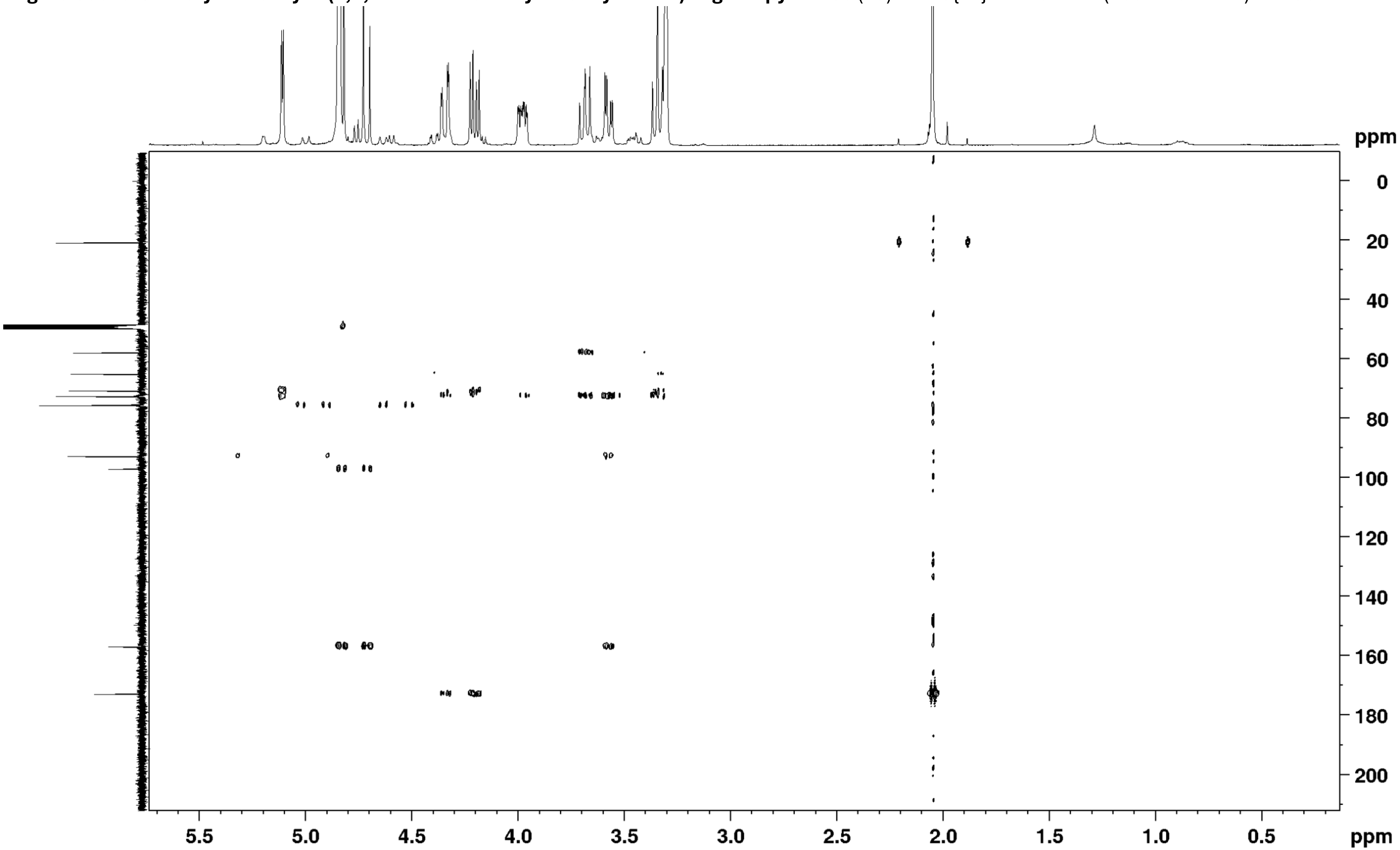

Figure S97: 6-*O*-acetyl-2-deoxy-2-(2,2,2-trichloroethoxycarbonylamino)-D-glucopyranose (29) $^{13}C\{^{1}H\}$ NMR (101 MHz) in MeOD

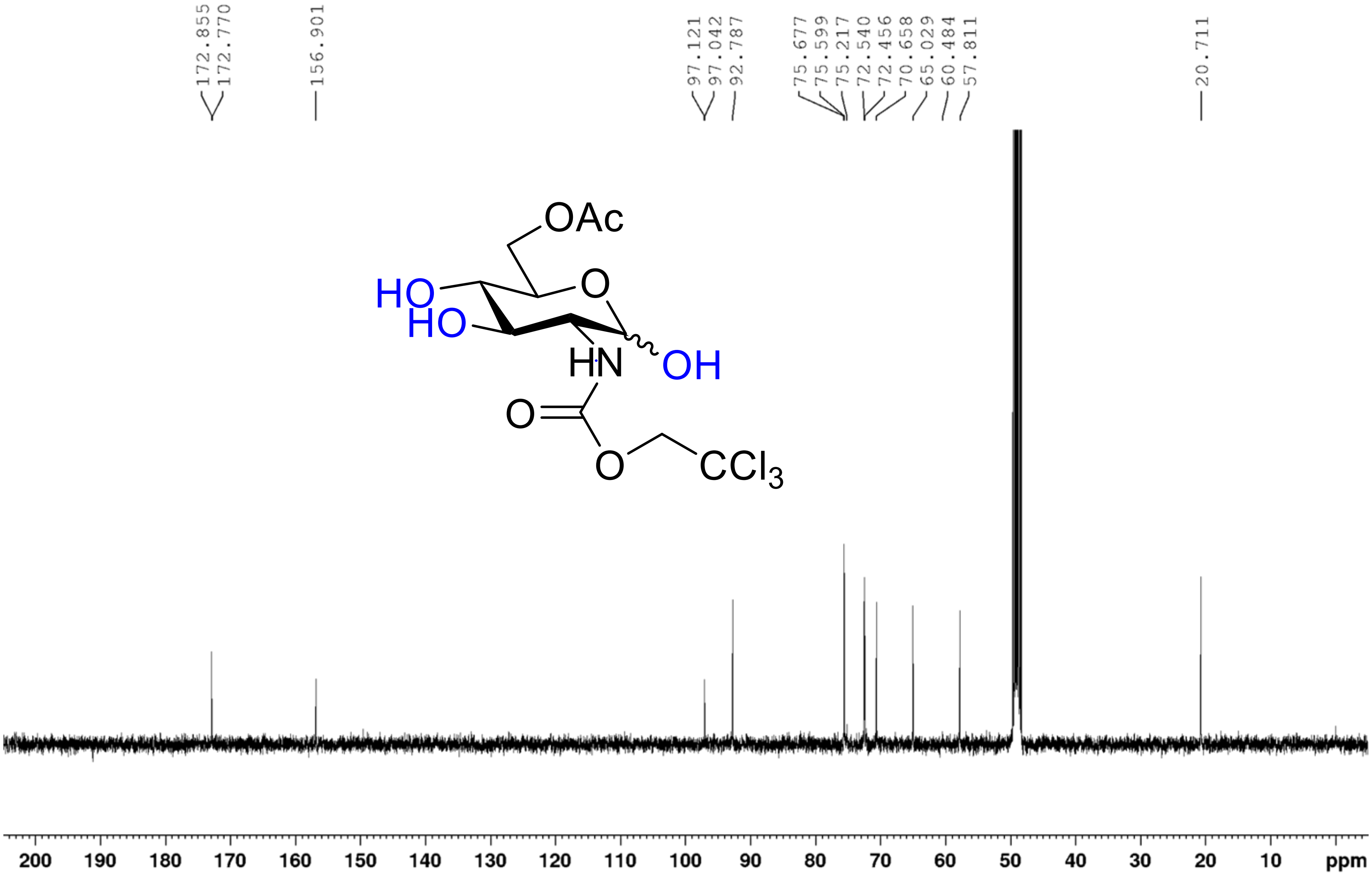

Figure S98: Mixture, where **1,6-di-*O*-acetyl-D-mannopyranoside (31)** is shown ^{1}H NMR (400 MHz) in MeOD

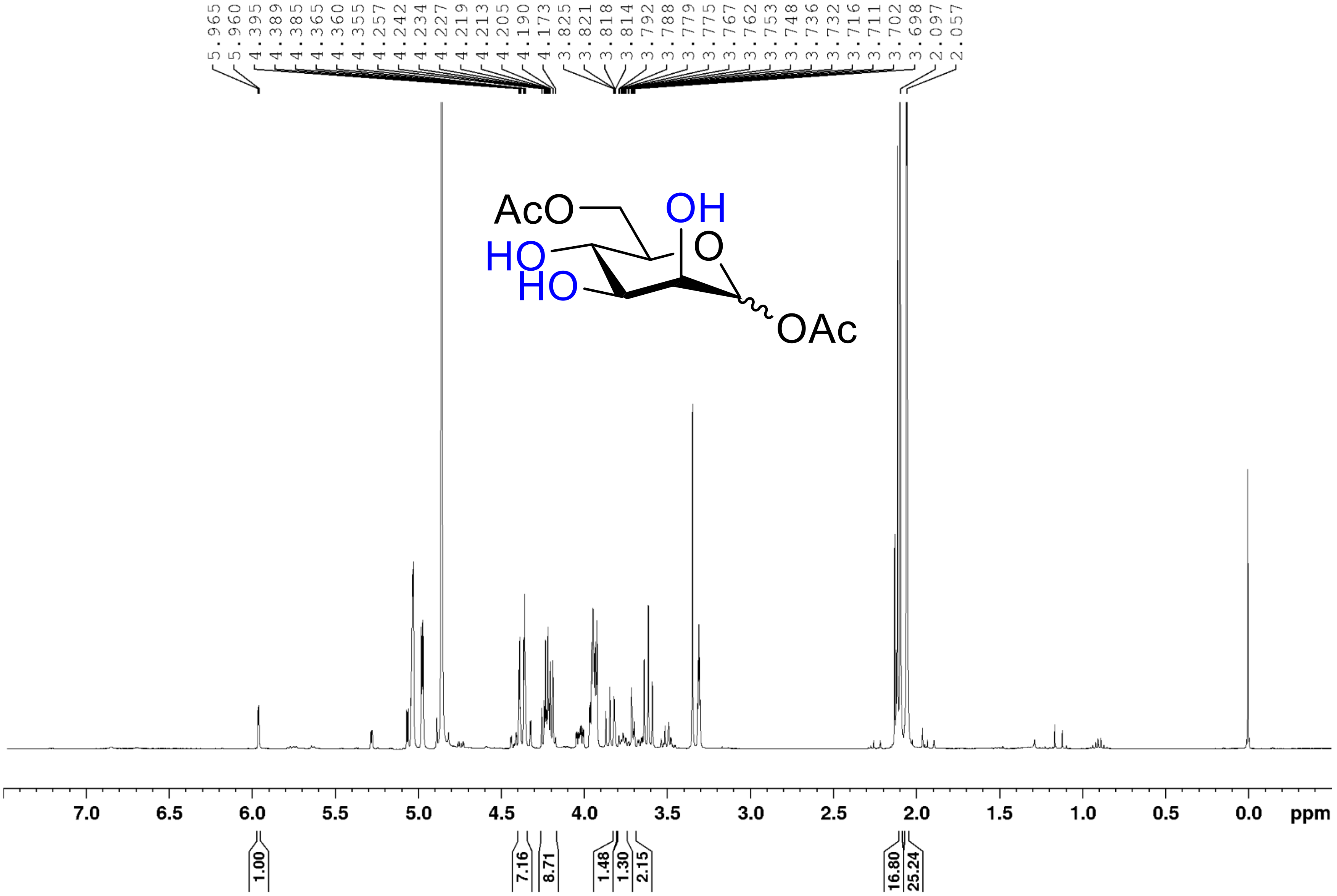

Figure S99: Mixture of **1,6/2,6/3,6-di-*O*-acetyl-D-mannopyranoside** (**31-33**) ^{1}H-^{1}H COSY NMR (400 MHz) in MeOD

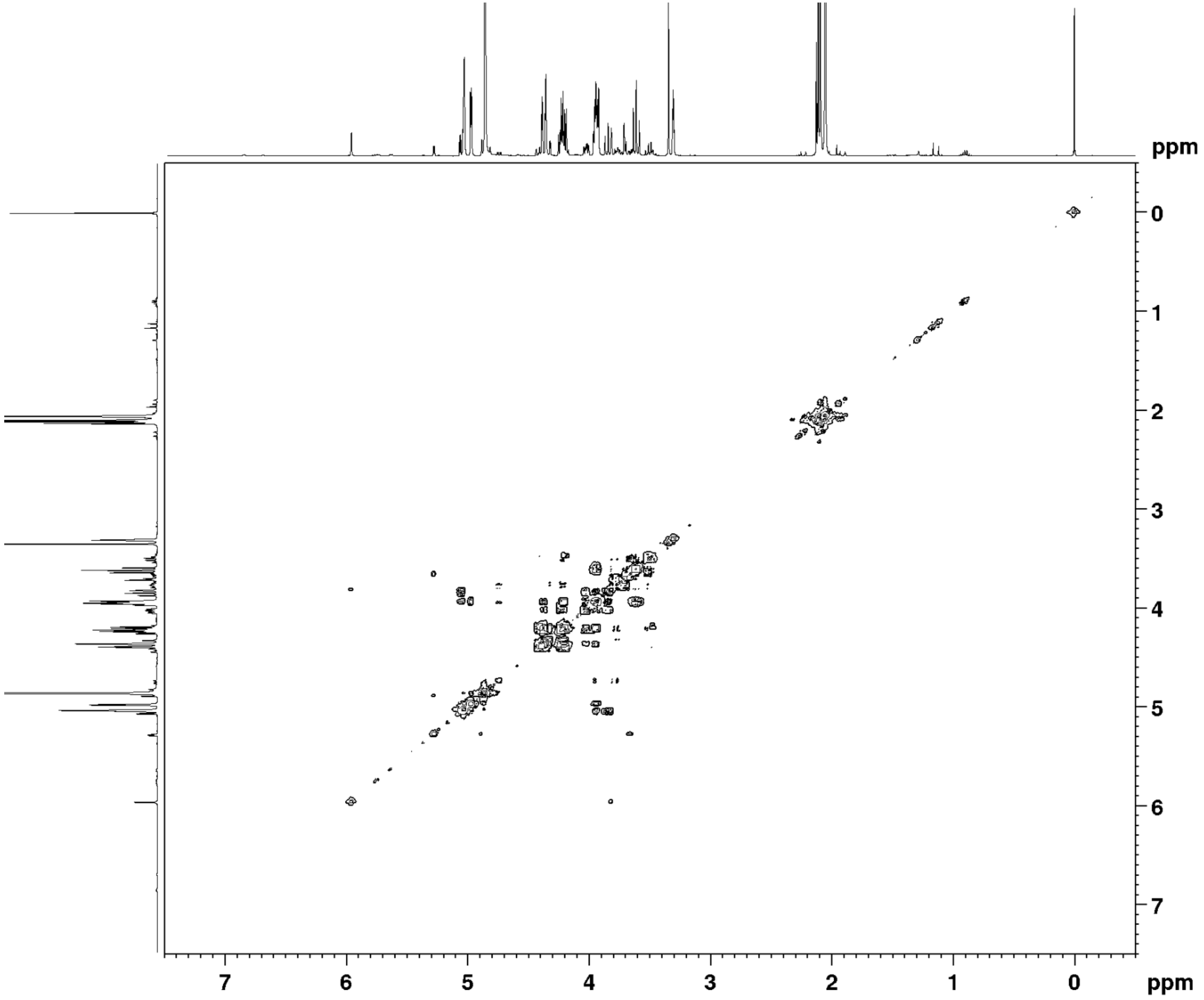

Figure S100: Mixture of **1,6/2,6/3,6-di-*O*-acetyl-D-mannopyranoside** (**31-33**) ^{1}H-$^{13}C\{^{1}H\}$ HSQC NMR (400 & 101 MHz) in MeOD

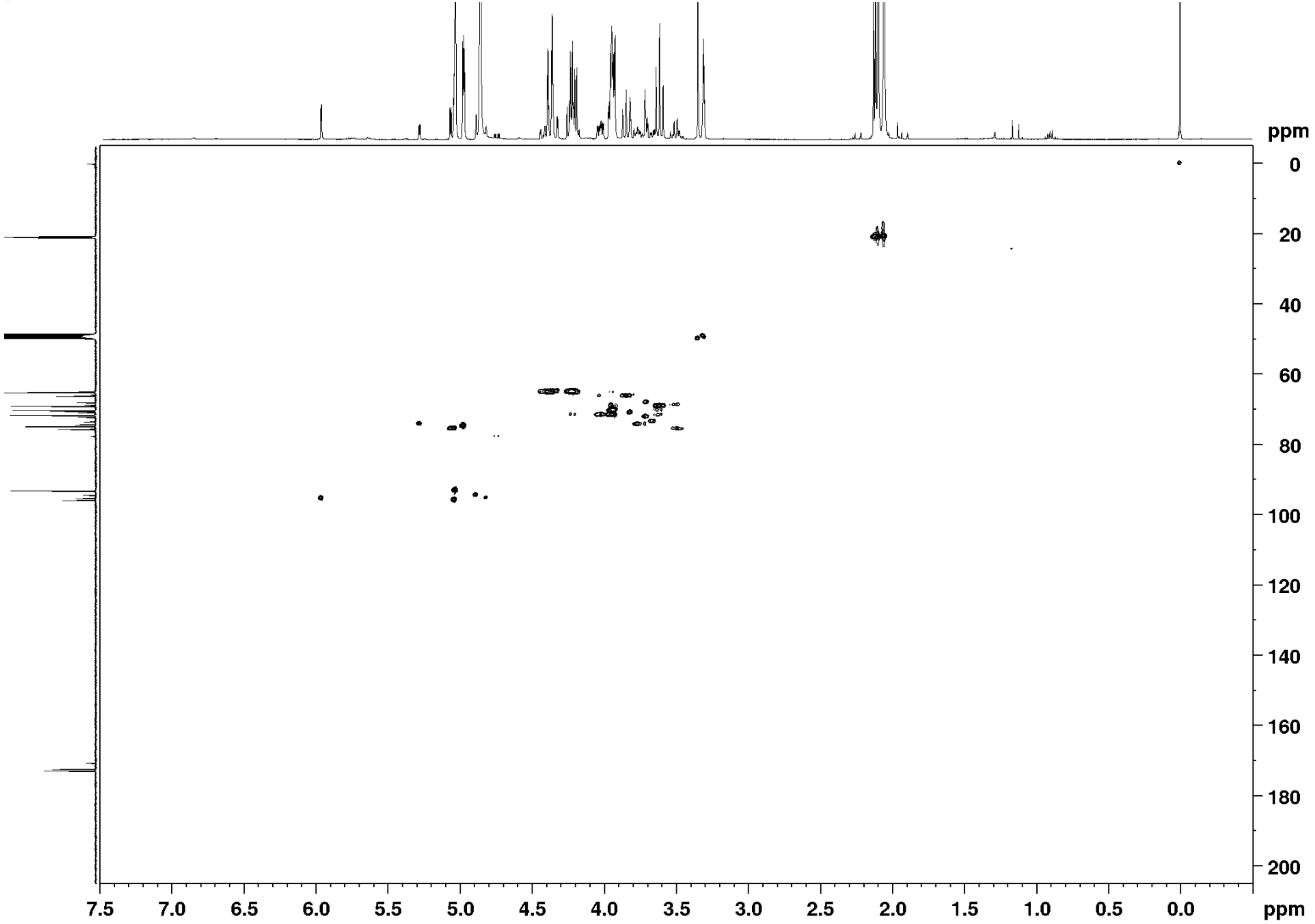

Figure S101: Mixture of **1,6/2,6/3,6-di-*O*-acetyl-D-mannopyranoside** (**31-33**) ^{1}H-$^{13}C\{^{1}H\}$ HMBC NMR (400 & 101 MHz) in MeOD

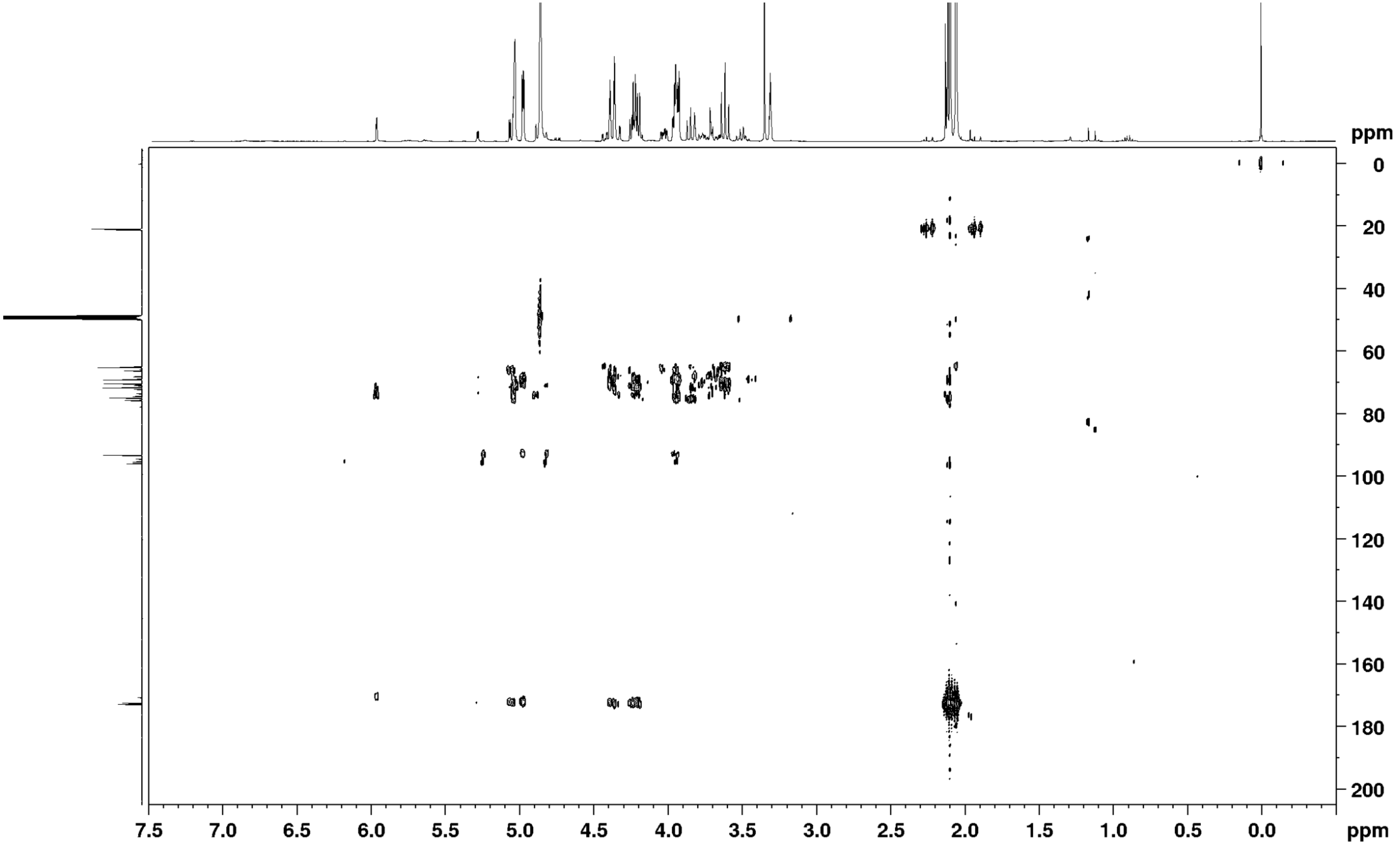

Figure S102: Mixture, where **1,6-di-*O*-acetyl-D-mannopyranoside** (**31**) is shown $^{13}C\{^{1}H\}$ NMR (101 MHz) in MeOD

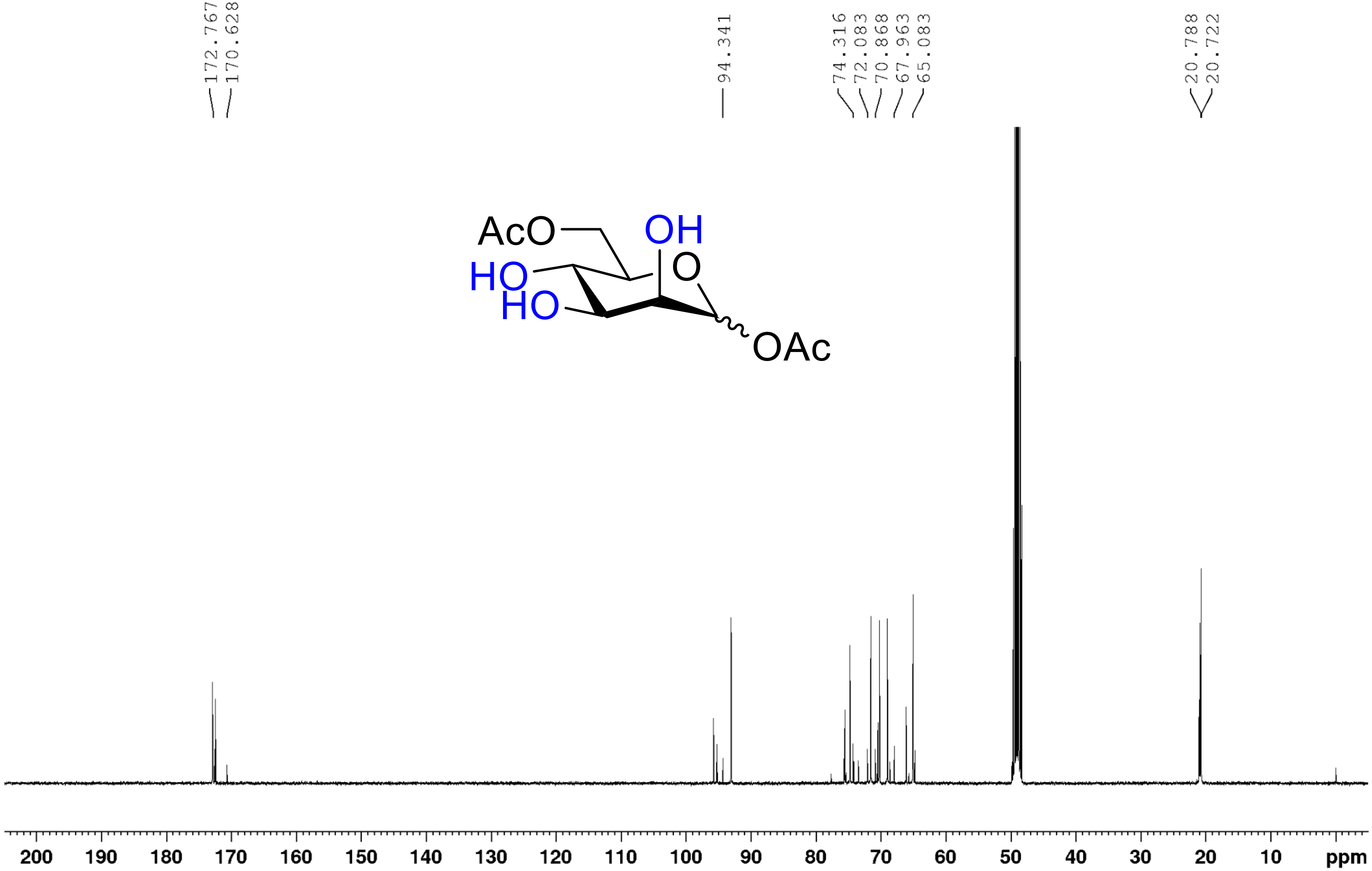

Figure S103: Mixture, where **2,6-di-*O*-acetyl-D-mannopyranose (32)** is shown α:β 12:88 ^{1}H NMR (400 MHz) in MeOD

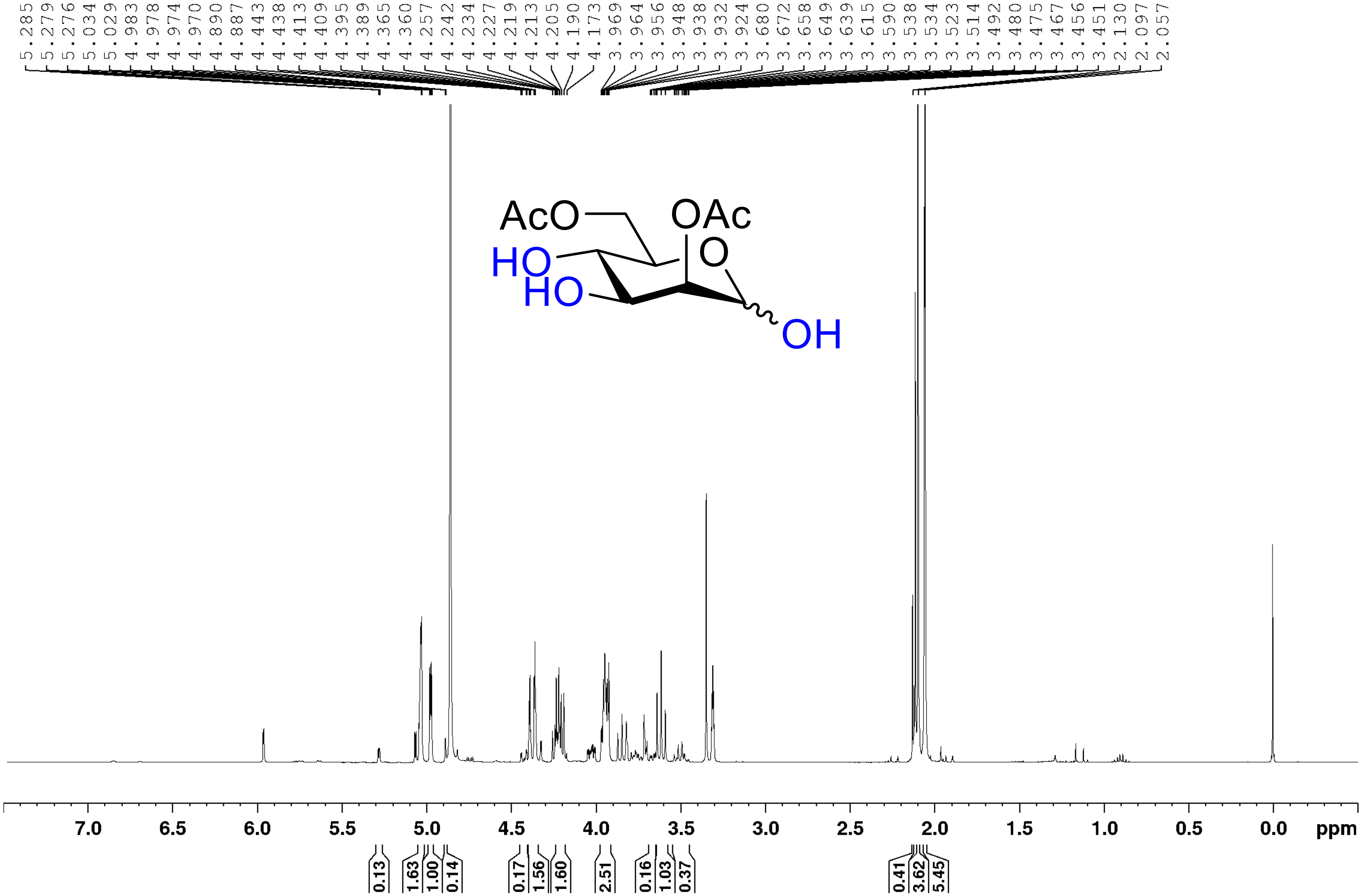

Figure S104: Mixture, where **2,6-di-*O*-acetyl-D-mannopyranose** (**32**) is shown α:β 12:88 $^{13}C\{^1H\}$ NMR (101 MHz) in MeOD

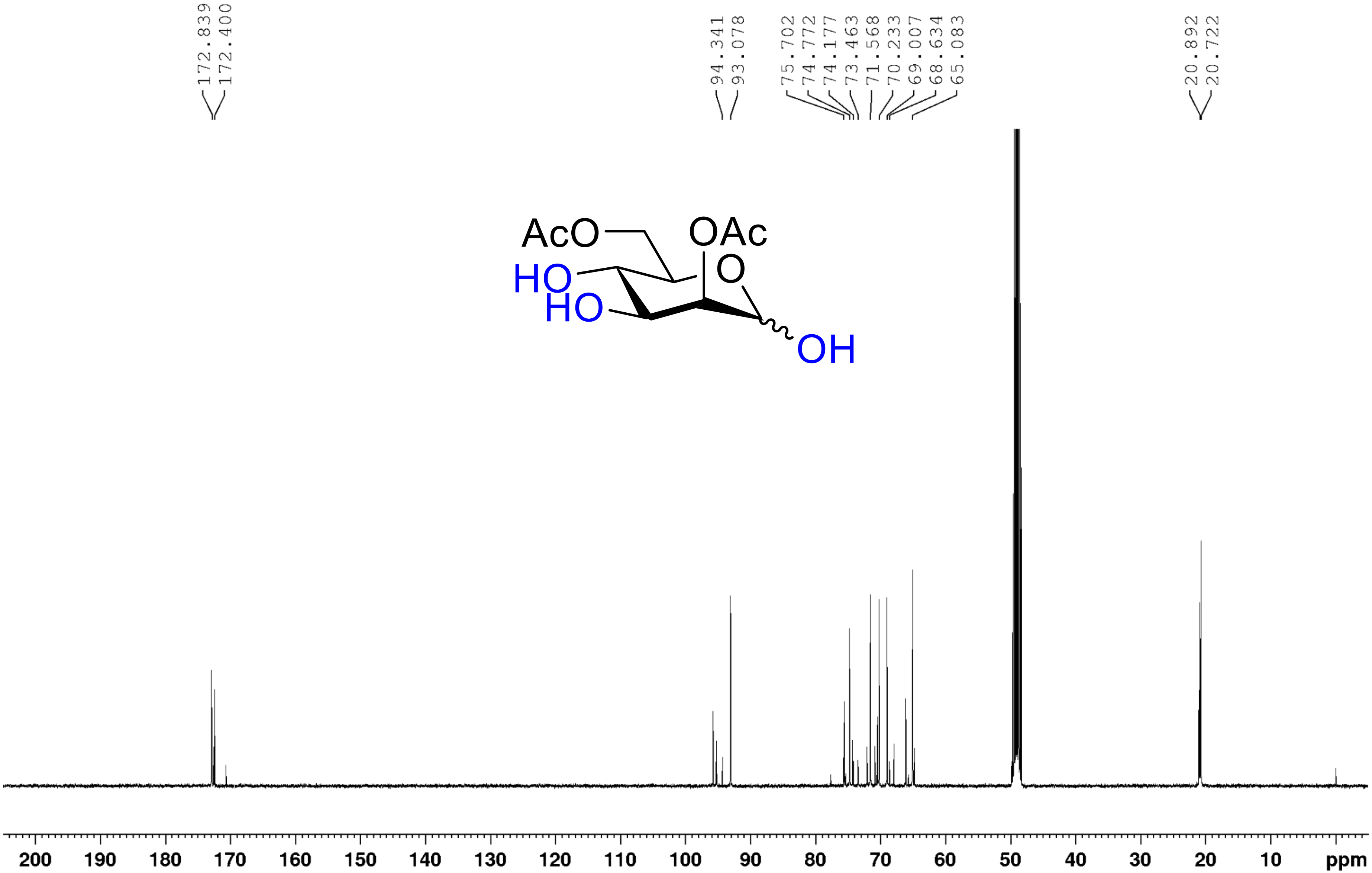

Figure S105: Mixture, where **3,6-di-*O*-acetyl-D-mannopyranose (33)** is shown ^{1}H NMR (400 MHz) in MeOD

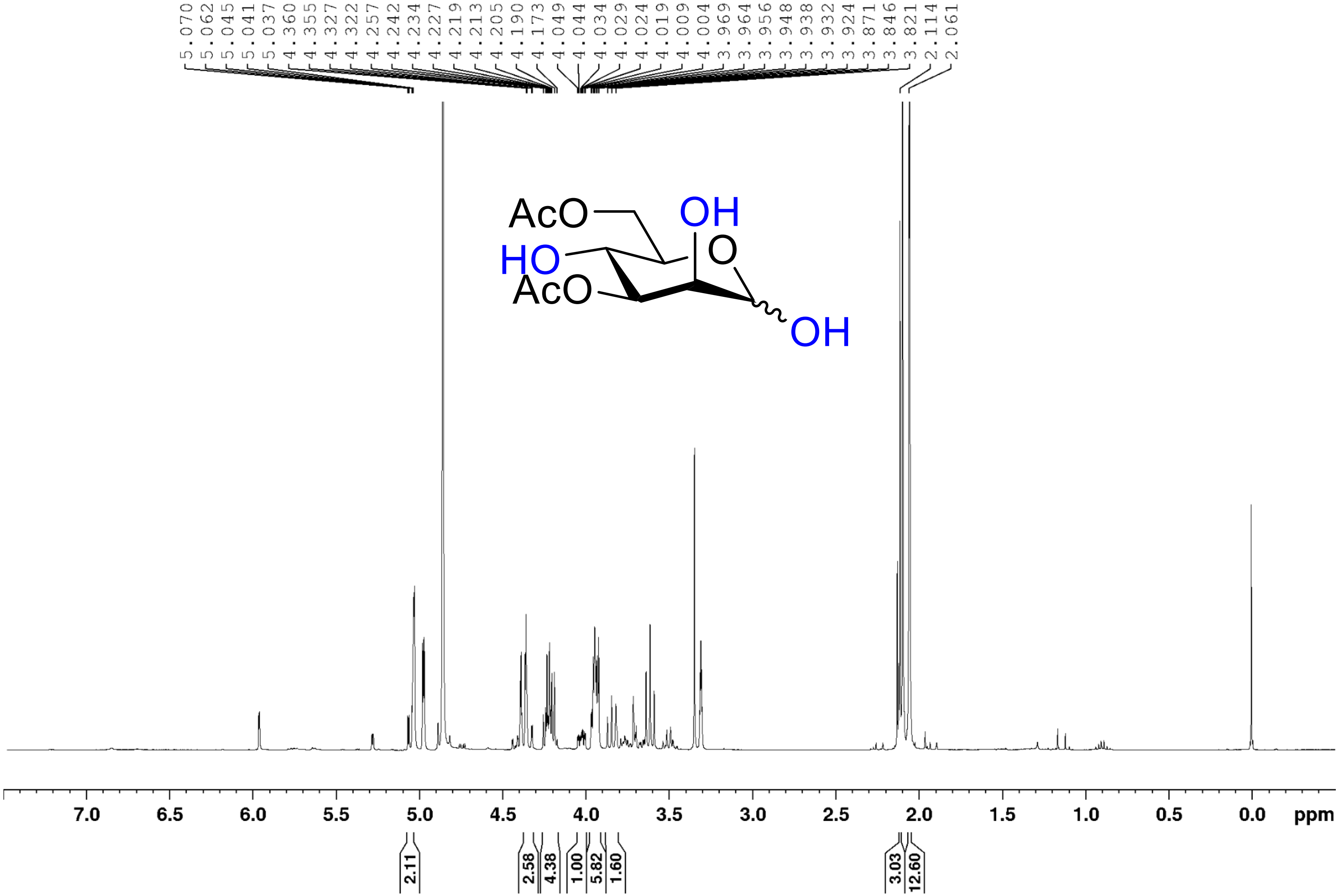

Figure S106: Mixture, where **3,6-di-*O*-acetyl-D-mannopyranose** (**33**) is shown $^{13}C\{^1H\}$ NMR (101 MHz) in MeOD

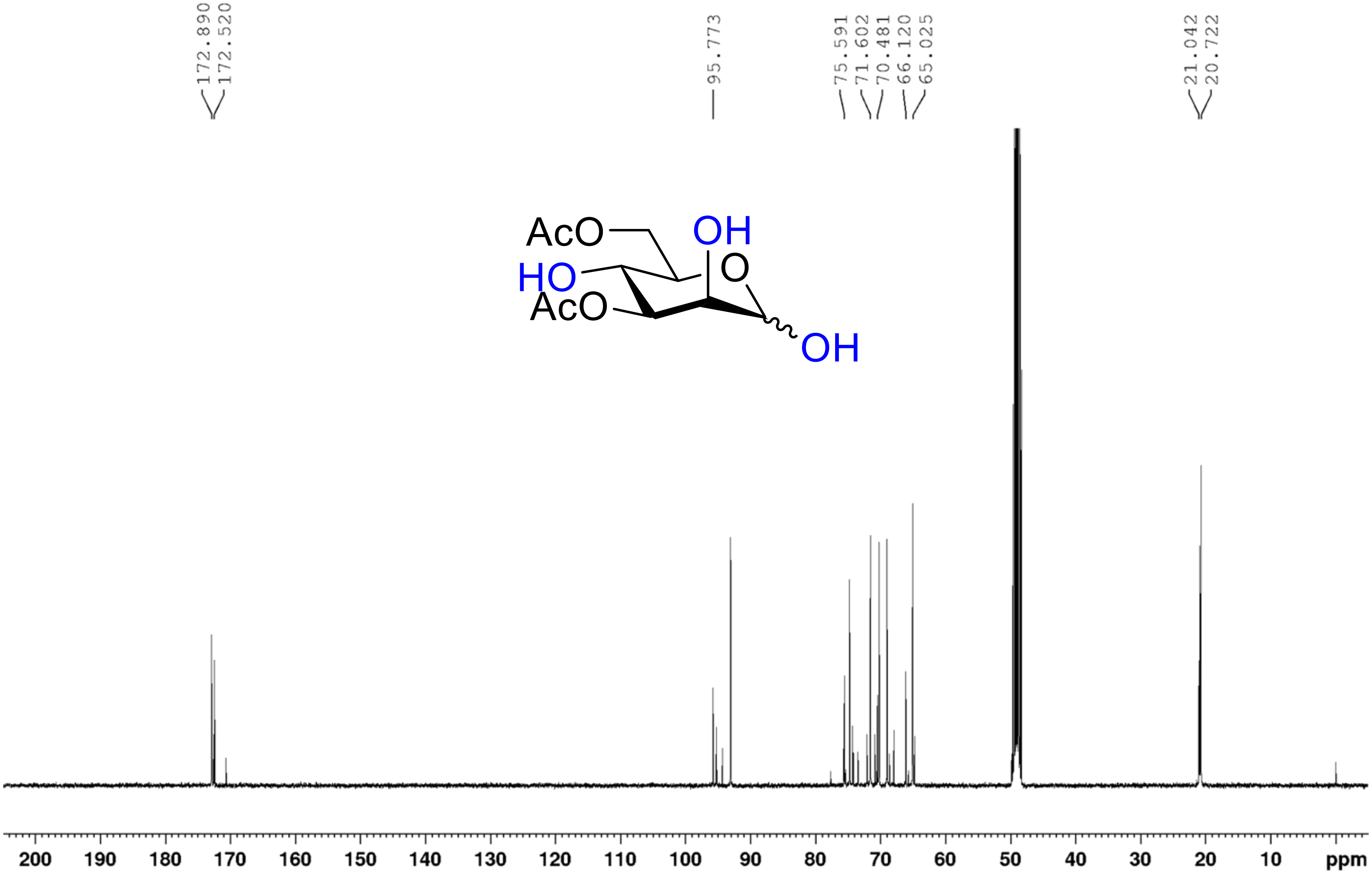

Figure S107: 4-*O*-acetyl-6-deoxy-L-mannopyranose (**35**) ^{1}H NMR (400 MHz) in MeOD

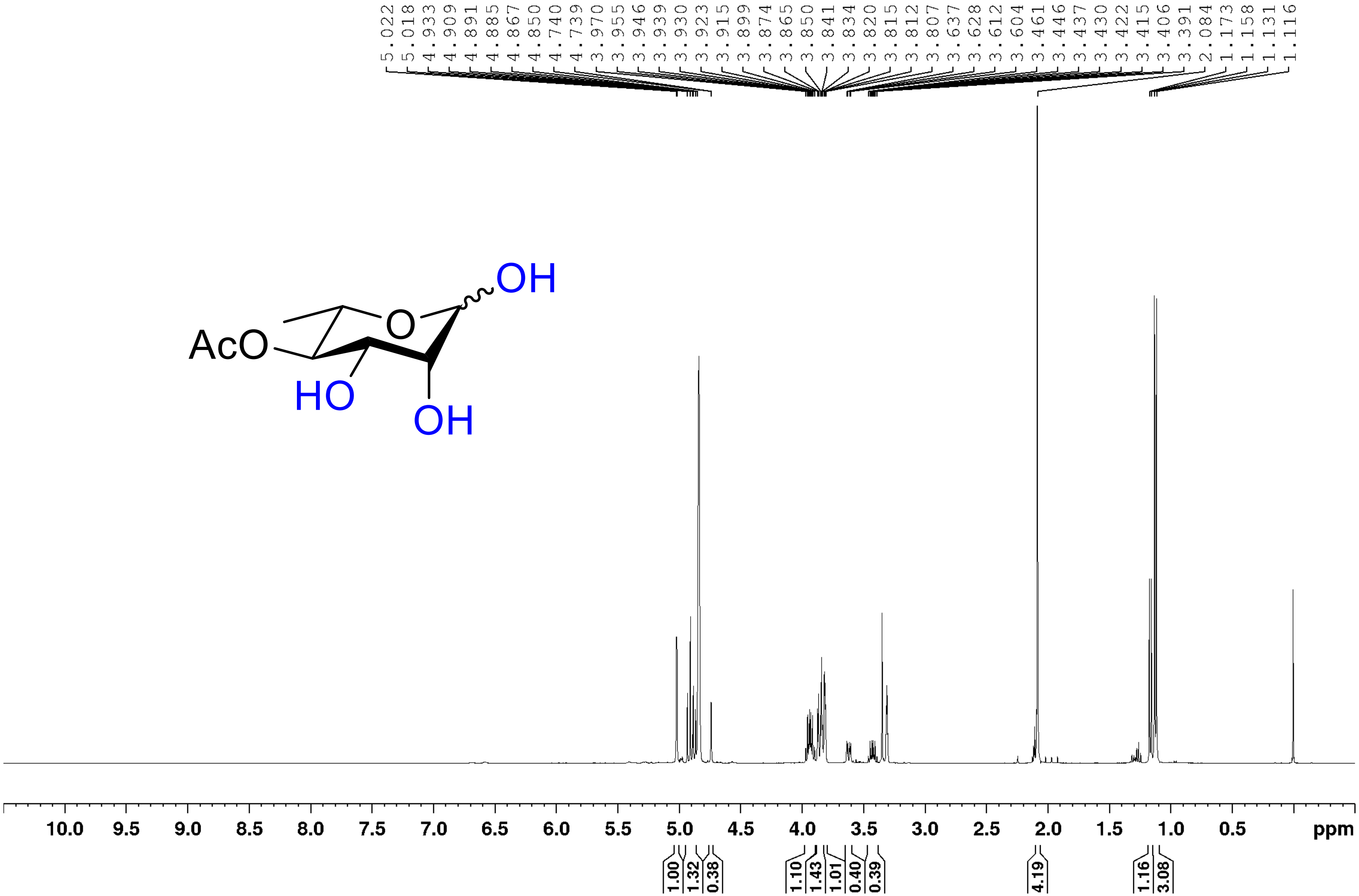

Figure S108: 4-*O*-acetyl-6-deoxy-L-mannopyranose (**35**) ^{1}H-^{1}H COSY NMR (400 MHz) in MeOD

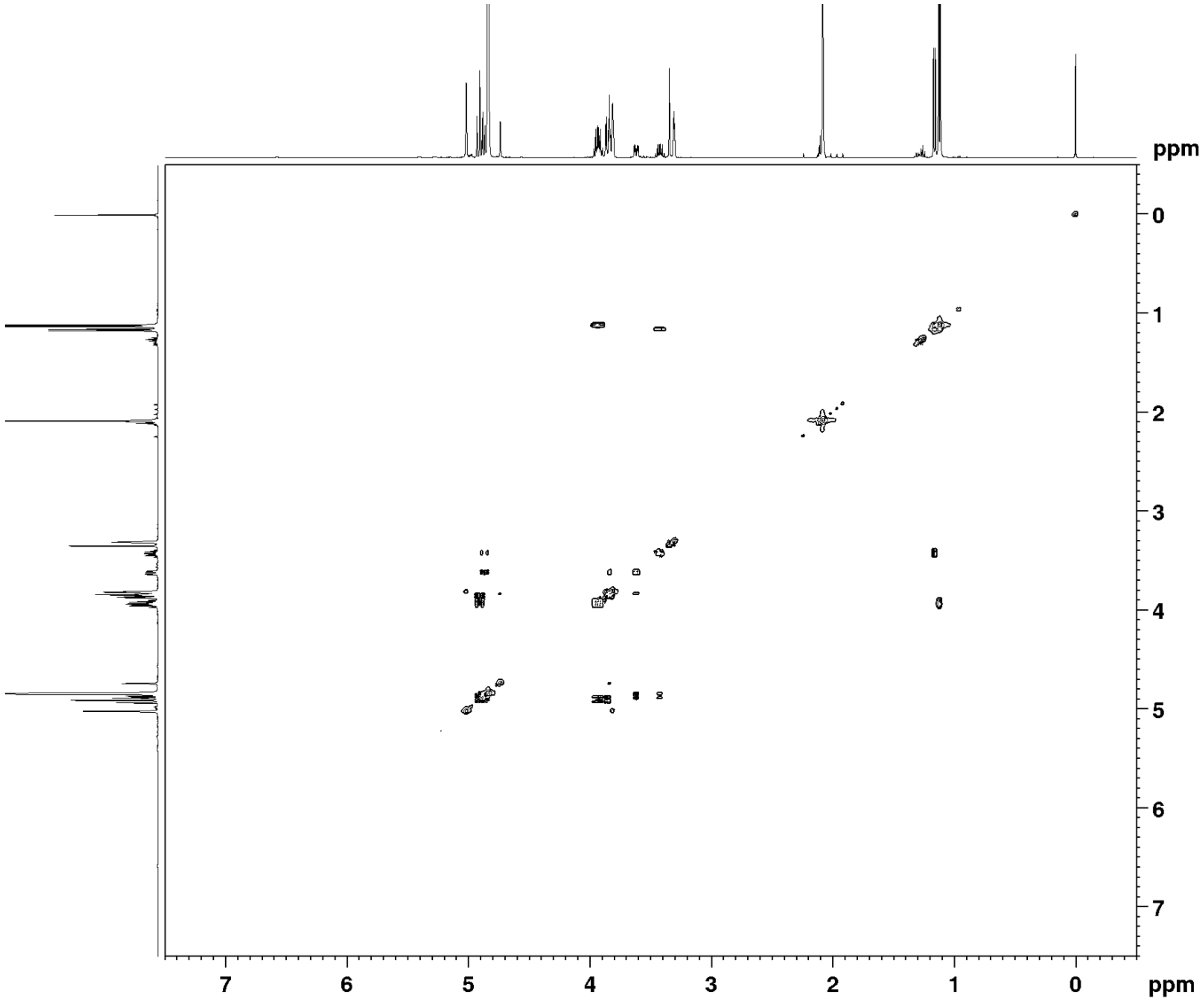

Figure S109: 4-*O*-acetyl-6-deoxy-L-mannopyranose (**35**) ^{1}H-^{13}C{^{1}H} HSQC NMR (400 & 101 MHz) in MeOD

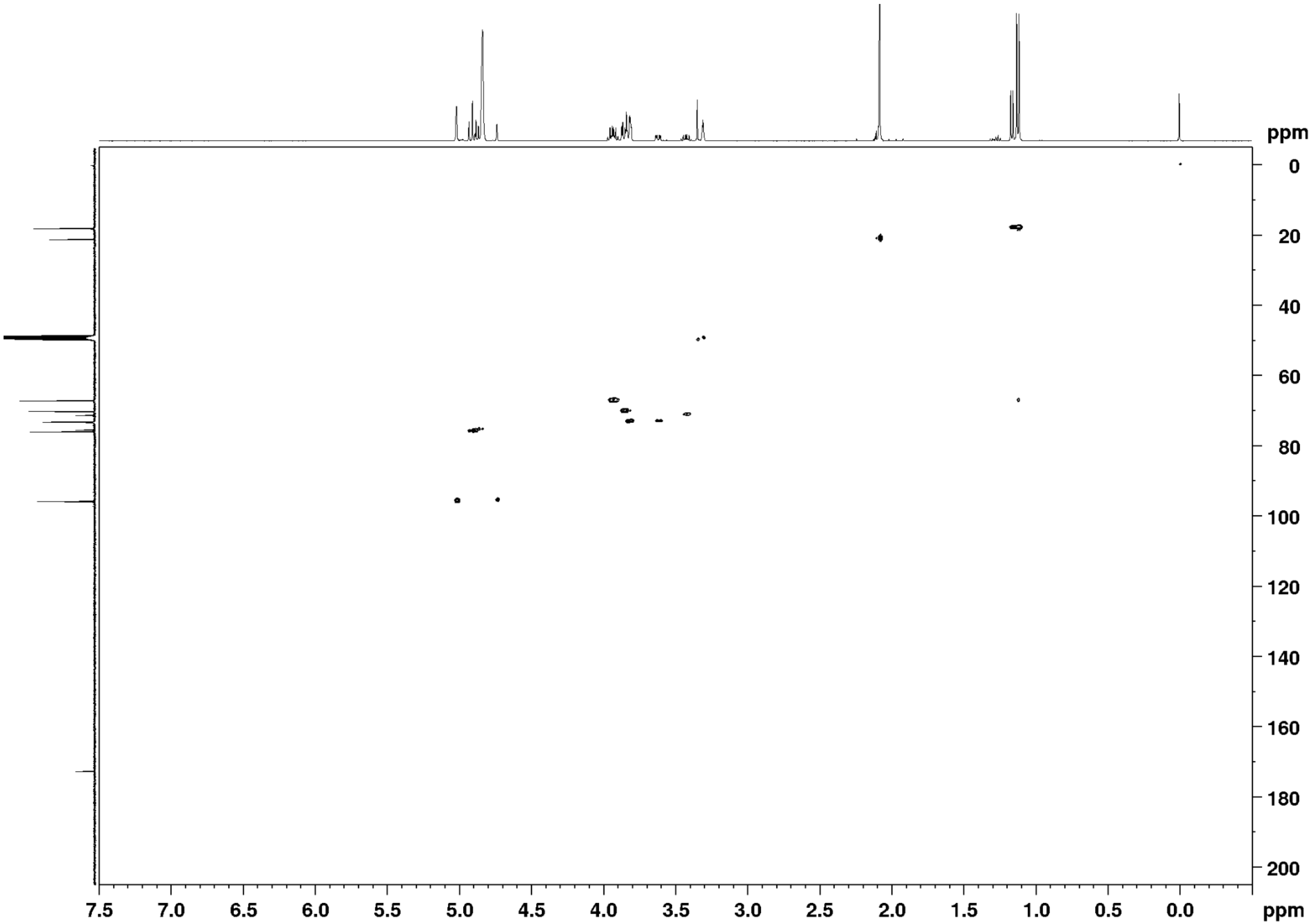

Figure S110: 4-*O*-acetyl-6-deoxy-L-mannopyranose (**35**) ^{1}H-$^{13}C\{^{1}H\}$ HMBC NMR (400 & 101 MHz) in MeOD

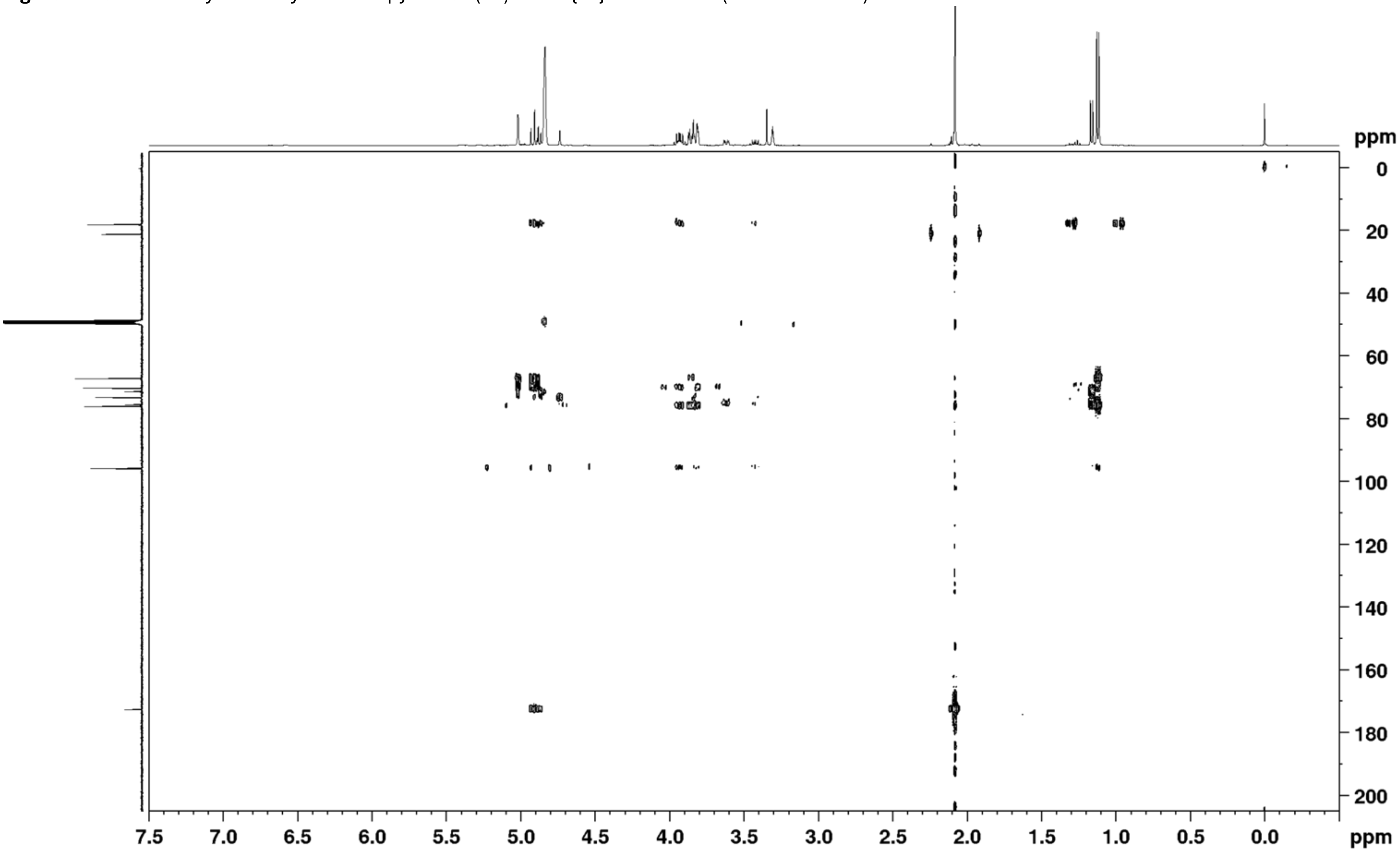

References

(1) Hunt, K. E.; García-Sosa, A. T.; Shalima, T.; Maran, U.; Vilu, R.; Kanger, T. Synthesis of 6'-Galactosyllactose, a Deviant Human Milk Oligosaccharide, with the Aid of *Candida Antarctica* Lipase-B. *Org. Biomol. Chem.* **2022**, *20* (23), 4724–4735. https://doi.org/10.1039/D2OB00550F.

(2) Braun, G. CELLOBIOSE. *Organic Syntheses* **1937**, *17*, 34. https://doi.org/10.15227/orgsyn.017.0034.

(3) Janczuk, A. J.; Zhang, W.; Andreana, P. R.; Warrick, J.; Wang, P. G. The Synthesis of Deoxy-α-Gal Epitope Derivatives for the Evaluation of an Anti-α-Gal Antibody Binding. *Carbohydr. Res.* **2002**, *337* (14), 1247–1259. https://doi.org/10.1016/S0008-6215(02)00159-3.

(4) Zhang, P.; Ng, K.; Ling, C.-C. Total Synthesis of LeA-LacNAc Pentasaccharide as a Ligand for Clostridium Difficiletoxin A. *Org. Biomol. Chem.* **2010**, *8* (1), 128–136. https://doi.org/10.1039/B914193F.

(5) Li, Z.; Gildersleeve, J. C. Mechanistic Studies and Methods To Prevent Aglycon Transfer of Thioglycosides. *J. Am. Chem. Soc.* **2006**, *128* (35), 11612–11619. https://doi.org/10.1021/ja063247q.

(6) Deore, B.; Ocando, J. E.; Pham, L. D.; Sanhueza, C. A. Anodic Reactivity of Alkyl *S* - Glucosides. *J. Org. Chem.* **2022**, *87* (9), 5952–5960. https://doi.org/10.1021/acs.joc.2c00222.

(7) Stubbs, K. A.; Macauley, M. S.; Vocadlo, D. J. A Highly Concise Preparation of O-Deacetylated Arylthioglycosides of N-Acetyl-d-Glucosamine from 2-Acetamido-3,4,6-Tri-O-Acetyl-2-Deoxy-α-d-Glucopyranosyl Chloride and Aryl Thiols or Disulfides. *Carbohydr. Res.* **2006**, *341* (10), 1764–1769. https://doi.org/10.1016/j.carres.2005.12.009.

(8) Balmond, E. I.; Benito-Alifonso, D.; Coe, D. M.; Alder, R. W.; McGarrigle, E. M.; Galan, M. C. A 3,4- *Trans* -Fused Cyclic Protecting Group Facilitates Α-Selective Catalytic Synthesis of 2-Deoxyglycosides. *Angew. Chem. Int. Ed.* **2014**, *53* (31), 8190–8194. https://doi.org/10.1002/anie.201403543.

(9) Cirla, A.; McHale, A. R.; Mann, J. Synthesis of Analogues of Calicheamicin and Neocarzinostatin Chromophore. *Tetrahedron* **2004**, *60* (18), 4019–4029. https://doi.org/10.1016/j.tet.2004.03.021.

(10) Hackbusch, S.; Watson, A.; Franz, A. H. Development of a Karplus Equation for 3JCOCH in Ester-Functionalized Glucopyranoses and Methylglucuronate. *Arkivoc* **2017**, *2017* (5), 268–292. https://doi.org/10.24820/ark.5550190.p010.113.

(11) Look, G. C.; Ichikawa, Y.; Shen, G. J.; Cheng, P. W.; Wong, C. H. A Combined Chemical and Enzymatic Strategy for the Construction of Carbohydrate-Containing Antigen Core Units. *J. Org. Chem.* **1993**, *58* (16), 4326–4330. https://doi.org/10.1021/jo00068a030.
